# RAFT enables controlled radical ring-opening polymerisation of cyclic ketene acetals for degradable nanoparticles

**DOI:** 10.1038/s42004-026-01997-6

**Published:** 2026-04-09

**Authors:** Fabian Mehner, Aniket R. Bukane, Daniel J. Keddie, Martin Geisler, Albena Lederer, Brigitte Voit, Jens Gaitzsch

**Affiliations:** 1 Leibniz-Institut für Polymerforschung Dresden e.V, Dresden, Germany; 2 Organic Chemistry of Polymers, TUD Dresden University of Technology, Dresden, Germany; 3 School of Chemistry, University of Nottingham, Nottingham, UK; 4 Department Chemistry and Polymer Science, Stellenbosch University, Stellenbosch, South Africa

**Keywords:** Polymer chemistry, Molecular self-assembly, Synthesis and processing, Polymer synthesis, Synthetic chemistry methodology

## Abstract

Following the demand for biodegradable polymers in biomedicine and cosmetics, the radical ring-opening polymerisation (RROP) of cyclic ketene acetals (CKAs) offers a robust synthesis approach to prepare a wide range of polyesters. Here we demonstrate a method to control the RROP of 2-methylene-1,3,6-trioxocane (MTC) in terms of molar mass, dispersity, end-group control and kinetics using the reversible addition and fragmentation chain transfer polymerisation (RAFT) methodology. Fine-tuning the ratio between the reactants allows for a better understanding of the interplay of RAFT and RROP. With so optimised reaction conditions, a well-defined PMTC-macroinitiator was obtained and a chain extension with MTC and 2-methylene-1,3-dioxepane (MDO) was applied to form P(CKA-b-CKA) block copolymers. These were then formulated into fully biodegradable polymeric nanoparticles with tuneable degradation time. This work hence pushes the boundaries of RROP towards a whole expanded range of defined homopolymers and fully CKA-based block-copolymers as well as their completely biodegradable nanoparticles. This controlled RROP opens more areas of application for RROP-based polyesters.

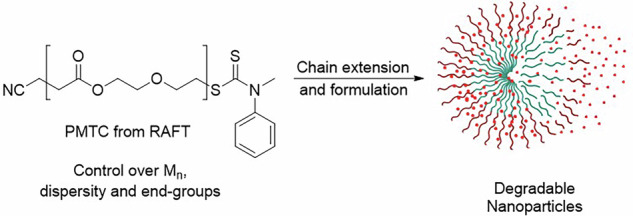

## Abstract (web summary)

The demand for biodegradable polymers in biomedicine and cosmetics drives interest in the radical ring-opening polymerisation (RROP) of cyclic ketene acetals (CKAs). Here, the authors demonstrate a controlled polymerisation method using RAFT to control the RROP of 2-methylene-1,3,6-trioxocane, enabling the creation of well-defined, fully biodegradable polymeric nanoparticles.

## Introduction

**Fig. 1:**
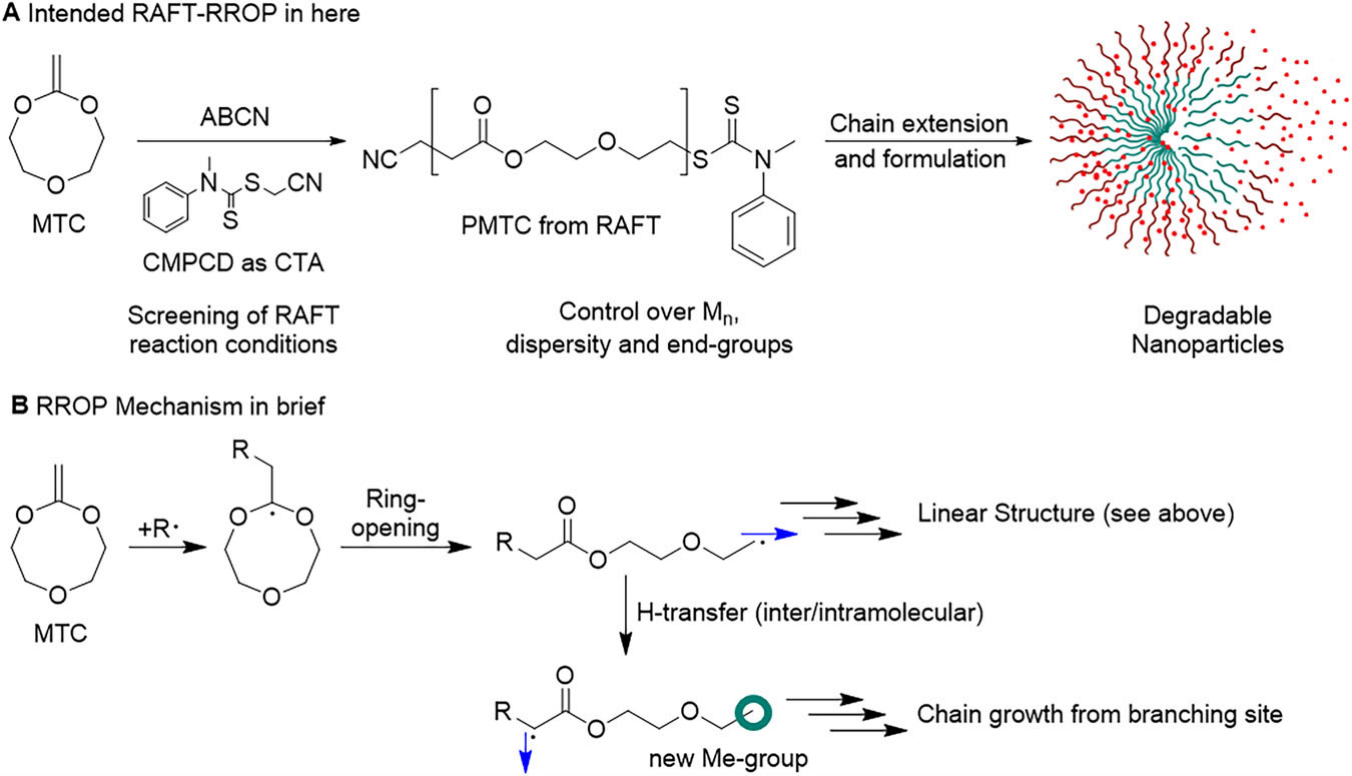
Indended RAFT polymerisation of MTC and polymerisation mechanism. **A** In this study, MTC gets polymerised using the shown RAFT agent in different reaction conditions, where CPMCD acts as CTA and ABCN as initiator. RAFT can be considered a controlled radical polymerisation, if control over molar mass, dispersity and end-groups can be established. The final polymer then gets chain-extended into block-copolymers, which form dye-loaded fully degradable NPs upon self-assembly. **B** In the RROP mechanism, the exocyclic double bond of the monomer (here: MTC) gets attacked by a radical and the ring subsequently opens towards the propagating radical. This can lead to the main linear structure, but also to a branched structure if inter- or intramolecular H-transfer occurs. Every H-transfer leads to an additional methyl group (green circle). Chain growth continues from the position of the radical as indicated by the blue arrows.

**Fig. 2:**
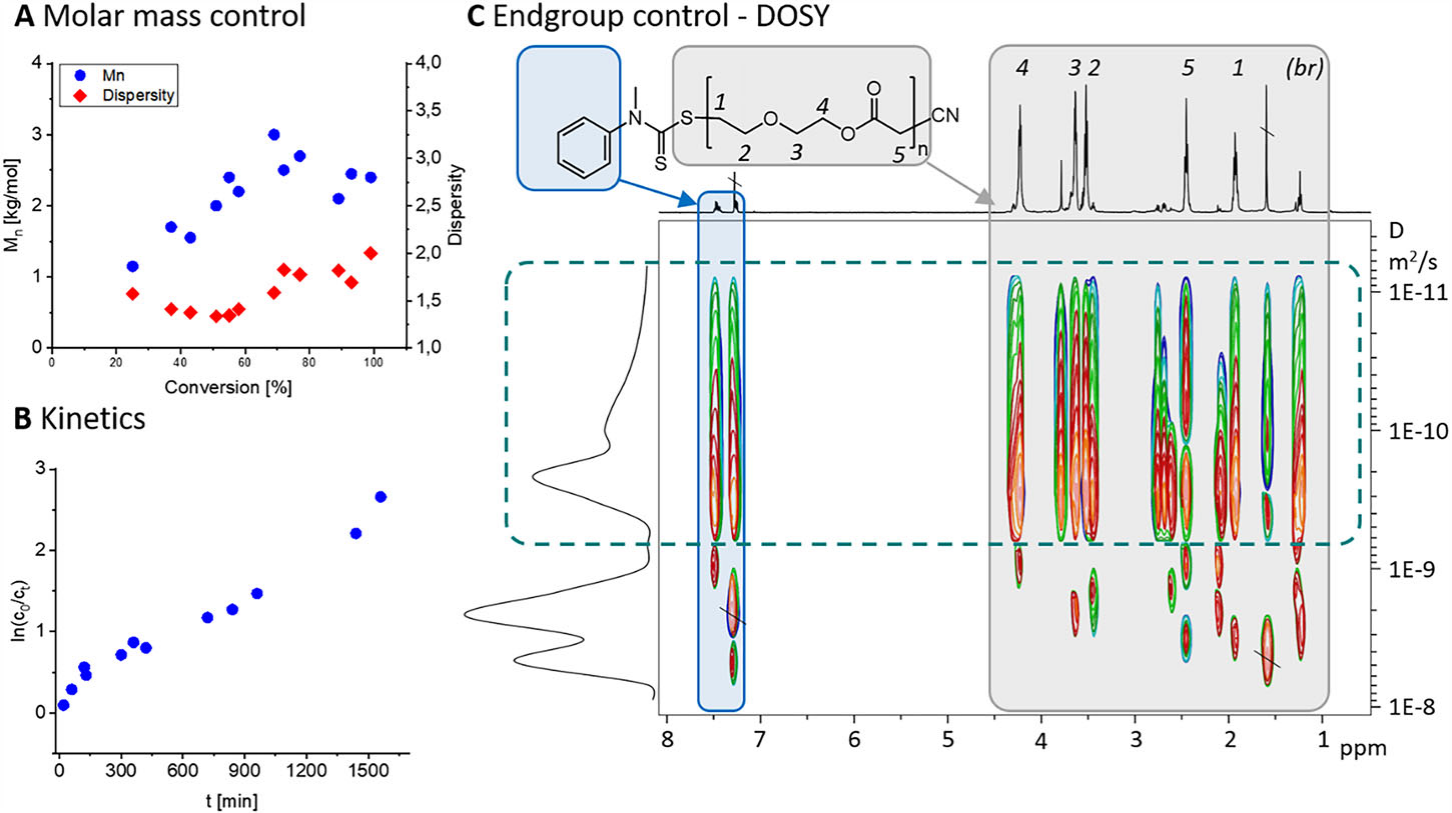
Proof of the controlled character of the RROP of MTC with PMTC-B-1:1-5.0. **A** Development of molar mass and dispersity over conversion, **B** pseudo first-order kinetics plot and **C** end group control shown by Diffusion ordered nuclear magnetic resonance spectroscopy (DOSY) on PMTC-B-1:1-10.0, reconstructed with inverse-Laplace-transform (ILT). The blue square highlights the aromatic protons stemming from the CMPCD and the grey square highlights the signals originating from the PMTC, incl. signal annotation 1-5 of the main signals ((br) belongs to methyl end group stemming from branching). Both are present on a molecule with the same diffusion coefficient. For clarity, small diffusion traces below the level of the dashed red line in the vertical dimension are not shown.

In order to move to a more sustainable future in polymer science, new biodegradable polymer materials with a broad range of tuneable properties have to be developed^1,2^. Targeting the utilisation in high-end applications, future materials have to have a well-defined structure and their synthesis has to be robust, time efficient and reproducible. The need for controlled synthesis routes like reversible deactivated radical polymerisations (RDRP)^3^ for established vinylenes, is hence apparent^2,4^. A method, that allows to prepare polyesters by a robust, radical pathway is the radical ring-opening polymerisation (RROP) of cyclic ketene acetals (CKAs)^5–8^. Underscoring their potential, a number of biodegradable and functional polymers have already been prepared by RROP and deployed in the high-end application of drug-delivery^9–16^. RROP has been widely used to prepare partially or fully enzymatically degradable polymers, rendering them biodegradable by the definition of the International Union of Pure and Applied Chemistry (IUPAC)^17^. Selected RROP homopolymers are even biodegradable following the stricter biodegradability-definition of the Organisation for Economic Co-operation and Development (OECD with standard 301F)^5,14,18,19^. In this context, the polymers from the CKAs 2-methylene-1,3,6-trioxocane (MTC, Fig. 1A) or 2-methylene-1,3-dioxepane (MDO) have been described as biodegradable components in partially degradable block copolymers and also nanoparticles (NPs, Fig. 1A) with tuneable degradability.

When designing the polymers for such RROP-based NPs, a fundamental decision has to be made. If the CKAs are copolymerised with vinylenes, a broad range of defined polymers from controlled radical polymerisations are available, but the polymers only degrade partially into a mixture of oligomers. If only CKAs are deployed, the typically applied free RROP (fRROP) leads to lesser defined polymers with broad dispersities, but they degrade completely into small molecules^10,16,20–22^. Polyesters from a controlled RROP of CKAs have the potential to overcome this dilemma.

In order to apply methods of an RDRP within RROP, the more complex reaction mechanism of RROP of CKAs^14,23^ needs to be discussed briefly. It starts with the addition of a radical to the exocyclic double bond of the CKA, leading to an intermediate tertiary radical. This is followed by the *β*-scission process as the ring-opening step, yielding a primary radical that propagates the polymerisation (Fig. 1B). Following the high reactivity of the primary radical, intra- and intermolecular H-transfers are typical side-reactions and lead to short- and long-chain branching (SCB and LCB), respectively^11,15,16,23^. In this context, branching will be referred to as density of branches (*DB*) in %. A *DB* of 10% means that 10 out of 100 repeating units are involved in either SCB or LCB. Both types of branching are commonly identified by the additional methyl group formed during the reaction (Fig. 1B)^14,23,24^. Trapping the primary radical has therefore the potential to reversibly deactivate the radical polymerisation and hence enable the desired control over this reaction. A number of reports discuss the application of RDRP protocols onto RROP of CKAs: (i) atom transfer radical polymerisation (ATRP), (ii) nitroxide-mediated radical polymerisation (NMRP) or (iii) reverse addition-fragmentation chain transfer (RAFT) polymerisation. In these approaches, successful end-group control was reported and initial but limited steps towards molar mass control could be achieved^11,25–31^. PCKAs with dispersities in the range of 1.2 could be prepared, but insights into the kinetic behaviour, the effects on the branching reaction and the formation of block-copolymers from CKAs remained limited so far. However, the mentioned factors are vital to claim control over a radical polymerisation. Considering that ATRP typically requires for metal-catalysts and NMRP for high reaction temperatures, both of them are not ideal for RROP of CKAs. NMRP has the additional shortcoming that it applies limited control over the polymerisation with a highly reactive propagating species. As RROP propagates via a highly reactive primary radical, NMRP was not considered for this study. RAFT, on the other hand, can be conducted at mild reaction conditions, has a high versatility towards various functional groups and uses no metal catalyst^32,33^. RAFT also provides a wide range of possible chain-transfer-agents (CTAs) to accommodate also highly reactive propagating species^11,16,32,34^. It therefore harmonises well with RROP as a robust RDRP technique to prepare polyesters by solely mixing radical source, CTA and monomer.

Even though reversibly deactivating a primary radical is possible in RAFT, it is still challenging. After addition of the CTA to the primary radical, the dormant radical on the CTA needs to be sufficiently unstable for the primary radical to fragment off. This is contrary to the selection of more reactive CTAs for (meth)acrylates, which form much more stable secondary and tertiary radicals as propagating species. In this respect, cyanomethyl (*N*-methyl-*N*-phenyl)carbamodithioate (CMPCD, Fig. 1A) is a promising commercially available lesser active CTA, displaying excellent chain transfer kinetics towards the propagating species of less-activated monomers, such as vinyl esters and vinylamides^33^. It destabilises the dormant radical through the positive mesomeric effect of nitrogen atom and was hence an ideal candidate for RAFT-RROP^32^. It was also already used for a MTC-ethylen copolymerisation^35^. It should be noted that successfully trapping the radical still means that the propagating species of RROP remains a primary radical. In its active state, this primary radical is still able to stabilise itself via an inter- or intramolecular H-transfer. Branching reaction are hence likely to occur and the *DB* needs to be looked into.

### Overview of the goal of this work and RROP mechanism

This study continues previous efforts by us and others to understand, control and promote the RROP specifically of MTC and MDO^36–39^, by extending it into RAFT as an RDRP. In order to gain a high level of control in RAFT-RROP, several reaction parameters, including initiator concentration, monomer concentration and the amount of CTA are looked into in detail. We conduct a close evaluation on how these various conditions affect molar mass-, end group- and kinetic control of the polymerisation. To prove the versatility of the concept, completely CKA-based P(MTC-*b*-MTC) and P(MTC-*b*-MDO) block copolymers were prepared and formulated into fully biodegradable NPs (Fig. 1A). The work will thus serve as a starting point for well-defined PCKA-homopolymers, CKA-based block copolymers and fully enzymatically degradable polymeric NPs from this chemistry.

## Results and discussion

### Verification of a controlled polymerisation

In order to be viewed a controlled radical polymerisation, the applied RAFT-RROP needs to show successful control over molar mass, dispersity, kinetics and end group. Especially the latter is crucial for the intended formation of block-copolymers. In light of dealing with a primary radical, branching is unlikely to be suppressed. In order to fully investigate the reaction, adjusting the concentration of the chain-transfer agent (CTA = CMPCD), the concentration of MTC and the ratio of CTA to the initiator 1,1′-azobis(cyclohexanecarbonitrile) (ABCN) had to be investigated.

In order it easier to follow these efforts, the sample series were labelled as follows: PMTC-X-Y-Z, where X denotes whether the reaction was performed in a bulk (B, 8 M) or diluted state (D, 4 M with tert-butanol as solvent), Y denotes the CTA:initiator ratio (1:1, 1:2, 1:5, 1:10), and Z denotes the relative amount of CTA with respect to the monomer in mol% (5.0 mol%, 2.5 mol% and 1.0 mol% of CTA). PMTC-B-1:1-5.0 would hence be prepared in bulk, using a CTA:initiator 1:1 ratio and 5.0 mol% of the CTA with respect to the monomer (MTC). In all of the upcoming experiments, the reactions were run at 85 °C and crude samples were analysed to avoid distortion of the kinetic data by work-up.

#### Controlled character of the RAFT-RROP

Linear growth of the number average molar mass (*M*_n_) with conversion is an important aspect of a controlled polymerisation. With PMTC-B-1:1-5.0 as a starting point, this was probed and a linear increase of *M*_n_ with increasing conversion up to a conversion of 80% was observed (Fig. 2A), indicating a successful implementation of this aspect of a controlled polymerisation. A continuously low dispersity of less than 2.0 further validated this aspect. At higher conversion above 80%, the dispersity increased to slightly above 2.0. This was likely due to dominating transfer-reactions and lead to a minor drop in the observed *M*_n_. However, this behaviour was to be expected for RAFT^33^ and the RROP of MTC as well^38^. In comparison to PMTC from the previously published fRROP, the *M*_n_ values for RAFT were only 10% of what was reached in fRROP (3 kg mol^−1^ instead of 30 kg mol^−1^) and the dispersity of 1.5 at 70% conversion was notably lower than the observed 2.5 for fRROP^37,38^. Both aspects are typical for RAFT and also indicate a successful implementation of this technique. Control over the reaction kinetics was further shown by the pseudo-first order kinetics behaviour. An almost linear growth of *M*_n_ over conversion was observed independent of the reaction conditions (Fig. 2B for PMTC-B-1:1-5.0, all others in Figure S1 with Supplementary Notes 1–2 of the SI). This meant that no to little deleterious side reactions took place. To validate the end group control, diffusion ordered nuclear magnetic resonance spectroscopy (DOSY) with inverse Laplace transform (ILT) reconstruction was performed on purified PMTC-B-1:1-10.0 (see Table S9, sample DO in Table 1). The polymer was used in order to ensure that all signals are visible and worked up to improve the signal-to-noise ratio. All aromatic signals in this spectrum can only stem from the aromatic ring of the CTA and were hence ascribed to the CMPCD group, where they match reported resonances (blue square in Fig. 2C)^40^. NMR signals from PMTC backbone dominated the spectrum and could be assigned easily following previously published results (Grey square in Fig. 2C)^38^. Both groups of NMR signals were assigned to a similar diffusion coefficient, proving that the PMTC chains contained the aromatic ring of the CTA (Green square in Fig. 2C). For reference, the ILT-reconstructed DOSY spectrum is given in comparison to a reconstruction by multicomponent fit (see Fig. S7, COSY and HSQC spectra in Figs. S8 and S9, respectively). The found distribution in the DOSY-traced diffusion coefficients in the range of ≈ 1 × 10^−9^ to ≈5 × 10^−10^ m^2^ s^−1^ agrees with the found hydrodynamic size distributions traced by triple-detection SEC (see Fig. S6 a). Signal assignments were confirmed by ^1^H,^1^H and ^1^H,^13^C 2D-NMR correlation experiments, respectively (see Figs. S8 and S9). End-group control as a cornerstone of a controlled polymerisation like RAFT was hence certain. In addition, control over *M*_n_ development and control over reaction kinetics were also proven with this first series of experiments. RAFT of the main monomer of this study (MTC) with this specific CTA (CMPCD) can hence be considered a controlled radical polymerisation, i.e. a cRROP protocol.

**Table 1:** Compilation of the end-point data (close to full conversion, see highlighted entries in the noted Tables S2PMTC-B-1:1-10.0S8 in the SI) of the kinetics run at different CTA:initiator (CMPCD:ABCN) ratios and the block-copolymers

No.^a^	Sample	Theoretical M_n_ (kg mol^−1^)	Experimental M_n_ (kg mol^−1^)	Đ	DB (%)	Kinetic Data
R1	PMTC-B-1:1-5.0	2.6	2.3 ± 0.2	1.8 ± 0.2	16.2 ± 0.4	Table S1
R2	PMTC-B-1:2-2.5	5.2	4.8 ± 0.2	2.2 ± 0.2	17.1 ± 1.5	Table S2
R3	PMTC-B-1:5-1.0	13.0	10.3 ± 3.0	3.5 ± 0.1	19.8 ± 0.4	Table S3
R4	PMTC-B-1:10-0.5	26.0	16.2 ± 2.8	3.8 ± 0.6	20.7 ± 3.4	Table S4
D1	PMTC-D-1:1-5.0	2.6	2.2 ± 0.3	1.5 ± 0.1	14.0 ± 4.9	Table S5
D2	PMTC-D-1:10-0.5	26.0	8.9 ± 4.3	3.5 ± 0.1	22.3 ± 1.4	Table S6
C1	PMTC-B-1:1-2.5	5.2	4.2^b^	2.0^b^	13.9 ± 0.9	Table S7
C2	PMTC-B-1:1-1.0	13.0	9.7^b^	2.7^b^	13.9 ± 0.7	Table S8
DO	PMTC-B-1:1-10.0^c^		5.5	1.5	12.0	(just DOSY)
CE1	Macro-CTA^c^	5.2	7.4	1.3	10.3	---
CE2	PMTC-*b*-PMTC	---	12.4	2.3	13.0	---
CE3	PMTC-*b*-MDO	---	11.0	2.1	13.0 (MTC)/20.0(MDO)	---

Table sees the theoretical molar mass calculated by the CTA:CKA (CMPCD:MTC) ratio and the average values from three separate batches and their standard deviation. Experimental M_n_ and Dispersity values are determined from SEC and *DB* determined by ^1^H-NMR.

^a^Samples “R#” were optimised for the CTA:initiator (CMPCD:ABCN) **r**atio, samples “C#” for the concentration of the **C**TA, samples “D#” for the dilution of the monomer, sample DO was prepared for DOSY measurements and samples “CE#” for the experiments on chain extension.

^b^Following a limited sample size, this data could only be retrieved from one synthesis with no error bars from repetitions.

^c^work-up protocol added.

### Varying reaction conditions

**Fig. 3:**
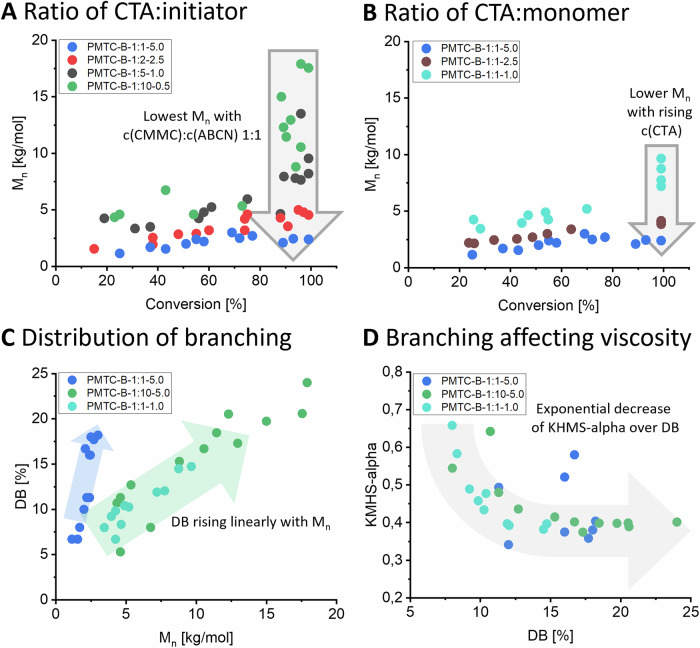
Compilation of the correlation of molar mass with conversion for different reaction conditions. **A** a different ratio of CMPCD:ABCN keeping the initiator concentration constant at 5 mol% (annotation “50” left out), **B** Variation of the CTA and Initiator concentration with a ratio 1:1 CMPCD:ABCN, **C** Distribution of branching with molar mass with varying amount of CMPCD and varying CMPCD:ABCN ratio, **D** Branching affects viscosimetric KMHS-alpha values, which are shown here with the same CMPCD/ABCN variations as in (**C**).

With a proven controlled polymerisation for a first set of reaction conditions, the ability to target different molar mass regimes was studied next. There are two ways to achieve this goal. One would be to keep the amount of the initiator (ABCN) constant, but lowering the concentration of the CTA (CMPCD). This approach will be discussed in this paragraph. Another approach would be to keep the CTA:initiator ratio constant and lower both amounts, which will be looked at afterwards. For the first method, the concentration of the initiator was kept at 5 mol% of the monomer. At the same time, the CTA-concentration was decreased from 5 mol% to 0.5 mol% (PMTC-B-1:1-5.0 to PMTC-1:10-0.5, Fig. 3A, R1-R4 in Table 1 and Tables S1PMTC-B-1:1-10.0S4 with Supplementary Note 3, Figs. S2–S4 (left column)). As expected, decreasing the relative amount of CTA lead to an increased *M*_n_. For 90% conversion, the *M*_n_ increased from 2.3 to 4.8, 10.3 and 16.2 kg mol^−1^ within the sample series PMTC-B-1:1-5.0 to -1:10-0.5, respectively. Dispersity increased from 1.8 to 3.8 within this series and the *DB* rose by comparatively small margin from 16.2 to 20.7%. In this series, the previously mentioned PMTC-B-1:1-5.0 as well as PMTC-B-1:2-2.5, showed a linear trend of the traced *M*_n_ over the conversion. This was not the case for the other two samples, hinting a partial loss of control with decreasing relative amount of CTA^38^. An increasing standard deviation of the final *M*_n_ between different batches of the series further validated this notion (Table 1). Being almost negligible at 0.2 kg mol^−1^ for ratios of 1:1 and 1:2 of CTA:initiator, the standard deviation increased to notable 3.0 and 2.8 kg mol^−1^ for 1:5 and 1:10 ratios, respectively. A comparison to the theoretical molar masses (*M*_n,t_) set by the CTA:monomer ratio followed the same trend as the values for 5 mol% (R1, 20 repeating units) and 2.5 mol% (R2, 50 repeating units) CTA were almost within the narrow margin of error of the experimental values (*M*_n,e_) (*M*_n,e_ = 2.3 ± 0.2 kg mol^−1^ vs *M*_n,t_ = 2.6 kg mol^−1^ and *M*_n,e_ = 4.8 ± 0.2 kg mol^−1^ vs *M*_n,t_ = 5.2 kg mol^−1^, respectively, Table 1). The *M*_n,t_ is therefore in close proximity to the detected values and a satisfying level of *M*_n_ control could be achieved. For 1.0 and 0.5 mol% CTA (R3 and R4, respectively), the theoretical values deviated more notably (*M*_n,e_ = 10.3 ± 3.0 vs. *M*_n,t_ = 13 kg mol^−1^ and *M*_n,e_ = 16.2 ± 2.8 vs. *M*_n,e_ = 26 kg mol^−1^, respectively, Table 1). Reducing the amount of CTA hence continuously increased the gap between the theoretical and *M*_n,e_, further validating the suggested loss of control over the polymerisation with decreasing CTA content. Moving from the polymerisation in bulk to a polymerisation in dilution (i.e. with solvent) with therefore a lower viscosity, did not increase control over the polymerisation and was hence not followed up on further (see Fig. S1, D1 and D2 in Table 1, all data in Tables S5 and S6, plotted in Figs. S2–S4 (middle column)).

#### Optimising the reaction conditions

Following the best kinetic and *M*_n_ control at a CTA:initiator ratio of 1:1, this ratio was kept for the second approach to control the *M*_n_. The amount of the CTA ( = CMPCD) was again reduced from 5.0 mol% to 2.5 mol% and 1.0 mol% relative to the CKA-concentration (PMTC-B-1:1-2.5 and PMTC-B-1:1-1.0, C1-C2 Table 1, all data Tables S7 and S8, plotted in Figs. S2–S4 (right column)). Similar to the values above, a CMPCD amount of 2.5 mol% in sample C1 lead to a *M*_n_ closer to the theoretical value than 1.0 mol% in sample C2 (4.2 and 9.7 kg mol^−1^, with theoretical values of 5.2 and 13.0 kg mol^−1^, respectively). Compared to varying CTA:initiator ratio discussed previously, locking the 1:1 ratio did lead to lower dispersity values (for example to 2.7 from 3.5 for 1.0 mol% CTA) and the *DB* was also reduced to 13.9%. Control over the *M*_n_ by the 1:1 CTA:monomer ratio was hence re-iterated. All data provided a clear and coherent indication that this ratio should be used in a RAFT-RROP.

All of these experiments were synthesised on low lab scale and yielded 50–500 mg of polymer. In order to evaluate scale-up, a PMTC-B-1:1-1.0 batch was started with 50 g of MTC and yielded 37 g of PMTC of 15.5 kg mol^−1^ after workup, showcasing that this polymerisation protocol also works at the upper end of the lab scale, albeit with a substantially increased dispersity (6.5). This ability of upscaling the reaction is of very high importance for a more widespread application of RAFT-RROP, even though significant technical optimisations like uniform heating or adjusting the addition of chemicals are required to achieve polymers with a lower dispersity.

#### Table comprising all polymers of this study

In RROP of CKAs, and hence also of MTC, the most notable additional characteristic is the *DB*, which can be retrieved from ^1^H NMR as noted earlier^14^. In fRROP, this could be somewhat pre-set in classic heating and under UV-promotion following an exponential correlation of the *DB* over conversion^38,41^. As RAFT now allowed for a linear growth of *M*_n_ with conversion, an additional handle to target PMTC with a predetermined *DB* became in range. Plotting the *DB* over *M*_n_ revealed an almost linear correlation at the mentioned ideal CTA:initiator ratio of 1:1 (PMTC-B-1:1-5.0 and PMTC-B-1:1-1.0, Fig. 3C). With less CMPCD in the mixture and hence less control over the polymerisation, this correlation became less clear (PMTC-B-1:10-5.0, Fig. 3C). This trend could be followed for all samples (Fig. S3)^38^. As an important note and in stark contrast to RAFT in poly(vinylenes), branching is not supressed in this case. It is, however, more controlled as the *DB* is growing linearly over the conversion and hence in a predictable manner. Considering that branching occurs from a primary radical at the chain end that undergoes intra- or intermolecular H-transfer (leading to SCB and LCB, respectively), this is within reason. RAFT only puts the primary radicals in a dormant state but does not change their reactivity once present in an active state. With increasing conversion and hence decreasing monomer concentration, the likelihood of H-transfer reactions increases and the *DB* hence grows with ongoing conversion. This is very much relevant as branching as an inherent feature of RROP is, for example, known to impact the encapsulation efficiency and degradation rate of PCKAs^42^. The linear growth of *DB* over conversion in RAFT should be seen as a viable handle to tune the properties of PMTC and PCKAs in general.

A property that depends on the *DB* is viscosity with the Kuhn-Mark-Houwink-Sakurada scaling exponent *α*_*KMHS*_. It is usually determined from a multi-detector SEC and can also be used to differentiate between SCB and LCB (KMHS plot, Fig. S1)^23^. In brief, a *α*_KMHS_ of 0.6–0.8 is typical for rather linear polymer chains and decreases with increased branching. Values between 0.5 and 0.6 have been reported to be typical for short-chain branched polymers. Lower values, i.e. *α*_KMHS_ < 0.5, are typical for polymers with long-chain branching^43–45^. All polymers of this study gave the same exponential decay of *α*_*KMHS*_ over *DB* as previously reported for PMDO^23^ (Figs. 3D and S3). Starting from 0.7 for *DB* = 5%, the values decay to around 0.4 with *DB* increasing to about 20%. In contrast to this general trend, shorter polymer series of PMTC-B-1:1-5.0 resulted in higher *α*_KMHS_ and DBs over 10%, suggesting purely SCB on these polymers. This would mean a successful suppression of intermolecular H-transfer in early stages of the reaction as no LCB takes place. Intramolecular H-transfer would still occur sometimes as the propagating primary radical stabilises itself to result in SCB. However, these values could also be due to the low *M*_n_ of this sample series. For all other series, values below 0.5 were typical for polymers with a DB > 10%. Linear polymers could be ruled out from ^1^H NMR as methyl units from the branching reaction were always present. This suggests a transition of polymers with SCB (intramolecular H-transfer) to LCB (intermolecular H-transfer) over the course of the reaction. With decreasing monomer concentration and increasing amount of polymer during the polymerisation, this can be reasoned with a higher probability of the primary radical reacting with another polymer chain over reacting with another monomer. Similar to the previous report on PMDO^23^, this can only be considered as a first indication, and further research into the differentiation between LCB and SCB is required.

### Chain-extension

#### Degradable NPs from chain-extension

**Fig. 4:**
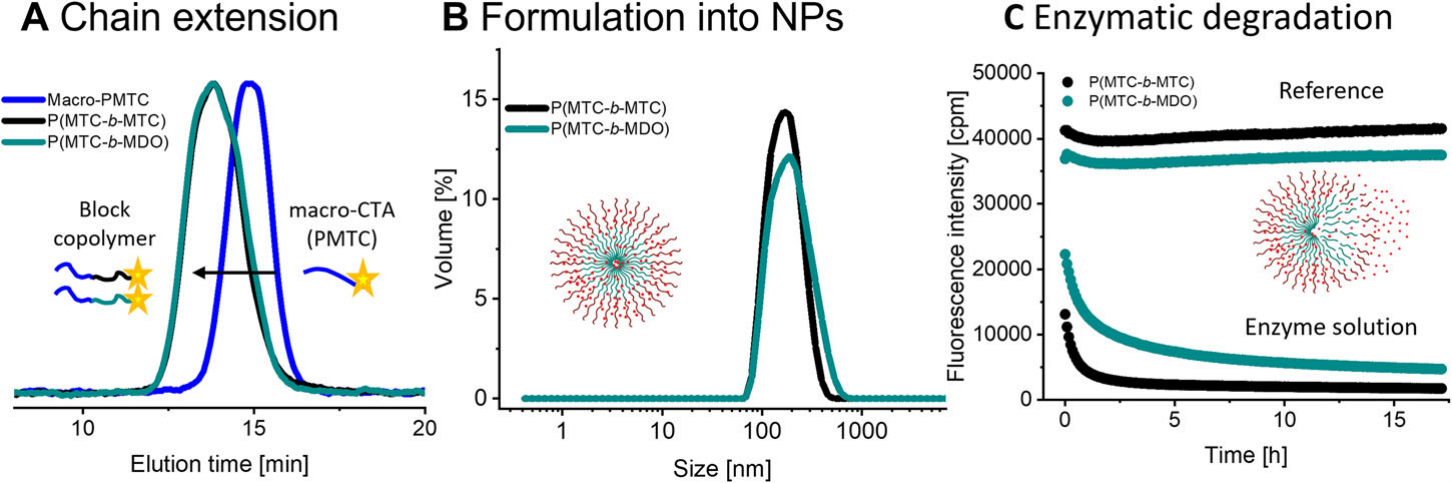
Chain-extended macro-CTAs form nanoparticles for enzymatic degradation. **A** SEC elugram of the polymers from chain-extension of the PMTC macro-CTA to prepare P(MTC-b-MTC) and P(MTC-b-MDO) block-copolymers with RAFT, **B** DLS-traces of the block copolymers formulated by nanoprecipitation, **C** Enzymatic degradation with Lipase (10 U mL^−1^) followed by fluorescence spectroscopy using Nile-Red as a dye and model-drug. The upper line represents the reference and the lower line the solution with enzyme and saline.

Another crucial aspect of any controlled polymerisation that spins from end-group control is the ability to form block-copolymers by chain extension. As end-group control was established for this system, chain extension was the next step. For this, a PMTC-B-1:1-2.5 was run until 80% conversion and worked up to yield a purified macro-CTA (M_n_ = 7.4 kg mol^−1^, *Ð* = 1.3, CE1 in Table 1). The work-up lead to a higher *M*_n_ and lower dispersity than the values reported in the kinetic experiments. Chain extension of the PMTC-macro-CTA was performed with: (i) MTC to keep the same reactivity of all reactants by not changing the CKA monomer and (ii) MDO to prepare the first P(CKA-b-CKA)-block copolymer with a different hydrophobicity and potentially different crystallinity in the blocks. In both cases, SEC clearly showed that the P(MTC)-macro-CTA was chain-extended towards the P(MTC-*b*-MTC) and P(MTC-*b*-MDO) (Fig. 4A). *M*_n_ increased from 7.4 kg mol^−1^ for the macro-CTA to 12.4 kg mol^−1^(CE2 in Table 1, P(MTC-*b*-MTC)) and 11.0 kg mol^−1^ (CE3 in Table 1, P(MTC-*b*-MDO)). The initial dispersity of 1.3 increased as well, but the final block-copolymers still had reasonably low dispersities of 2.1 for P(MTC-*b*-MTC) and 2.3 for P(MTC-*b*-MDO) (Table 1). A second PMTC block of 5 kg mol^−1^ (equal to 40 repeating units) and a PMDO-block of 3.5 kg mol^−1^ (equal to 30 repeating units) was hence added to the macro-CTA. Especially the formation of P(MTC-*b*-MDO) is highly relevant as it shows that the concept of RAFT-RROP is transferable to CKAs other than just MTC (^1^H NMR in Fig. S5). Looking at the SEC traces, the slight tailing towards lower *M*_n_ point towards an incomplete re-initiation (i.e. left-over macro-CTA) as the most likely reason for the increase in dispersity and could be expected for any RAFT process^34^. An extensive 2D NMR analysis (DOSY, COSY and HMBC) of P(MTC-*b*-MTC) (Figs. S10–S13) and P(MTC-*b*-MDO) (Figs. S14–S17) did confirm the presence of the RAFT group on both block-copolymers, solidifying the observation of a successful chain-extension. Also, for these two materials, the DOSY-traced distribution of diffusion coefficients is aligning with the hydrodynamic size distribution observed by triple-detection SEC (see Fig. S6b, c). Note, that this served as a proof-of-concept and further optimisation of the chain-extension will likely allow for lower dispersity of the block copolymers in the future.

Successful use of active end-groups for chain extension was hence proven. RROP of CKAs as monomers becomes a controlled RAFT-RROP with the CTA discussed in here ( = CMPCD). It is a controlled radical ring-opening polymerisation (cRROP). The inclusion of MDO showed that the concept can be applied to other CKAs as well and the synthesis of a fully CKA-based block-copolymer readied the materials for an application as biodegradable NPs.

### Nanoparticle formation and degradation

From the many pathways to reach NPs, nanoprecipitation was shown to be effective for P(CKAs) from fRROP^42,46^. It was hence performed in here using the block copolymers just discussed. In brief, the polymer samples were dissolved in THF as a volatile solvent, dropped into deionised water and stirred overnight to yield well-defined NPs. The NPs were analysed for their size by dynamic light scattering (DLS, Fig. S19 with Supplementary Note 5) and (cryogenic) transmission electron microscopy (TEM and cryo-TEM, Fig. S20 with Supplementary Note 6). A similar DLS-trace was observed for NPs from both block-copolymers. Comparable sizes of 170 nm and 190 nm with a low polydispersity index (PDI) of 0.09 and 0.12 were reached for NPs from P(MTC-*b*-MTC) and P(MTC-*b*-MDO), respectively. TEM and cryo-TEM confirmed the uniform NPs from the block-copolymers. The contrast between the ice-matrix and the polymer particles in cryo-TEM was low, reducing the overall reproducibility of these measurements (Fig. S20). A control for NPs of PMTC from fRROP showed a reasonable DLS trace as well, but resulted in disassembled NPs in TEM (Fig. S20). Such unstable NPs from fRROP are in agreement with earlier work^42^. In other words, RAFT did not only allow for a cRROP, but also produced block-copolymers, which enabled an expanded range of stable and defined NPs fully based on PCKAs.

With the NPs in-hand, this allowed for applying our existing platform for studying drug encapsulation, drug release and biodegradation by IUPAC-standards in one go^16,22^. In this assay, the NPs based on PCKAs encapsulate Nile-Red as a fluorophore and are then treated with Lipase (10 U mL^−1^) to hydrolyse the ester bonds. In order for the enzyme to be effective, the solution had to be adjusted towards 20 mM NaCl, but the NPs from RAFT remained stable under these conditions (DLS in Fig. S19). As Nile Red becomes non-fluorescent in a hydrophilic environment, release of the dye from the NPs during the enzymatic degradation of the PCKAs can be followed using fluorescence spectroscopy. Time-course fluorescence measurements showed complete release of Nile Red and hence full hydrolysis of the block-copolymers forming the NPs (Fig. 4C). To back-up these findings, the degradation products were analysed by SEC-MALS and as expected, the polymer peak disappeared completely. Full enzymatic degradation of the block-copolymers was hence confirmed (Fig. S21 with Supplementary Note 7). It was also notable that NPs from P(MTC-*b*-MDO) degraded slower than the NPs from P(MTC-*b*-MTC), albeit not by much (Fig. 4C). This was expected since PMDO is a semi-crystalline polymer, and those are known to slow-down enzymatic degradation^23,47,48^. Differential Scanning Calorimetry (DSC) was hence conducted to check for semi-crystalline domains. Although not strong, a small melting peak in the range of 15–25 °C could be identified in the DSC trace of P(MTC-*b*-MDO) and was consistent with previously published data^23^. Since no crystallisation peak could be detected, this should be seen as an additional note of a very small degree of crystallisation. However, this small melting peak was not in the DSC trace of P(MTC-*b*-MTC) (Fig. S18). In very good consistency, the differences in degradation rate of the NPs and the melting peak of P(MTC-*b*-MDO) are both small, but notable.

Following the complete biodegradability, RAFT-RROP of CKAs hence introduces various levels of controls from synthesis all the way to degradation and is hence a very suitable RDRP protocol.

## Conclusion

This work introduced RAFT-RROP as a controlled radical polymerisation of CKAs with CMPCD as a CTA, i.e. RAFT agent. Control over the polymerisation was shown on various levels. With a 1:1 ratio of CTA:initiator (CMPCD:ABCN), the *M*_n_ of the sample polymer PMTC could be pre-set up to 15 kg mol^−1^ by the amount of CTA in the system. Control could be attained up to 80% conversion. Using the same CTA:initiator ratio, the dispersity of the polymers was low throughout the polymerisation, only surpassing 1.5 when conversions > 80% were reached. Up until that point the *M*_n_ of the polymers grew linearly over the conversion, verifying a controlled radical polymerisation. With this linear correlation, the previously observed growth of the *DB* over conversion could now be translated into a linear growth of the *DB* with *M*_n_ in a defined set of reactions conditions. More control over the *DB* was hence reached as well. Since RAFT enabled end-group control, the polymers could be chain-extended with both, MTC and MDO leading to well-defined fully CKA-based block copolymers. These are a major accomplishment themselves, but also showed that cRROP with this RAFT agent is a more broadly applicable concept as it can be transferred to other CKAs like MDO. NPs from these block-copolymers were fully degradable and the semi-crystallinity of an individual block like PMDO provided an initial small handle to control the degradation speed of the NPs and – by that – the release rate of the previously included dye. As the dye was fully released once the polymers were degraded, this already shows possible fields of application, may it be as sensors (decreasing fluorescence) or drug delivery in biomedical applications. All of this goes to show that RAFT with CMPCD as a CTA is powerful way to introduce all characteristics of a controlled radical polymerisation to RROP of CKAs.

## Methods

All Chemicals were purchased from Sigma-Aldrich, Merck Millipore, Fisher Scientific and Acros Organics. Diethylene glycol, chloroacetaldehyde dimethanoloacetal, potassium *tert*-butoxide, Lipase from Pseudomonas cepacia and 1,1’-azobis(cyclohexanecarbonitrile) were purchased from Sigma Aldrich. Cyanomethyl (*N*-methyl-*N*-phenyl)carbamodithioate was obtained as a gift from Boron Molecular. *tert*-Butanol was purchased from Thermo Fisher Scientific. All chemicals were used without further purification.

### Synthetic procedures

#### 2-Chloromethyl-1,3,6-trioxocane (MTC-Cl) and 2-methylene-1,3,6-trioxocane (MTC)

The synthesis of MTC and MTC-Cl was performed as reported in literature and is described in detail in section 1 of the SI^47^. In brief, diethylene glycol, chloroacetaldehyde dimethanoloacetal and DOWEX WX2 was mixed at 120 °C, the product was distilled to obtain the MTC-Cl. The chloroacetal was then mixed with *tert*-butanol and potassium *tert*-butoxide and heated to 170 °C for 30 min in a microwave reactor. The product was extracted with diethyl ether and distilled multiple times to gain MTC as a clear, transparent liquid.

### Polymerisation of MTC

All polymerisation reactions were performed in a closed round bottom flask heated by an oil bath at 85 °C. The reaction mixture consisting of MTC, *tert*-butanol (for the diluted samples), 1,1’-azobis(cyclohexanecarbonitrile) (ABCN) and CMPCD ( = the CTA) were degassed for 15 min prior to polymerisation. After the respective time points, a sample of 0.1 mL was taken out of the reaction solution and quenched by exposure to air and cooling the mixture. The samples were then analysed for their molar mass and branching behaviour by SEC-MALS with a viscosity detection and for their conversion and degree of branches by ^1^H NMR spectroscopy.

### Chain-extension

To perform chain-extension, a macro-RAFT agent was prepared by repeating the PMTC-B-1:1-2.5 protocol. For this, 1 g of MTC (7.7 mmol) were mixed with 47 mg ABCN (0.19 mmol) and 43 mg CMPCD (0.19 mmol) (*M*_n_ = 7.5 kg mol^−1^, *Ð* = 1.3). It was run for 22 h until about 85% conversion, precipitated 2x in diethyl ether and dried in a vacuum oven to yield 0.50 g of the macro-CTA. It was then mixed with 280 mg of MDO or 300 mg of MTC and 6.4 mg of ABCN. After the PMTC-macro-initiator has been well-dissolved in the CKA-solution, the samples were stirred for 24 h at 85 °C and after drying in a drying-oven, directly used for the formulation-experiments. These experiments have been conducted in duplicates to have enough sample for the following experiments. As the focus of the paper is between the difference between the blocks, it is not specifically noted, which batch has been used.

### Size exclusion chromatography

To analyse molar mass of the polymer, a similar protocol to previous studies was used^23,38^. SEC coupled with a triple detection setup was used comprised of a degasser, an isocratic pump (both series Agilent 1200), an autosampler (Agilent series 1100) and 2 × SEC columns PLgel MIXED-C columns (300–7.5 mm; 5 μm particle size) by Agilent Technologies Inc. (USA). The downstream detection system was comprised of a multi-angle light scattering (MALS) DAWN® HELEOS® II, a viscometric detector Viscostar® III and a differential refractometer Optilab® T-rEX, all by Wyatt Technology Corp. (USA). Separations were performed in THF (stabilised with 0.025% BHT) as eluent with a flow rate of 1 mL min^−1^. For that, 53 μL of polymer solution were injected having an analyte concentration of 4 mg mL^−1^. Data recording and analysis was done using the Software Astra® by Wyatt Technology Corp. (USA), version 7.3.2. Initial MALS detector normalisation, delay volume alignment and band broadening correction were adjusted by measuring a narrowly distributed PS standard with a *M*_w_ of 30,000 g mol^−1^ (PS80317, Pressure Chemicals Co, USA) and of *Đ* ≤ 1.06 and validated by measurements of other PS standards with higher *M*_w_. All RI elugrams were normalised to the largest peak being set to 1.0. Hence, no y-axis is shown in all cases.

### NMR spectroscopy

^1^H-NMR measurements were performed using a Bruker Advance III 500 spectrometer operating at 500.134 MHz for ^1^H and at 125.74 MHz for ^13^C. Deuterated chloroform (CDCl_3_) was used as solvent and reference (δ(^1^H) = 7.26 ppm; δ(^13^C) = 77.0 ppm). The measurements were carried out at 30 °C using standard pulse sequences of the TopSpin 3.6.4 software package (Bruker Biospin). Processing was done using MestreNova V6.0. 2D ^1^H DOSY experiments were performed using the pulse sequence dstebpgp3s with a gradient strength of 5.35 G mm^−1^, a diffusion delay of Δ = 200 ms, a gradient pulse length of δ = 2 ms, and 32 gradient steps with linear spacing. DOSY data processing was done with the Bruker Dynamics software, version 2.8.8 using both, multicomponent (8 function components) fit and ILT. ^1^H,^1^H COSY, ^1^H,^13^C HSQC and HMBC were measured using the pulse sequences cosygpqf or cosygpppqf, hsqcedetgpsisp2.3, and hmbcgplpndqf from the Bruker pulse sequence library, respectively.

### Formulation of PCKA-based NPs

In order to formulate the prepared block-copolymers, nanoprecipitation was applied as an established formulation strategy. As reported previously^42^, 15 mg of the polymers were dissolved in 1 mL THF and added dropwise to 15 mL of deionised water under constant stirring. After stirring overnight, the 1.0 mg mL^−1^ solution was measured by DLS to determine the size and distribution of the NPs. To test the degradability of the NPs, they were mixed with 125 µL of 0.1 M NaCl solution (20 mL final concentration) and lipase to obtain a concentration of 10 U mL^−1^.

### Dynamic light scattering (DLS) and zeta potential measurements

Dynamic light scattering was used to determine NPs size using a Zetasizer Nano spectrometer (Malvern Instruments Ltd.) equipped with a 633 nm laser at a fixed angle of 173° and a Wyatt DynaPro DLS Plate Reader (Wyatt Technology Corporation, USA). Samples were equilibrated for 30 s at 25 °C before measurement. A Zetasizer Nano spectrometer was also used to measure the zeta potential of the NPs. All samples were measured in triplicate. NPs were analysed at a concentration of 1.0 mg mL^−1^. For the degradation tests described below, a concentration of 0.5 mg mL^−1^ was used.

### Fluorescence spectroscopy and enzymatic degradation

To follow the degradation of the NPs, a previously established protocol was adapted^16^. In brief, Nile red as a fluorophore was encapsulated into the polymeric NPs by a co-nanoprecipitation by mixing the polymer and the dye before the formulation. The respective polymer sample (15 mg) was dissolved in 1.41 mL THF and mixed with 0.36 mL of a Nile-red solution (0.05 mg mL^−1^ in THF) and slowly dropped into deionised water. Following the hydrophobic environment of the polymer, compared to water, an enhanced fluorescence is observed, as long as the NPs remain stable, or the dye remains encapsulated in the NP. The NP-solution was then either mixed in a ratio of 1:4 (NP solution:test solution) with (i) 0.1 M saline solution as a control, or (ii) 0.1 M saline solution with lipase of a concentration of 50 U mL^−1^. Each of these test- and enzyme-solutions were prepared individually for three times and from each mixture, three samples were placed in the microplate reader to allow for a statistical certainty of the measurements (triplets of triplets). Using an Infinite® M Plex, multimode microplate reader, monochromator optics, the samples were heated to 37 °C and analysed for their fluorescence in an interval of 10 min with an excitation wavelength of 550 nm, an emission wavelength of 630 nm and a bandwidth of 3 nm. Following the enzymatic degradation and therefore the release of Nile-red into an aquatic environment, the fluorescence intensity decreased representing the degradation and disassembly of the NPs. Control samples did not see the addition of lipase.

Methodologies of DSC (Supplementary Note 4), DLS (Supplementary Note 5)^22^ and (cryo-)TEM (Supplementary Note 6)^49^ were adapted from earlier work (see citations) and are noted in the SI.

## Supplementary information

Supporting Information

Description of Additional Supplementary Files

Supplementary Data

## Data Availability

All raw data is available at Zenodo: 10.5281/zenodo.17160419 Annotation for the NMR files in the supplementary data with the time points as noted in the SI: DR1: PMTC-B-1:5-1.0, spectra 11-34; DR2: PMTC-B-1:2-2.5, spectra 11-34, DR3: PMTC-B-1:1-5.0, spectra 11-34; DR4: PMTC-B-1:10-0.5, spectra 1-34; DR5: PMTC-D-1:1-5.0, spectra 1-33; DR6: PMTC-D-1:10-0.5, spectra 1-33; DR7: PMTC-B-1:1-1.0, spectra 1-34; DR8: PMTC-B-1:1-2.5, spectra 1-34.

## References

[1] Koltzenburg, S., Maskos, M. & Nuyken, O. *Polymere: Synthese, Eigenschaften und Anwendungen.* 2nd edn (Springer Spektrum Berlin, 2014).

[2] Braungart, M. & McDonough, W. *Cradle to Cradle* (Farrar, Straus and Giroux, 2009).

[3] reversible-deactivation radical polymerization. 5.0.0 ed.; International Union of Pure and Applied Chemistry (IUPAC): 2025.

[4] Mangold, H. &von Vacano, B. The frontier of plastics recycling: rethinking waste as a resource for high-value applications. *Macromol. Chem. Phys*. **223**, 2100488 (2022).

[5] Agarwal, S. Chemistry, chances and limitations of the radical ring-opening polymerization of cyclic ketene acetals for the synthesis of degradable polyesters. *Polym. Chem.* **1**, 953–964 (2010).

[6] Bailey, W. J., Ni, Z. & Wu, S. R. Synthesis of poly-epsilon-caprolactone via a free-radical mechanism - free-radical ring-opening polymerization of 2-methylene-1,3-dioxepane. *J. Polym. Sci. Part A: Polym. Chem.* **20**, 3021–3030 (1982).

[7] Bailey, W. J., Ni, Z. & Wu, S. R. Free-radical ring-opening polymerization of 4,7-dimethyl-2-methylene-1,3-dioxepane and “5,6-benzo-2-methylene-1,3-dioxepane. *Macromolecules* **15**, 711–714 (1982).

[8] Bailey, W. J. 22 - Ring-opening polymerization. *Compr. Polym. Sci. Suppl.* **33**, 283–320 (1989).

[9] Tardy, A. et al. A comprehensive kinetic study of the conventional free-radical polymerization of seven-membered cyclic ketene acetals. *Polym. Chem.* **8**, 5139–5147 (2017).

[10] Guégain, E., Zhu, C., Giovanardi, E. & Nicolas, J. Radical ring-opening copolymerization-induced self-assembly (rROPISA). *Macromolecules* **52**, 3612–3624 (2019).

[11] Pesenti, T. et al. Increasing the hydrophilicity of cyclic ketene acetals improves the hydrolytic degradation of vinyl copolymers and the interaction of glycopolymer nanoparticles with lectins. *Biomacromolecules* **24**, 991–1002 (2023). 36724405 10.1021/acs.biomac.2c01419

[12] Nicolas, J., Mura, S., Brambilla, D., Mackiewicz, N. & Couvreur, P. Design, functionalization strategies and biomedical applications of targeted biodegradable/biocompatible polymer-based nanocarriers for drug delivery. *Chem. Soc. Rev.* **42**, 1147–1235 (2013). 23238558 10.1039/c2cs35265f

[13] Pesenti, T. & Nicolas, J. 100th anniversary of macromolecular science viewpoint: degradable polymers from radical ring-opening polymerization: latest advances, new directions, and ongoing challenges. *ACS Macro Lett.* **9**, 1812–1835 (2020). 35653672 10.1021/acsmacrolett.0c00676

[14] Deng, Y., Mehner, F. & Gaitzsch, J. Current standing on radical ring-opening polymerizations of cyclic ketene acetals as homopolymers and copolymers with one another. *Macromol. Rapid Commun.* **44**, 2200941 (2023). 10.1002/marc.202200941 36881376

[15] Deng, Y., Frezel, A., Mehner, F., Friedel, P. & Gaitzsch, J. Amine-bearing cyclic ketene acetals for pH-responsive and degradable polyesters through radical ring-opening polymerisation. *Polym. Chem.* **14**, 4275–4281 (2023).

[16] Deng, Y. et al. Amphiphilic block copolymers PEG-PMTCs: synthesis, self-assembly, degradation properties and biocompatibility. *Biomacromolecules* **25**, 303–314 (2024). 38039186 10.1021/acs.biomac.3c00992

[17] Vert, M. et al. Terminology for biorelated polymers and applications (IUPAC Recommendations 2012). *Pure Appl. Chem.* **84**, 377–410 (2012).

[18] Horie, K. et al. Definitions of terms relating to reactions of polymers and to functional polymeric materials (IUPAC Recommendations 2003). *Pure Appl. Chem.* **76**, 898 (2004).

[19] Du, Y. et al. Comparative study of N-Vinyl pyrrolidone and cyclic ketene acetal copolymer degradation under alkaline, enzymatic, or wastewater conditions. *Polymer* **309**, 127444 (2024).

[20] Zhu, C. & Nicolas, J. Towards nanoparticles with site-specific degradability by ring-opening copolymerization induced self-assembly in organic medium. *Polym. Chem.* **12**, 594–607 (2021).

[21] Lages, M. et al. Degradable polyisoprene by radical ring-opening polymerization and application to polymer prodrug nanoparticles. *Chem. Sci.* **14**, 3311–3325 (2023). 36970097 10.1039/d2sc05316k PMC10034157

[22] Castro, N. L. S. et al. Fine-tuning the crystallinity and enzymatic degradation of nanoparticles produced by radical ring-opening polymerization (RROP). *J. Polymer Sci.* **63**, 3777–3785 (2025).

[23] Mehner, F., Geisler, M., Arnhold, K., Komber, H. & Gaitzsch, J. Structure-property relationships in polyesters from UV-initiated radical ring-opening polymerization of 2-Methylene-1,3-dioxepane (MDO). *Acs Appl. Polymer Mater.* **4**, 7891–7902 (2022).

[24] Tardy, A., Nicolas, J., Gigmes, D., Lefay, C. & Guillaneuf, Y. Radical ring-opening polymerization: scope, limitations, and application to (Bio)degradable materials. *Chem. Rev.* **117**, 1319–1406 (2017). 28085265 10.1021/acs.chemrev.6b00319

[25] Yuan, J.-Y., Pan, C.-Y. & Tang, B. Z. “Living” free radical ring-opening polymerization of 5,6-Benzo-2-methylene-1,3-dioxepane using the atom transfer radical polymerization method. *Macromolecules* **34**, 211–214 (2001).

[26] Tao He, Y.-F. Z. & Pan, C. ai-Y. uan Controlled/“living” radical ring-opening polymerization of 5,6-Benzo-2-Methylene-1,3-Dioxepane based on reversible addition-fragmentation chain transfer mechanism. *Polym. J.* **34**, 138–143 (2002).

[27] Wickel, H., Agarwal, S. & Greiner, A. Homopolymers and random copolymers of 5,6-Benzo-2-methylene-1,3-dioxepane and methyl methacrylate: structural characterization using 1D and 2D NMR. *Macromolecules* **36**, 2397–2403 (2003).

[28] Tardy, A. et al. Scope and limitations of the nitroxide-mediated radical ring-opening polymerization of cyclic ketene acetals. *Polym. Chem.* **4**, 4776–4787 (2013).

[29] Yen Wei, E. J. C., Jia, X. & Wang B. First example of free radical ring-opening polymerization with some characteristics of a living polymerization. *Chem. Mater*. **8**, 604–606 (1996).

[30] Wei, Y. en, Xinru Jia, E. J. C. & Wang, C. E. Controlled free radical ring-opening polymerization and chain extension of the “living” polymer. *J. Polymer Sci. Part A: Polymer Chem.* **36**, 761–771 (1998).

[31] Bell, C. A., Hedir, G. G., O’Reilly, R. K. & Dove, A. P. Controlling the synthesis of degradable vinyl polymers by xanthate-mediated polymerization. *Polym. Chem.* **6**, 7447–7454 (2015).

[32] Keddie, D. J., Moad, G., Rizzardo, E. & Thang, S. H. RAFT agent design and synthesis. *Macromolecules* **45**, 5321–5342 (2012).

[33] Keddie, D. J. A guide to the synthesis of block copolymers using reversible-addition fragmentation chain transfer (RAFT) polymerization. *Chem. Soc. Rev.* **43**, 496–505 (2014). 24129793 10.1039/c3cs60290g

[34] Hartlieb, M. Photo-iniferter RAFT polymerization. *Macromol. Rapid Commun.* **43**, 2100514 (2022). 10.1002/marc.202100514 34750911

[35] Zeng, T.-Y., Xia, L., Zhang, Z., Hong, C.-Y. & You, Y.-Z. Dithiocarbamate-mediated controlled copolymerization of ethylene with cyclic ketene acetals towards polyethylene-based degradable copolymers. *Polym. Chem.* **12**, 165–171 (2021).

[36] Kwon, S., Lee, K., Bae, W. & Kim, H. Precipitation polymerization of 2-Methylene-1,3-dioxepane in supercritical carbon dioxide. *Polym. J.* **40**, 332–338 (2008).

[37] Mehner, F. et al. Supercritical RROP: exploring the radical ring-opening polymerisation of 2-methylene-1,3,6-trioxocane in supercritical CO2 as a green solvent. *Polymer* **309**, 127373 (2024).

[38] Mehner, F., Meissner, T., Seifert, A., Lederer, A. & Gaitzsch, J. Kinetic studies on the radical ring-opening polymerization of 2-methylene-1,3,6-trioxocane. *J. Polymer Sci.* **61**, 1882–1892 (2023).

[39] Zeng, T. et al. Degradable PE-based copolymer with controlled ester structure incorporation by cobalt-mediated radical copolymerization under mild condition. *iScience* **23**, 100904 (2020). 10.1016/j.isci.2020.100904 PMC7044514 32106055

[40] Abreu, C. M. R. et al. Reversible addition–fragmentation chain transfer polymerization of vinyl chloride. *Macromolecules* **45**, 2200–2208 (2012).

[41] Meissner, T., Friedel, P., Carroll, J. A., Barner-Kowollik, C. & Gaitzsch, J. Photochemical action plots evidence UV-promoted radical ring-opening polymerisation of cyclic ketene acetals. *Polym. Chem.* **16**, 704–711 (2025).

[42] Axioti, E. et al. Nanoprecipitation and drug delivery with PMTC: toward biomedical application of polyesters from radical ring-opening polymerization. *Macromol. Biosci*. **26**, e00432 (2025). 10.1002/mabi.202500432 PMC12829526 41111163

[43] Lederer, A. & Burchard, W. Solution viscosity. in *Hyperbranched Polymers: Macromolecules in between Deterministic Linear Chains and Dendrimer Structures*. 88–135 (Royal Society of Chemistry: 2015).

[44] Brandt, J. et al. Investigation of thermoreversible polymer networks by temperature dependent size exclusion chromatography. *Polym. Chem.* **8**, 6598–6605 (2017).

[45] Geisler, M. et al. Topology analysis of chain walking polymerized polyethylene: an alternative approach for the branching characterization by thermal FFF. *Macromolecules* **52**, 8662–8671 (2019).

[46] Deng, Y., Debbeler, J., Traeger, A. & Gaitzsch, J. Degradable nanocarriers capable of gene delivery derived from pH-responsive polyester: RROP copolymerization between cyclic ketene acetals. *Macromol. Rapid Commun.* e00722 (2025). 10.1002/marc.202500722 PMC13384792 41100726

[47] Folini, J., Murad, W., Mehner, F., Meier, W. & Gaitzsch, J. Updating radical ring-opening polymerisation of cyclic ketene acetals from synthesis to degradation. *Eur. Polym. J.* **134**, 109851 (2020).

[48] Hillmyer, M. A., Petersen, M. A., Yin, L. G. & Kokkoli, E. Synthesis and characterization of reactive PEO-PMCL polymersomes. *Polym. Chem.* **1**, 1281–1290 (2010).

[49] Bunk, C. et al. Amphiphilic tetra-PCL-b-PEG star block copolymers using benzoxazinone-based linking groups. *Polym. Chem.* **14**, 1965–1977 (2023).

