# Supplementary material for: RAFT enables controlled radical ring-opening polymerisation of cyclic ketene acetals for degradable nanoparticles
Source: Commun Chem. 2026 Apr 9;9:156. doi: 10.1038/s42004-026-01997-6 (PMC13069079; doi:10.1038/s42004-026-01997-6)
Supplement: Supplementary file 1 — Supporting Information [file 42004_2026_1997_MOESM1_ESM.pdf]

Supporting Information

RAFT enables controlled radical ring-opening polymerisation of cyclic ketene acetals for degradable nanoparticles

Fabian Mehner[a,b], Aniket Bukane [b], Daniel Keddie[c], Martin Geisler [a],

Albena Lederer [a,d], Brigitte Voit [a,b] and Jens Gaitzsch [a,*]

[a] Leibniz-Institut für Polymerforschung Dresden e.V., Hohe Straße 6, 01069 Dresden, Germany

[b] Organic Chemistry of Polymers, TUD Dresden University of Technology, Dresden, 01062, Germany

[c] School of Chemistry, University of Nottingham, University Park, Nottingham, NG7 2RD, United Kingdom

[d] Department Chemistry and Polymer Science, Stellenbosch University, Matieland, South Africa

* Corresponding author: gaitzsch@ipfdd.de (J.G.)

Contents of the supplementary data and methods:

1. Polymer analysis: branching, molar mass and KMHS-values 2
2. Screening of reaction conditions of the RAFT agents 3
3. Chain-extension and block copolymers 10
4. Formulation and self-assembly 18
5. Degradation by Lipase from Pseudomonas cepacia 21
6. References 23

All raw data is available at Zenodo: DOI: 10.5281/zenodo.17160419

Keywords: RROP, MTC, RAFT, Polyester, block copolymers, nanoparticles

1. Polymer analysis: branching, molar mass and KMHS-values

Supplementary note 1: The analysis of the polymer samples studied in this work was performed using size exclusion chromatography (SEC) and nuclear magnetic resonance spectroscopy (NMR). As discussed in previous studies, the molar mass was determined by a combination of a refractive index detection to determine concentration and a light scattering detection to determine the absolute molar mass of the polymers. The Kuhn-Mark-Houwink-Sakurada plot (KMHS) was used to quantify the long chain branches and the NMR spectroscopy allowed to determine conversion and the degree of branches (DB). For details, please refer to one of the previous publications on PMTC and the branching-analysis of PMDO.[1-4]

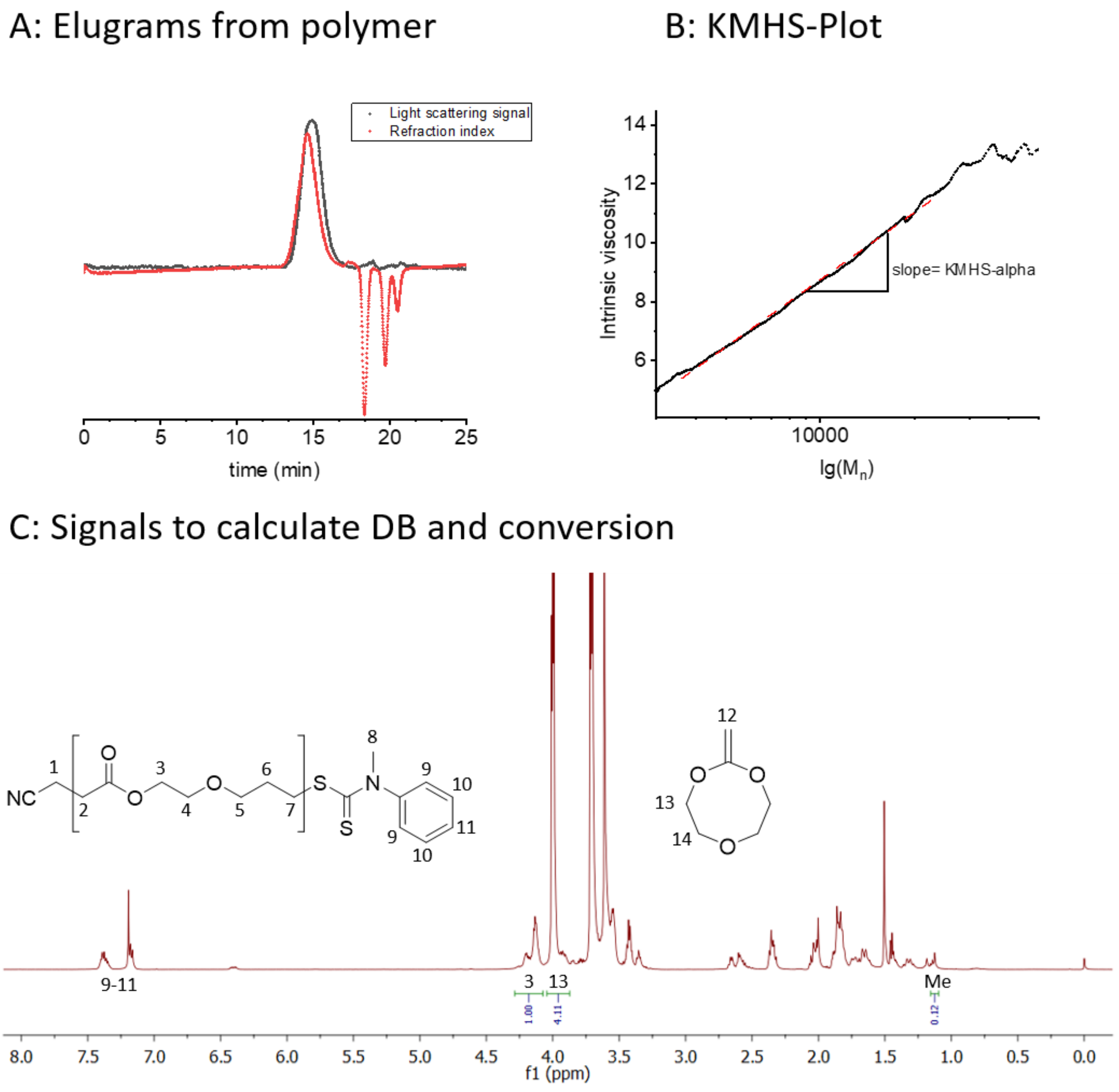

Figure S 1: A) SEC elugrams from a selected PMTC-sample (PMTC-B-1:1-5.0), B) Respective KMHS-plot as a plot of the intrinsic viscosity with varying molar mass used to determine the KMHS-alpha. C) Chemical structure of the polymer and monomer and ^{1}H NMR spectrum containing both with assignment of major signals.

Supplementary note 2: As depicted in Figure S1, using RAFT well-defined, monomodal SEC-traces were observed; the KMHS-values were calculated from the slope of the KMHS-plot and the DB and conversion from ^{1}H NMR spectra, following the established equations:

$$Conversion = \frac{I(3)}{I(3) + \frac{1}{2}\ I(13)}$$

$$DB = \frac{2}{3} \cdot \frac{I(Me)}{I(3)}$$

2. Screening of reaction conditions of the RAFT agents

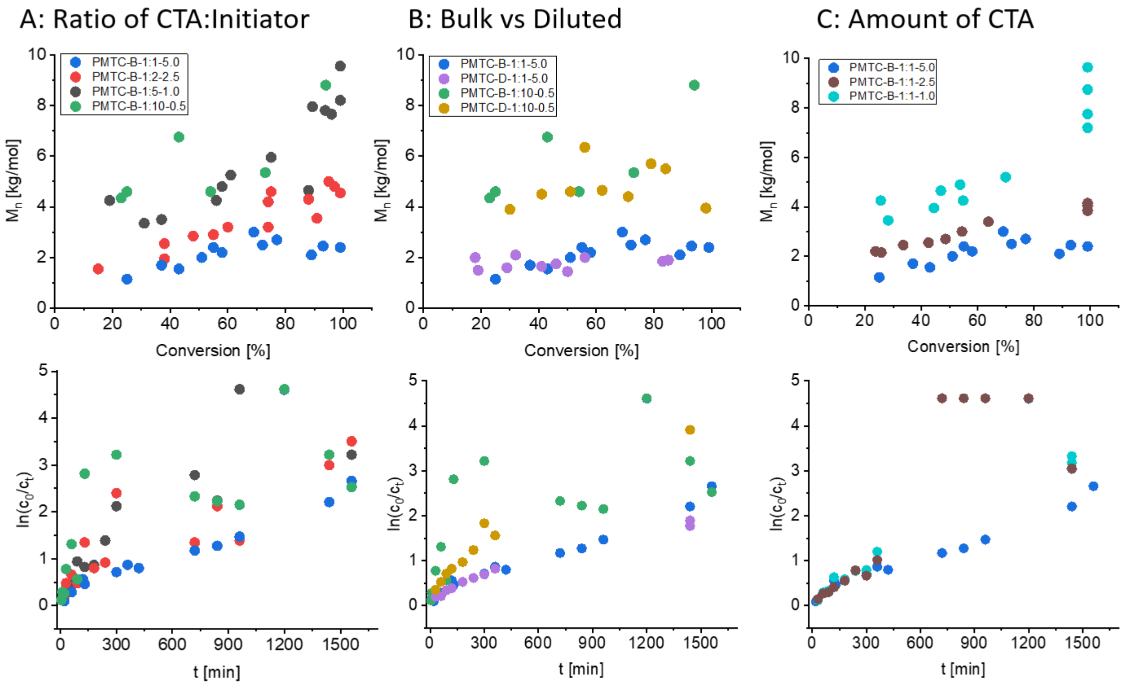

Figure S 2 Proof of the controlled character of RROP with RAFT for the pseudo-first order kinetics and the correlation of molar mass with conversion for: A) A varying ratio of CTA:Initiator, B) a comparison between RROP in bulk and diluted state and C) a varying ratio of CKA:CTA with a fixed ratio of CTA:Initiator of 1:1.

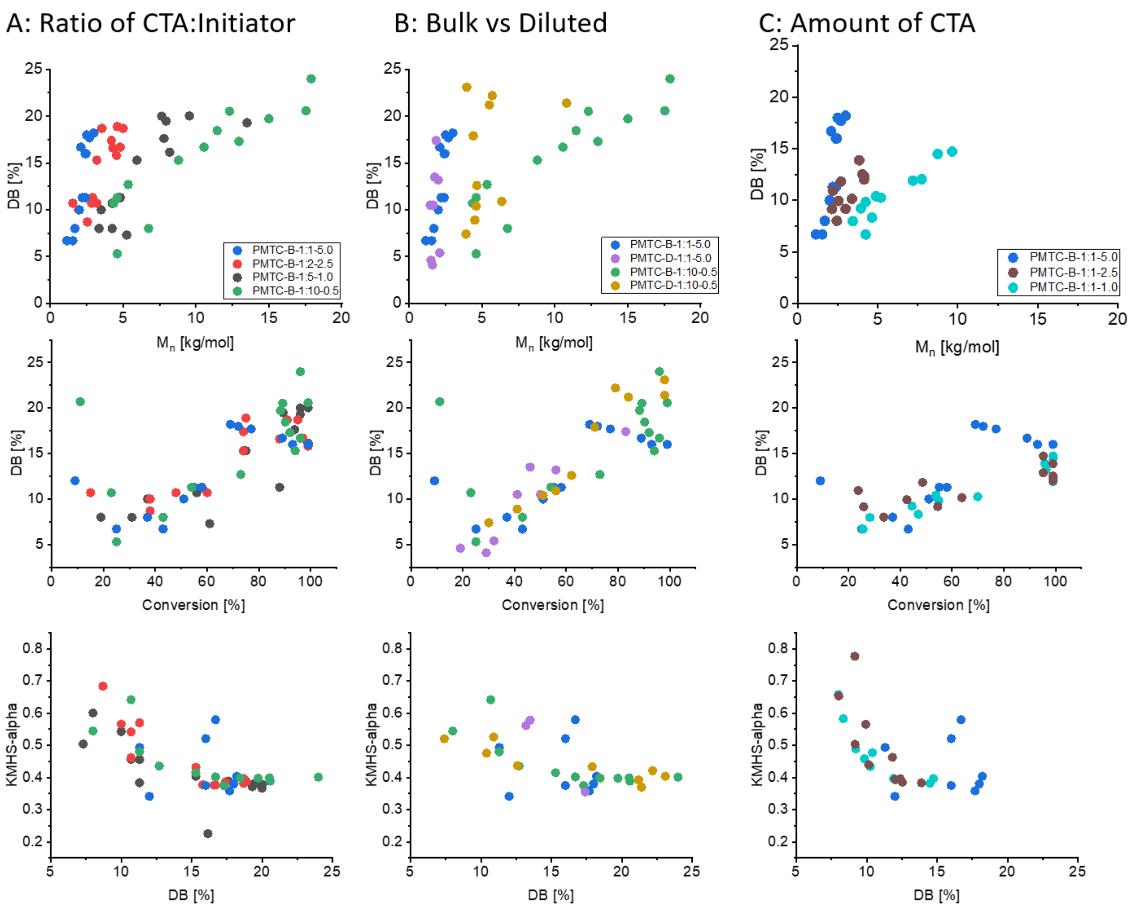

Figure S 3: Correlation of the DB with molar mass, correlation of DB with conversion and the KMHS-parameters as a measure of LCB with the DB for: A) A varying ratio of CTA:Initiator, B) a comparison between RROP in bulk and diluted state and C) a varying ratio of CKA:CTA with a fixed ratio of CTA:Initiator of 1:1.

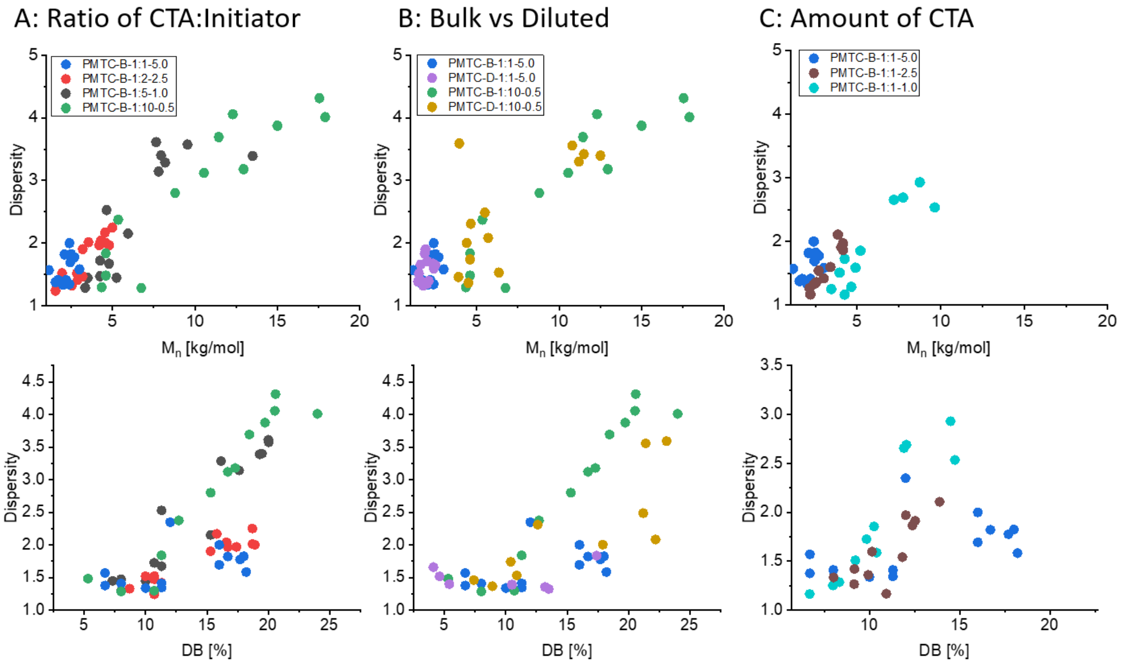

Figure S 4: Correlation of the dispersity with molar mass or DB for: A) A varying ratio of CTA:Initiator, B) a comparison between RROP in bulk and diluted; C) a varying ratio of CKA:CTA with a fixed ratio of CTA:Initiator of 1:1.

Supplementary note 3: In the following tables, details on the polymers prepared in the kinetic studies is depicted. For details on the elugrams, KMHS-plots and the NMR-data, please refer to the published raw-data. Since the KMHS-data are based on viscosimetric analysis and therefor require for a sufficient molar mass, not for all polymer samples, KMHS-alpha values could be determined. For samples, where unreliable KMHS-values could be determined, this was marked with "-".

Table S 1: Compilation of the analytics for PMTC-B-1:1-5.0#: reaction time, conversion and DB determined by ^{1}H-NMR spectroscopy and molar mass, dispersity and KMHS-alpha values determined by SEC-MALS in THF.

Spec number	t [min]	Conversion [%]	DB [%]	M_n [kg/mol]	Đ	KMHS-alpha
11	30*	-	-	-		
12	60*	25	6.7	1.2	1.6	-
13	130*	37	8.0	1.7	1.4	-
14	300*	51	10.0	2.0	1.3	-
15	1440*	89	16.7	2.1	1.8	0.580
16	20**	9	12.0	outlier	2.3	0.342
17	120**	43	6.7	1.6	1.4	-
18	180**	58	11.3	2.2	1.4	-
19	420**	55	11.3	2.4	1.3	0.494
20	1560**	93	16.0	2.5	1.7	0.521
31	720***	69	18.2	3.0	1.6	0.404
32	840***	72	18.0	2.5	1.8	0.381
33	960***	77	17.7	2.7	1.8	0.359
34	1200***	99	16.0	2.4	2.0	0.375

#ratio of CTA:initiator of 1:1 with 5 mol% concentration relative the MTC, in bulk; *first batch,** second batch,*** third batch. Green = used for end-point calculations in Table 1 of the main manuscript.

Table S 2: Compilation of the analytics for PMTC-B-1:2-2.5#: reaction time, conversion and DB determined by ^{1}H-NMR spectroscopy and molar mass, dispersity and KMHS-alpha values determined by SEC-MALS in THF.

Spec number	t [min]	Conversion [%]	DB [%]	M_n [kg/mol]	Đ	KMHS-alpha
11	30*	38	10.0	2.0	1.5	0.566
12	60*	48	10.7	2.9	1.5	0.462
13	130*	74	15.3	3.2	1.9	0.432
14	300*	91	18.7	3.6	2.0	0.397
15	1440*	95	18.7	5.0	2.3	0.382
16	20**	15	10.7	1.6	1.2	-
17	90**	38	8.7	2.6	1.3	0.684
18	180**	55	11.3	2.9	1.4	0.570
19	240**	60	10.7	3.2	1.5	0.542
20	1560**	97	16.7	4.8	2.0	0.377
31	720***	74	17.4	4.2	2.0	0.386
32	840***	88	16.6	4.3	2.0	0.377
33	960***	75	18.9	4.6	2.0	0.388
34	1200***	99	15.8	4.6	2.2	0.378

#CTA:initiator of 1:2 with 5 mol% concentration of the initiator relative the MTC, in bulk; *first batch, ** second batch, *** third batch. Green = used for end-point calculations in Table 1 of the main manuscript.

Table S 3: Compilation of the analytics for PMTC-B-1:5-1.0#: reaction time, conversion and DB determined by ^{1}H-NMR spectroscopy and molar mass, dispersity and KMHS-alpha values determined by SEC-MALS in THF.

Spec number	t [min]	Conversion [%]	DB [%]	M_n [kg/mol]	Đ	KMHS-alpha
11	30*	31	8.0	3.4	1.3	0.601
12	60*	37	10.0	3.5	1.4	0.543
13	130*	56	10.7	4.3	1.7	0.457
14	300*	88	11.3	4.7	2.5	0.384
15	1440*	96	19.3	13.5	3.4	0.372
16	20**	19	8.0	4.3	1.5	-
17	90**	61	7.3	5.3	1.4	0.505
18	180**	58	11.3	4.8	1.7	0.456
19	240**	75	15.3	6.0	2.2	0.404
20	1560**	96	20.0	7.7	3.6	0.367
31	720***	94	17.6	7.8	3.1	0.389
32	840***	89	19.5	8.0	3.4	0.382
33	960***	99	16.2	8.2	3.3	0.226
34	1200***	99	20.0	9.6	3.6	0.379

#CTA:initiator of 1:5 with 5 mol% concentration of the initiator relative the MTC, in bulk; *first batch,* second batch,*** third batch. Green = used for end-point calculations in Table 1 of the main manuscript

Table S 4: Compilation of the analytics for PMTC-B-1:10-0.5#: reaction time, conversion and DB determined by ¹H-NMR spectroscopy and molar mass, dispersity and KMHS-alpha values determined by SEC-MALS in THF.

Spec number	t [min]	Conversion [%]	DB [%]	M_n [kg/mol]	Đ	KMHS-alpha
1	30*	54	11.3	4.6	1.8	0.480
2	60*	73	12.7	5.4	2.4	0.436
3	130*	94	15.3	8.8	2.8	0.415
4	300*	96	16.7	10.6	3.1	0.402
5	1440*	96	24.0	17.9	4.0	0.402
6	5**	11	20.7	-	-	-
7	10**	25	5.3	4.6	1.5	-
8	20**	23	10.7	4.4	1.3	0.642
9	90**	43	8.0	6.8	1.3	0.545
10	1560**	92	17.3	13.0	3.2	0.375
31	720***	90	18.5	11.5	3.7	0.399
32	840***	89	20.5	12.3	4.1	0.399
33	960***	88	19.7	15.0	3.9	0.398
34	1200***	99	20.6	17.6	4.3	0.389
	Average	Endpoints		16.2±2.8		

#CTA:initiator of 1:10 with 5 mol% concentration of the initiator relative the MTC, in bulk; *first batch,** second batch,*** third batch. Green = used for end-point calculations in Table 1 of the main manuscript.

Table S 5: Compilation of the analytics for PMTC-D-1:1-5.0#: reaction time, conversion and DB determined by ¹H-NMR spectroscopy and molar mass, dispersity and KMHS-alpha values determined by SEC-MALS in THF.

Spec number	t [min]	Conversion [%]	DB [%]	M_n [kg/mol]	Đ	KMHS-alpha
1	60*	19	4.6	1.5	1.5	-
2	120*	32	5.4	2.1	1.4	-
3	240*	46	13.5	1.8	1.3	0.579
4	360*	56	13.2	2.0	1.4	0.562
5	1440*	83	17.4	1.9	1.8	0.355
6	30**	18	-	2.0	1.7	0.373
7	90**	29	4.1	1.6	1.7	-
8	180**	41	10.5	1.7	1.4	-
9	300**	50	10.5	1.5	1.4	-
10	1440**	85	-	1.9	1.9	0.376
31	720***	-	-	2.4	1.6	0.390
32	840***	-	-	2.4	1.7	0.381
33	960***	-	-	2.5	1.6	0.396

#CTA:initiator of 1:1 with 5 mol% concentration of the initiator relative the MTC, 50 % diluted; *first batch, ** second batch,*** third batch. Green = used for end-point calculations in Table 1 of the main manuscript.

Table S 6: Compilation of the analytics for PMTC-D-1:10-0.5#: reaction time, conversion and DB determined by 1H-NMR spectroscopy and molar mass, dispersity and KMHS-alpha values determined by SEC-MALS in THF.

Spec number	t [min]	Conversion [%]	DB [%]	M_n [kg/mol]	Đ	KMHS-alpha
1	60*	41	8.9	4.5	1.4	-
2	120*	56	10.9	6.4	1.5	0.526
3	240*	71	17.9	4.4	2.0	0.434
4	360*	79	22.2	5.7	2.1	0.422
5	1440*	98	23.1	4.0	3.6	0.404
6	30**	30	7.4	3.9	1.5	0.521
7	90**	51	10.4	4.6	1.7	0.476
8	180**	62	12.6	4.7	2.3	0.437
9	300**	84	21.2	5.5	2.5	0.393
10	1440**	98	21.4	10.8	3.6	0.370
31	720***	-	-	11.2	3.3	0.394
32	840***	-	-	11.5	3.4	0.395
33	960***	-	-	12.5	3.4	0.402

#CTA:initiator of 1:10 with 5 mol% concentration of the initiator relative the MTC, 50 % diluted; *first batch,* second batch,*** third batch. Green = used for end-point calculations in Table 1 of the main manuscript

Table S 7: Compilation of the analytics for PMTC-B-1:1-2.5#: reaction time, conversion and DB determined by ^{1}H-NMR spectroscopy and molar mass, dispersity and KMHS-alpha values determined by SEC-MALS in THF.

Spec number	t [min]	Conversion [%]	DB [%]	M_n [kg/mol]	Đ	KMHS-alpha
1	60*	24	10.9	2.2	1.2	-
2	120*	34	8.0	2.5	1.3	0.654
3	240*	54	9.2	3.0	1.4	0.503
4	360*	64	10.1	3.4	1.6	0.440
5	1440*	95	12.9	-	-	-
6	30**	13		-	-	-
7	90**	26	9.2	2.2	1.3	0.777
8	180**	43	9.9	2.6	1.4	0.566
9	300**	49	11.8	2.7	1.5	0.463
10	1440**	95	14.7	-	-	-
31	720***	99	12.4	4.2	1.9	0.397
32	840***	99	12.5	4.1	1.9	0.386
33	960***	99	12.0	4.2	2.0	0.395
34	1200***	99	13.9	3.9	2.1	0.384

#CTA:initiator of 1:1 with 2.5 mol% concentration of the initiator relative the MTC, 50 % diluted; *first batch,* second batch,*** third batch. Green = used for end-point calculations in Table 1 of the main manuscript

Table S 8: Compilation of the analytics for PMTC-B-1:1-1.0#: reaction time, conversion and DB determined by ^{1}H-NMR spectroscopy and molar mass, dispersity and KMHS-alpha values determined by SEC-MALS in THF.

Spec number	t [min]	Conversion [%]	DB [%]	M_n [kg/mol]	Đ	KMHS-alpha
1	60	26	6.7	4.3	1.2	-
2	120	47	8.3	4.7	1.3	0.583
3	240	54	10.4	4.9	1.6	0.478
4	360	70	10.3	5.2	1.9	0.434
5	1440	96	13.2	-	-	-
6	30	11		-	-	-
7	90	28	8.0	3.5	1.3	0.658
8	180	44	9.2	4.0	1.5	0.489
9	300	55	9.8	4.3	1.7	0.458
10	1440	96	13.9	-	-	-
31	720	99	11.9	7.2	2.7	0.397
32	840	99	12.0	7.8	2.7	0.393
33	960	99	14.7	9.7	2.5	0.397
34	1200	99	14.5	8.8	2.9	0.382

#CTA:initiator of 1:10 with 5 mol% concentration of the CTA relative the MTC, 50 % diluted; *first batch,* second batch,*** third batch. Green = used for end-point calculations in Table 1 of the main manuscript.

3. Chain-extension and block copolymers

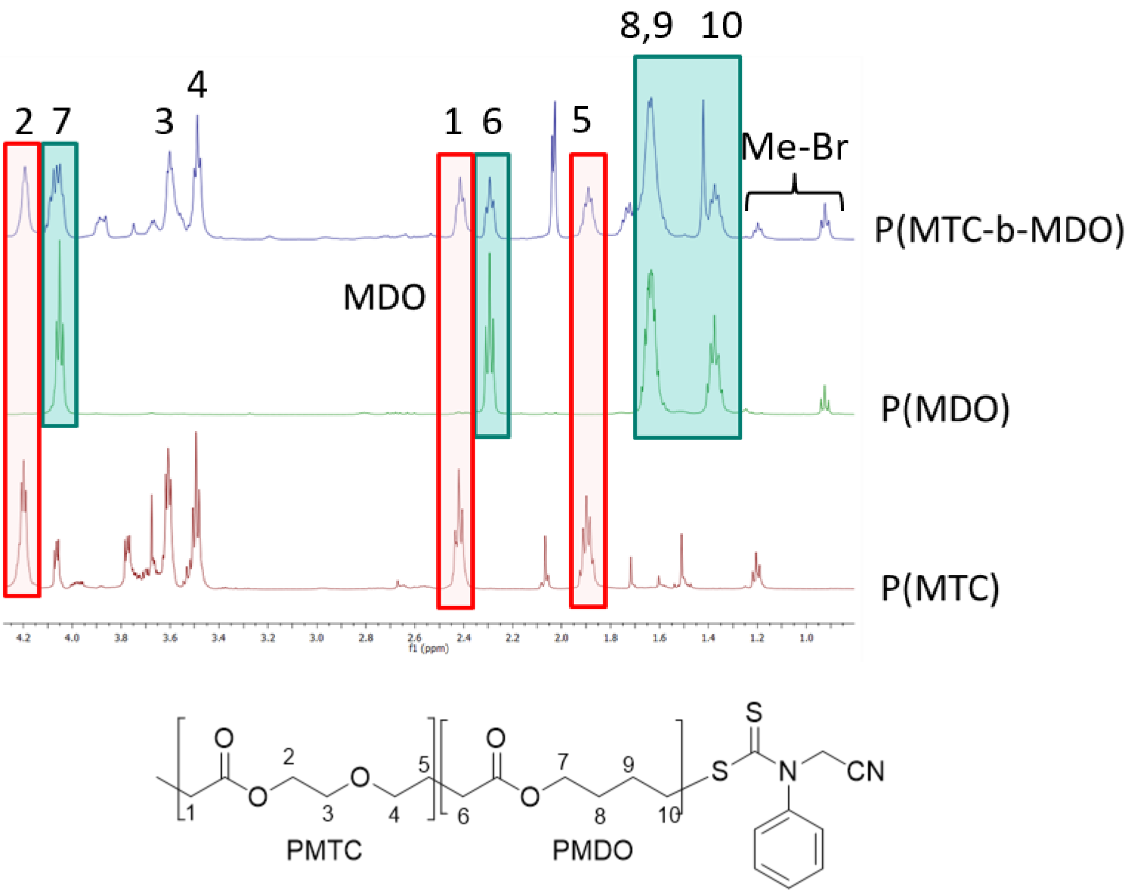

Figure S 5: ^{1}H-NMR of the chain-extended P(MTC-b-MDO) with the respective signals for PMDO and PMTC.

Table S 9: Summary of the molar mass number averages M_n and distributions ($Đ = M_w/M_n$)

**Short Name**	Polymer	Mn	Dispersity
**DO**	PMTC-B-1:1:10 for DOSY	5.5 kg/mol	1.50
**CE1**	Macro-RAFT	7.5 kg/mol	1.30
**CE2**	P(MTC-*b*-MTC)	12.4 kg/mol	2.08
**CE3**	P(MTC-*b*-MDO)	11.0 kg/mol	2.31

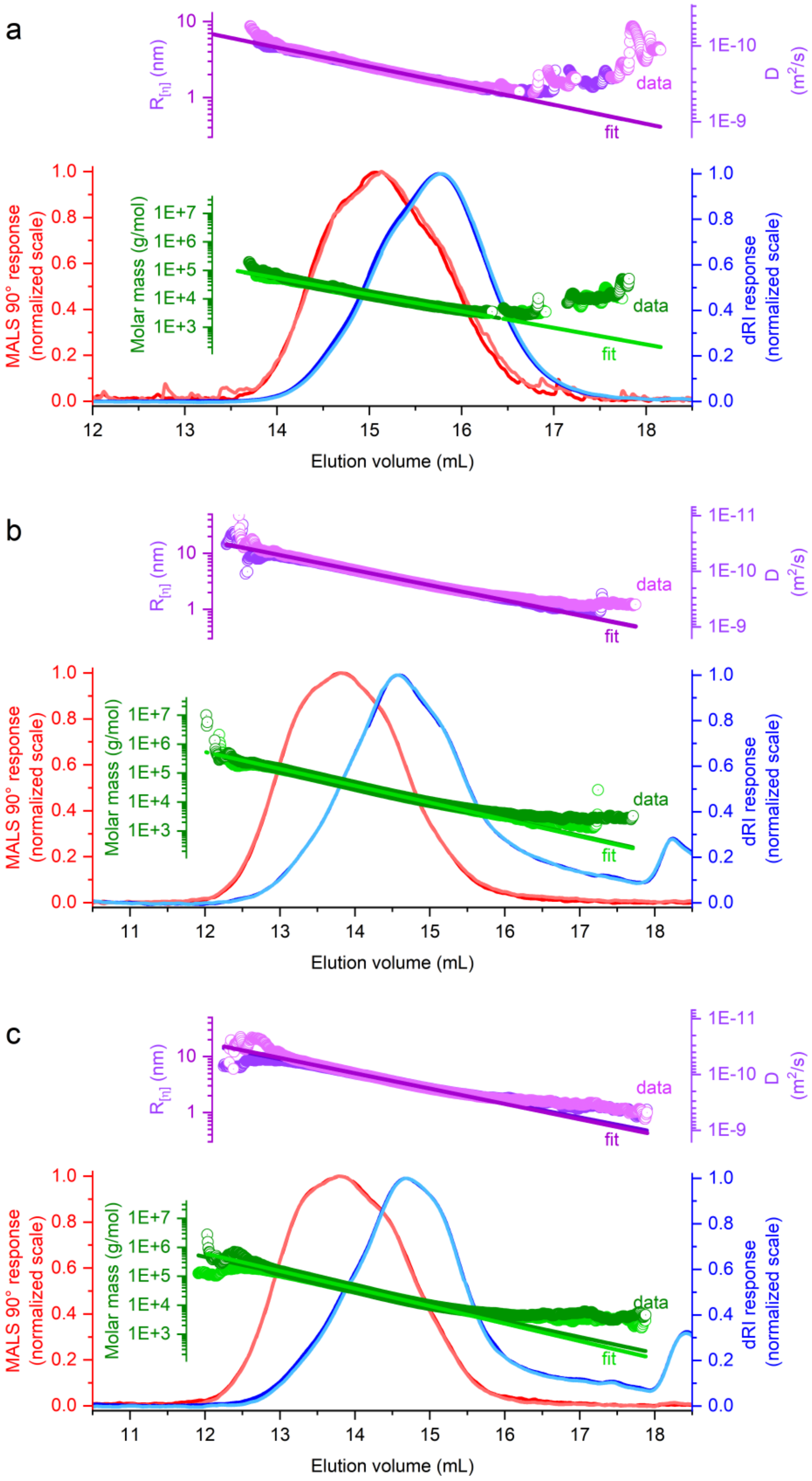

*Figure S6: Elugrams from triple-detection SEC of PMTC-B-1:1-10.0 (a), of P(MTC-*b*-MTC) (b) and of P(MTC-*b*-MDO) (c), measured in THF at 25 °C. The diffusion axis was calculated based on the measured viscosity radius $R_{[\eta]}$ (hydrodynamic radius) and the dynamic viscosity of THF at 25 °C η_{THF} = 0.456 mL/g. The diffusion coefficients are in comparable order of magnitude to the diffusion coefficients measured by DOSY NMR, since the dynamic viscosity of Chloroform at 30 °C is relatively close to the one of THF with η_{CHL} = 0.513 mL/g.[5x] The difference in viscosity between chloroform and deuterated chloroform is insignificant.*

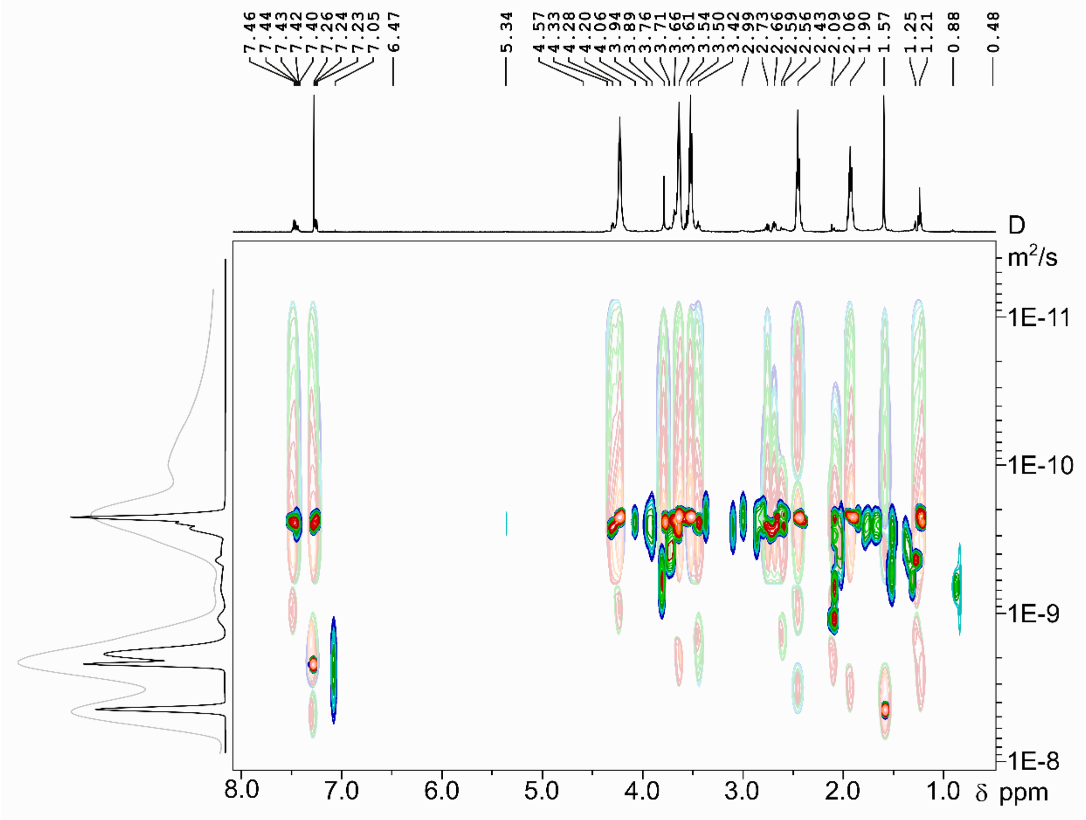

Figure S7: 1H DOSY of PMTC-B-1:1-10.0 in $CDCl_3$ processed as multicomponent fit, superimposed to the ILT fit (pale) as show in Fig. 2 of the main article.

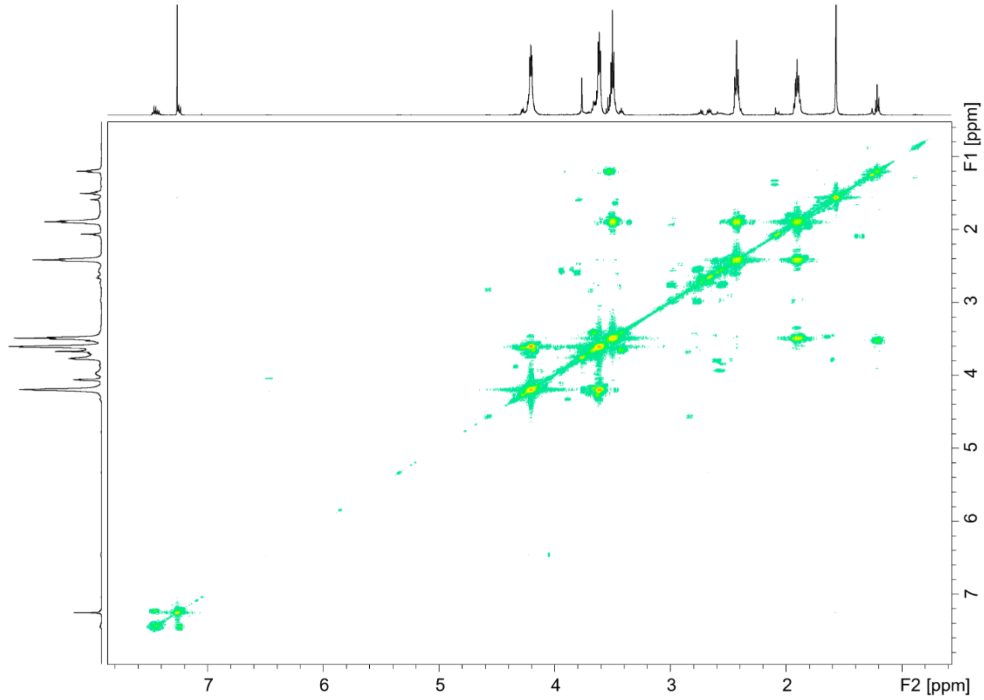

Figure S8: $^{1}H,^{1}H$ COSY of PMTC-B-1:1-10.0 in $CDCl_3$.

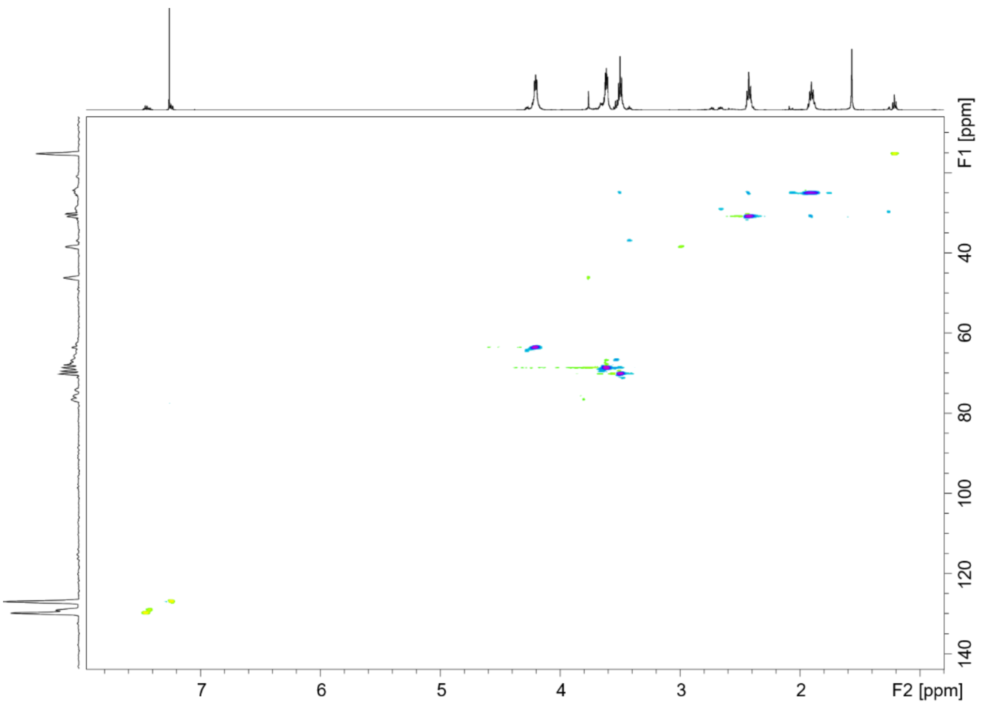

Figure S9: ^{1}H,^{13}C HSQC of PMTC-B-1:1-10.0 in $CDCl_3$.

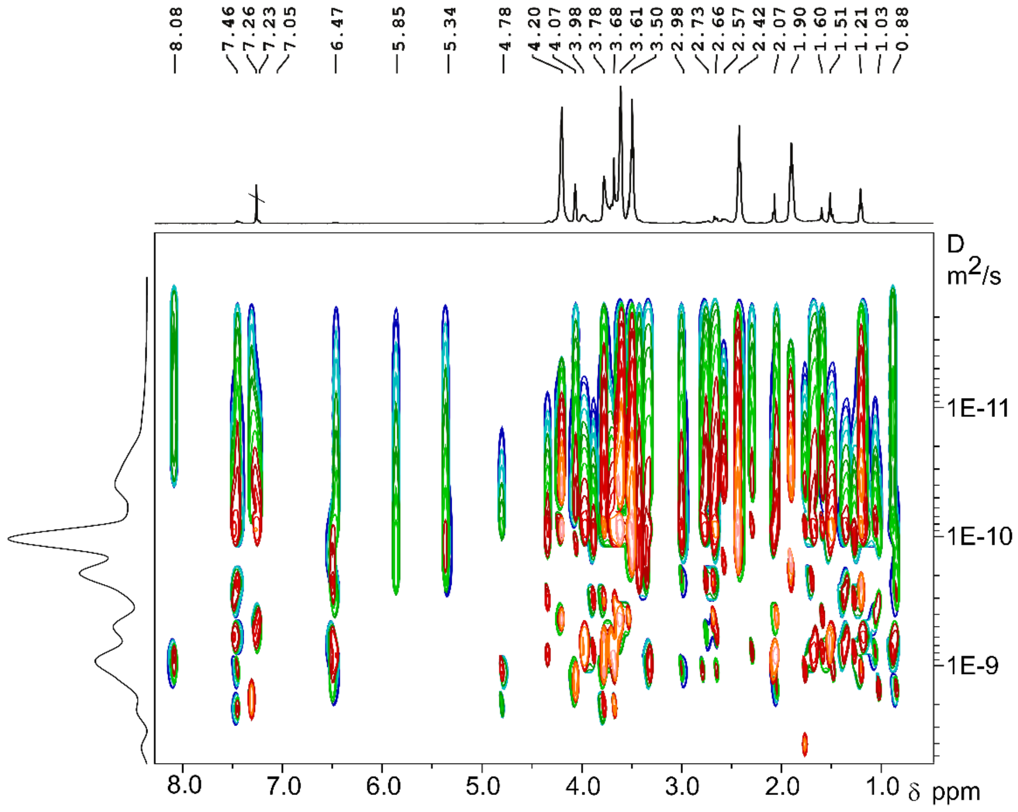

Figure S10: 1H DOSY of P(MTC-b-MTC) in $CDCl_3$ processed as ILT fit.

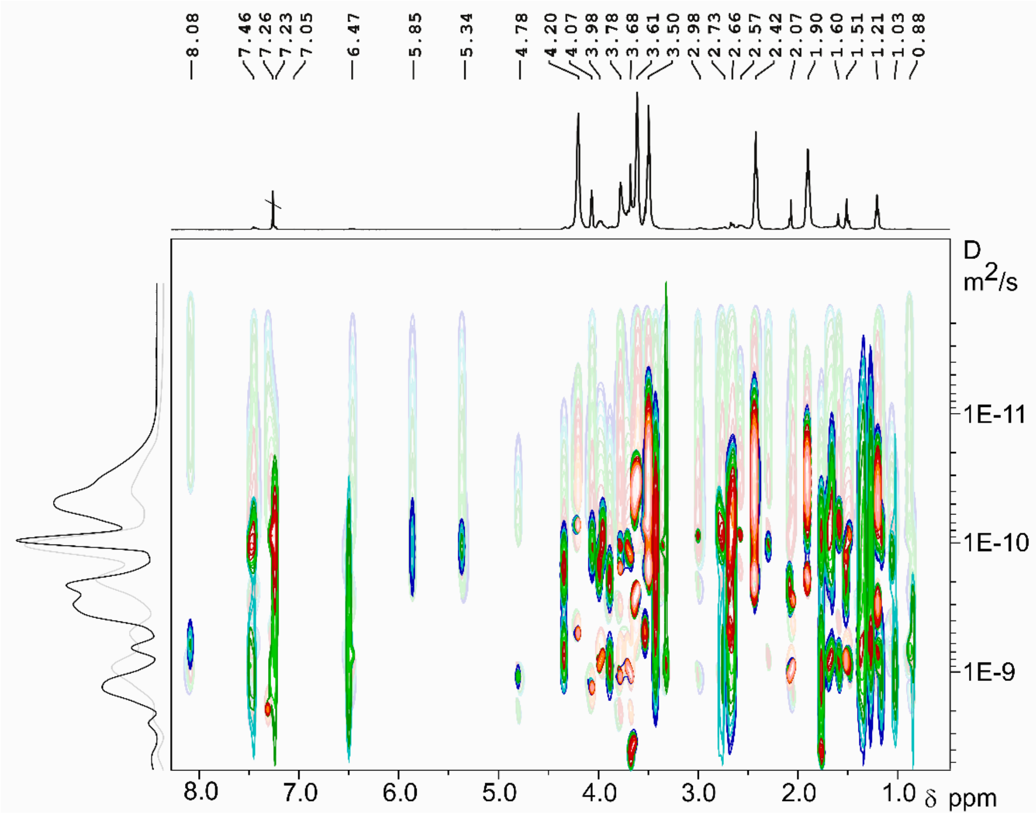

*Figure S11: 1H DOSY of P(MTC-*b*-MTC) in $CDCl_3$ processed as multicomponent fit, superimposed to the ILT fit (pale) as show in Fig. S9.*

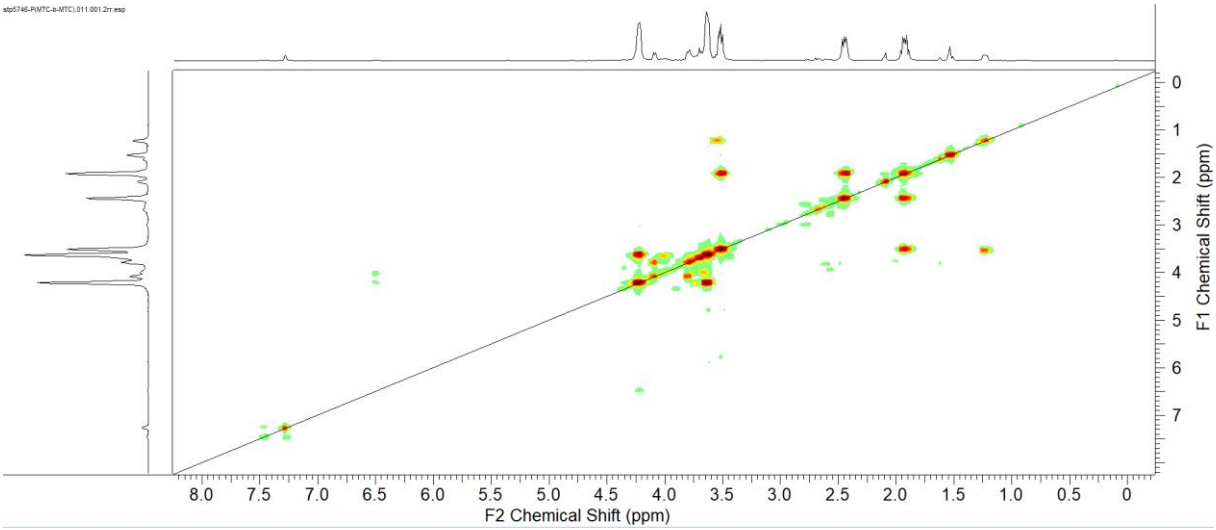

*Figure S 12: 1H,1H COSY of P(MTC-*b*-MTC) in $CDCl_3$.*

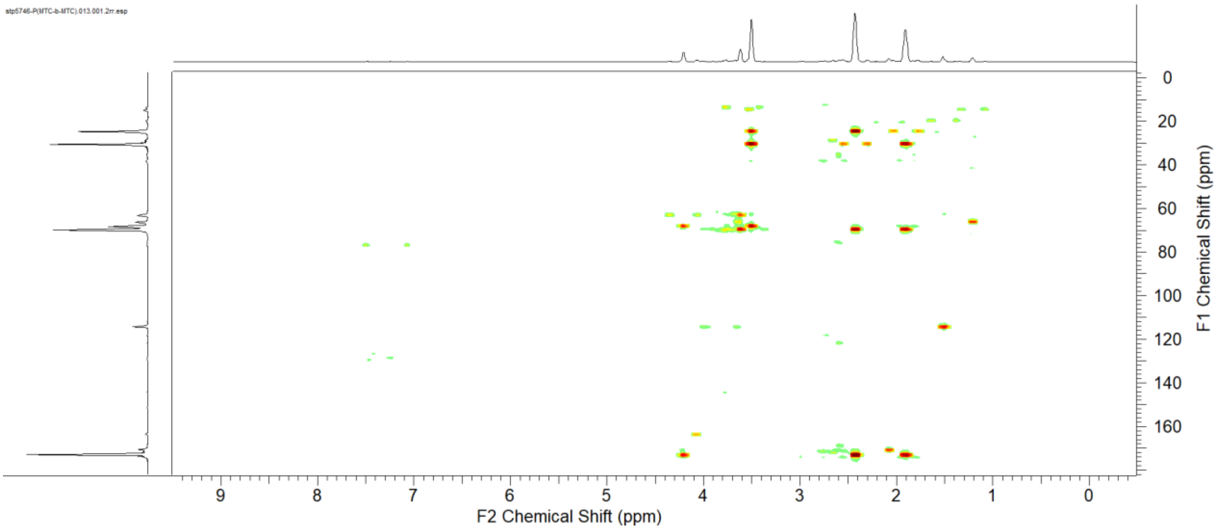

*Figure S 13: 1H,13C HMBC of P(MTC-*b*-MTC) in $CDCl_3$.*

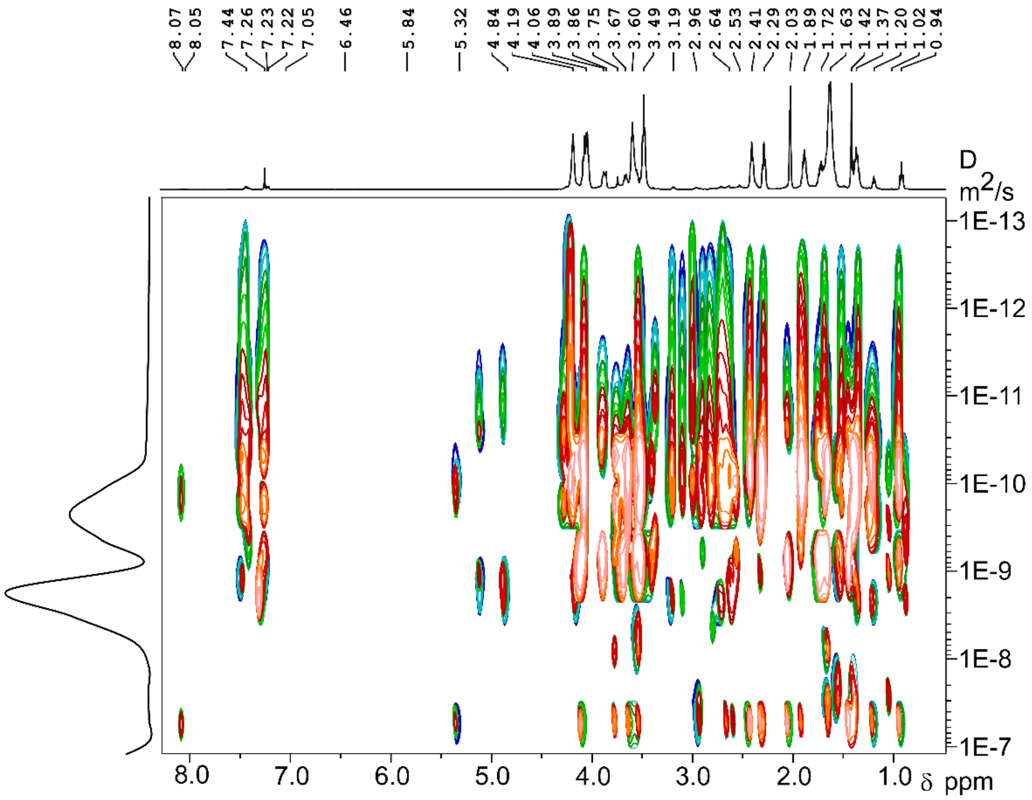

*Figure S 14: 1H DOSY of P(MTC-*b*-MDO) in CDCl₃ processed as ILT fit.*

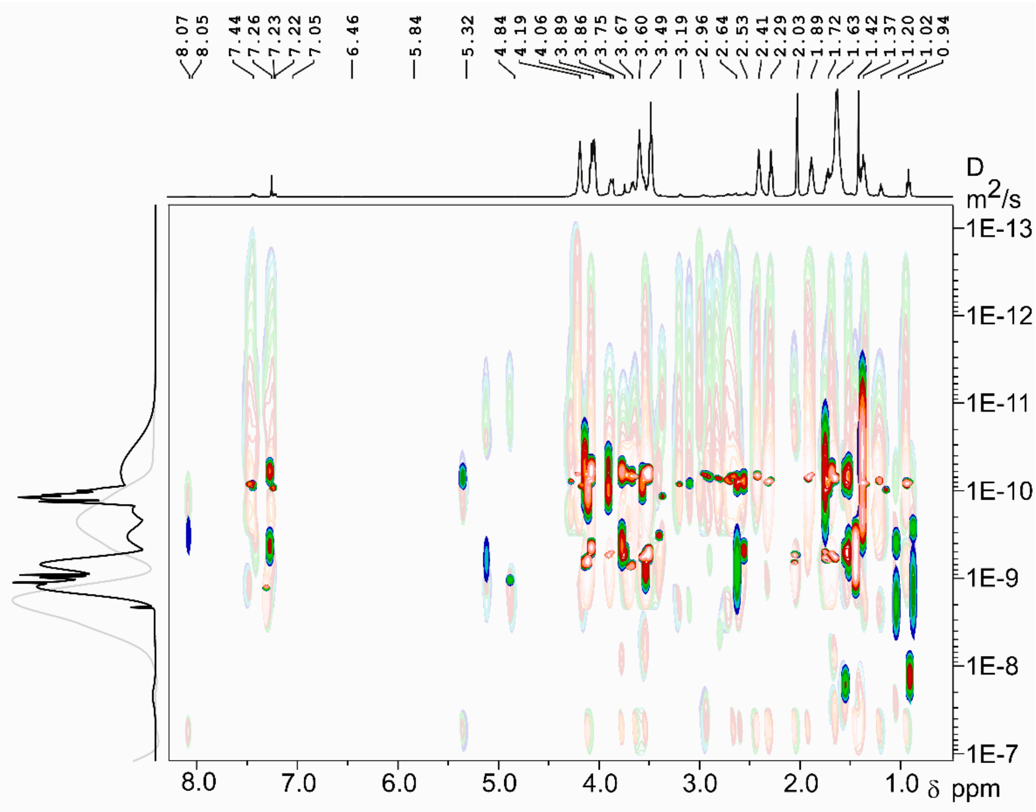

*Figure S 15: 1H DOSY of P(MTC-*b*-MDO) in $CDCl_3$ processed as multicomponent fit, superimposed to the ILT fit (pale) as show in Fig. S13.*

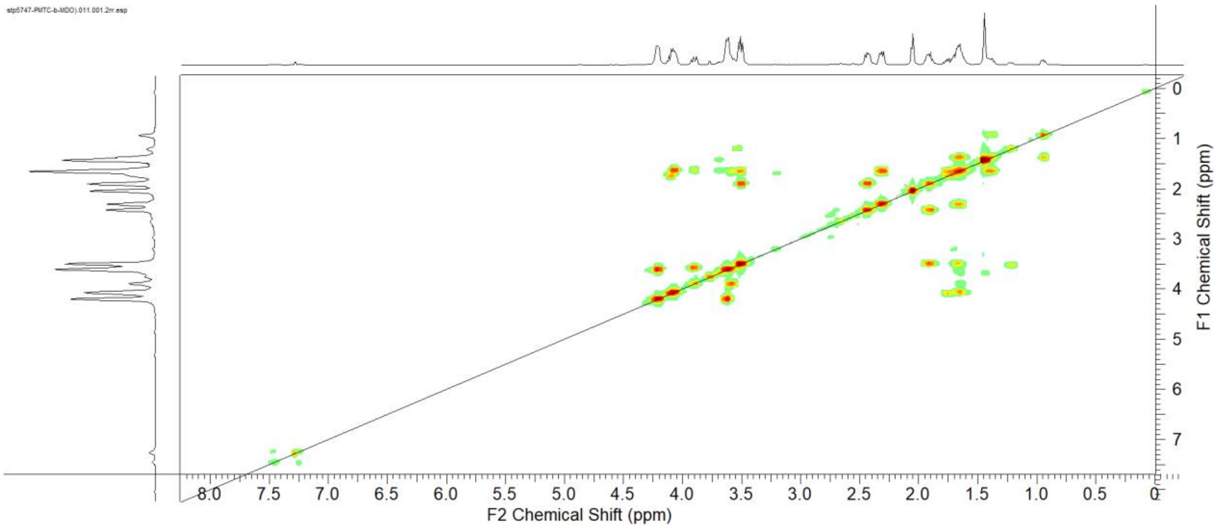

*Figure S 16: 1H,1H COSY of P(MTC-*b*-MDO) in $CDCl_3$.*

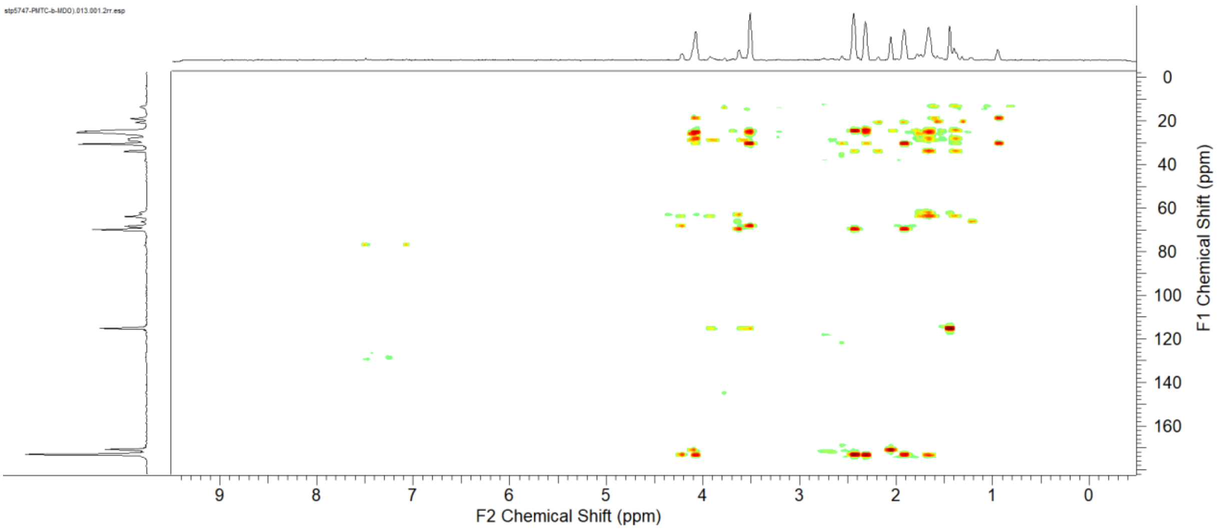

Figure S 17: 1H,13C HMBC of P(MTC- b*-MDO) in $CDCl_3$.*

DSC of the block-copolymers:

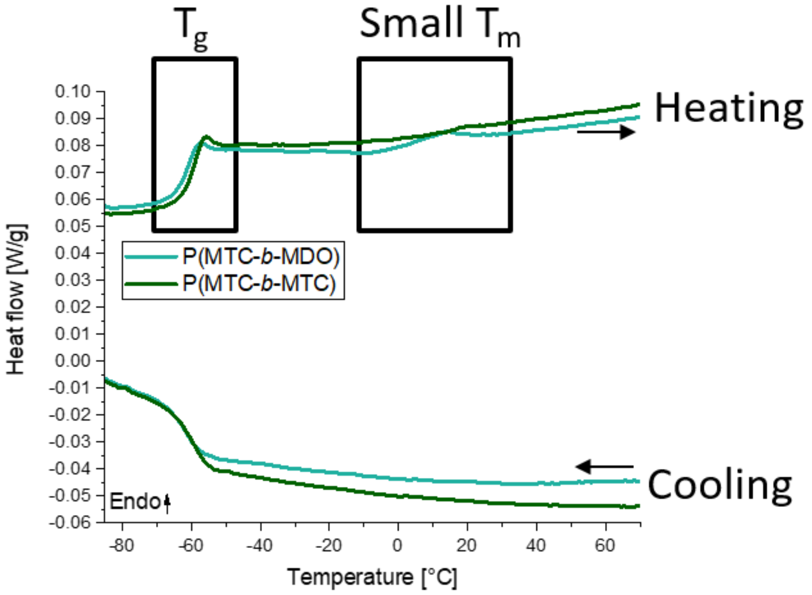

*Figure S 18: Thermograms (DSC) of the block-copolymers where both show a glass transition temperature, but PMTC-*b*-PMDO also shows a small, but notable melting temperature, which is not visible in the cooling curves*

Supplementary note 4: DSC was conducted on a DSC Q2000 / TA Instruments using a nitrogen atmosphere and a standard Al DSC-pan. The following heating cycle was used:

Equilibrate at -90.00°C; Modulate Temperature 0,31 °C every 40 sec; Isothermal for 5.00 min; 1: Ramp 2.00°C/min to 80.00°C; Isothermal for 0,50 min; 2: Ramp 2.00°C/min to -90.00°C; Isothermal for 5 min; 3: Ramp 2.00°C/min to 80.00°C. The second heating cycle is shown in Fig. S18 with supplementary note 4.

4. Formulation and self-assembly

Supplementary note 5: Degradation by lipase was probed by fluorescence spectroscopy with Nile red as a fluorophore. It was co-precipitated with the polymer and thus encapsulated in the NPs. Since the dye fluoresces only in a hydrophobic environment, a reduction in fluorescence intensity is used as a measure of the degradation alongside a "drug release" study. The NPs were kept in saline solution as a reference and in a solution of saline + enzyme for the degradation study. Following the reference, a constant fluorescence intensity observed for all samples also served as a reference to track the leaching of the model-drug and to test the overall stability of the NPs. (see DLS in section 5)

Protocol: NPs as described in main manuscript (Formulation of PCKA-based nanoparticles) were measured with Malern Zetasizer Nano as prepared and evaluated using the Intensity- and number-%.

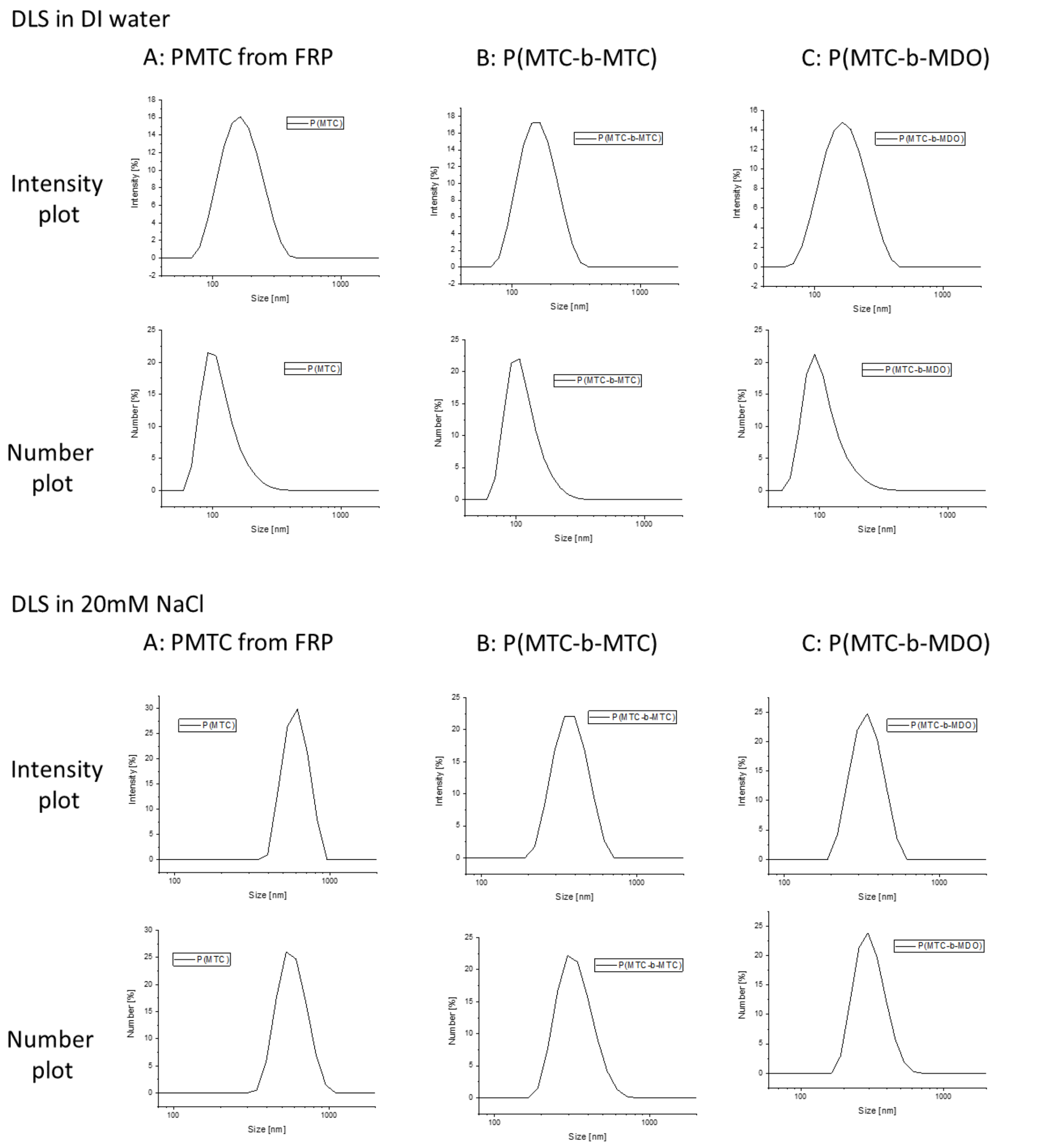

*Figure S 19: DLS traces of polymers from frp as comparison and the chain-extended P(MTC-*b*-MTC) as well as P(MTC-*b*-MDO) NPs from nanoprecipitation in DI water (top) and 20mM NaCl (bottom).*

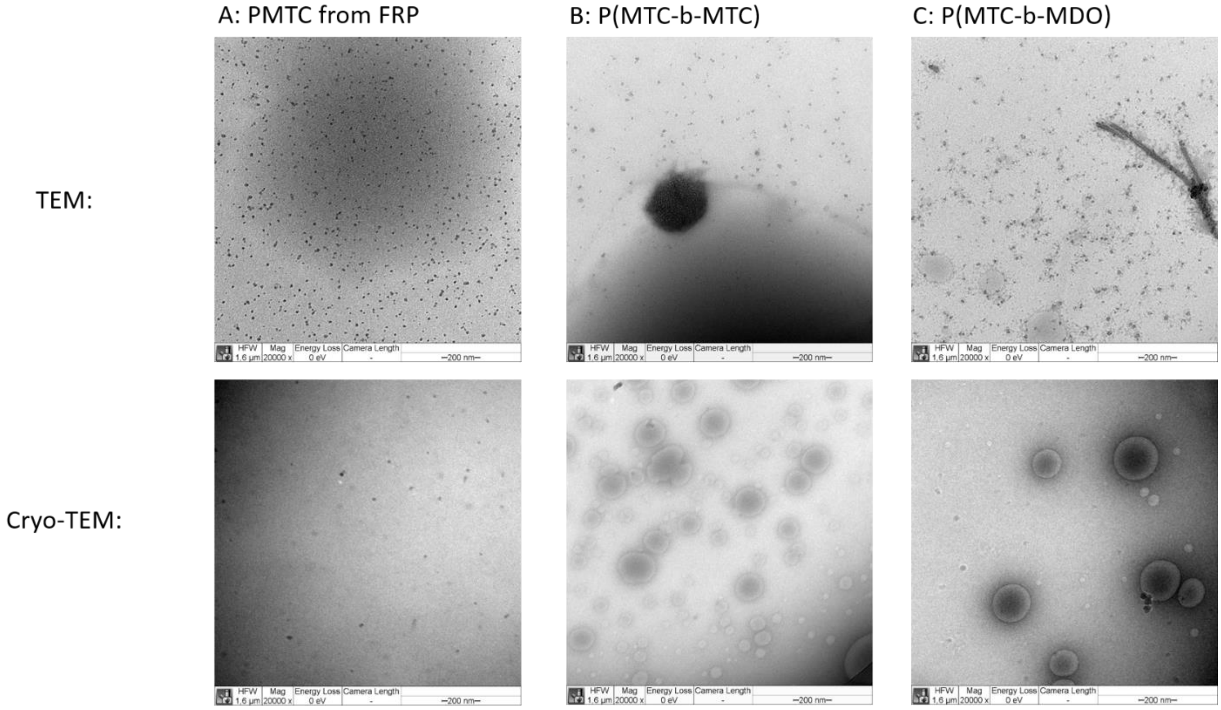

*Figure S 20: TEM and cryo-TEM of nanoparticles prepared by nanoprecipitation of A) a PMTC from FRP, B) a P(MTC-*b*-MTC)-block copolymer and C) a P(MTC-*b*-MDO) block copolymer.*

Methodology (adapted from an established protocol)[6]:

Supplementary note 6: TEM images were recorded with a Libra120 (Carl Zeiss Microsopy GmbH, Oberkochen, Germany) operated at 120 kV. Images were recorded in bright field at small defocus to improve the contrast. For the measurement, 2 µL of sample solution were dripped on copper grids coated with a formvar/carbon foil. After a waiting time of 2 minutes, the sample was blotted with filter paper to remove excess specimen not adsorbed on the grid. Afterwards, 2 µL staining solution of 2 wt% uranyl acetate in water were dripped on the grid and excess staining solution was blotted with a filter paper after one minute.

Cryo-TEM images were acquired using the same equipment as for the TEM measurements. 2 µL of sample solution were dropped on the holey grids (Qunatifoil type R3.5/1), blotted with filter paper after 5 seconds, and rapidly frozen in liquid ethane at −178 °C using a Leica GP (Grid Plunging) device (Leica Microsystems GmbH, Wetzlar, Germany). All images were recorded in bright field at −172 °C.

As depicted in Figure S5, the NPs could not be detected by a TEM in a dry phase. Likely due to the drying process, the water, that stabilised the swollen NPs was removed and the polymer-water-interactions removed. Therefore, the NPs disassembled and instead of the detected NPs in the range of 150-200 nm observed in the DLS, smaller particles and polymer-aggregates were observed. Note, that in the P(MTC-b-MDO)-sample some particles seemed to remain stable. However, these

observations were not reproducible for the polymer-sample and should be considered a hint towards the shape of the NPs, but treated with caution.

In the cryo-TEM, the swollen NPs in solution are shock frozen (the freezing rate of thousands of Kelvin per second) which allowed to circumvent the destabilising drying-process. However, due to the fact that water was the main component of the cryo-image, a contrast between the swollen polymer-NPs (with a high water content) and the surrounding water matrix was very low. As depicted for the PMTC from FRP, no particles could be found in the cryo-TEM suggesting a high water content in these NPs. The NPs from the block copolymers P(MTC-b-MTC) and P(MTC-b-MDO) could be observed in the cryo-TEM likely due to a lower water-content and a phase-separation allowing for a higher polymer-concentration and therefore a better contrast. However, the number of stable NPs, that were observed with this method remained limited, but allowed to validate the DLS-results.

5. Degradation by Lipase from Pseudomonas cepacia

Supplementary note 7: To validate the enzymatic degradation test with fluorescence spectroscopy, the samples before and after degradation were measured by SEC in THF.

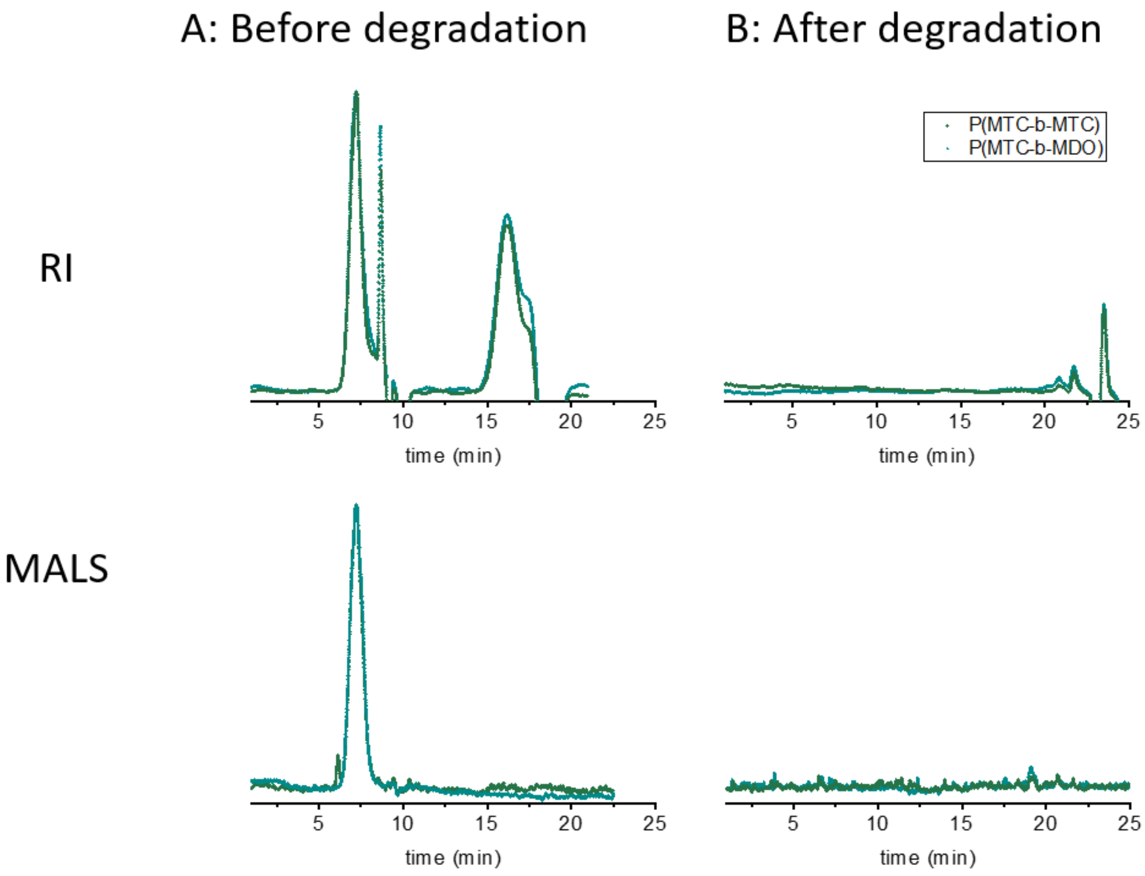

Figure S 21: SEC elugrams of the polymers and the degradation products in THF from the enzymatic degradation discussed by fluorescence-spectroscopy.

As observed by the disappearance of the polymer signals in the elugram, the polymers could successfully be degraded (see Figure S21).

6. References

1. F. Mehner, B. Hopkins, M. Reynolds-Green, D. J. Keddie, S. M. Howdle and J. Gaitzsch, Supercritical RROP: Exploring the radical ring-opening polymerisation of 2-methylene-1,3,6-trioxocane in supercritical CO2 as a green solvent, *Polymer*, 2024, **309**.
2. F. Mehner, M. Geisler, K. Arnhold, H. Komber and J. Gaitzsch, Structure–Property Relationships in Polyesters from UV-Initiated Radical Ring-Opening Polymerization of 2-Methylene-1,3-dioxepane (MDO), *ACS Applied Polymer Materials*, 2022, **4**, 7891-7902.
3. F. Mehner, T. Meissner, A. Seifert, A. Lederer and J. Gaitzsch, Kinetic studies on the radical ring-opening polymerization of 2-methylene-1,3,6-trioxocane, *Journal of Polymer Science*, 2023, **61**, 1882-1892.
4. Y. Deng, F. Mehner and J. Gaitzsch, Current Standing on Radical Ring-Opening Polymerizations of Cyclic Ketene Acetals as Homopolymers and Copolymers with one another, *Macromol Rapid Commun*, 2023, **44**, e2200941.
5. J. Gmehling, Chloroform Dynamic Viscosity (vis_c47) and Tetrahydrofuran Dynamic Viscosity (vis_c159), *SpringerMaterials database*, 2024, accessed 30/10/2025.
6. C. Bunk, H. Komber, M. Lang, N. Fribiczer, M. Geisler, P. Formanek, L. Jakisch, S. Seiffert, B. Voit and F. Böhme, Amphiphilic tetra-PCL-b-PEG star block copolymers using benzoxazinone-based linking groups, *Polym. Chem.*, 2023, **14**, 1965-1977.
