# Supplementary material for: RAFT enables controlled radical ring-opening polymerisation of cyclic ketene acetals for degradable nanoparticles
Source: Commun Chem. 2026 Apr 9;9:156. doi: 10.1038/s42004-026-01997-6 (PMC13069079; doi:10.1038/s42004-026-01997-6)
Supplement: Supplementary file 2 — Description of Additional Supplementary Files [file 42004_2026_1997_MOESM2_ESM.pdf]

Description of Additional Supplementary Files:

File: Supplementary Data

Description: All NMR spectra used in this manuscript
