# Supplementary material for: RAFT enables controlled radical ring-opening polymerisation of cyclic ketene acetals for degradable nanoparticles
Source: Commun Chem. 2026 Apr 9;9:156. doi: 10.1038/s42004-026-01997-6 (PMC13069079; doi:10.1038/s42004-026-01997-6)

Key to samples in manuscript vs in this file:

DR1: PMTC-B-1:5-1.0

DR2: PMTC-B-1:2-2.5

DR3: PMTC-B-1:1-5.0

DR4: PMTC-B-1:10-0.5

DR5: PMTC-D-1:1-5.0

DR6: PMTC-D-1:10-0.5

DR7: PMTC-B-1:1-1.0

DR8: PMTC-B-1:1-2.5

PMTC-B-1:1-10.0 is included in the supporting information

CE1 = DRR3-4

CE2 and CE3 are included in the supporting information

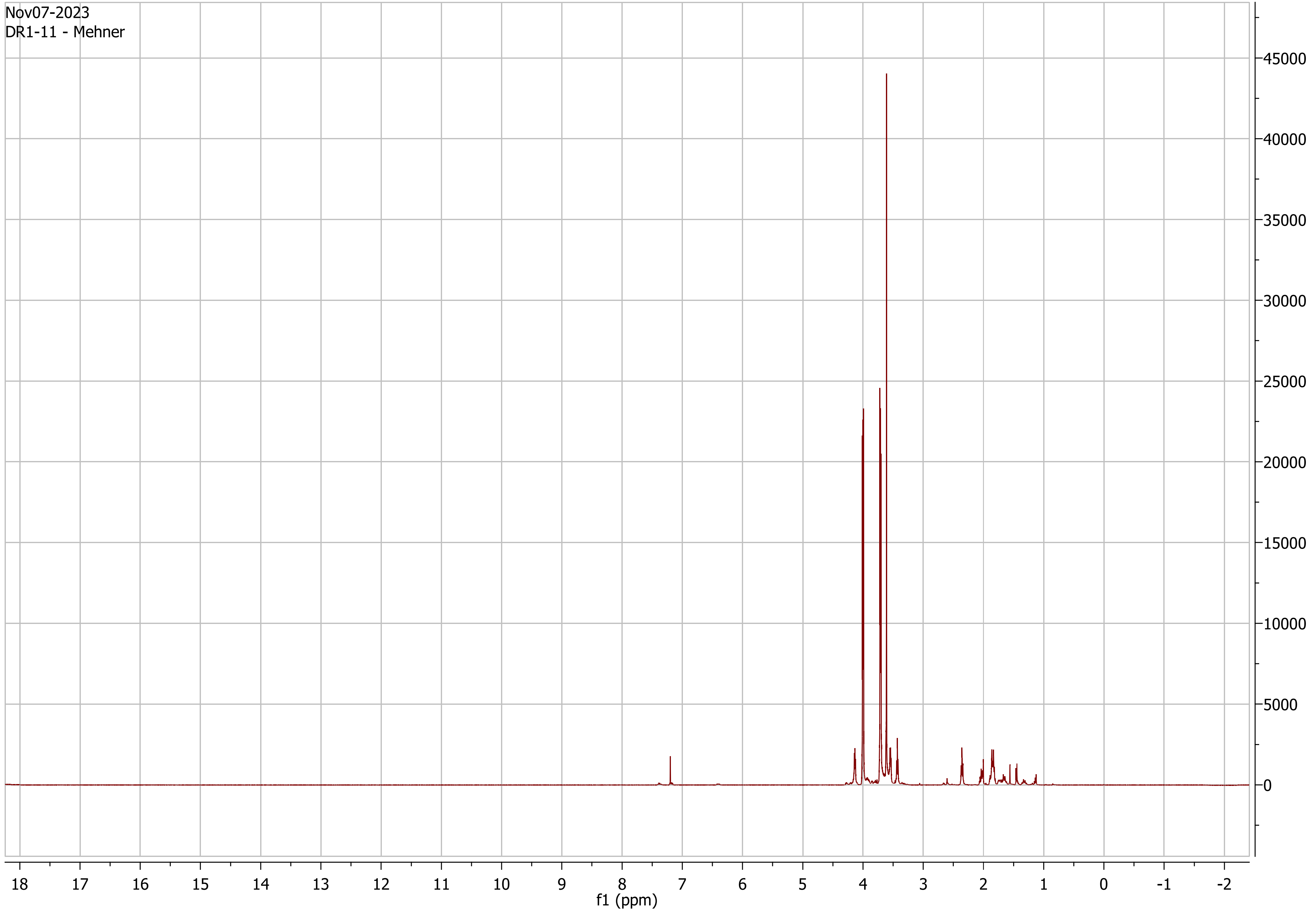
Nov07-2023
DR1-11 - Mehner
45000
40000
35000
30000
25000
20000
15000
10000
5000
0
18 17 16 15 14 13 12 11 10 9 8 7 6 5 4 3 2 1 0 -1 -2
f1 (ppm)

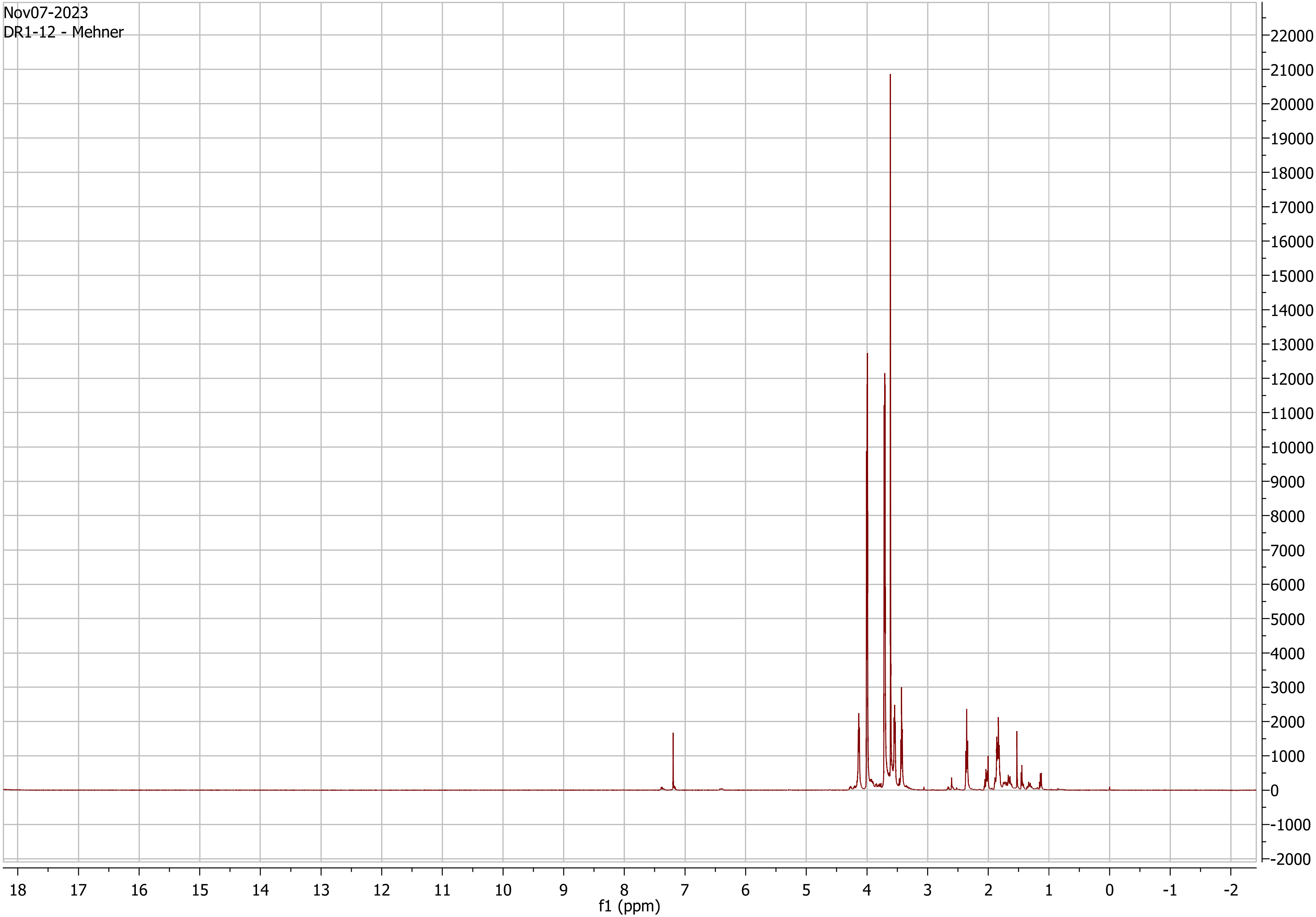
Nov07-2023
DR1-12 - Mehner
22000
21000
20000
19000
18000
17000
16000
15000
14000
13000
12000
11000
10000
9000
8000
7000
6000
5000
4000
3000
2000
1000
0
-1000
-2000
18 17 16 15 14 13 12 11 10 9 8 7 6 5 4 3 2 1 0 -1 -2
f1 (ppm)

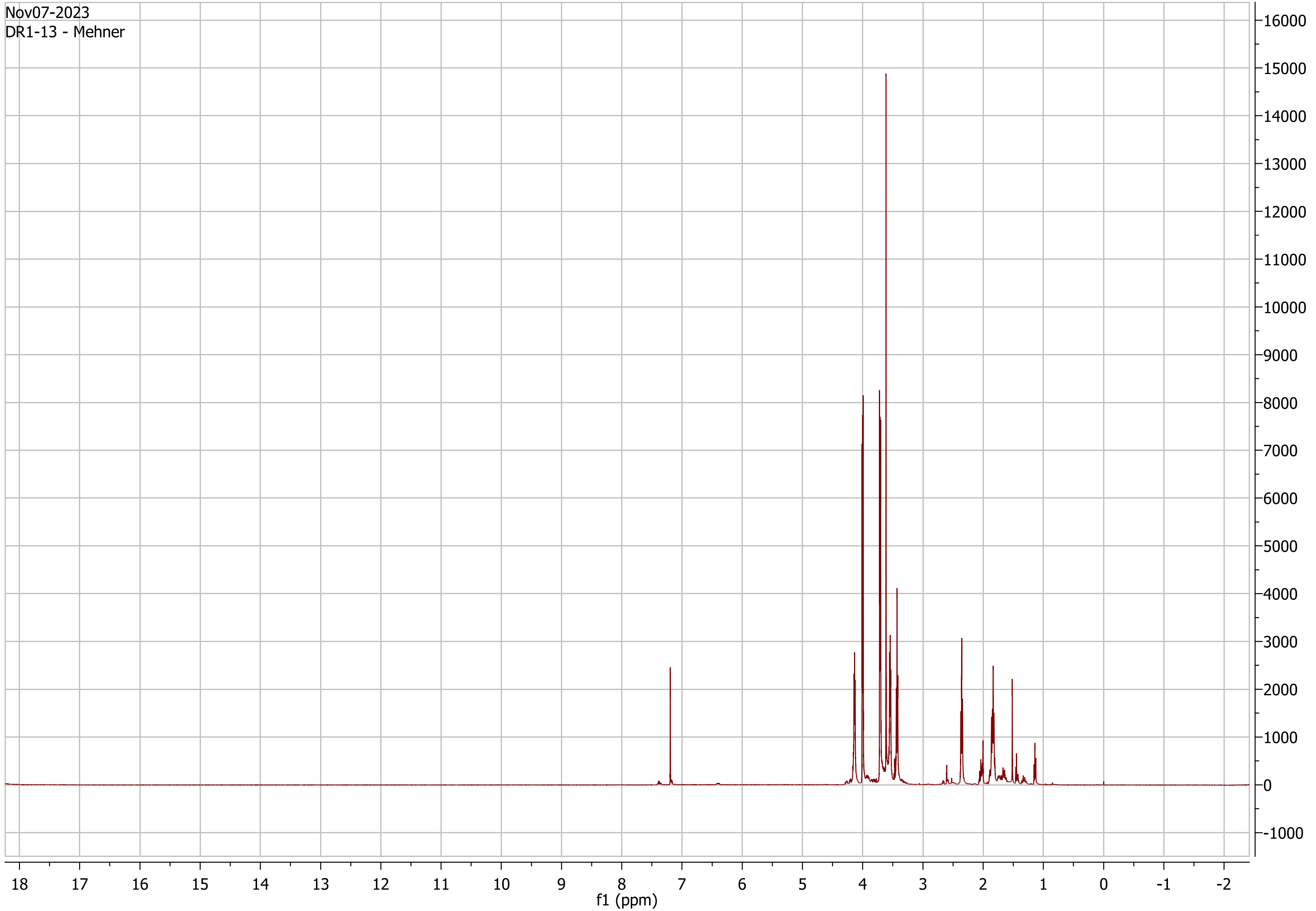

Nov07-2023
DR1-13 - Mehner
f1 (ppm)

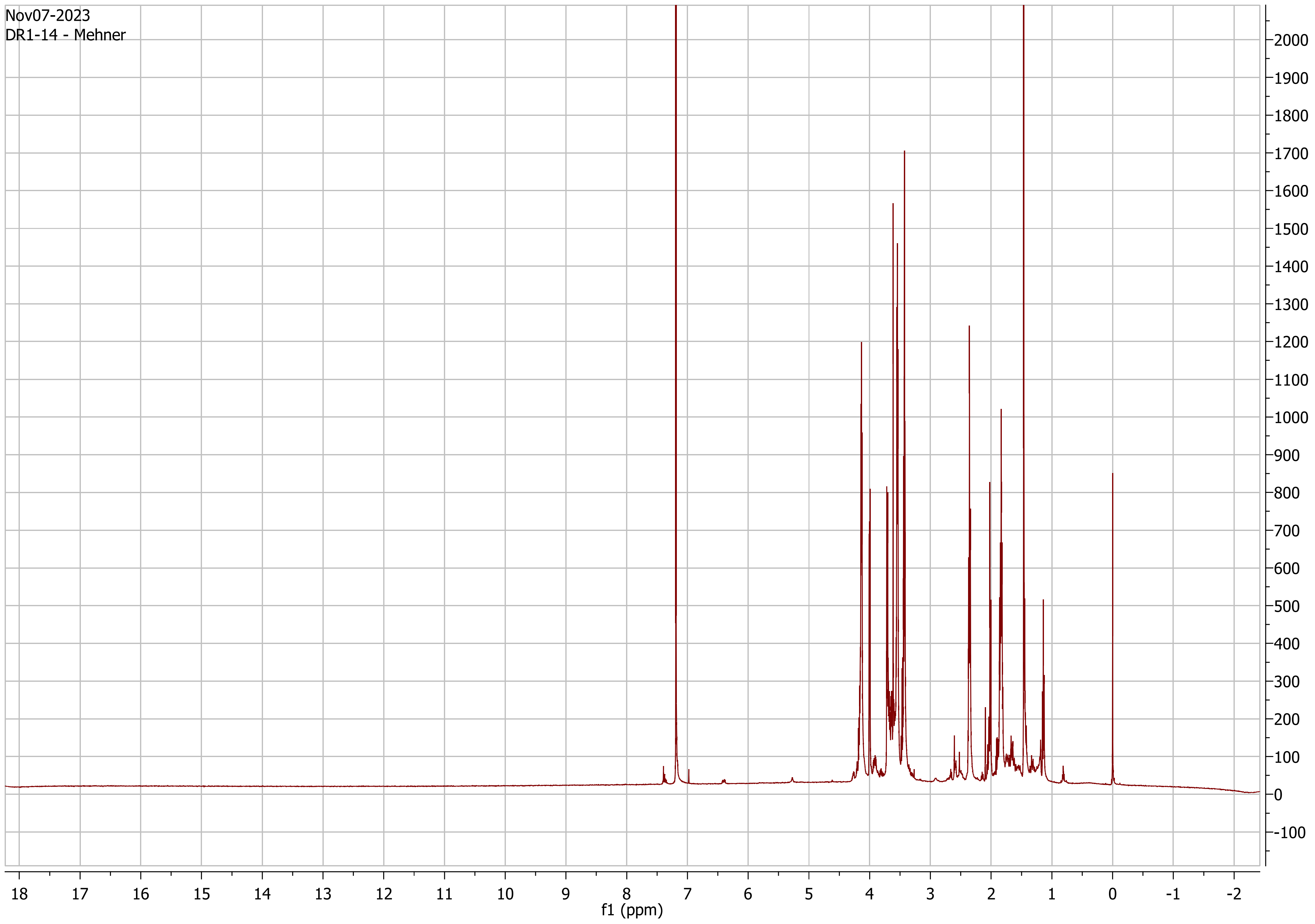

Nov07-2023
DR1-14 - Mehner
f1 (ppm)

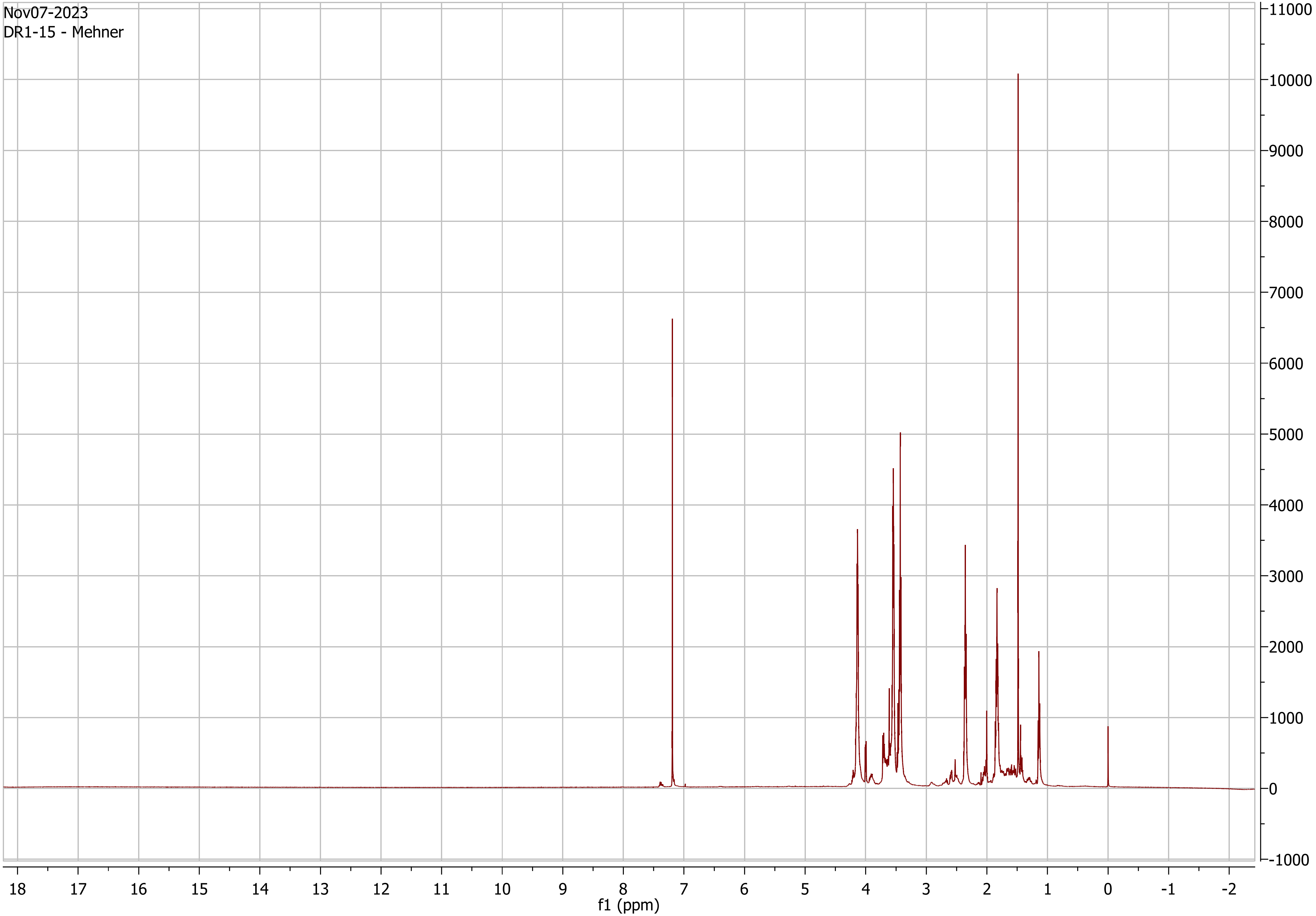
Nov07-2023
DR1-15 - Mehner
f1 (ppm)

Nov14-2023
DR1-16 -Mehner

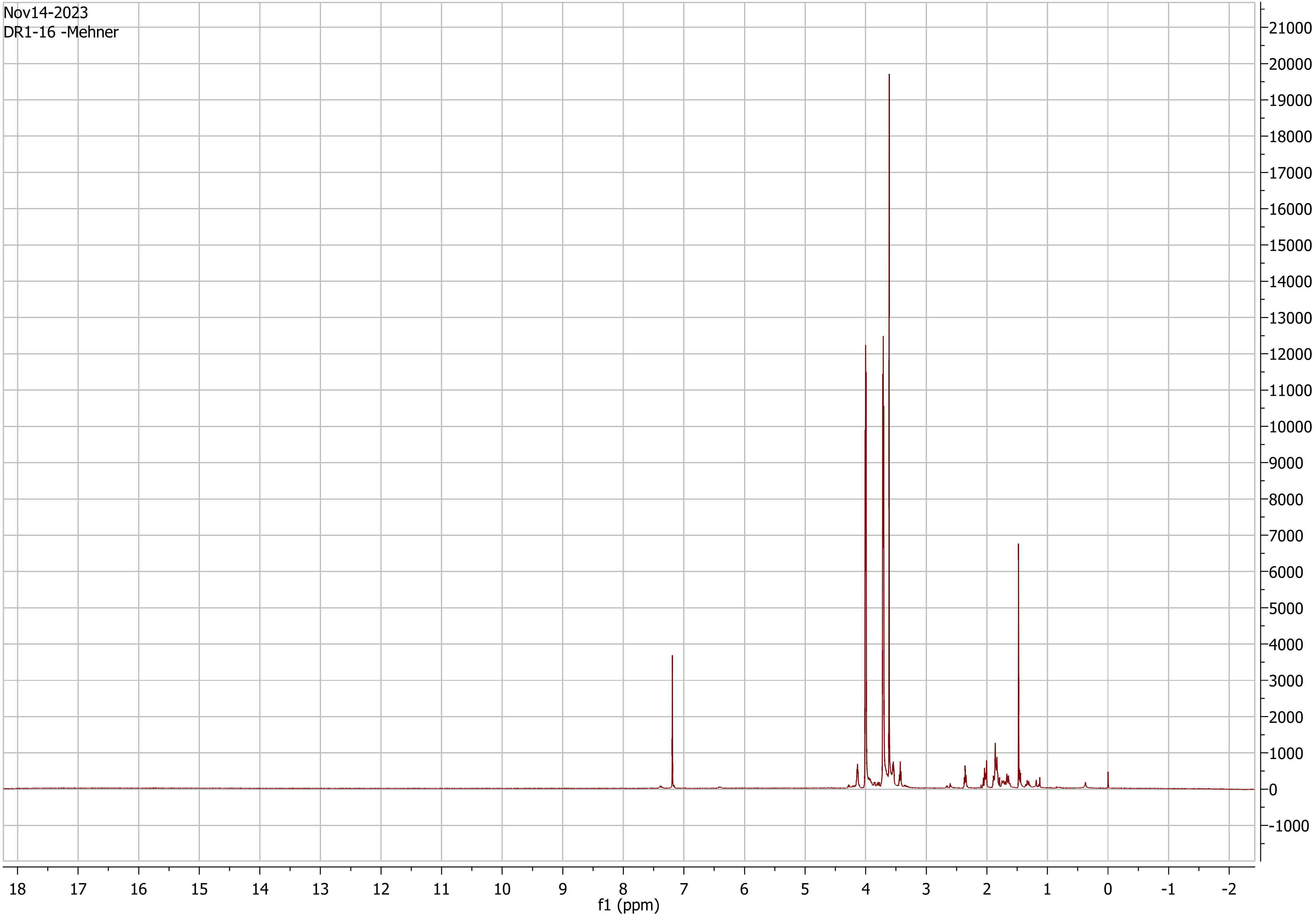

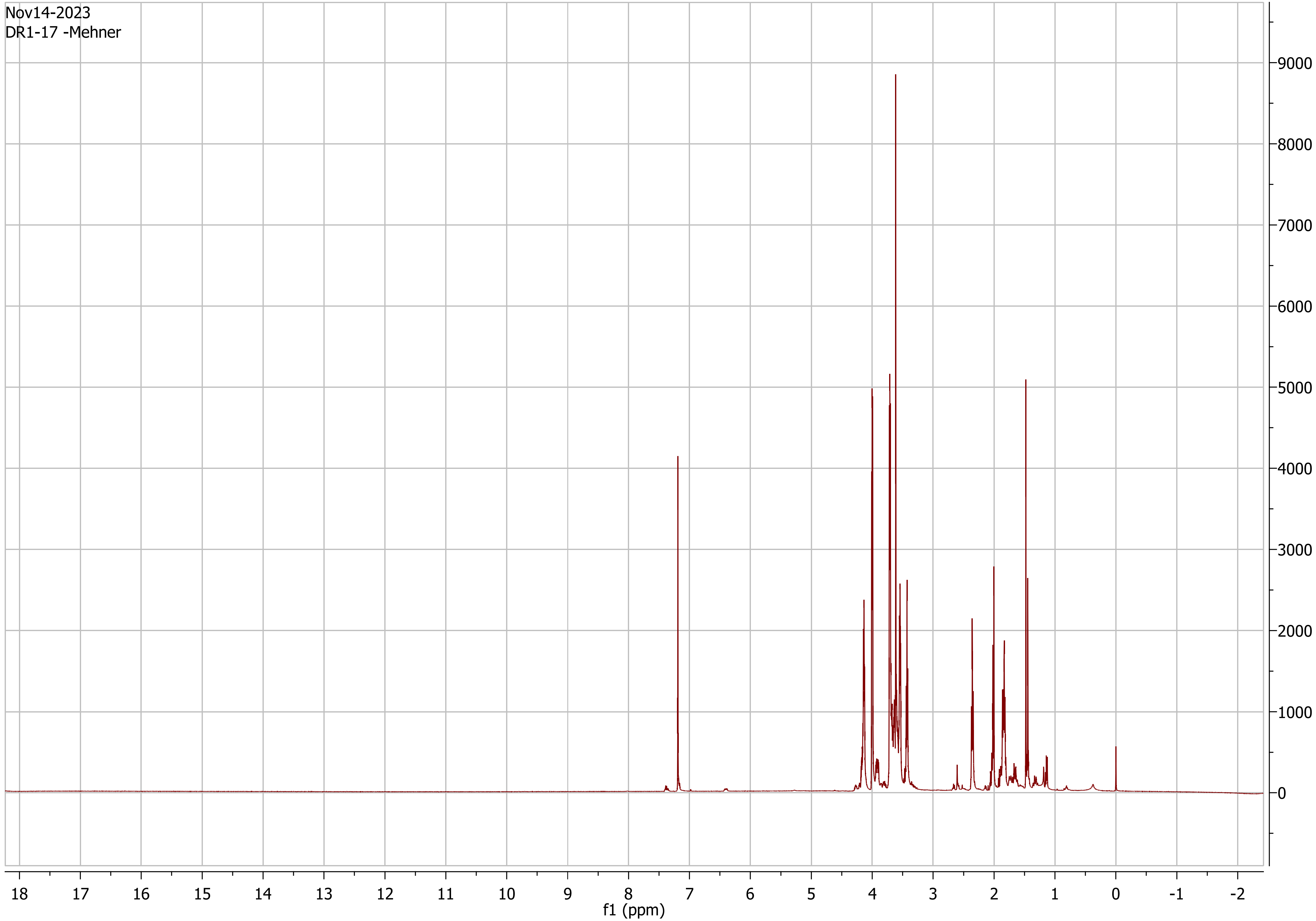

Nov14-2023
DR1-17 -Mehner
9000
8000
7000
6000
5000
4000
3000
2000
1000
0
18
17
16
15
14
13
12
11
10
9
8
7
6
5
4
3
2
1
0
-1
-2
f1 (ppm)

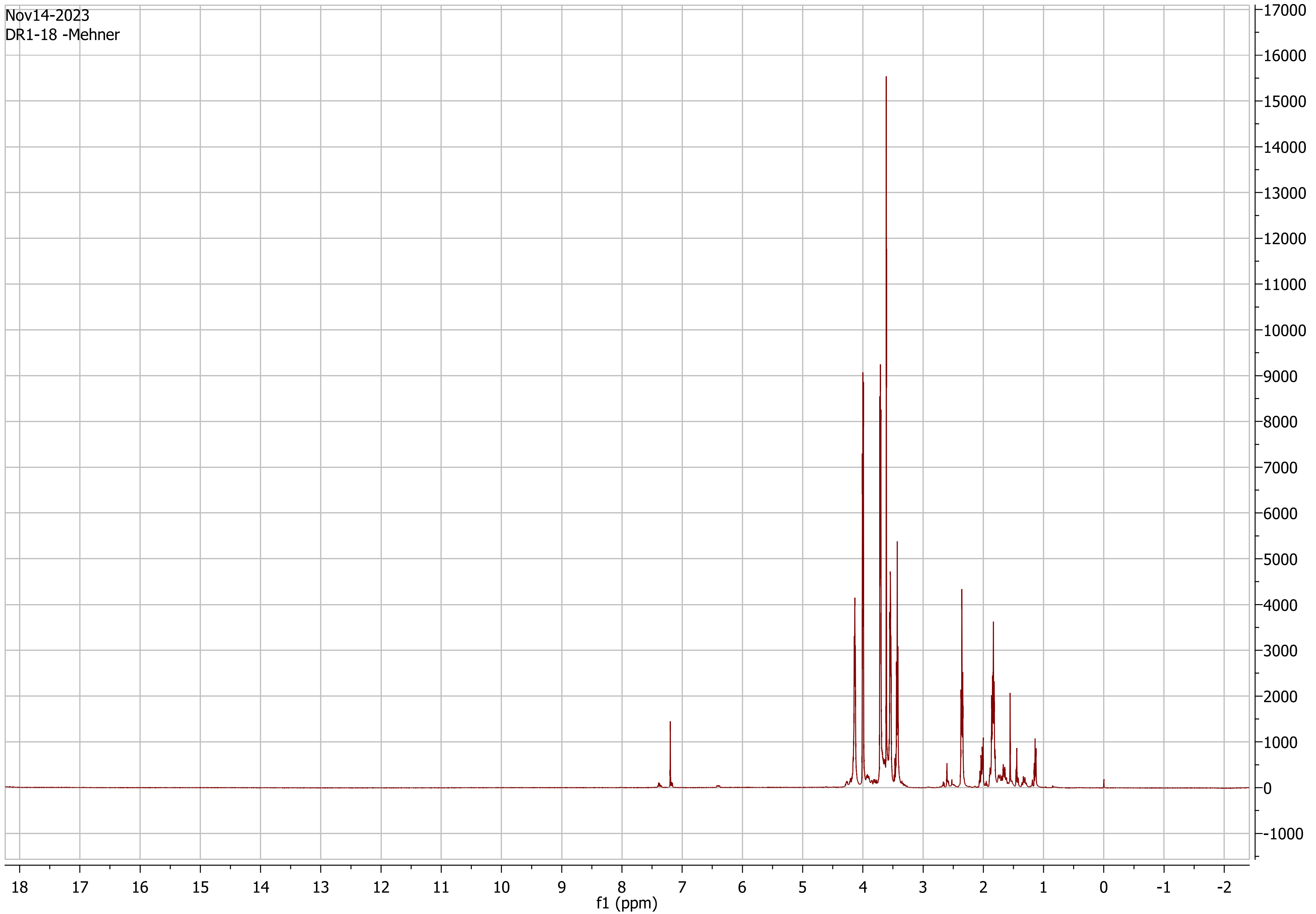
Nov14-2023
DR1-18 -Mehner
f1 (ppm)

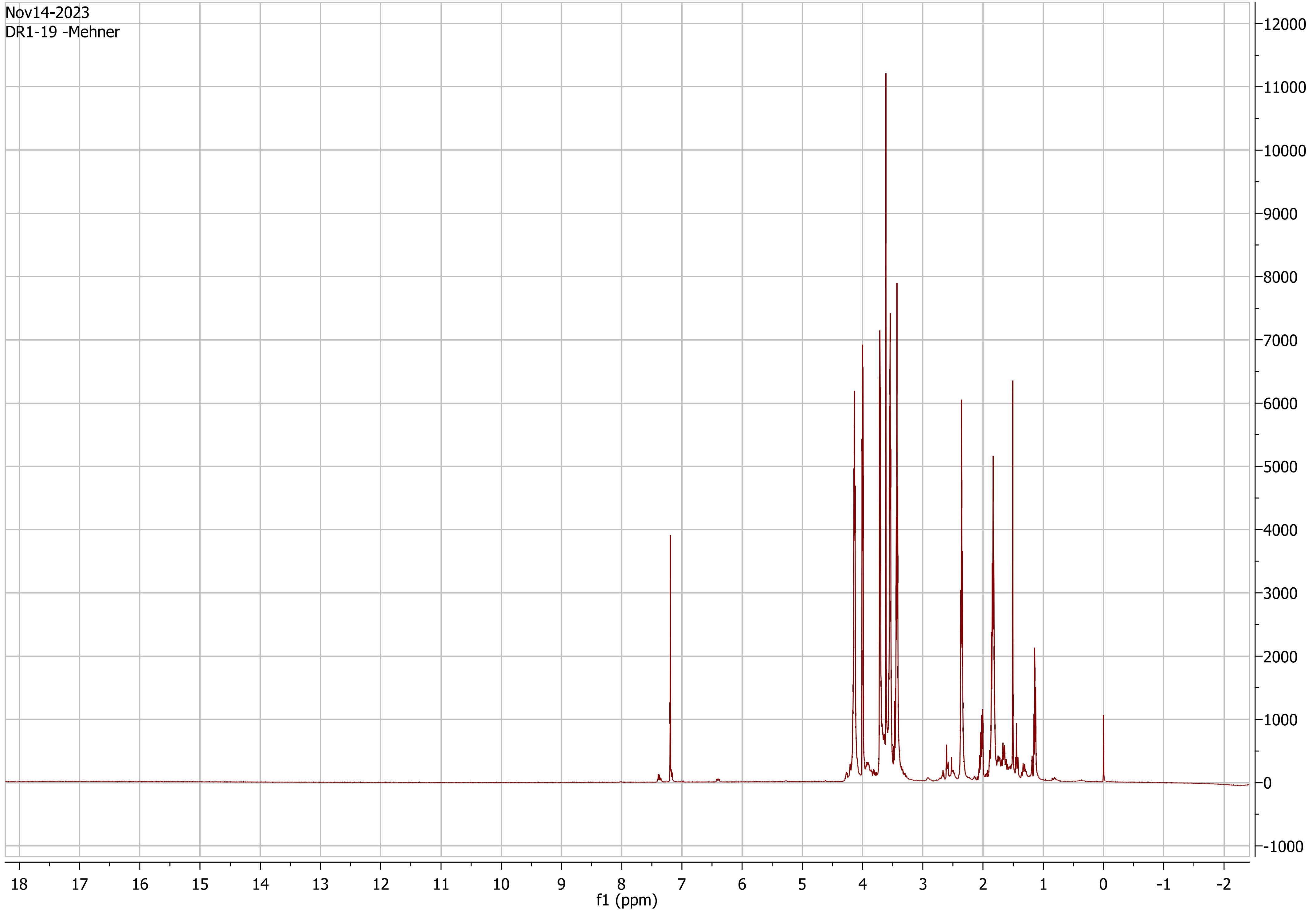

Nov14-2023
DR1-19 -Mehner
f1 (ppm)

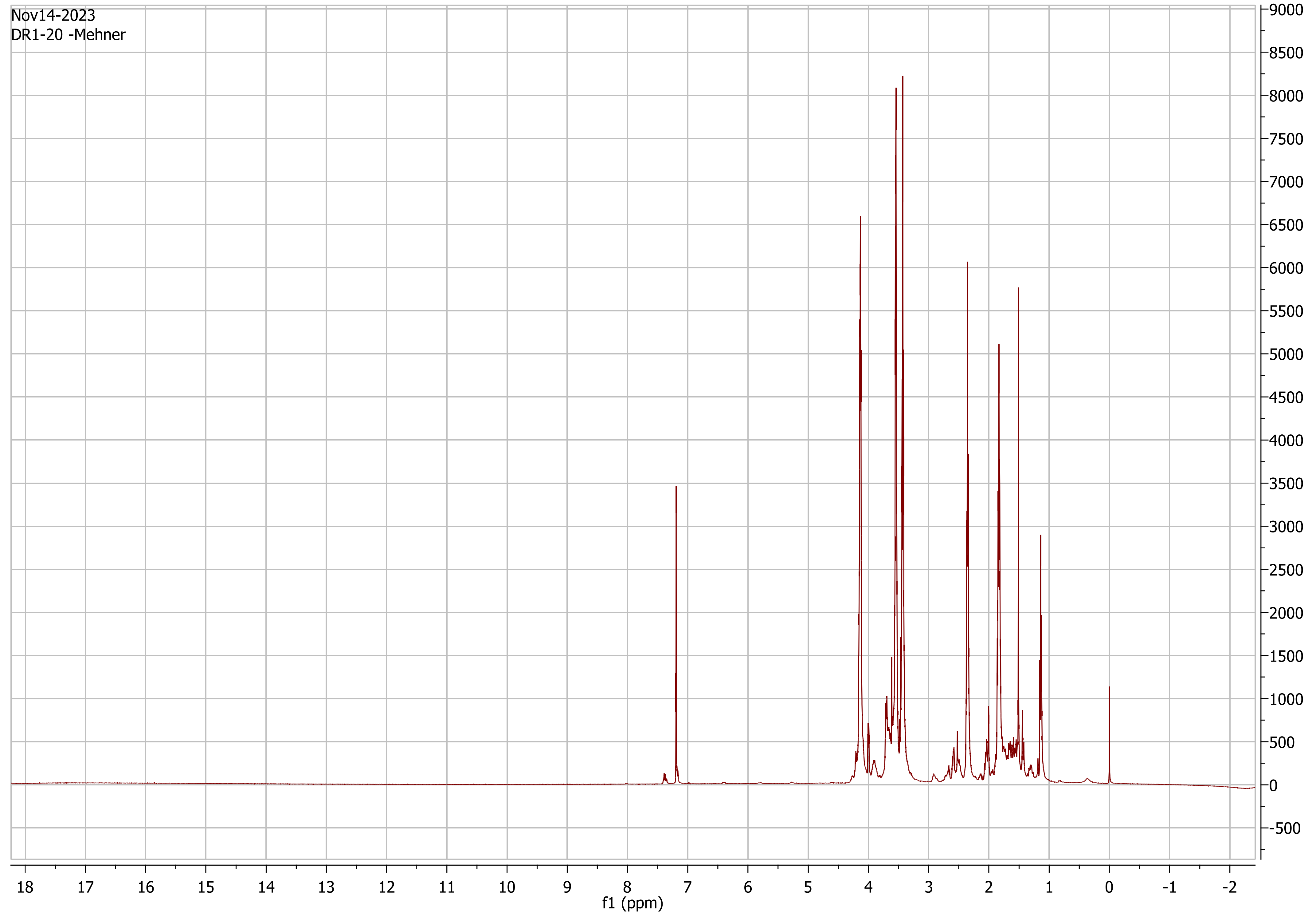

Nov14-2023
DR1-20 -Mehner
f1 (ppm)

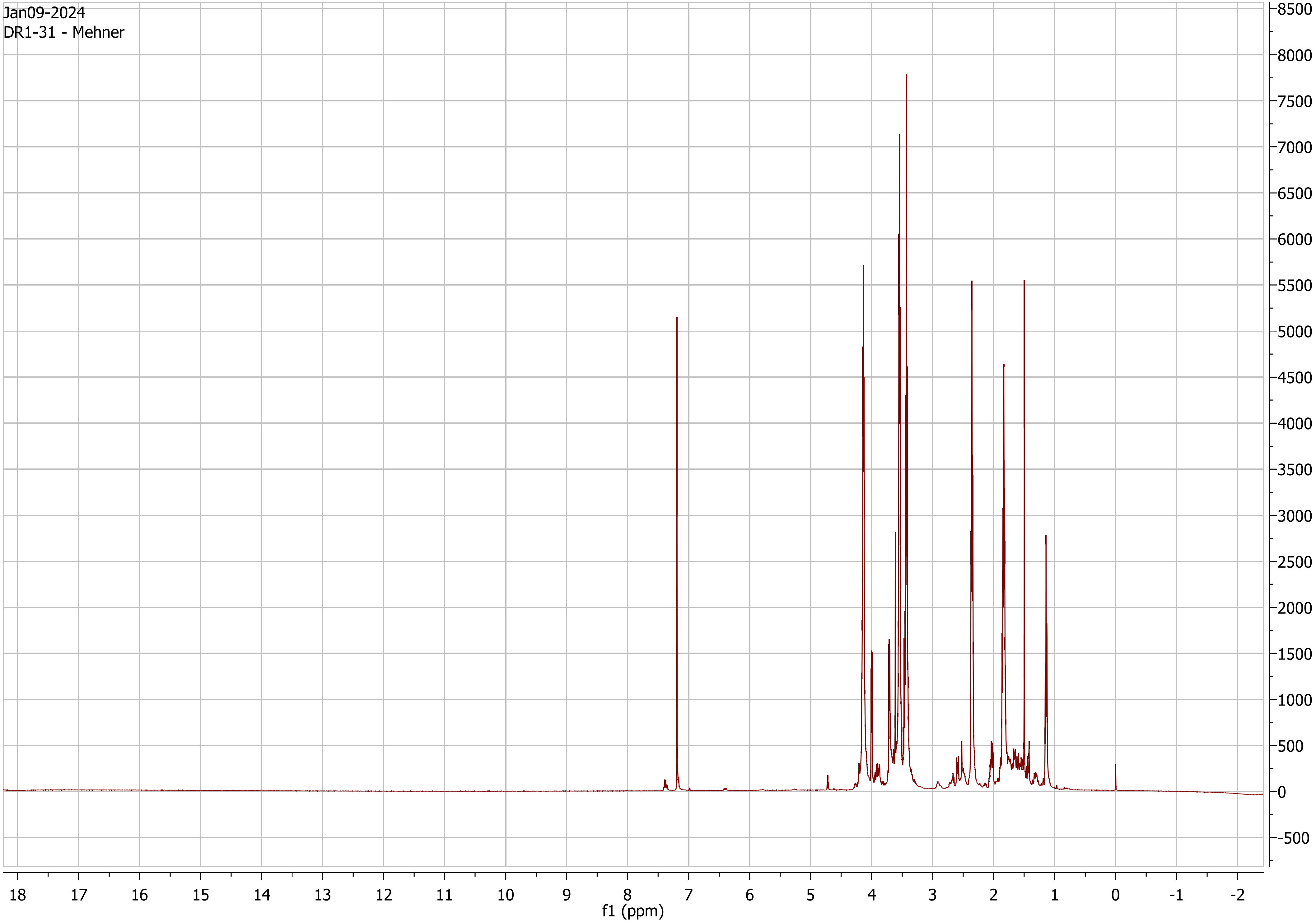

Jan09-2024
DR1-31 - Mehner
f1 (ppm)

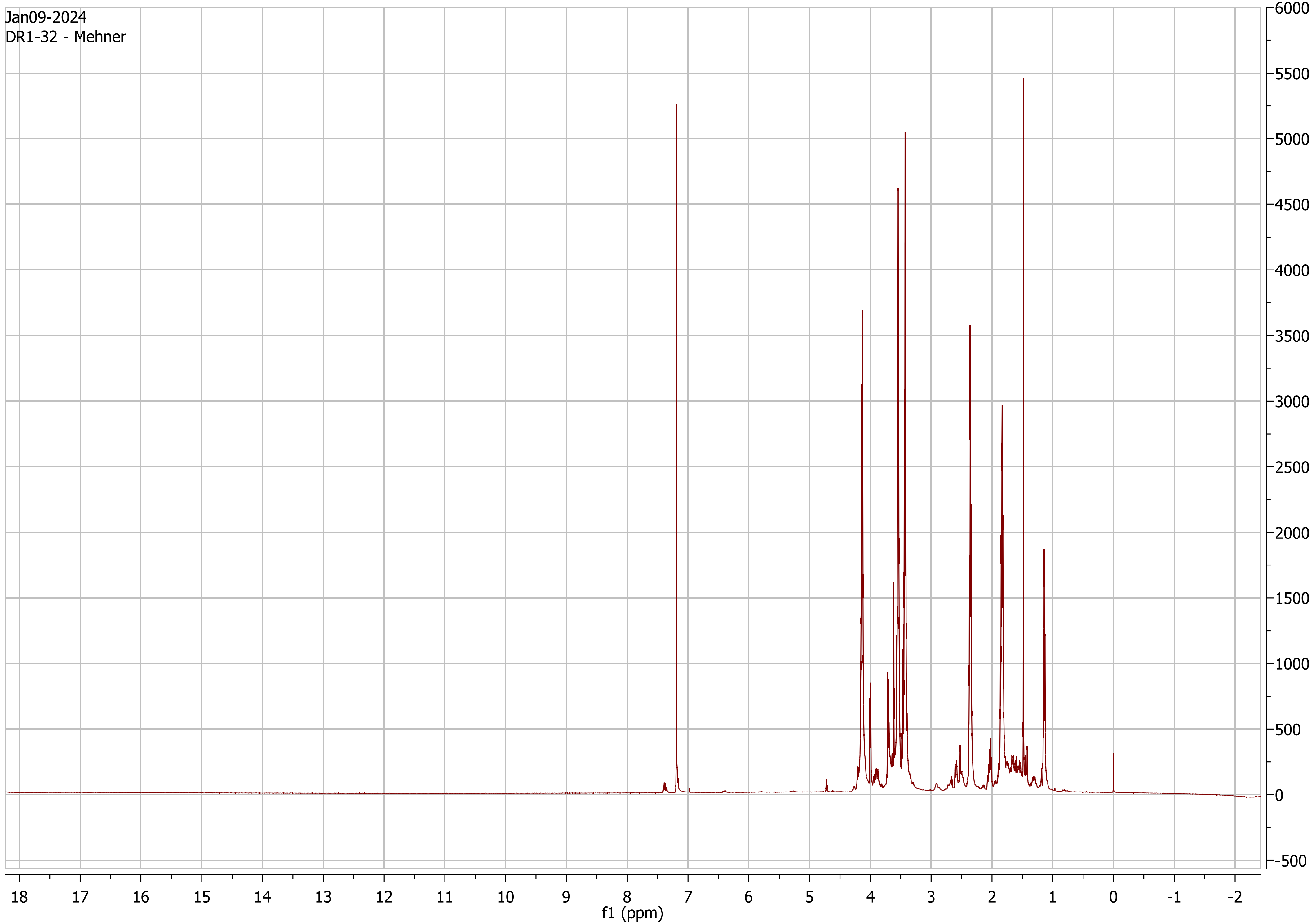
Jan09-2024
DR1-32 - Mehner
f1 (ppm)

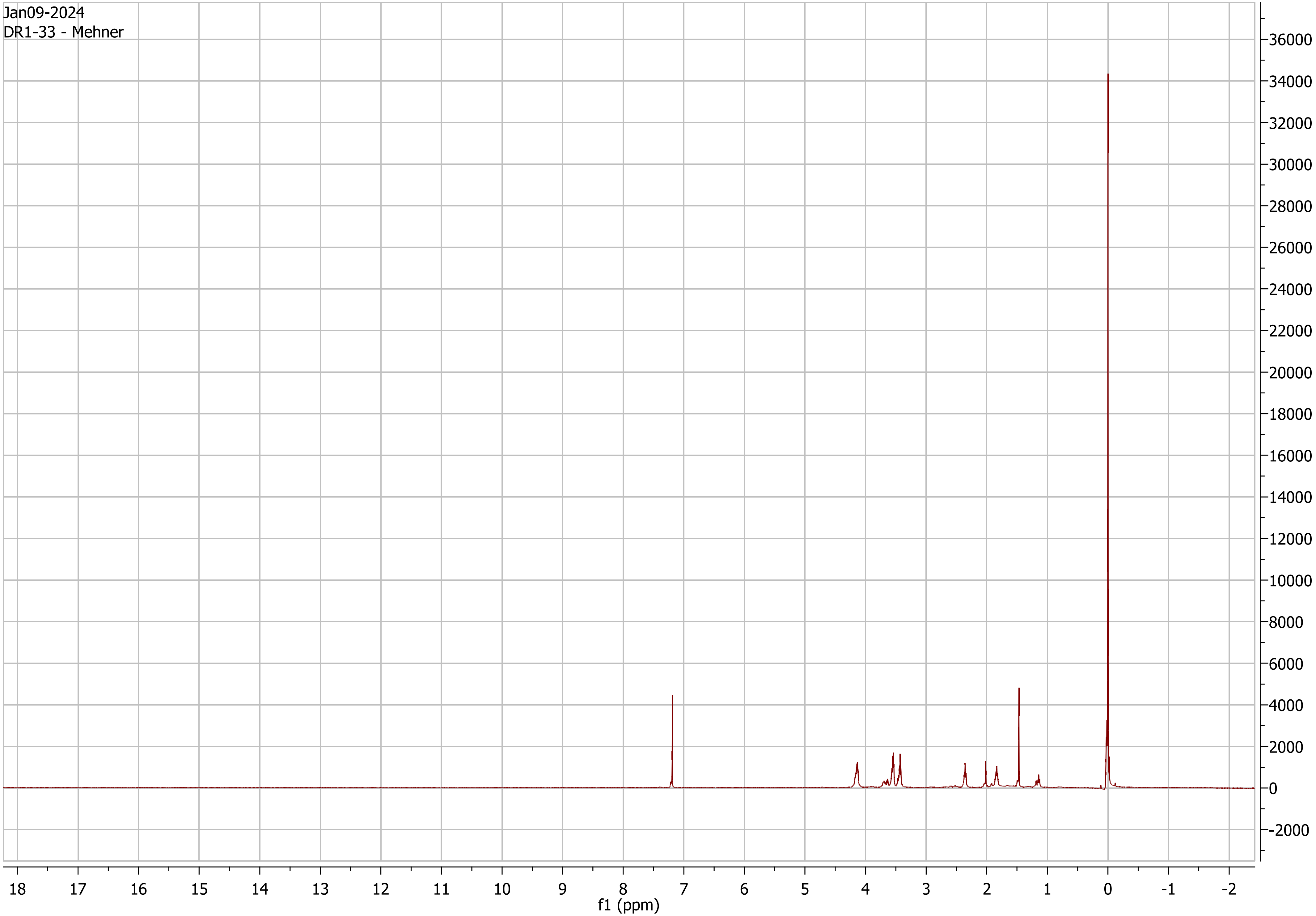

Jan09-2024
DR1-33 - Mehner
f1 (ppm)

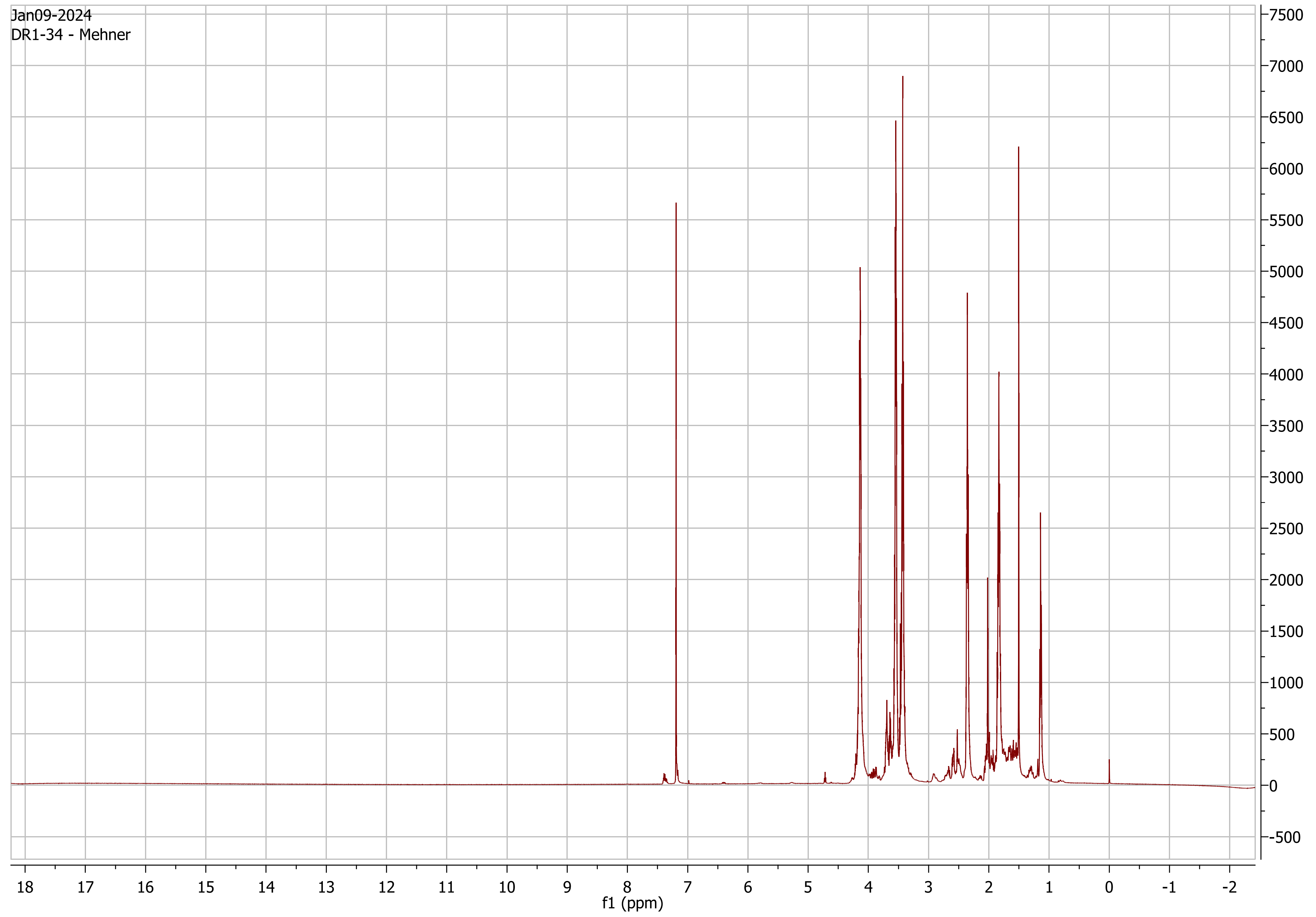

Jan09-2024
DR1-34 - Mehner
f1 (ppm)

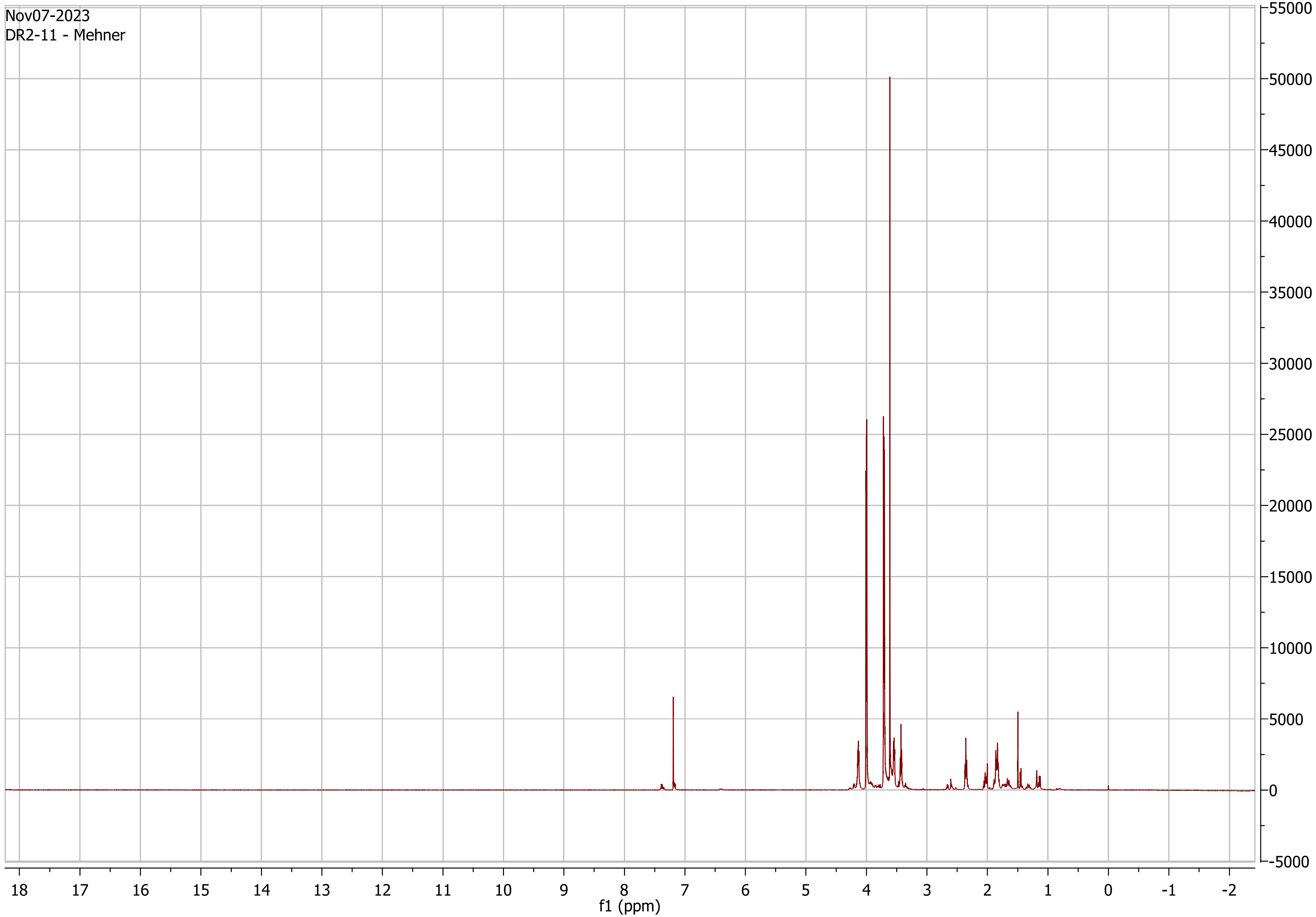

Nov07-2023
DR2-11 - Mehner
55000
50000
45000
40000
35000
30000
25000
20000
15000
10000
5000
0
-5000
18 17 16 15 14 13 12 11 10 9 8 7 6 5 4 3 2 1 0 -1 -2
f1 (ppm)

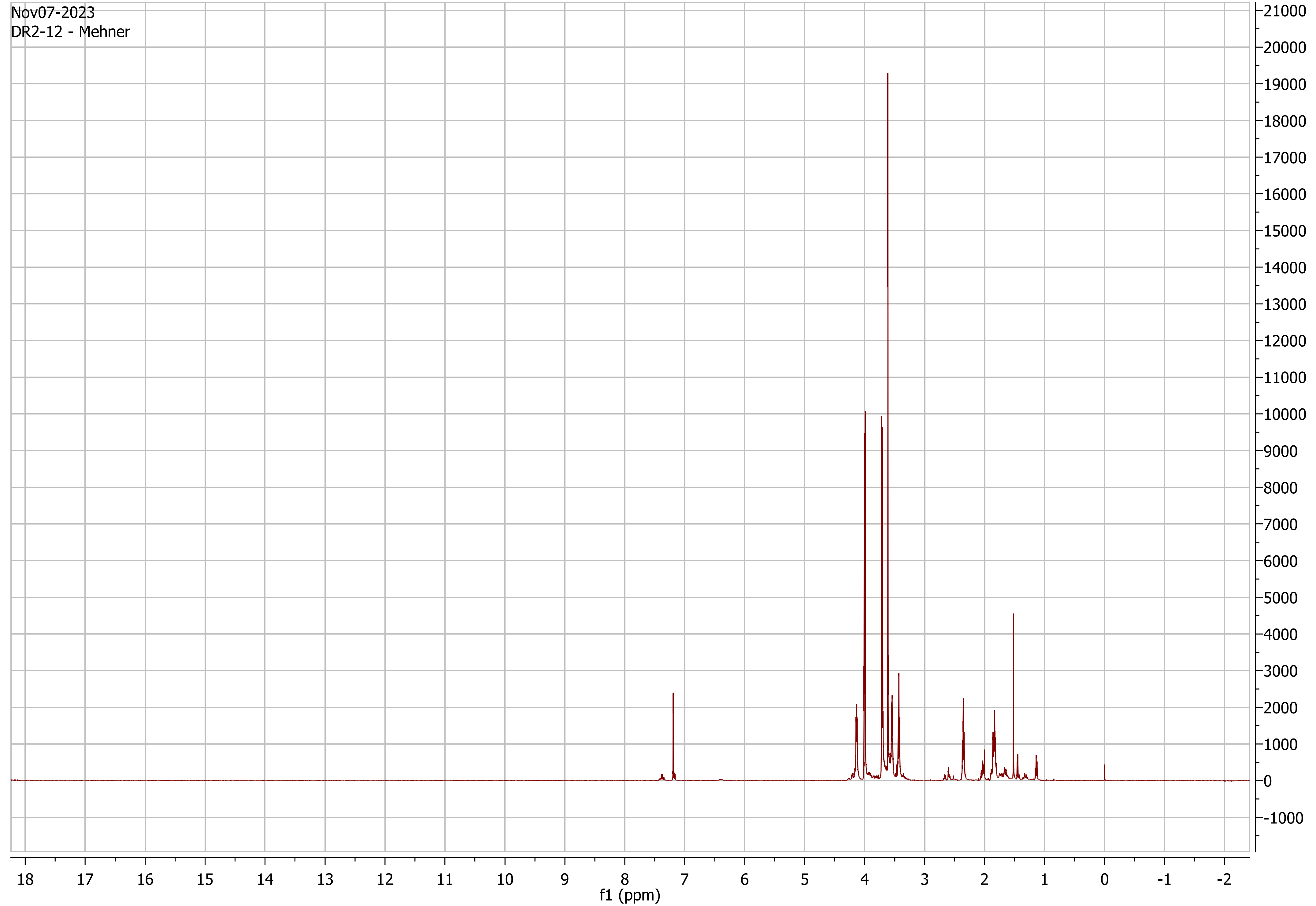

Nov07-2023
DR2-12 - Mehner
f1 (ppm)

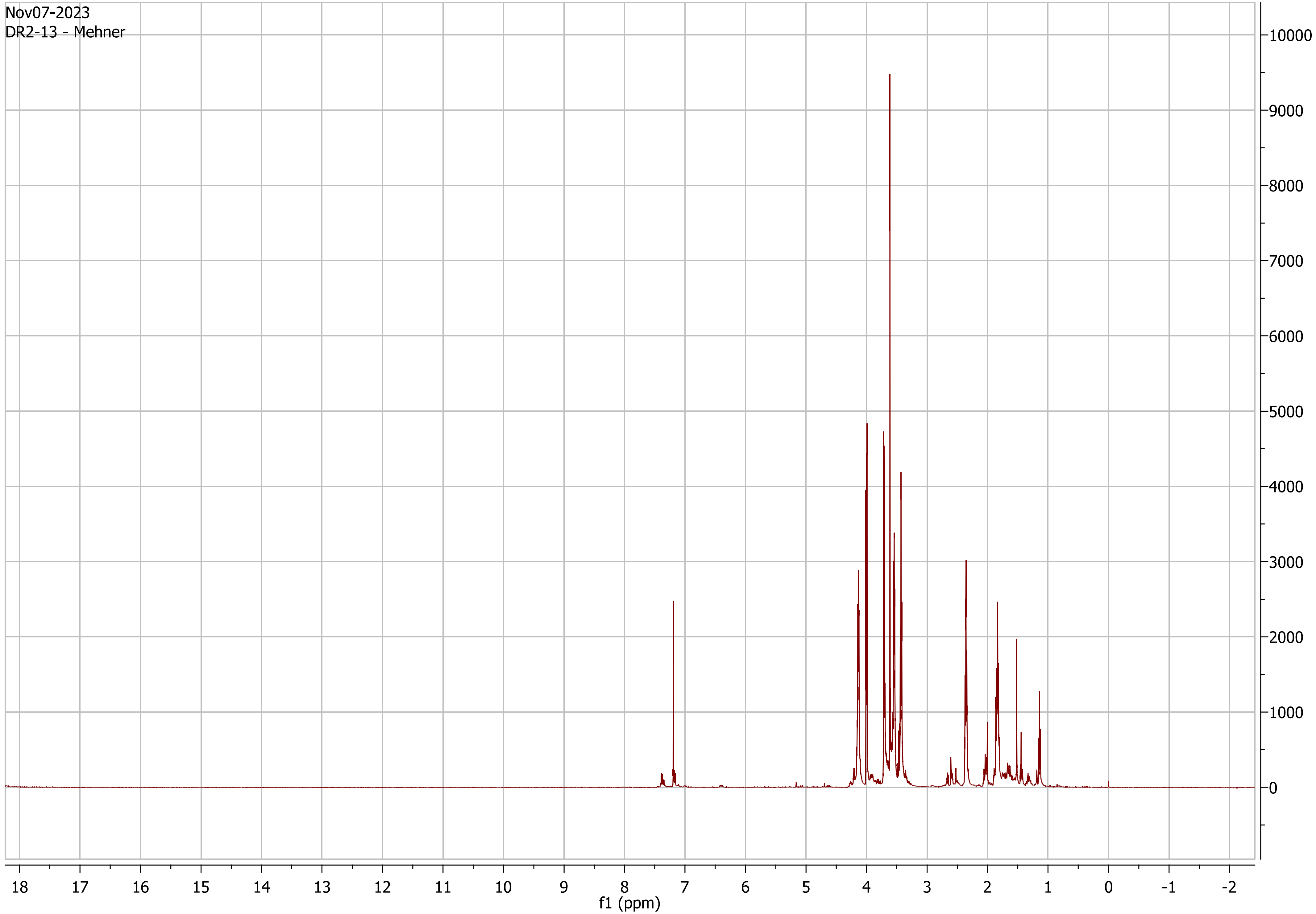
Nov07-2023
DR2-13 - Mehner
f1 (ppm)

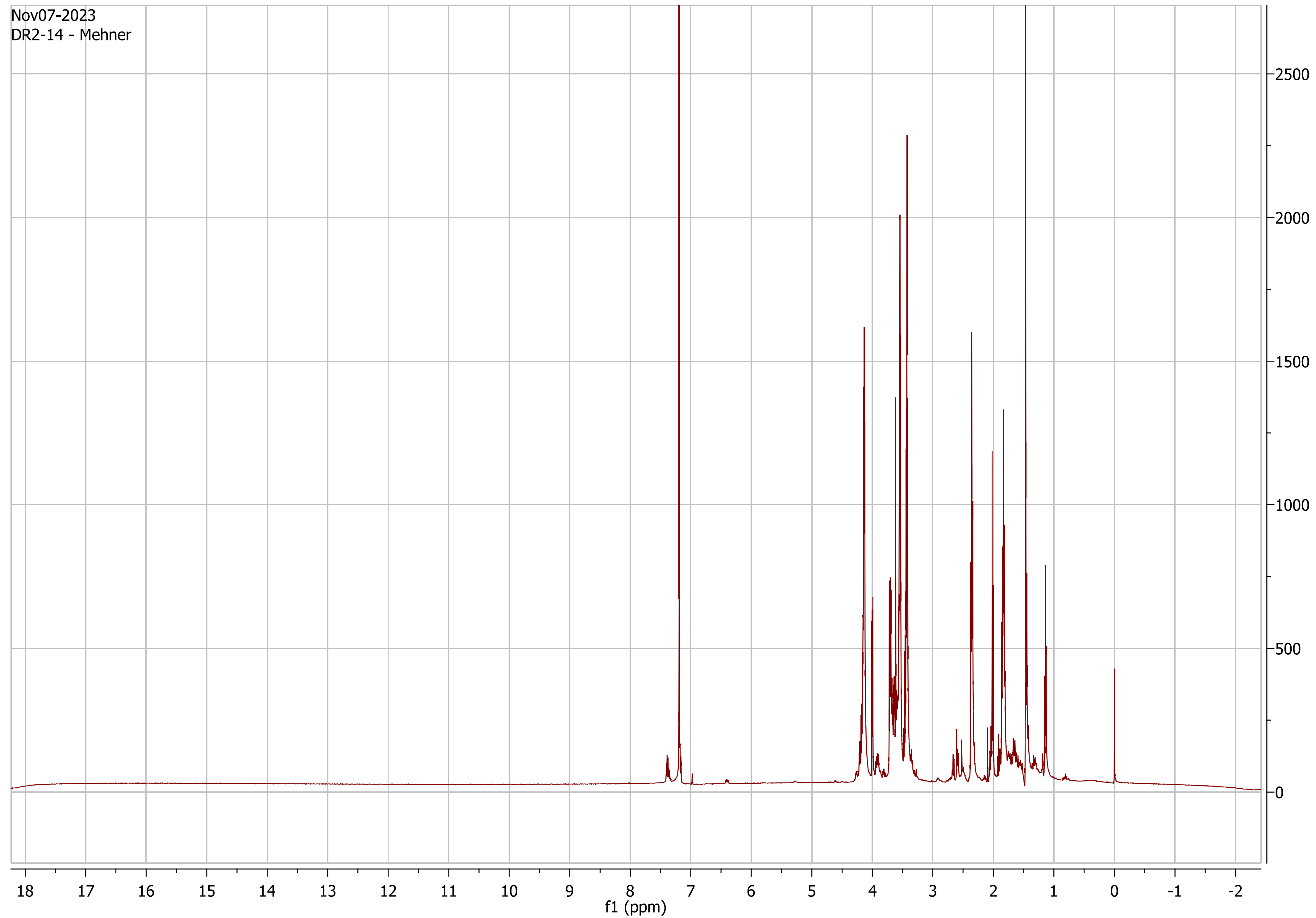

Nov07-2023
DR2-14 - Mehner
2500
2000
1500
1000
500
0
18
17
16
15
14
13
12
11
10
9
8
7
6
5
4
3
2
1
0
-1
-2
f1 (ppm)

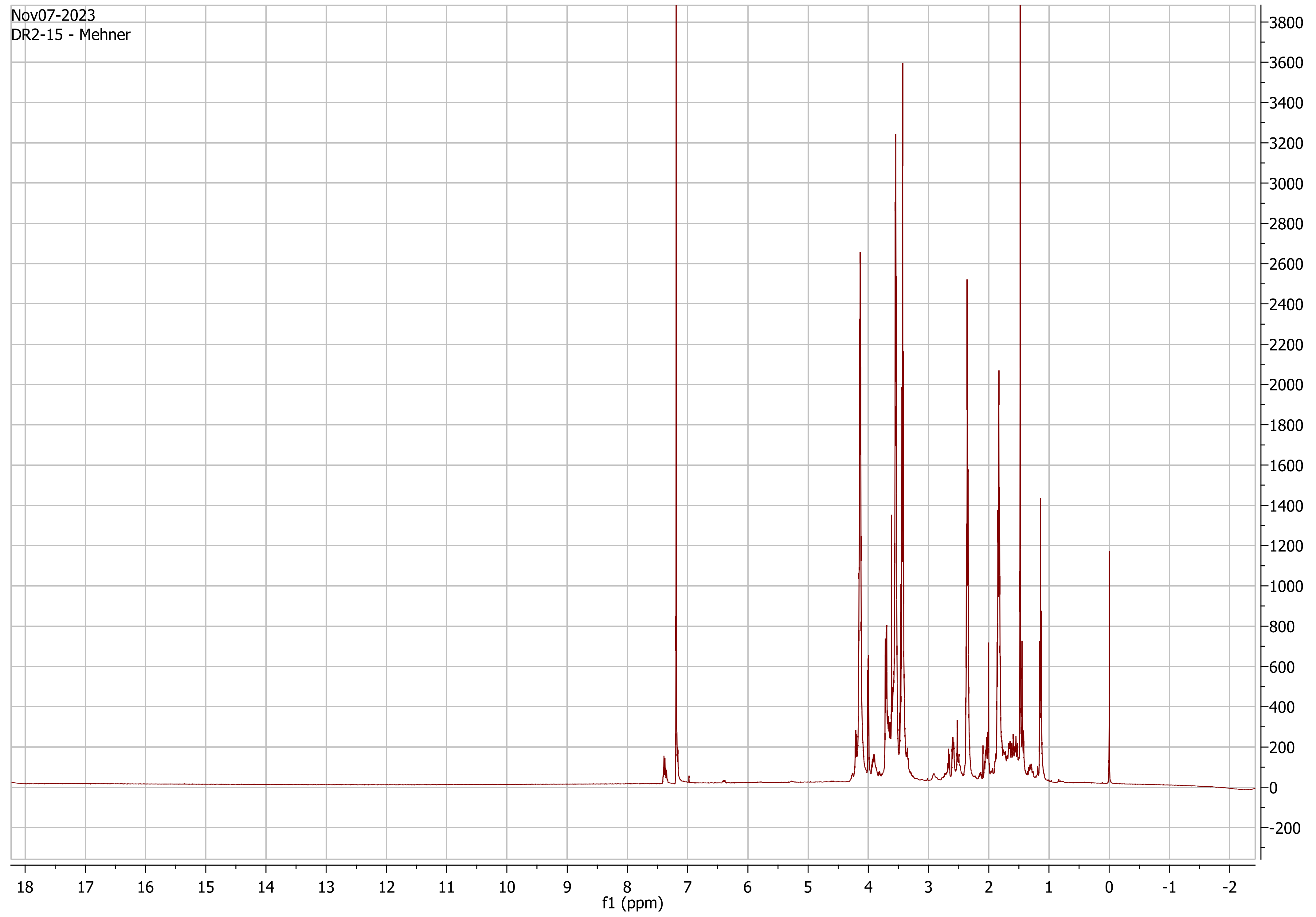

Nov07-2023
DR2-15 - Mehner
f1 (ppm)

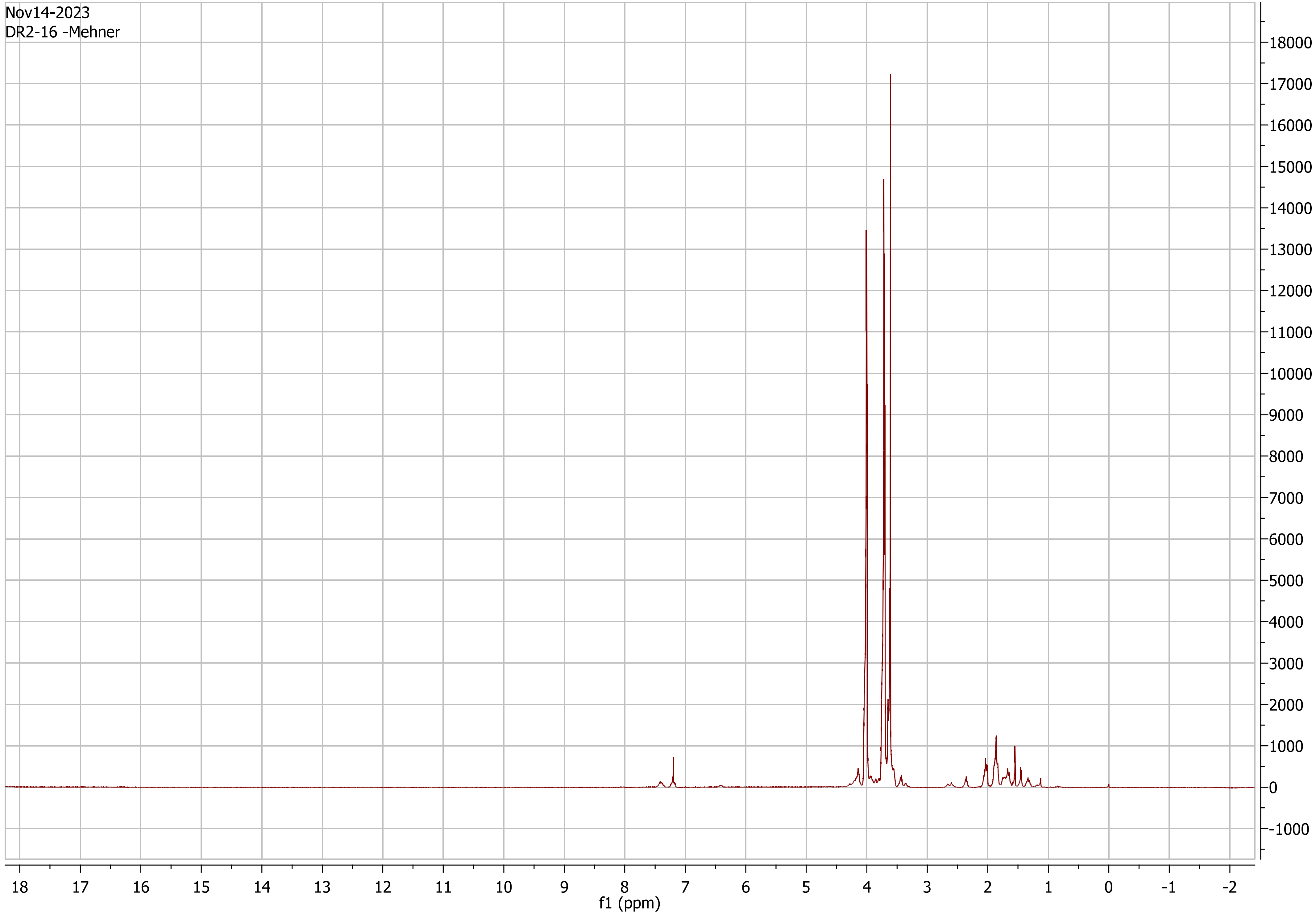

Nov14-2023
DR2-16 -Mehner
f1 (ppm)

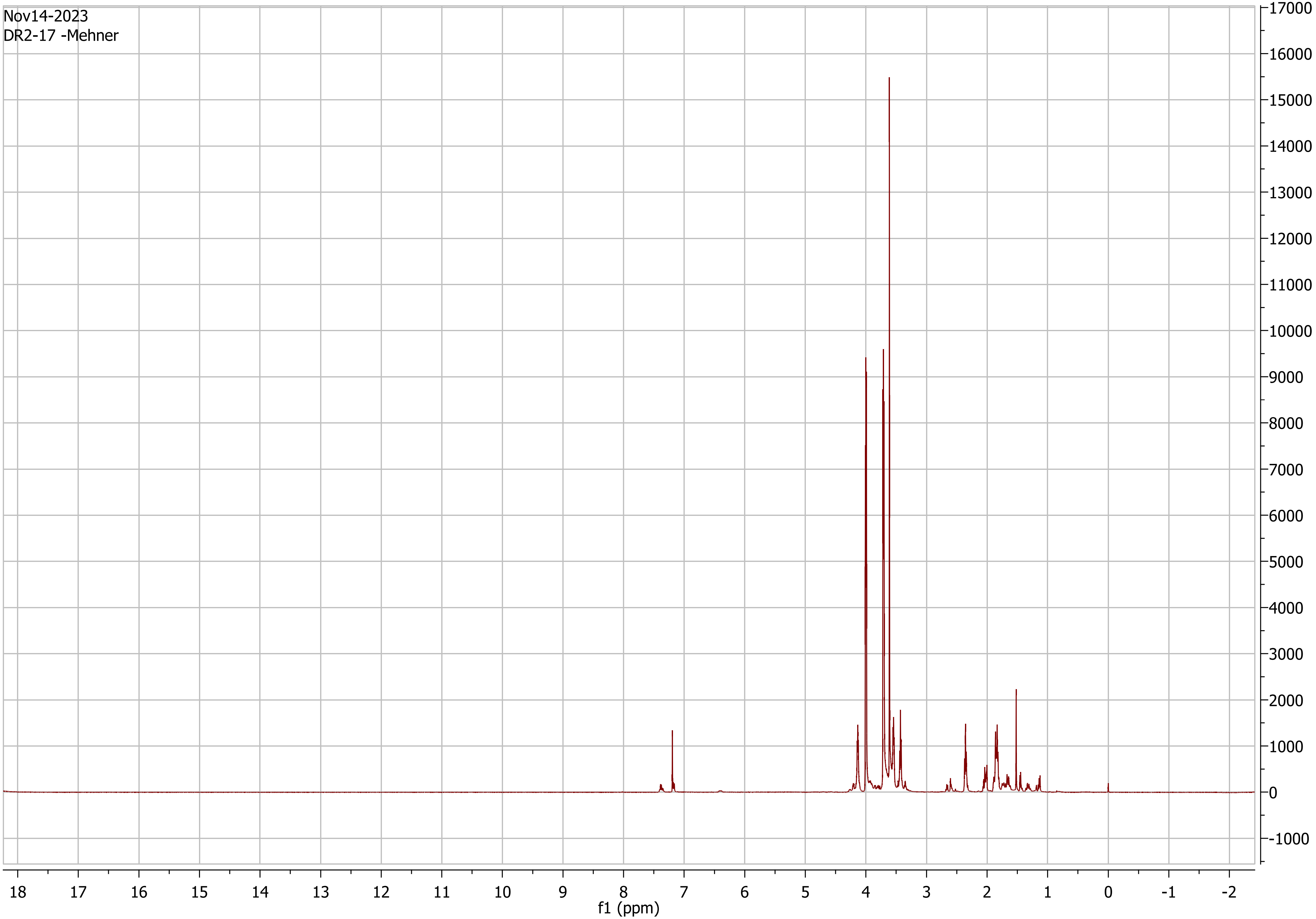
Nov14-2023
DR2-17 -Mehner
f1 (ppm)
18
17
16
15
14
13
12
11
10
9
8
7
6
5
4
3
2
1
0
-1
-2
17000
16000
15000
14000
13000
12000
11000
10000
9000
8000
7000
6000
5000
4000
3000
2000
1000
0
-1000

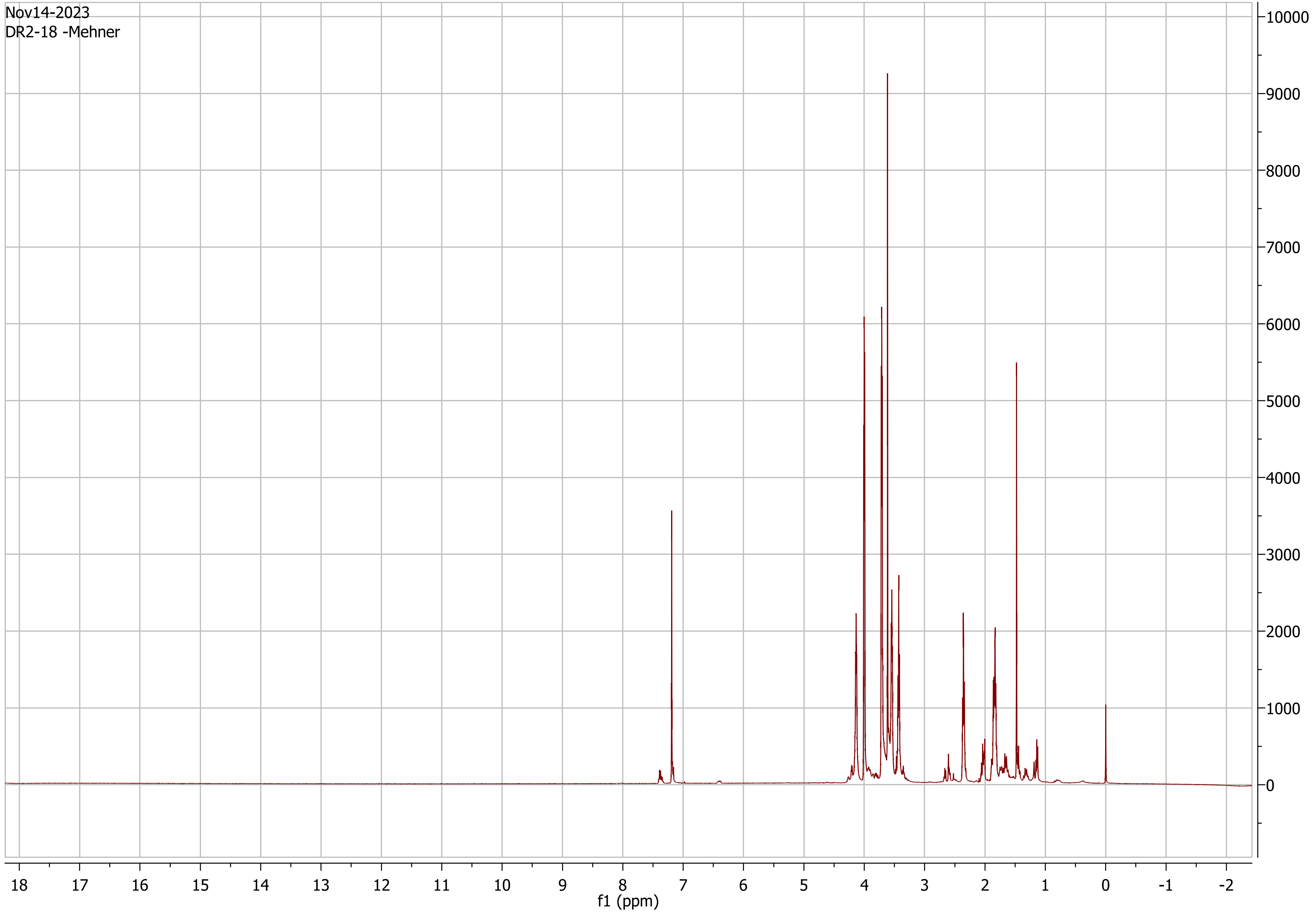

Nov14-2023
DR2-18 -Mehner
f1 (ppm)

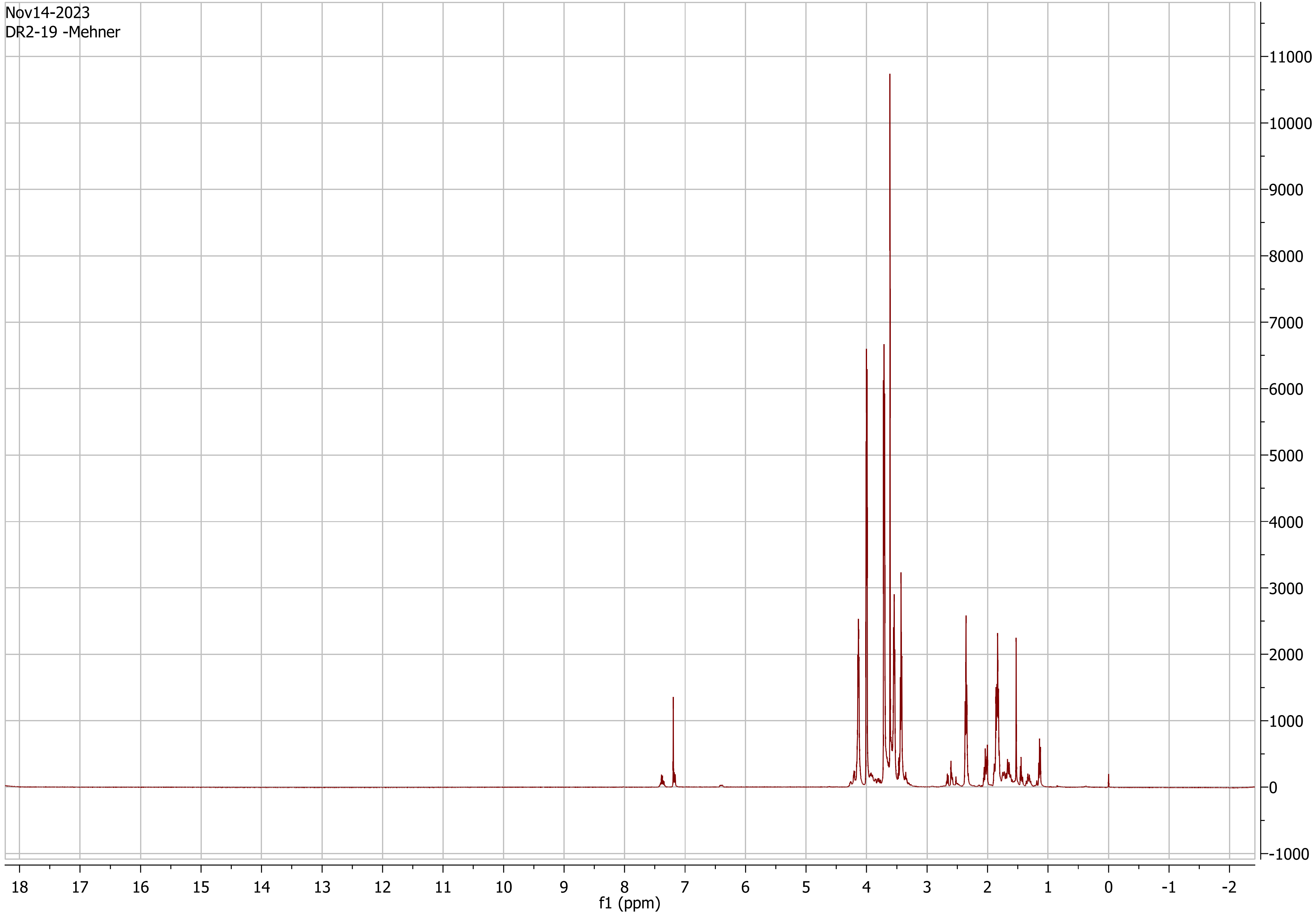

Nov14-2023
DR2-19 -Mehner
f1 (ppm)

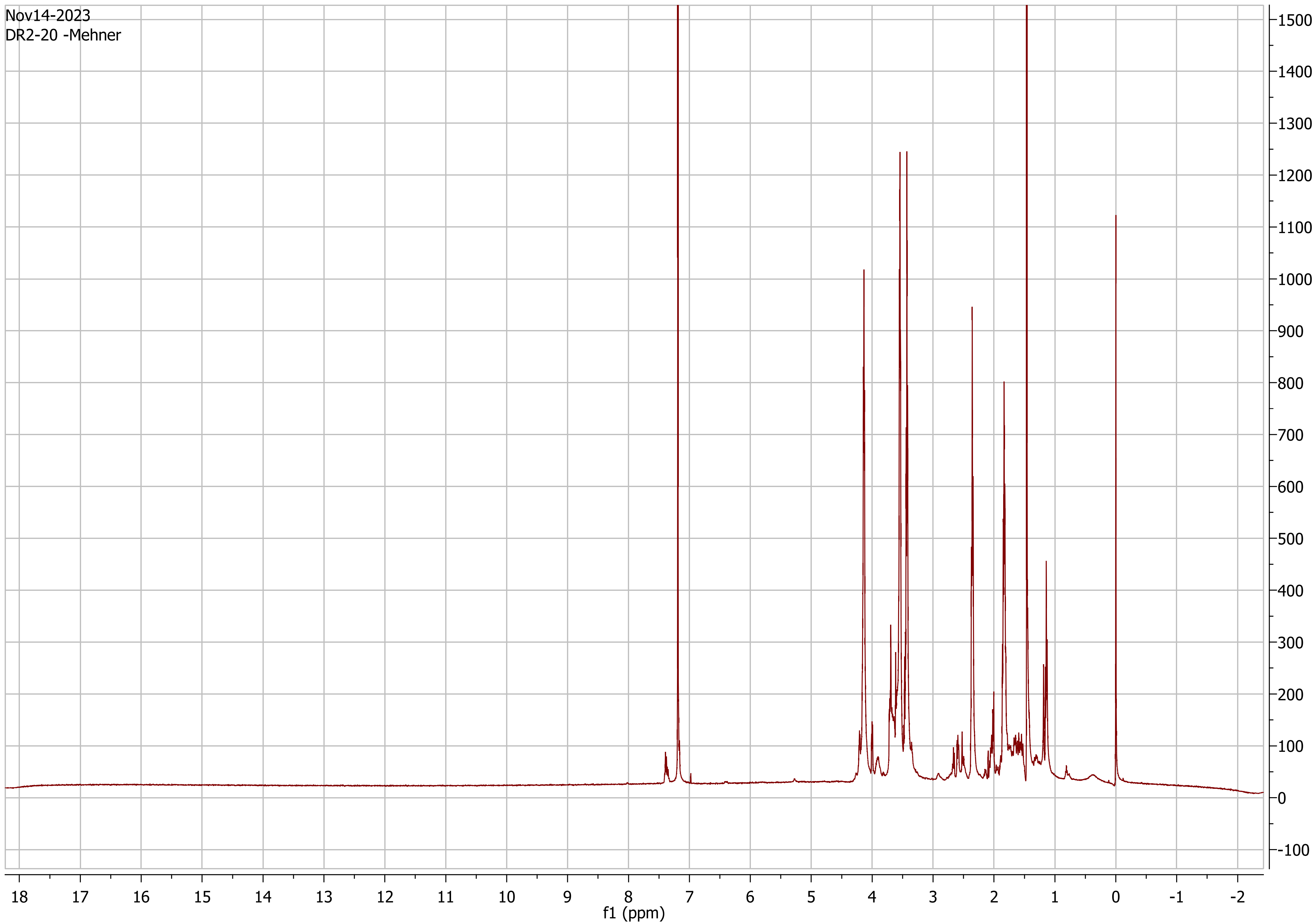

Nov14-2023
DR2-20 -Mehner
f1 (ppm)

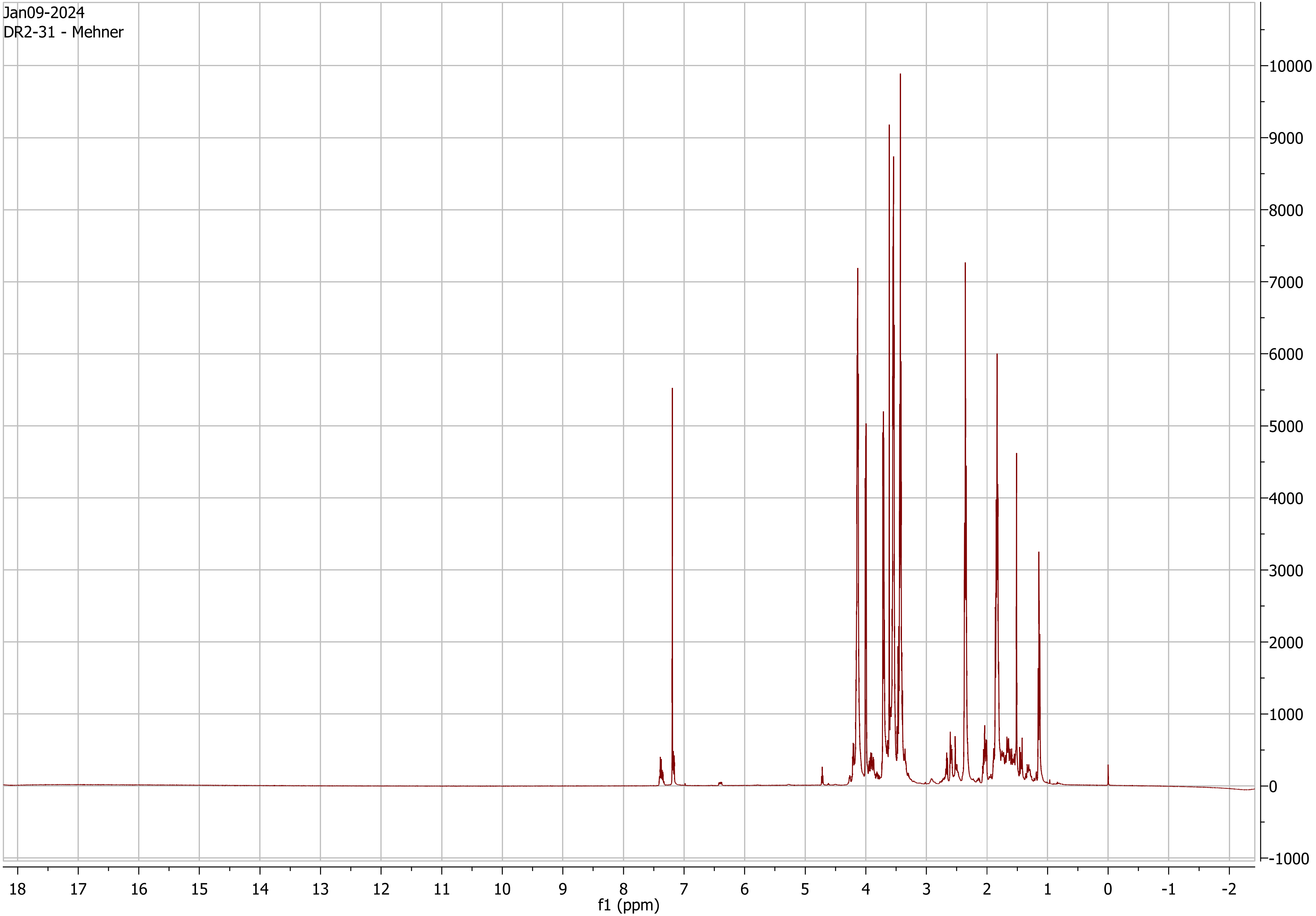

Jan09-2024
DR2-31 - Mehner
10000
9000
8000
7000
6000
5000
4000
3000
2000
1000
0
-1000
18
17
16
15
14
13
12
11
10
9
8
7
6
5
4
3
2
1
0
-1
-2
f1 (ppm)

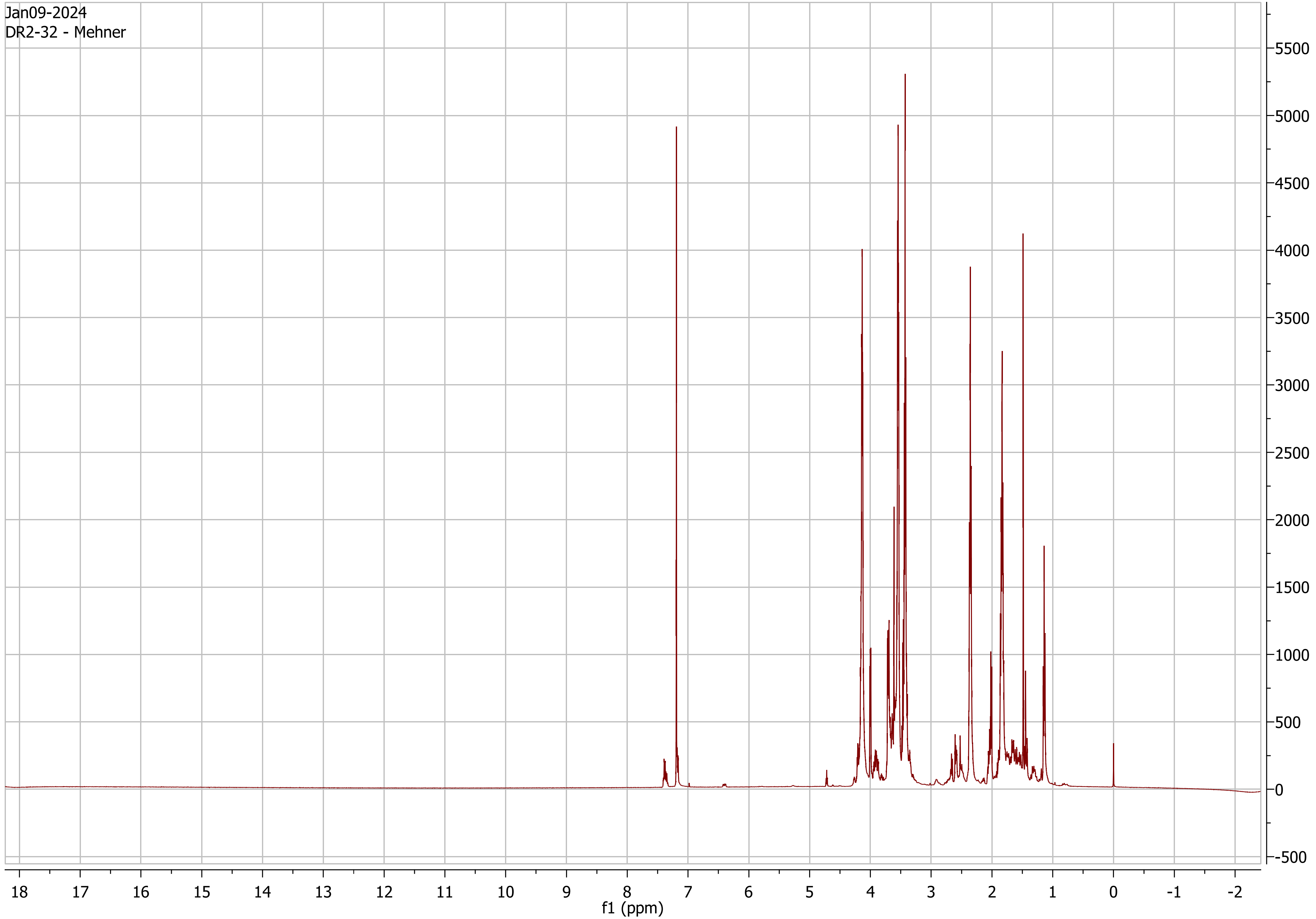

Jan09-2024
DR2-32 - Mehner
f1 (ppm)

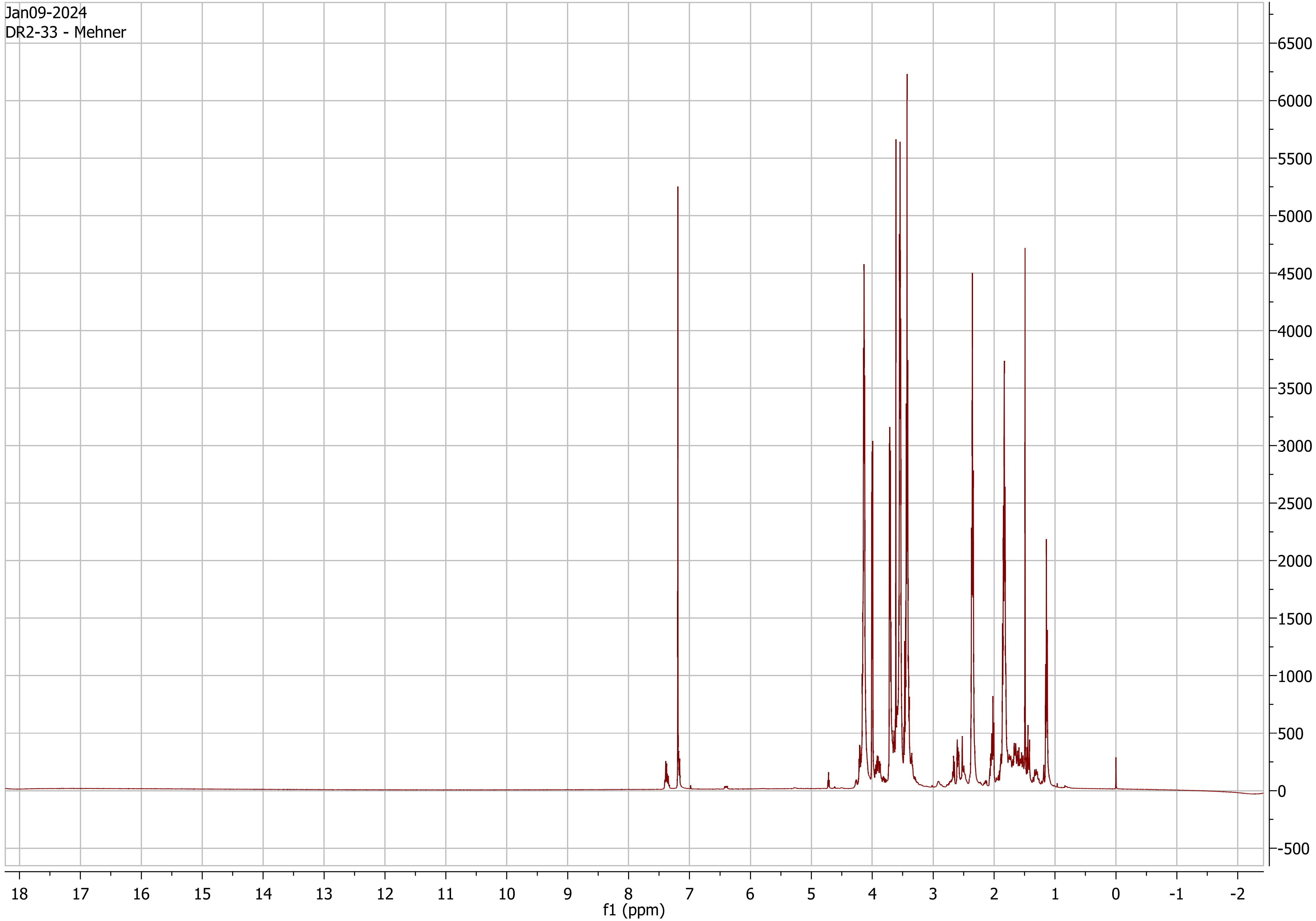

Jan09-2024
DR2-33 - Mehner
f1 (ppm)

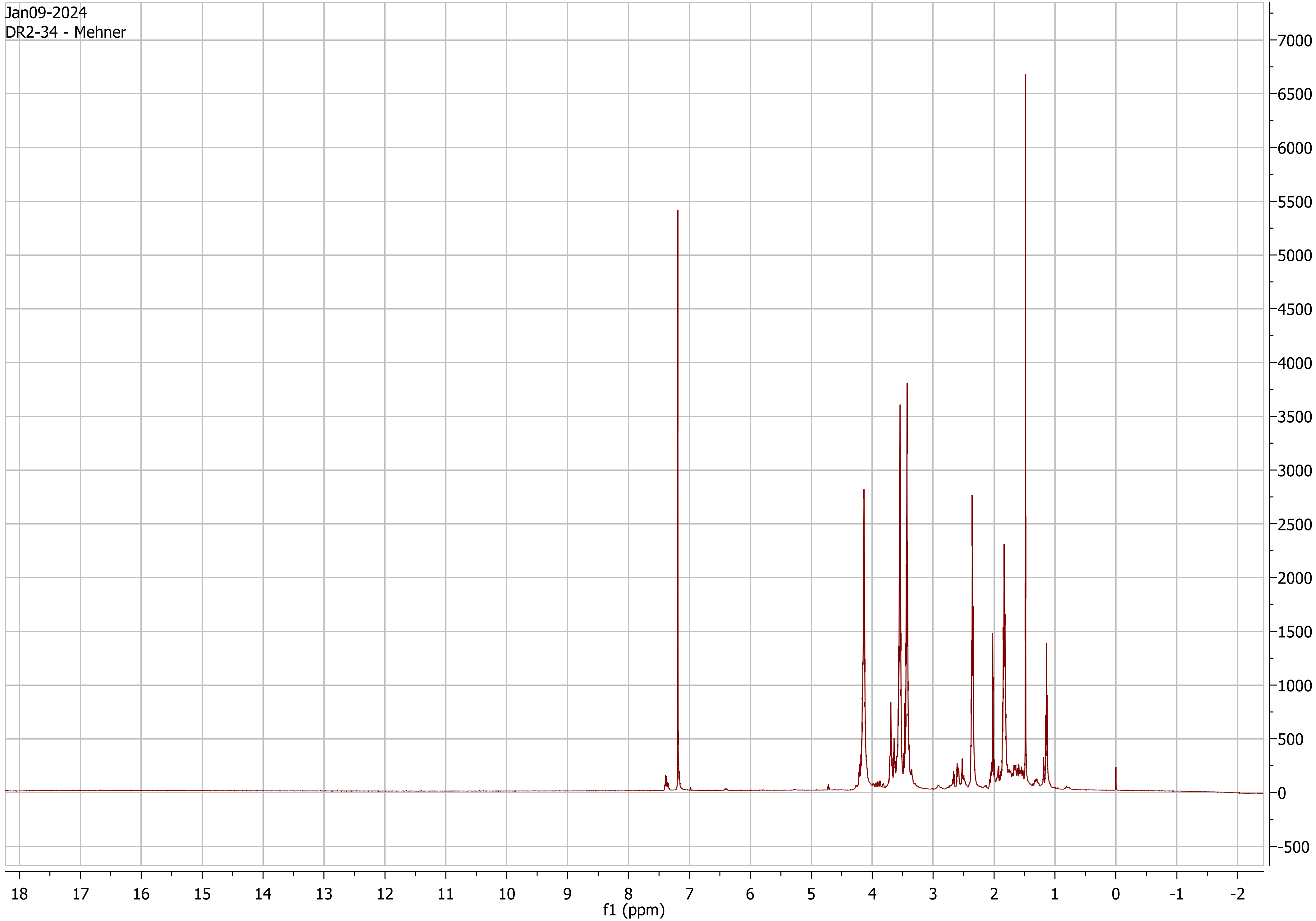

Jan09-2024
DR2-34 - Mehner
f1 (ppm)

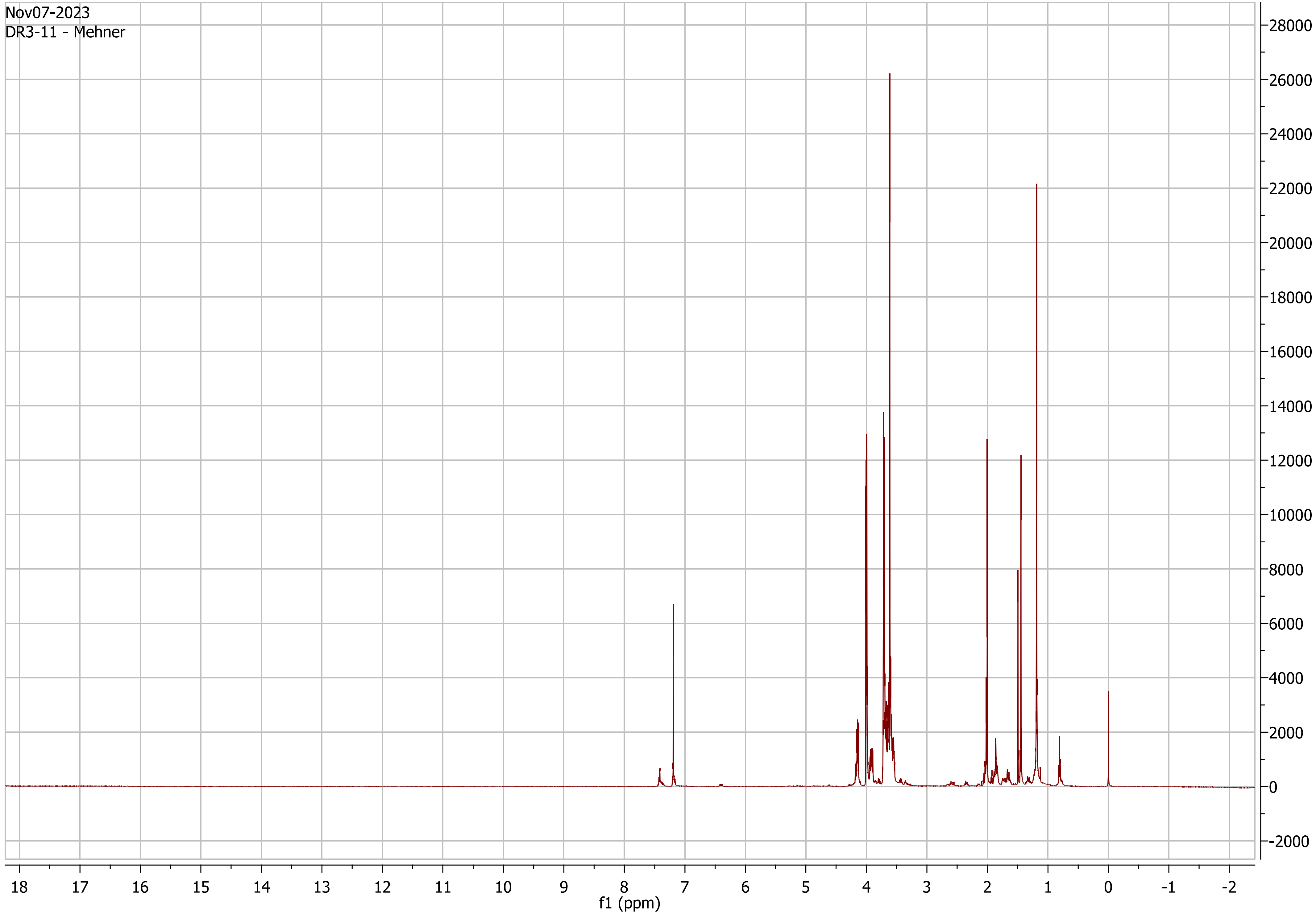
Nov07-2023
DR3-11 - Mehner
f1 (ppm)

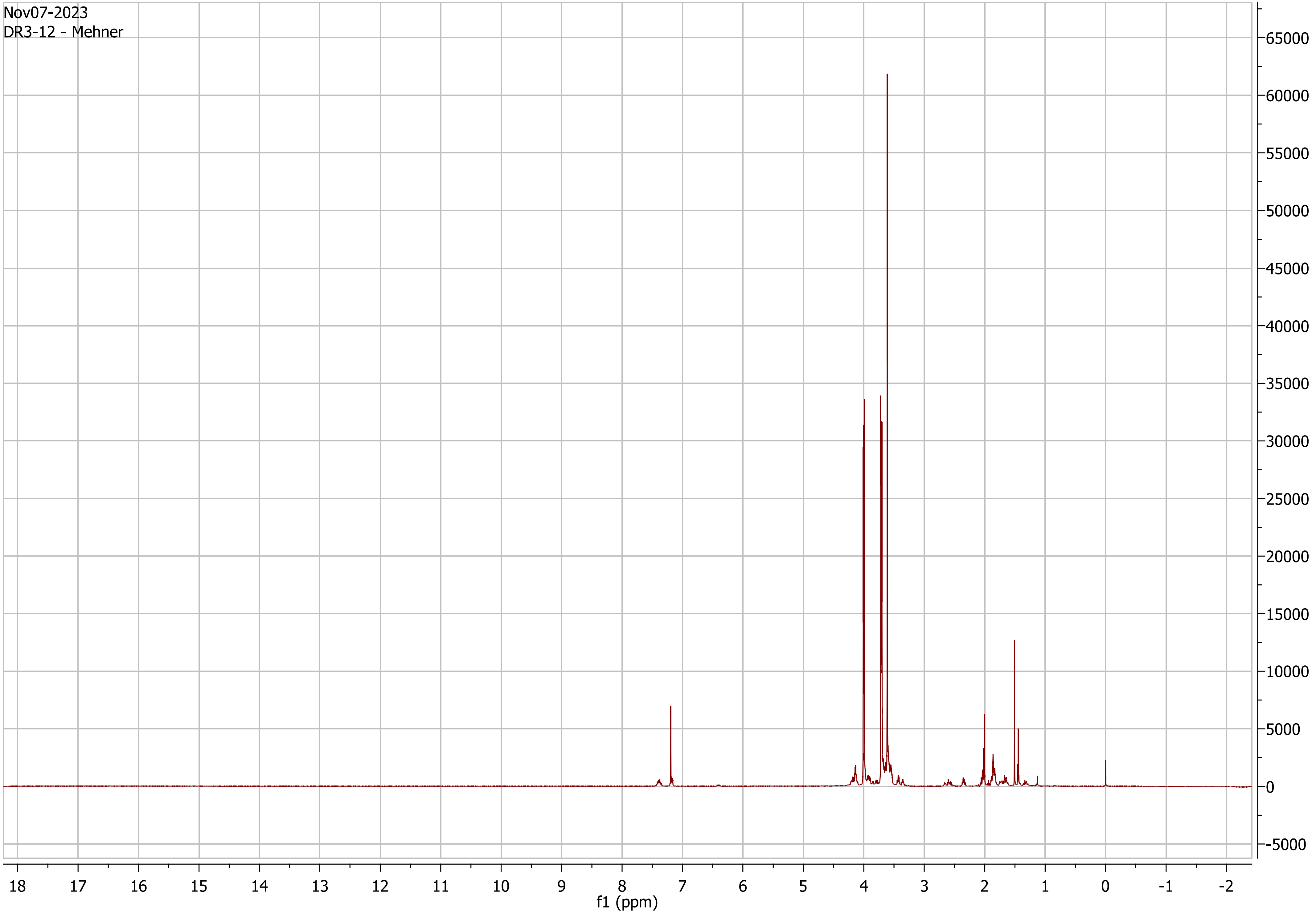
Nov07-2023
DR3-12 - Mehner
f1 (ppm)

Nov07-2023
DR3-13 - Mehner

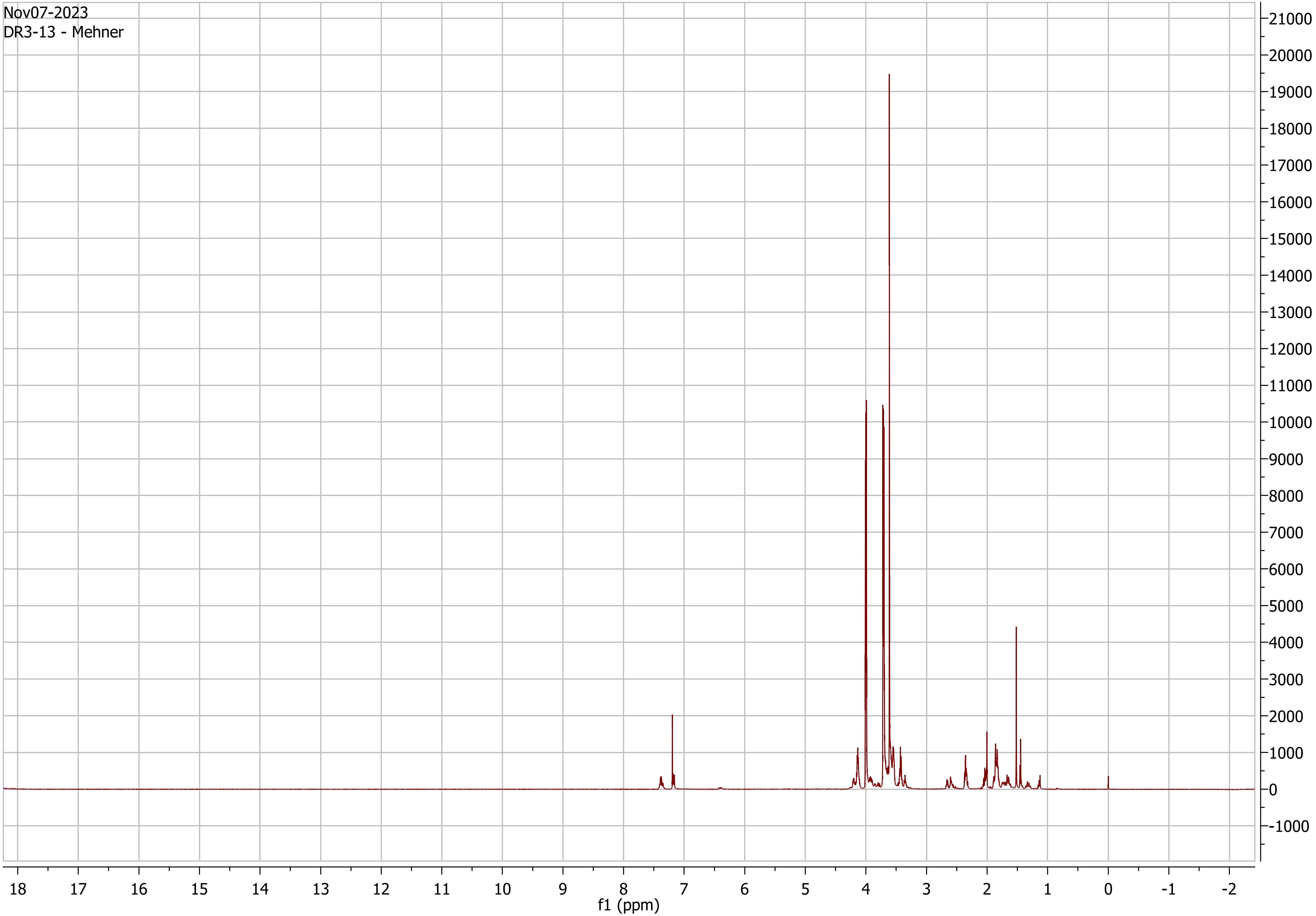

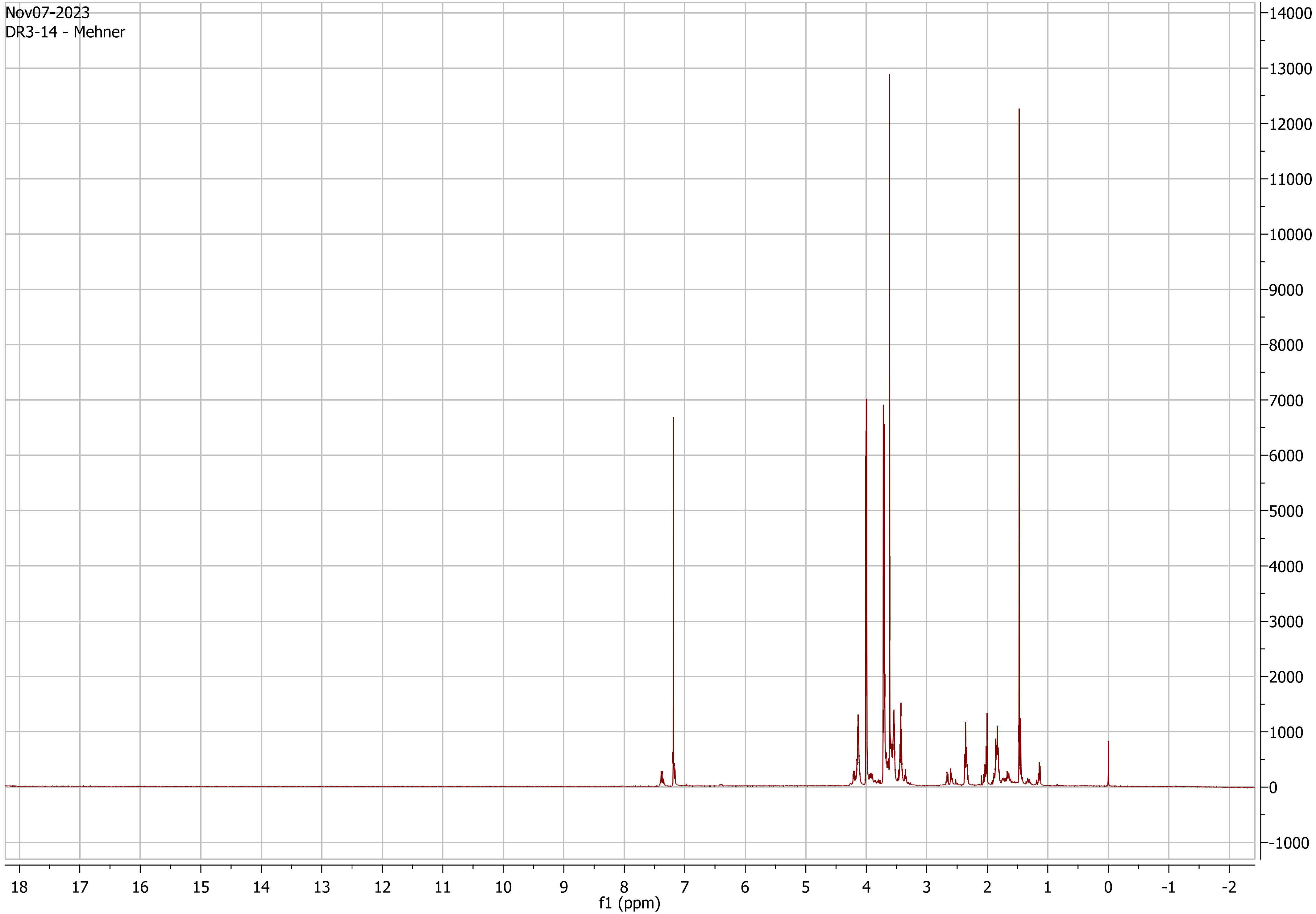
Nov07-2023
DR3-14 - Mehner
f1 (ppm)

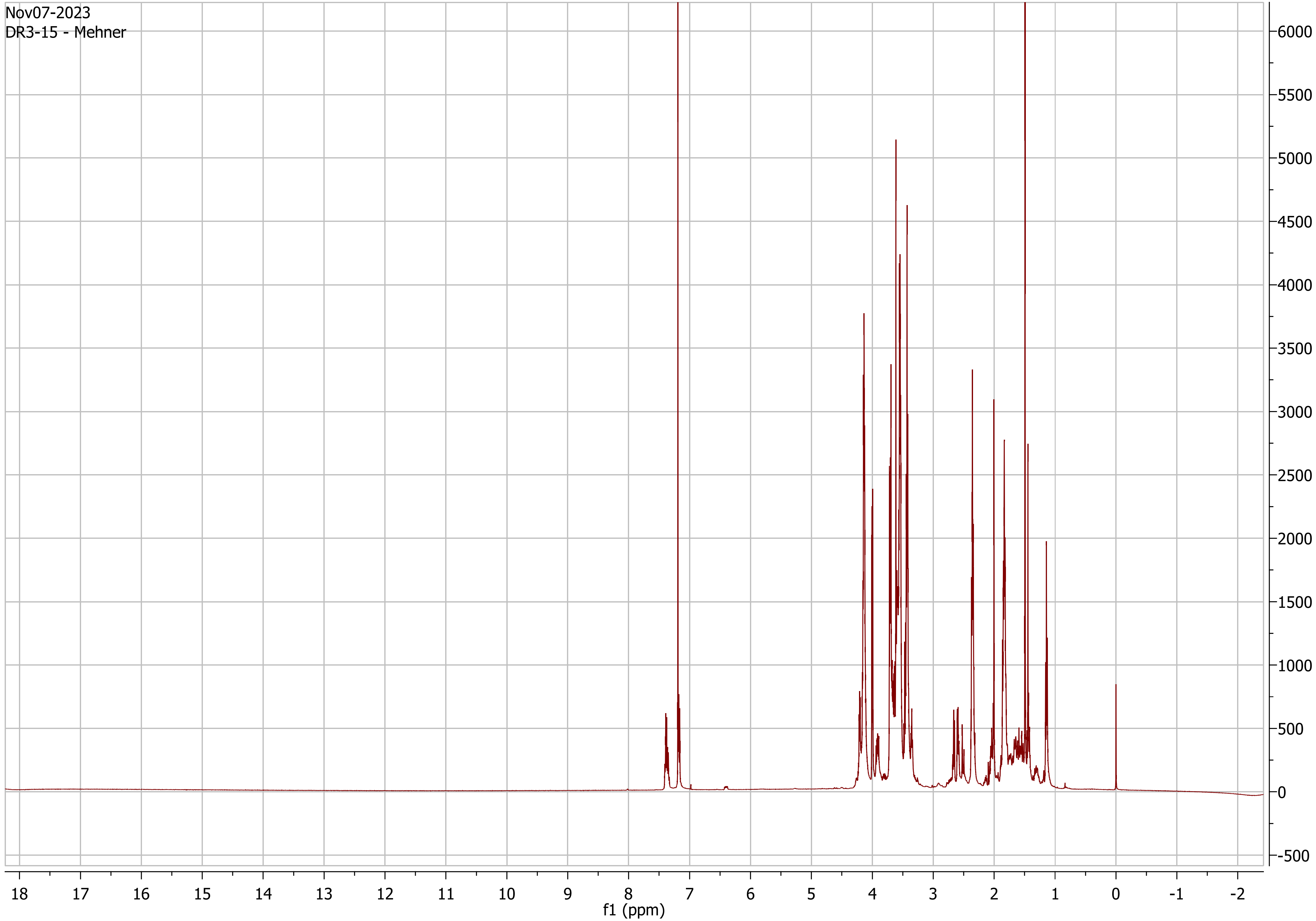

Nov07-2023
DR3-15 - Mehner
f1 (ppm)

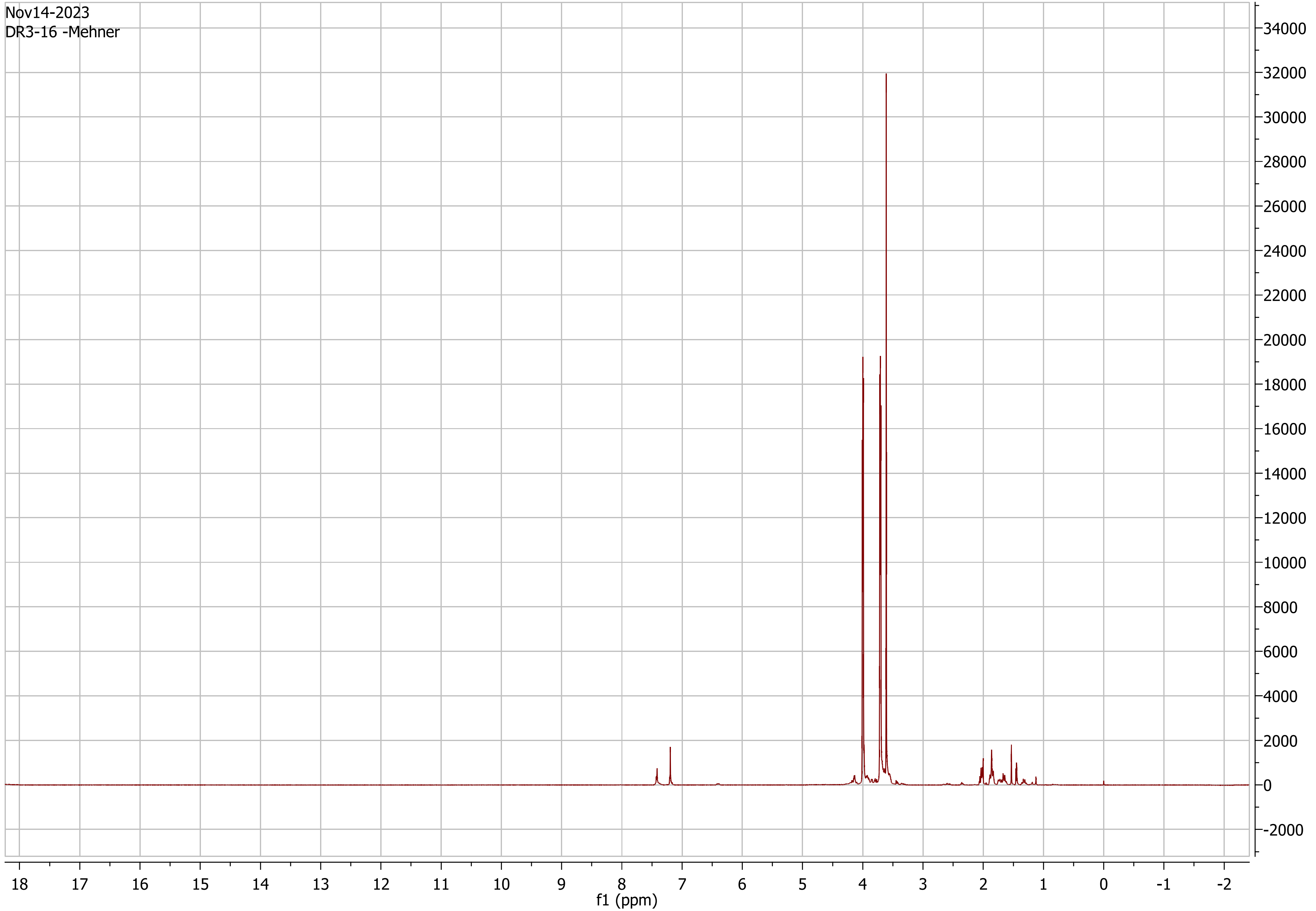
Nov14-2023
DR3-16 -Mehner
f1 (ppm)

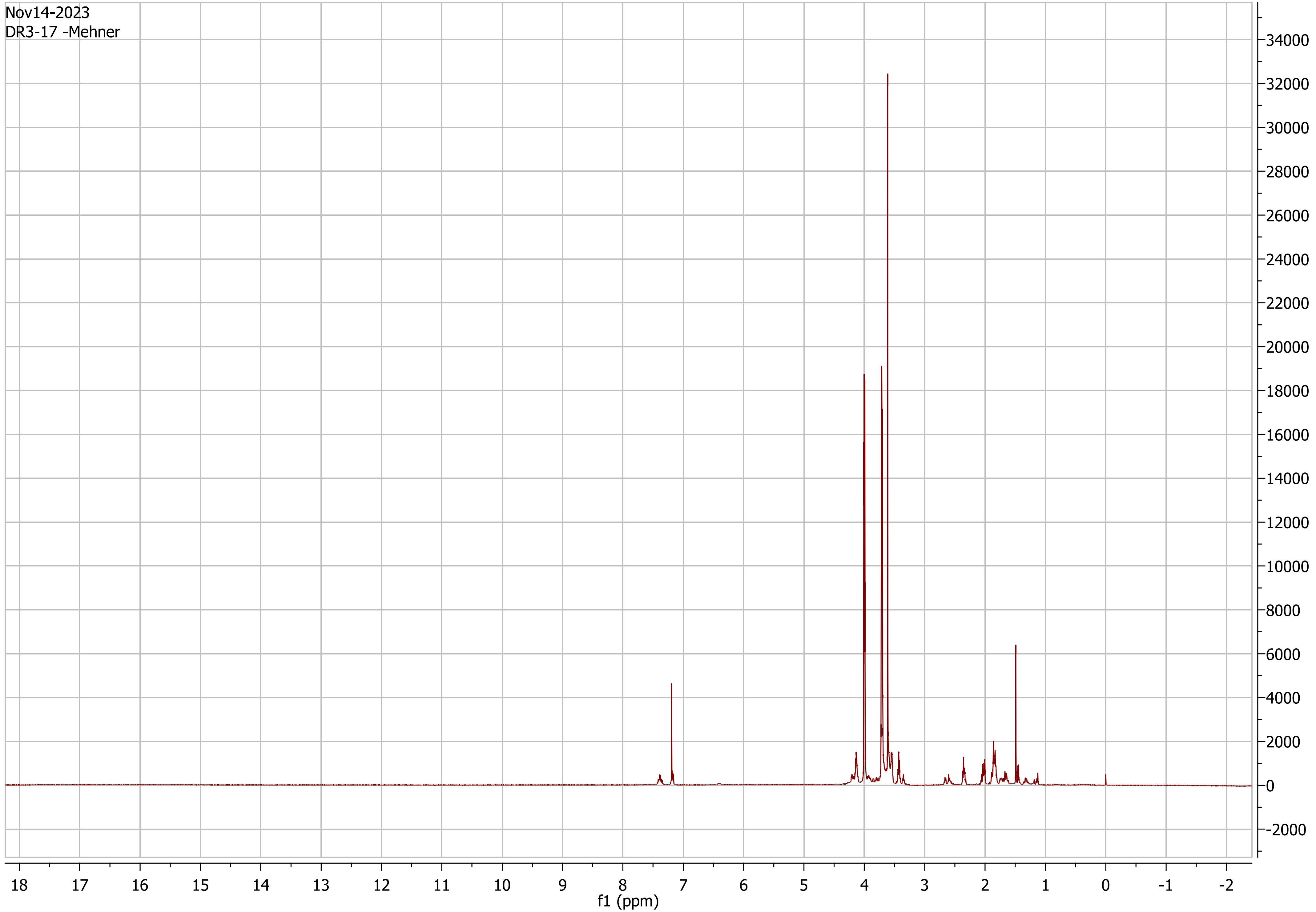
Nov14-2023
DR3-17 -Mehner
f1 (ppm)

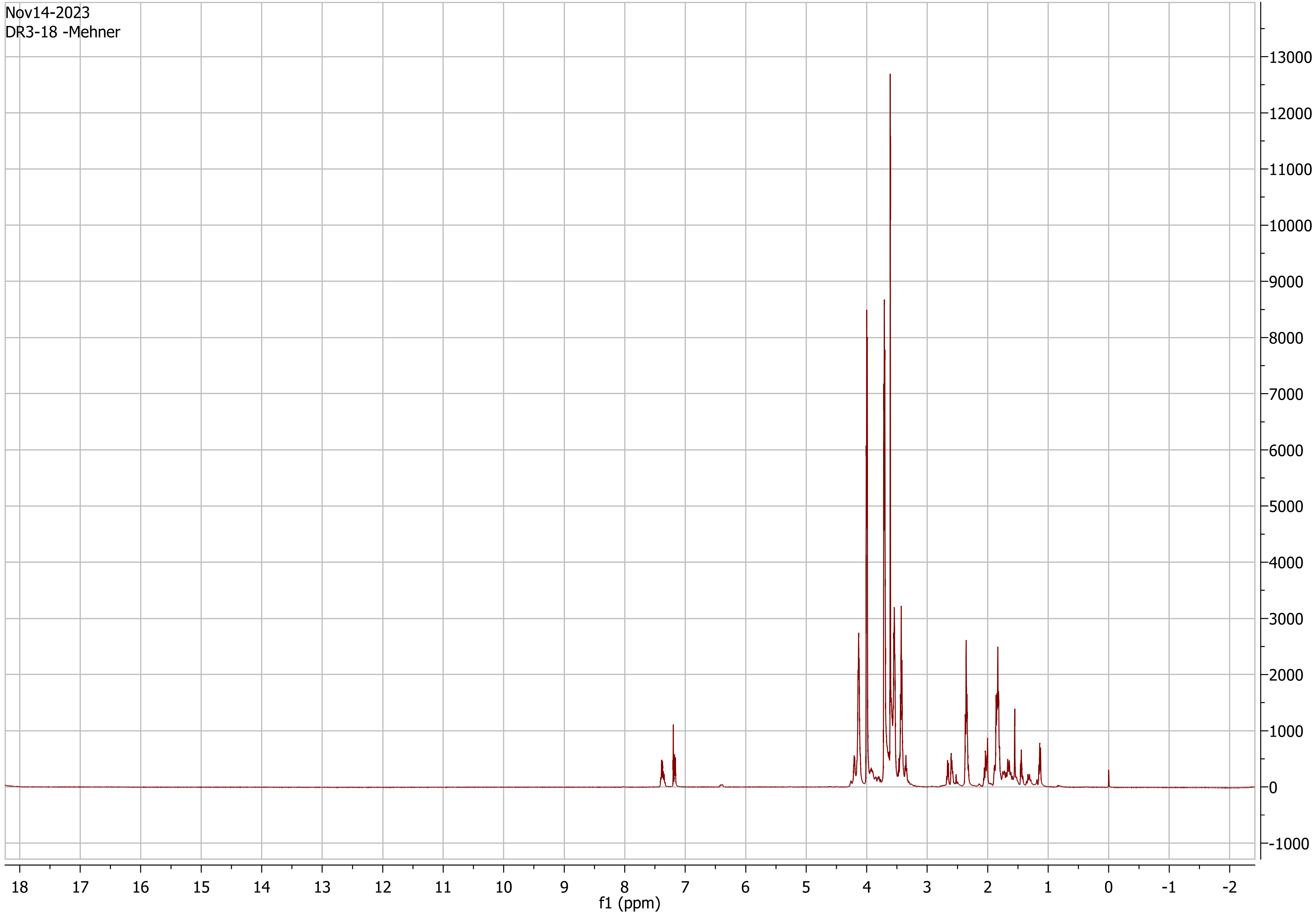

Nov14-2023
DR3-18 -Mehner
13000
12000
11000
10000
9000
8000
7000
6000
5000
4000
3000
2000
1000
0
-1000
18
17
16
15
14
13
12
11
10
9
8
7
6
5
4
3
2
1
0
-1
-2
f1 (ppm)

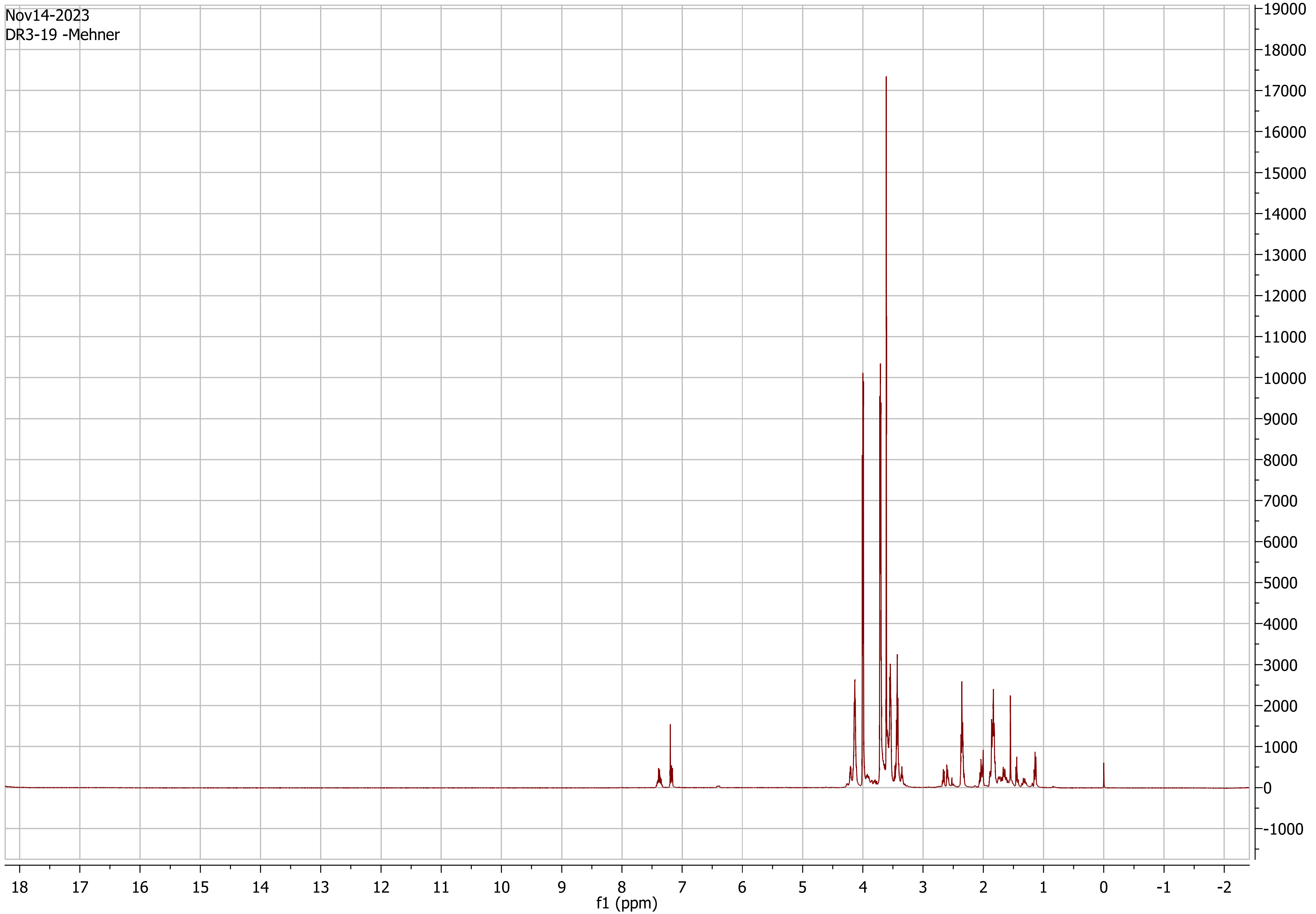

Nov14-2023
DR3-19 -Mehner
f1 (ppm)

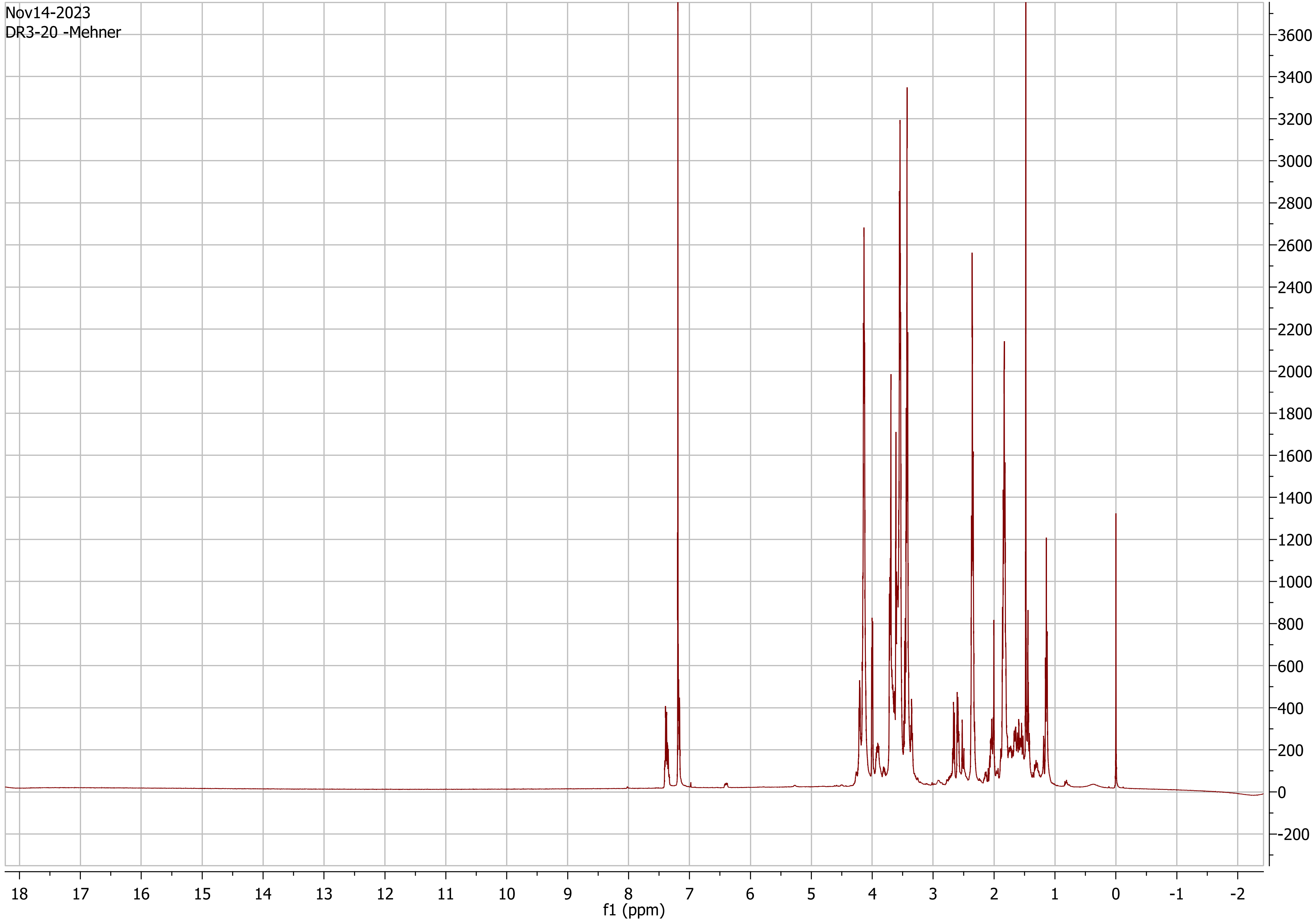
Nov14-2023
DR3-20 -Mehner
f1 (ppm)

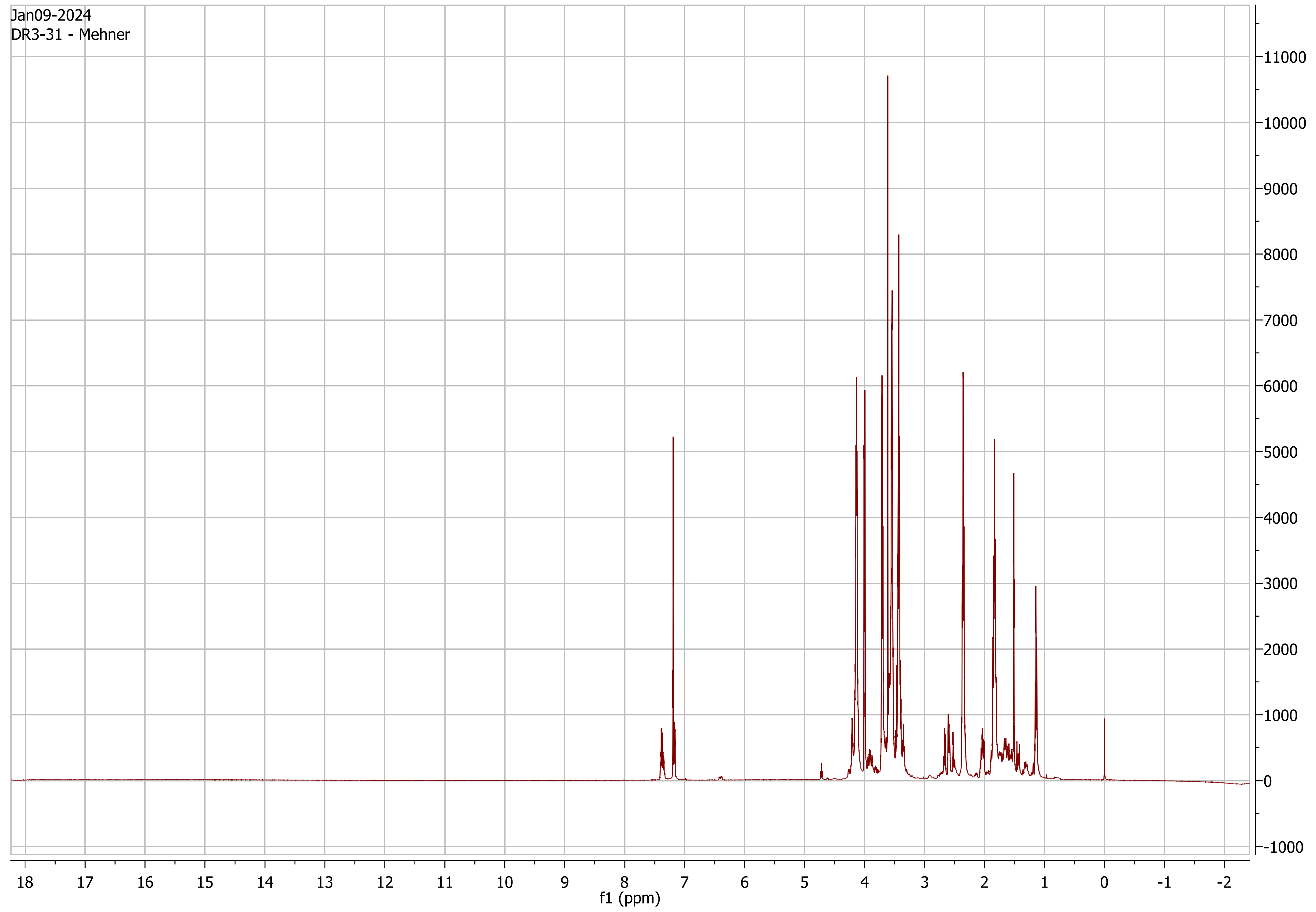

Jan09-2024
DR3-31 - Mehner
11000
10000
9000
8000
7000
6000
5000
4000
3000
2000
1000
0
-1000
18
17
16
15
14
13
12
11
10
9
8
7
6
5
4
3
2
1
0
-1
-2
f1 (ppm)

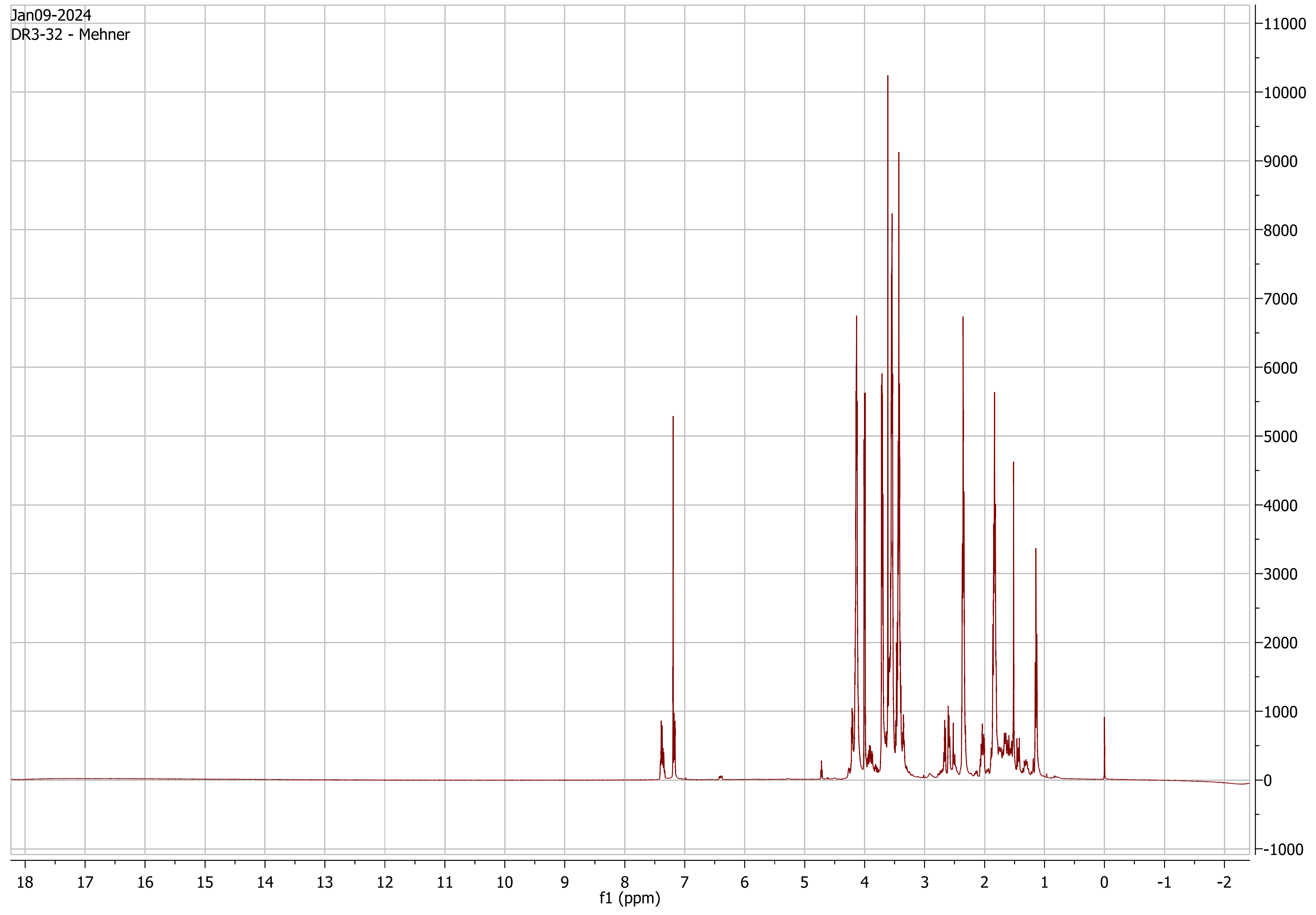

Jan09-2024
DR3-32 - Mehner
f1 (ppm)

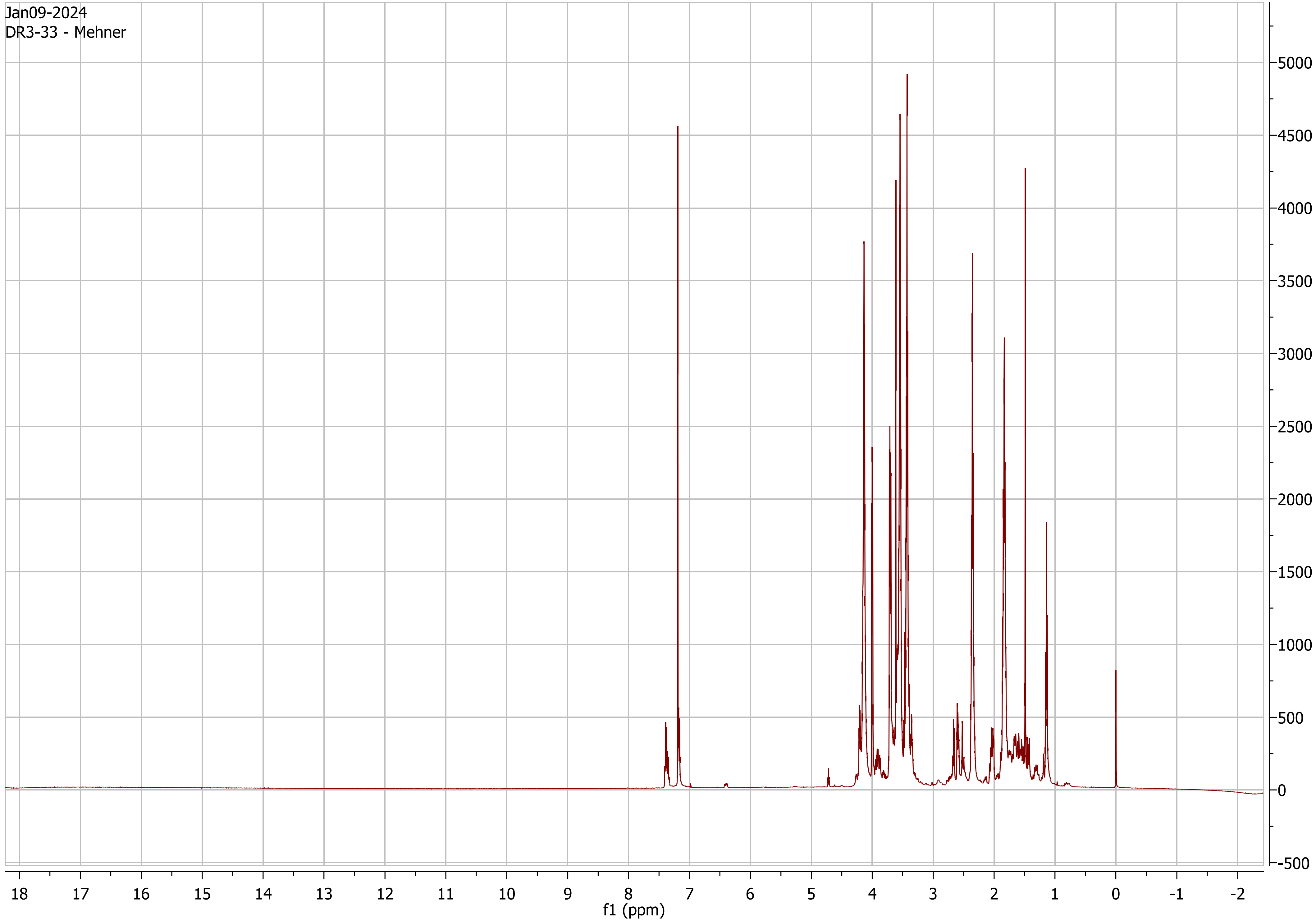

Jan09-2024
DR3-33 - Mehner
f1 (ppm)

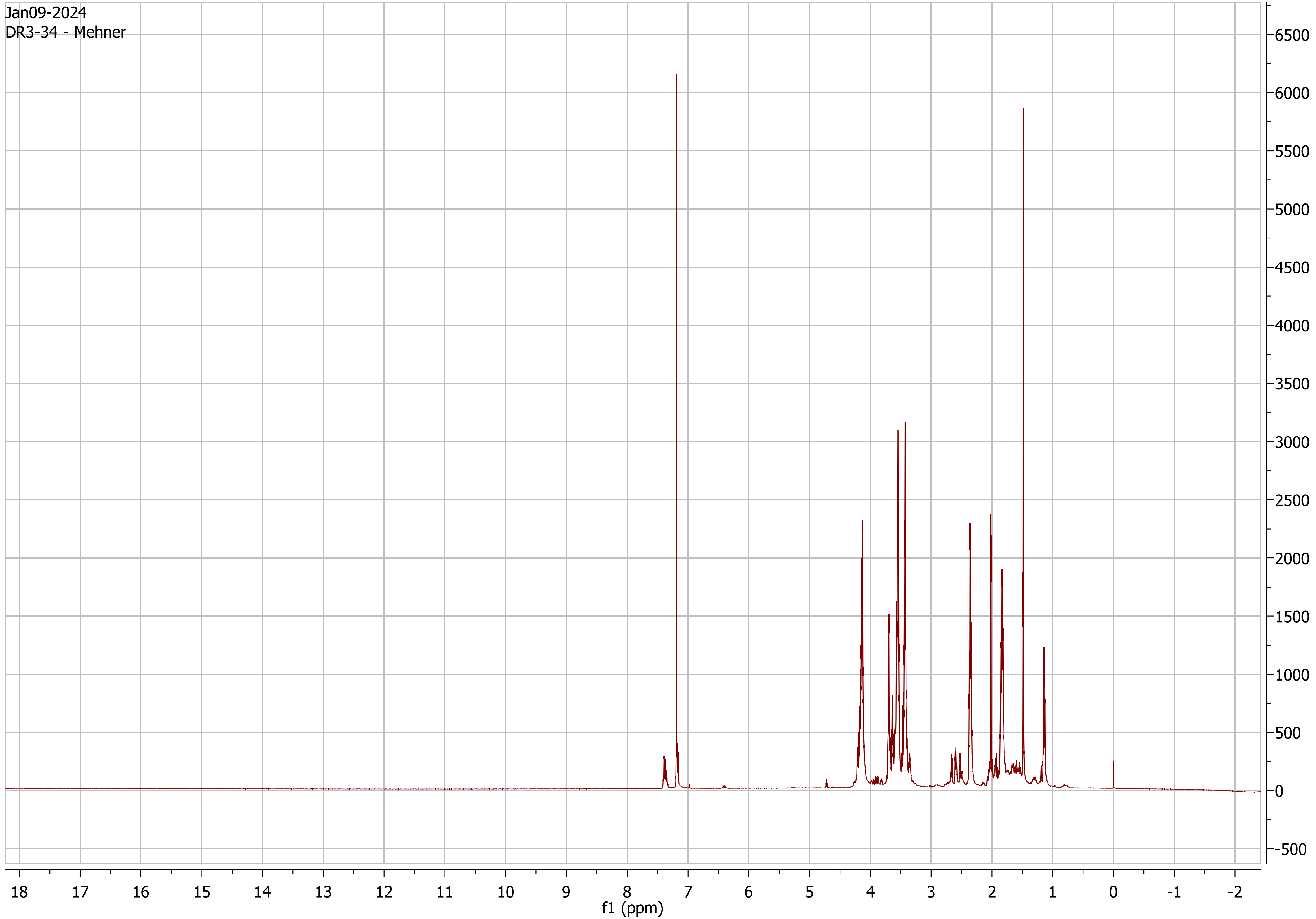

Jan09-2024
DR3-34 - Mehner
f1 (ppm)
18
17
16
15
14
13
12
11
10
9
8
7
6
5
4
3
2
1
0
-1
-2
6500
6000
5500
5000
4500
4000
3500
3000
2500
2000
1500
1000
500
0
-500

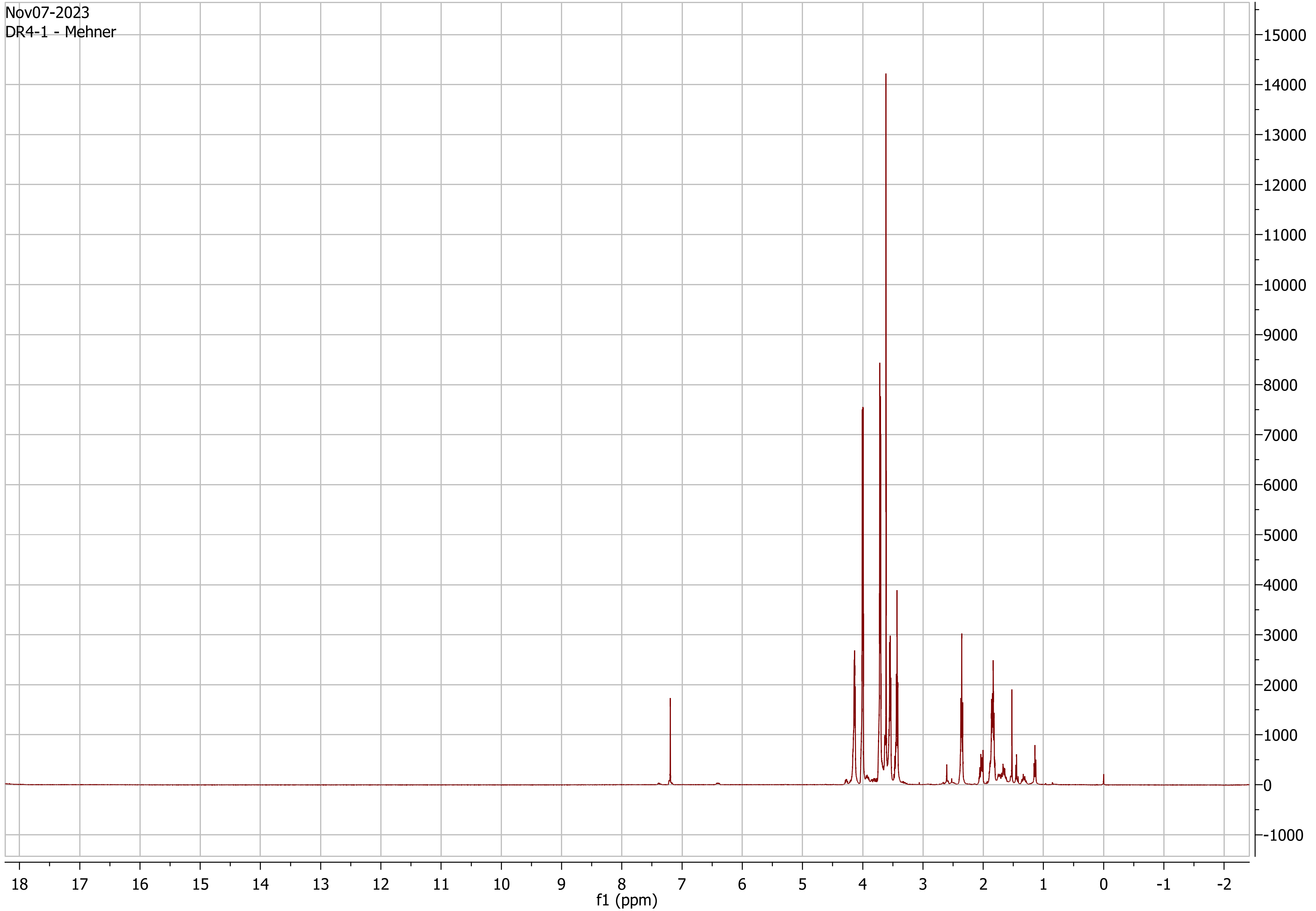

Nov07-2023
DR4-1 - Mehner
f1 (ppm)

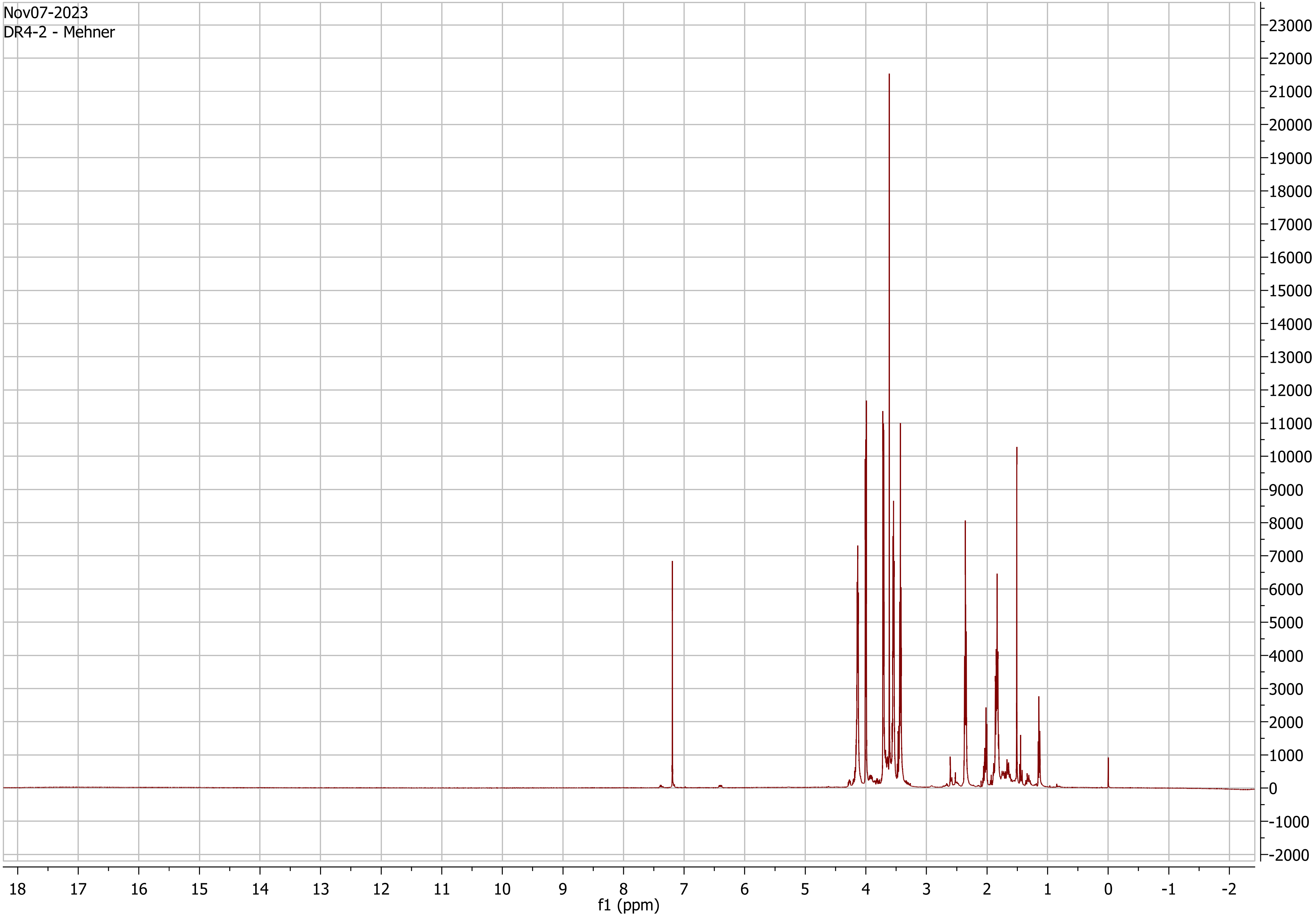
Nov07-2023
DR4-2 - Mehner
f1 (ppm)

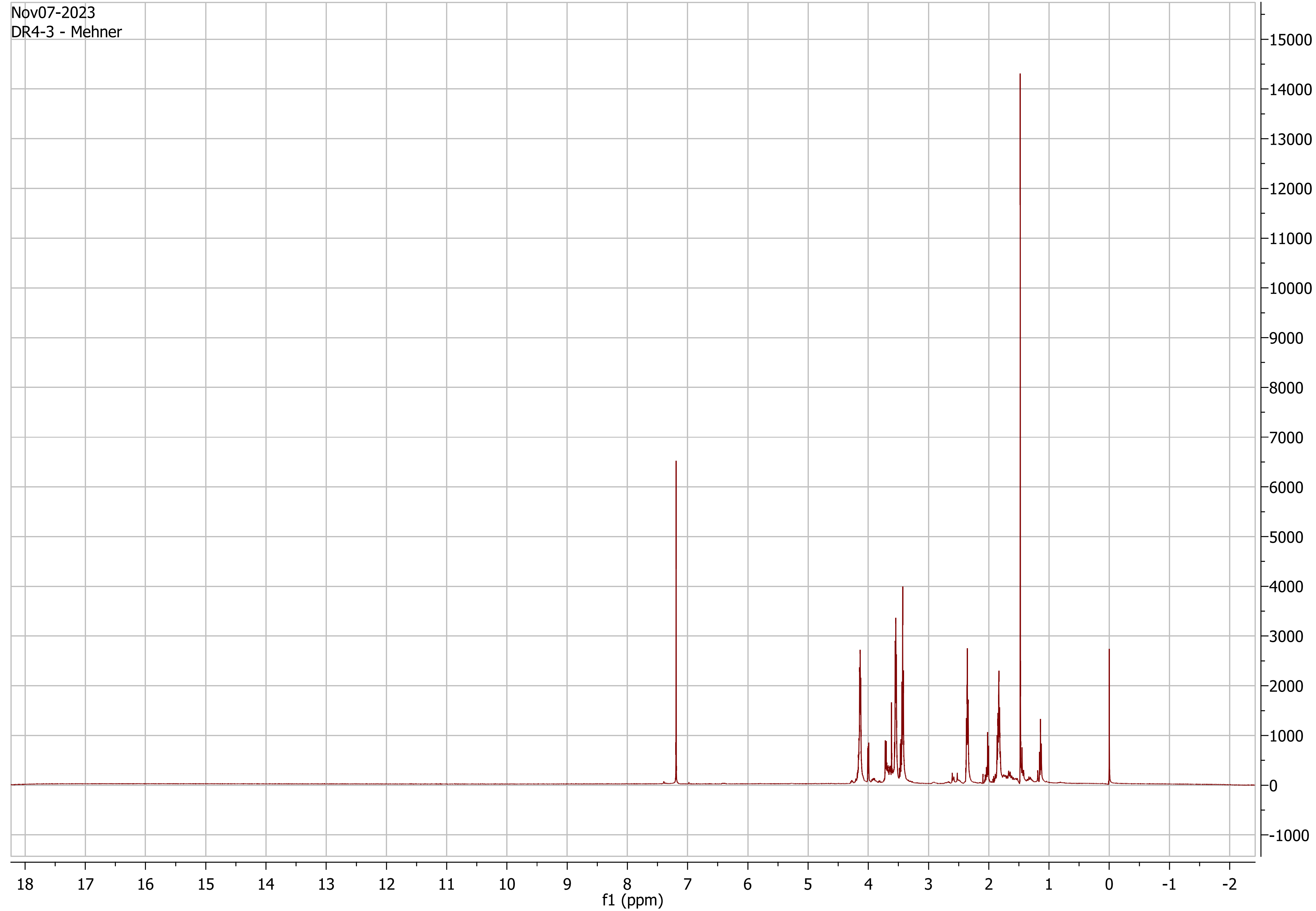
Nov07-2023
DR4-3 - Mehner
f1 (ppm)

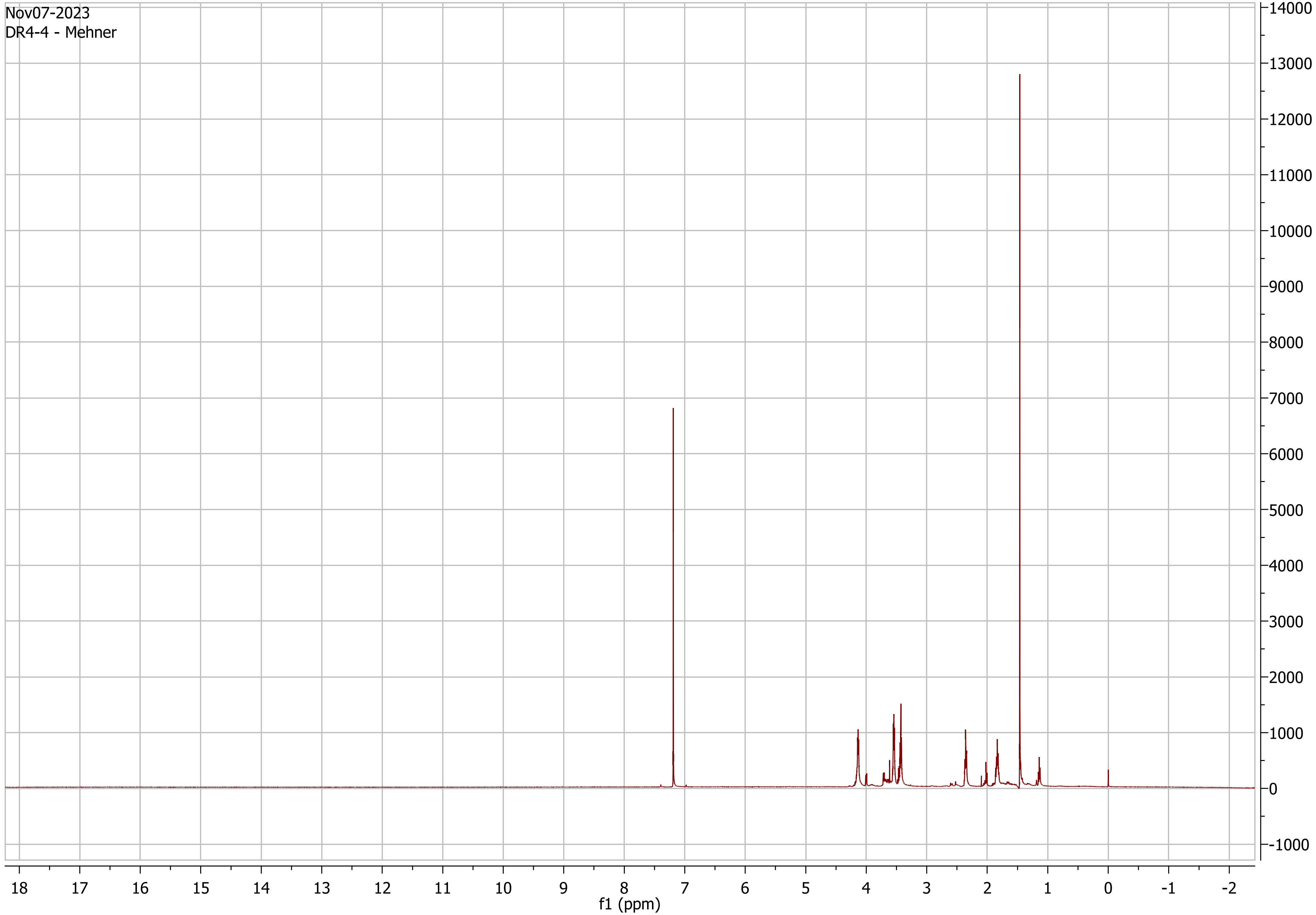
Nov07-2023
DR4-4 - Mehner
f1 (ppm)

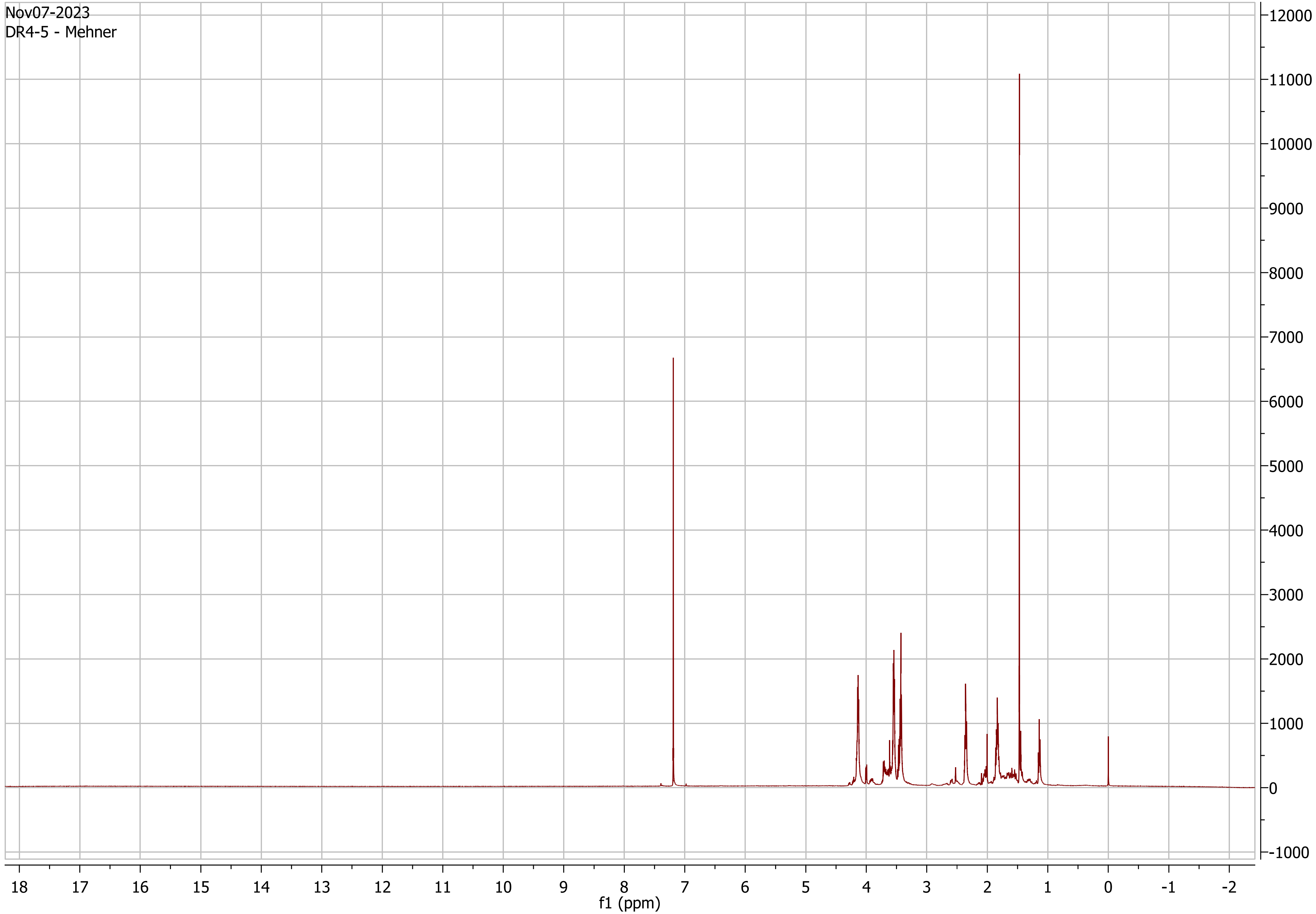

Nov07-2023
DR4-5 - Mehner
f1 (ppm)

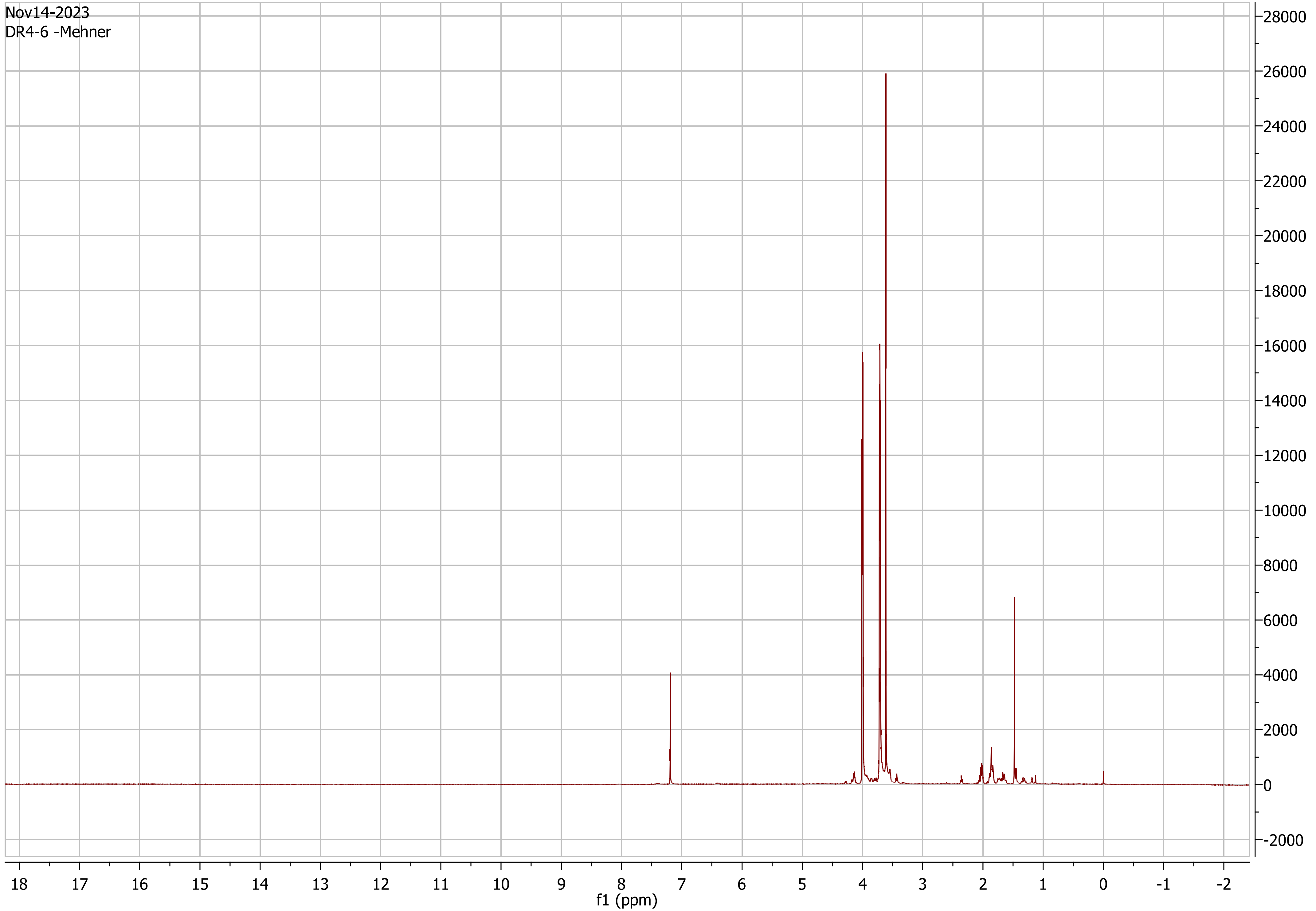
Nov14-2023
DR4-6 -Mehner
f1 (ppm)

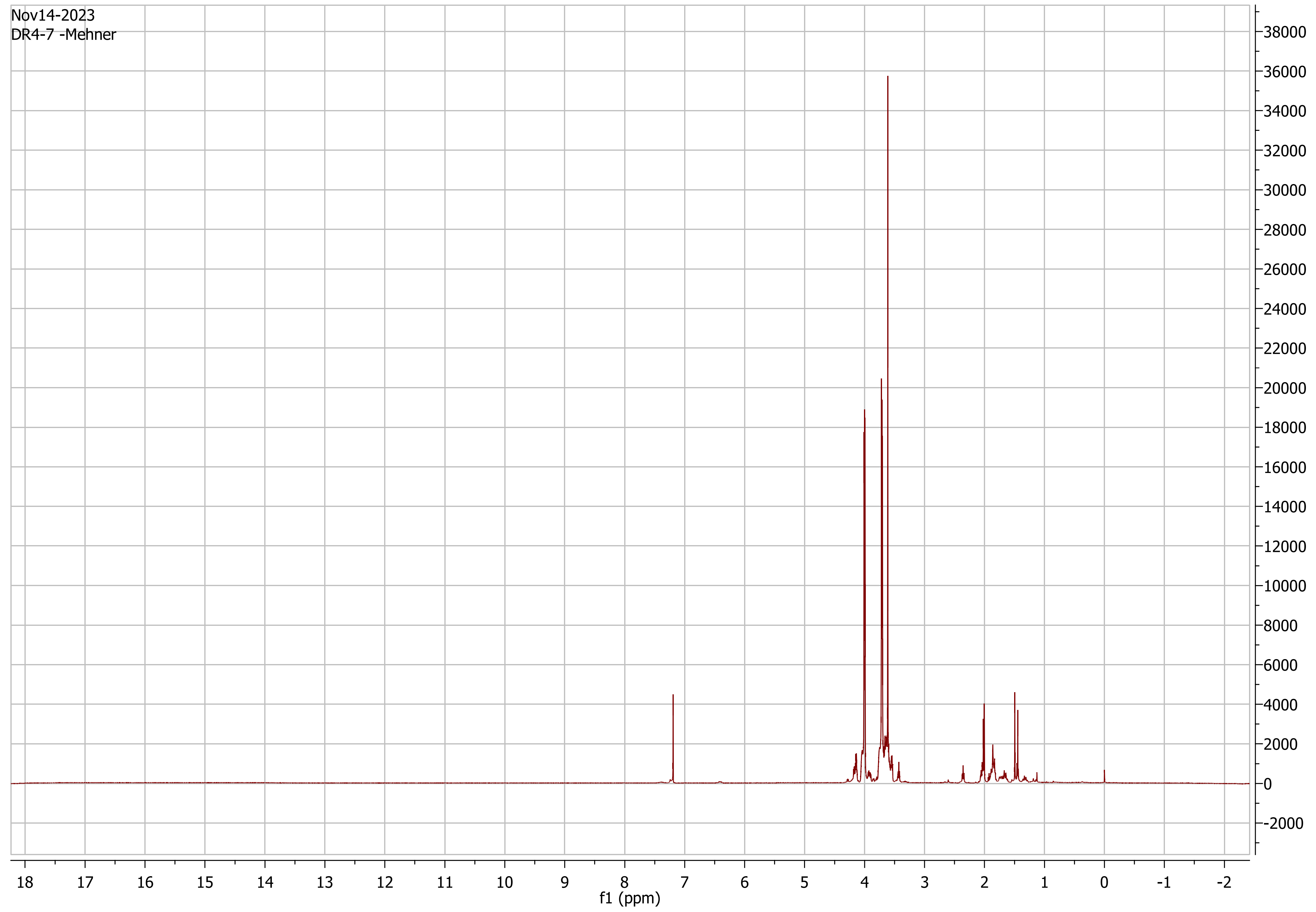
Nov14-2023
DR4-7 -Mehner
f1 (ppm)

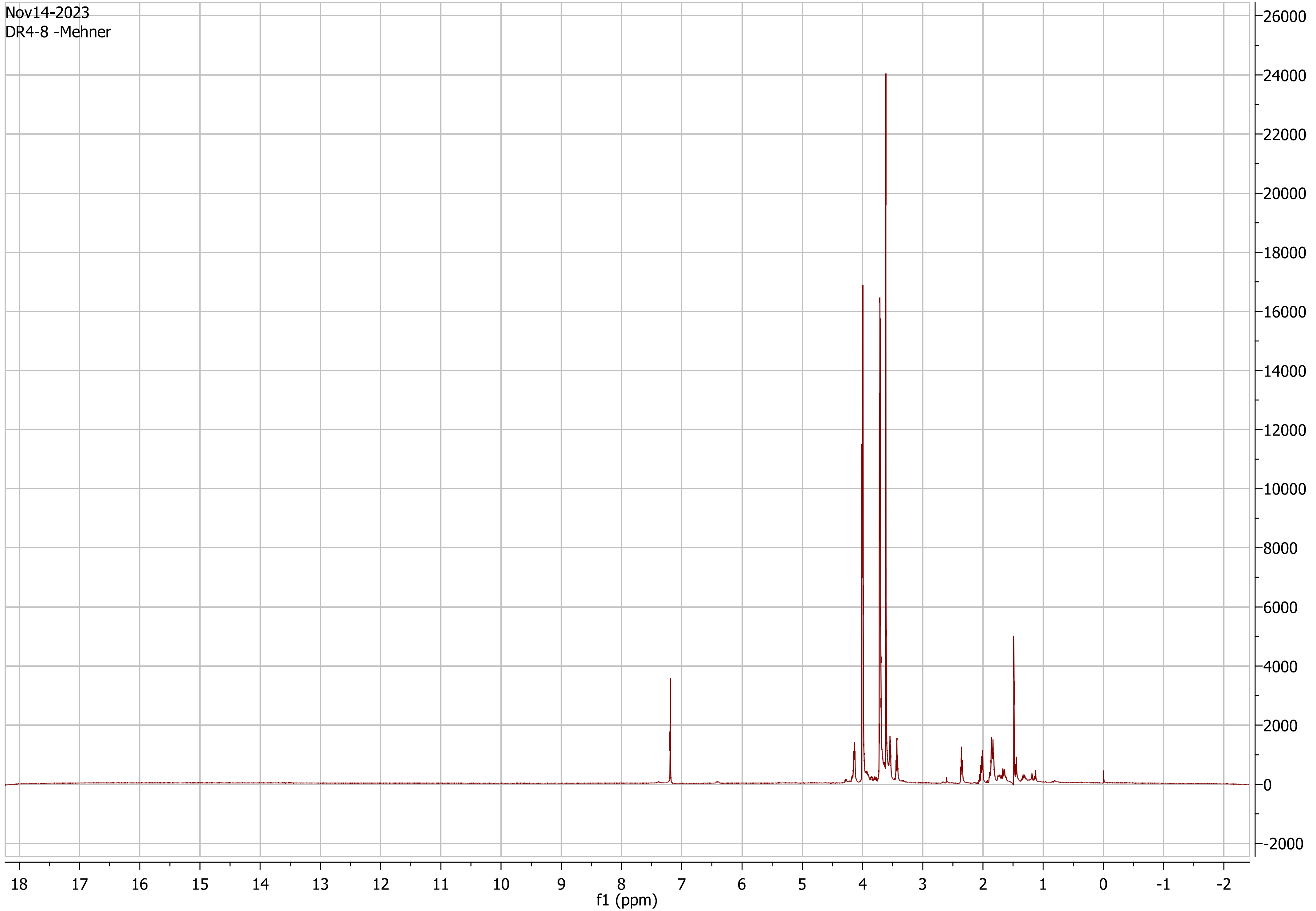

Nov14-2023
DR4-8 -Mehner
f1 (ppm)

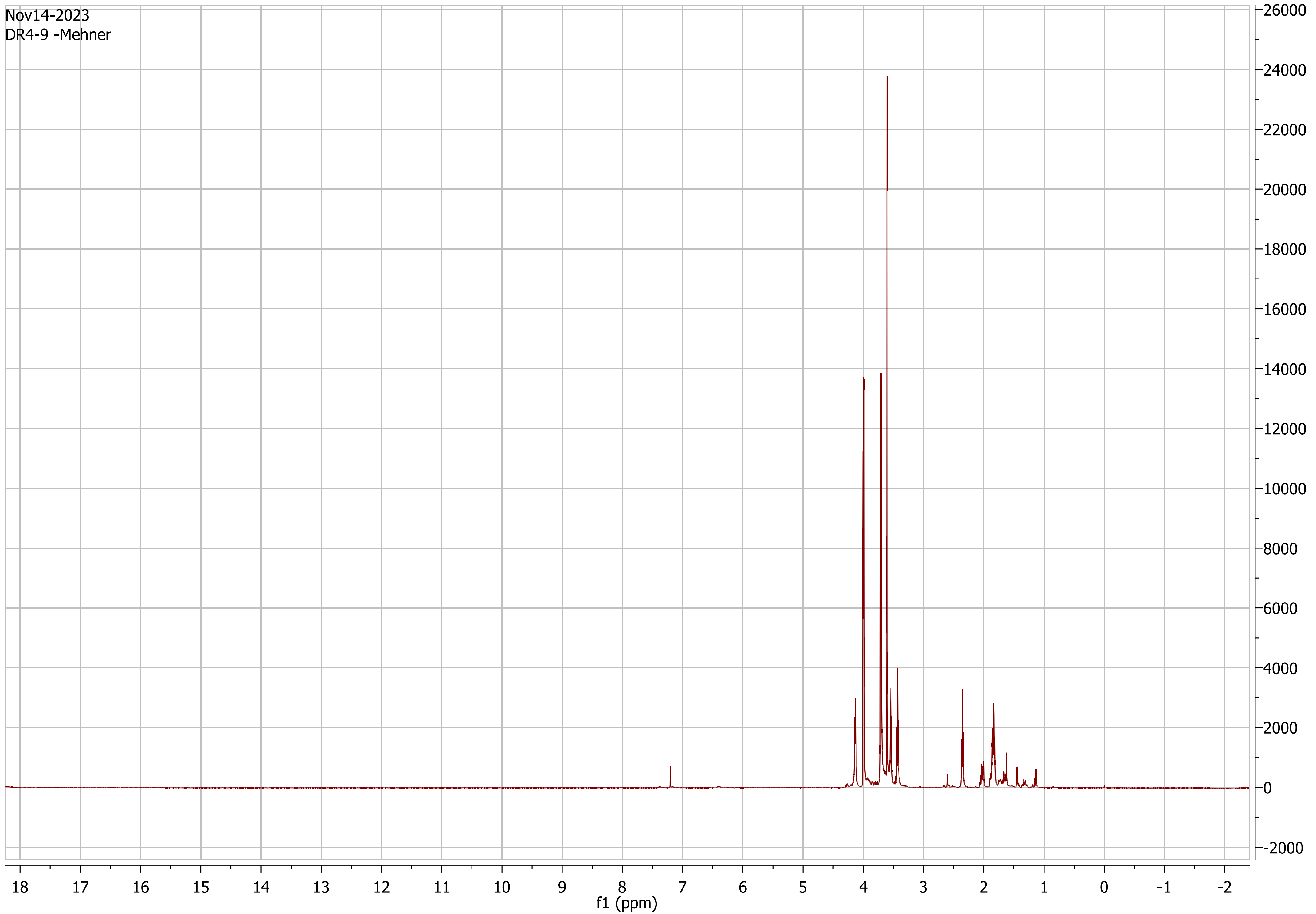
Nov14-2023
DR4-9 -Mehner
f1 (ppm)

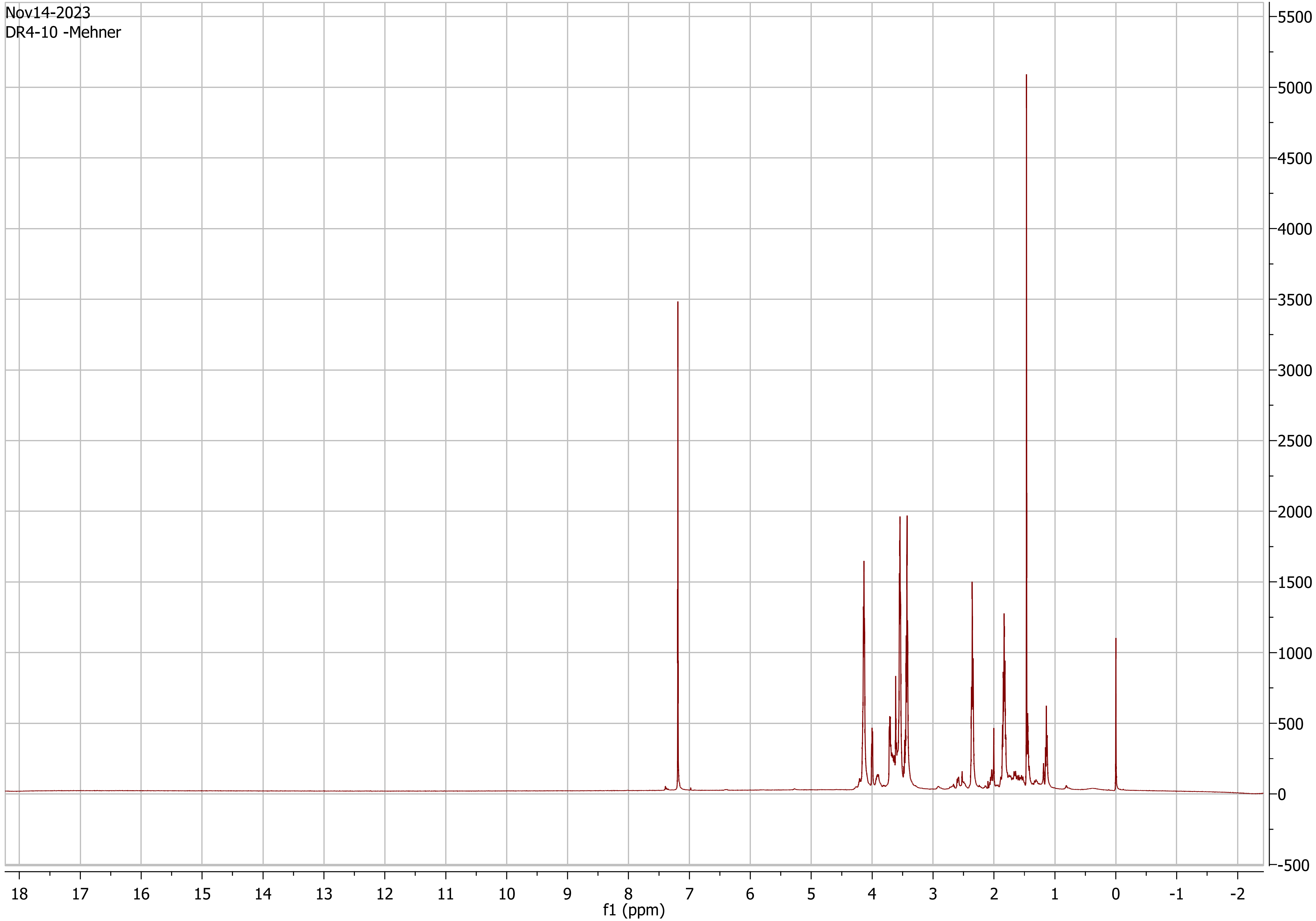

Nov14-2023
DR4-10 -Mehner
5500
5000
4500
4000
3500
3000
2500
2000
1500
1000
500
0
-500
18
17
16
15
14
13
12
11
10
9
8
7
6
5
4
3
2
1
0
-1
-2
f1 (ppm)

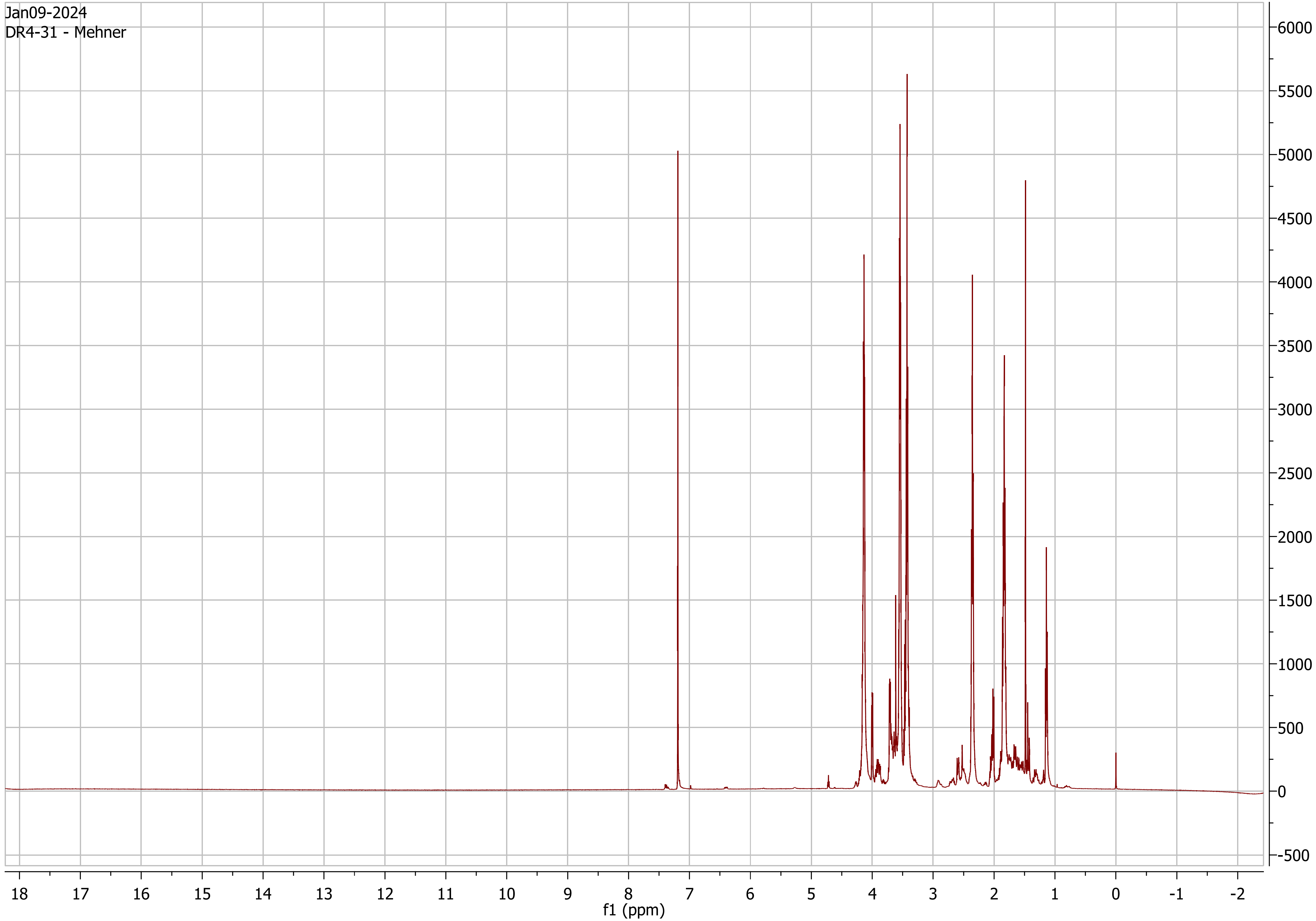

Jan09-2024
DR4-31 - Mehner
f1 (ppm)

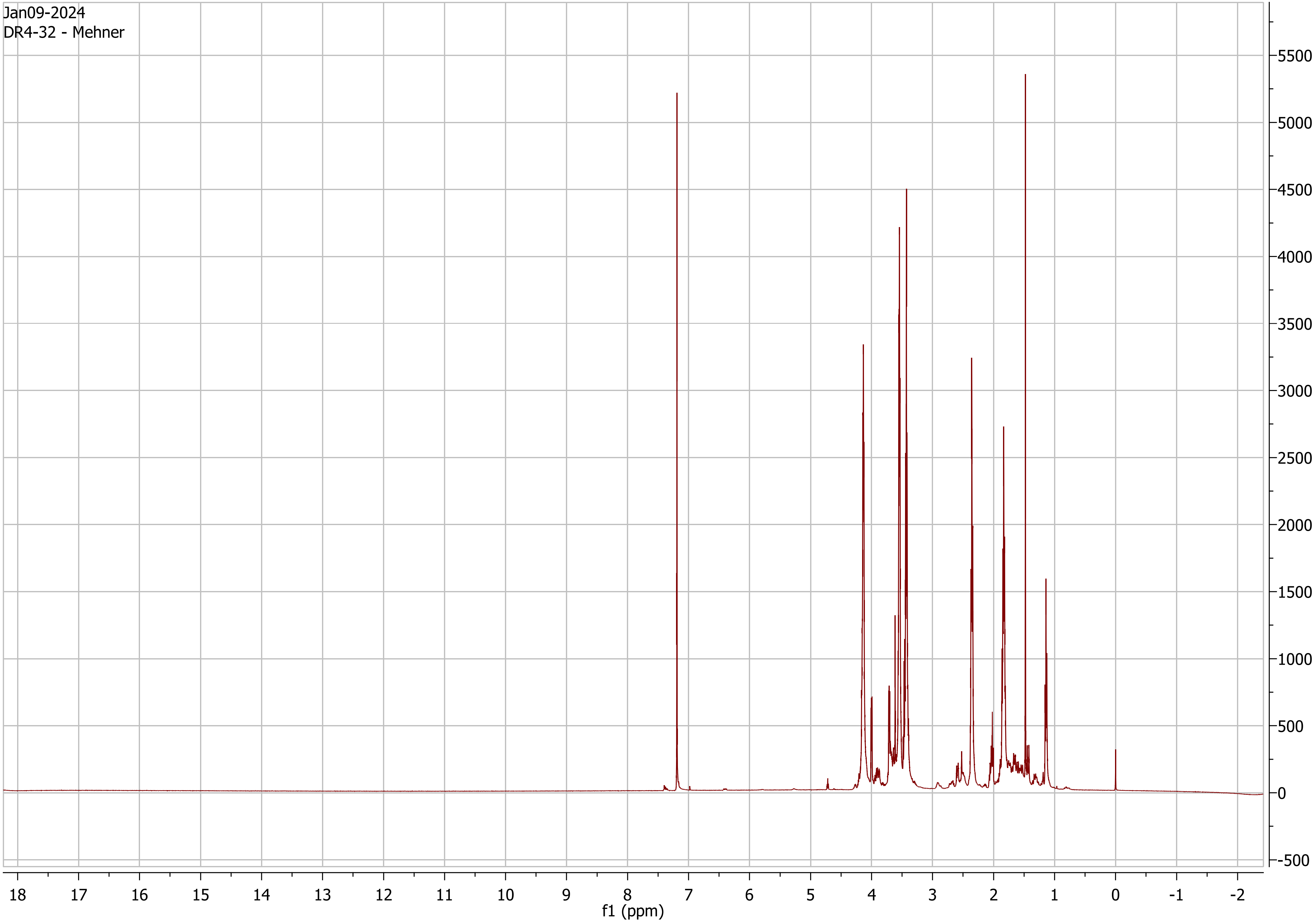
Jan09-2024
DR4-32 - Mehner
f1 (ppm)

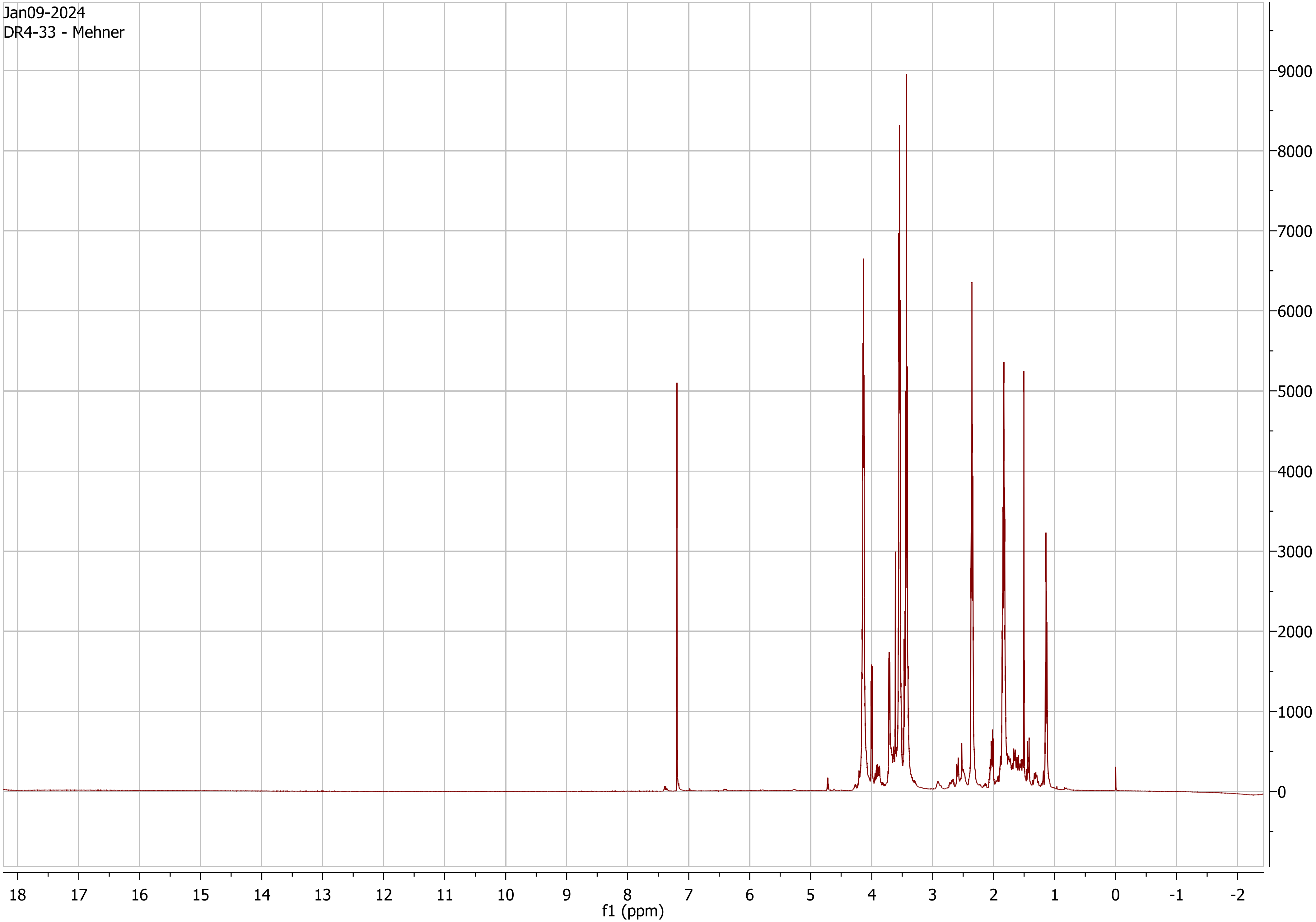

Jan09-2024
DR4-33 - Mehner
9000
8000
7000
6000
5000
4000
3000
2000
1000
0
18
17
16
15
14
13
12
11
10
9
8
7
6
5
4
3
2
1
0
-1
-2
f1 (ppm)

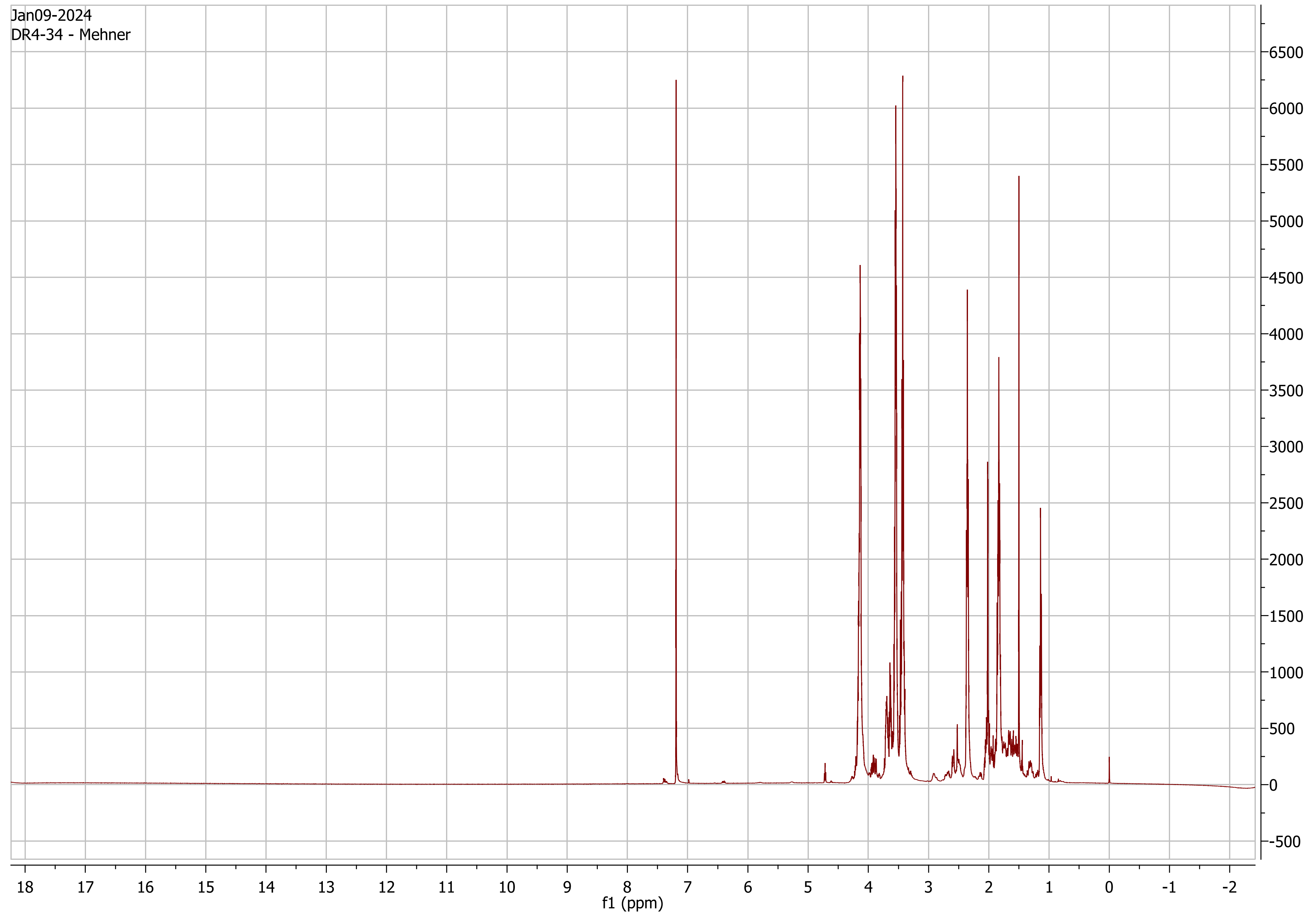
Jan09-2024
DR4-34 - Mehner
f1 (ppm)

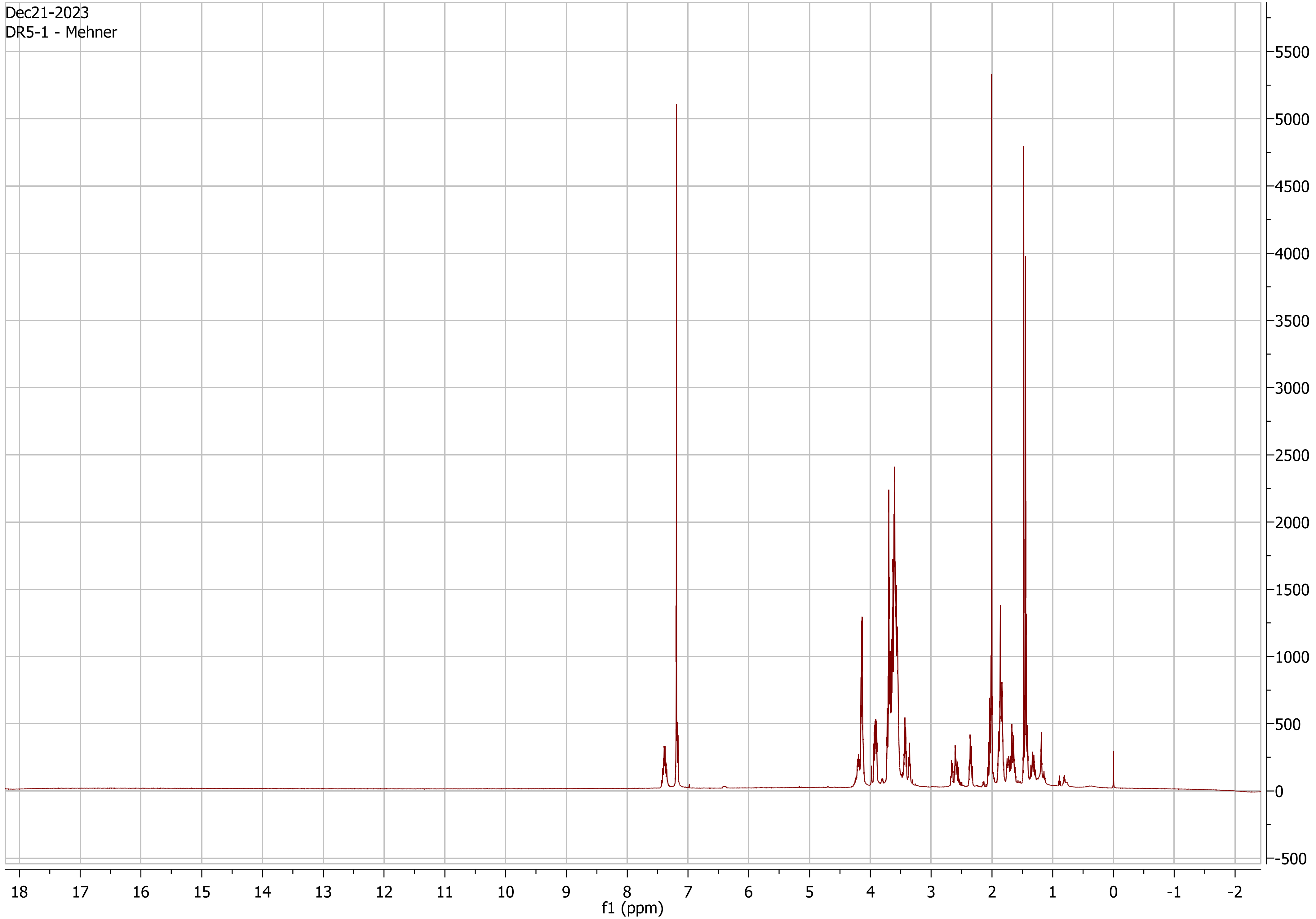
Dec21-2023
DR5-1 - Mehner
f1 (ppm)

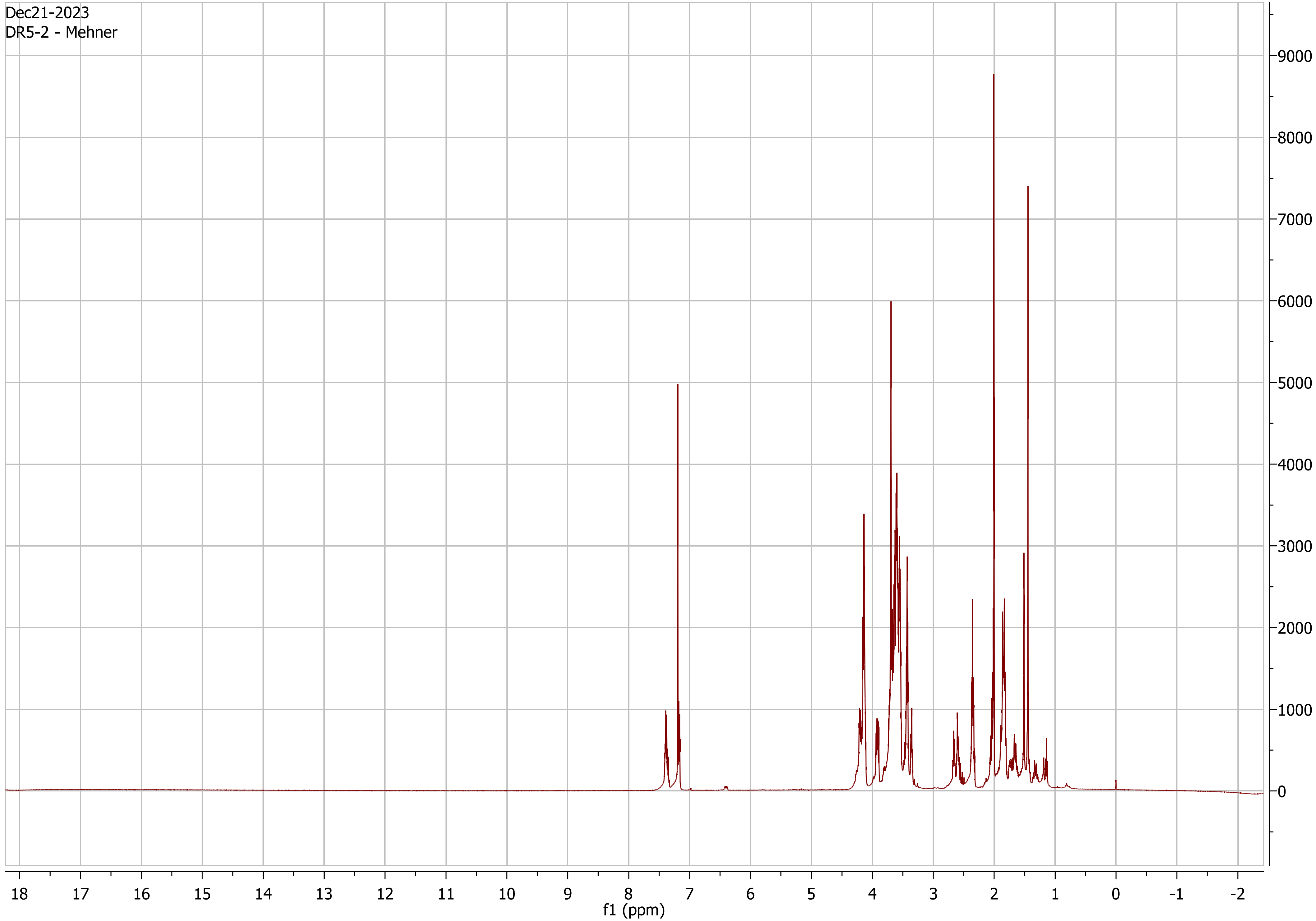

Dec21-2023
DR5-2 - Mehner
9000
8000
7000
6000
5000
4000
3000
2000
1000
0
18
17
16
15
14
13
12
11
10
9
8
7
6
5
4
3
2
1
0
-1
-2
f1 (ppm)

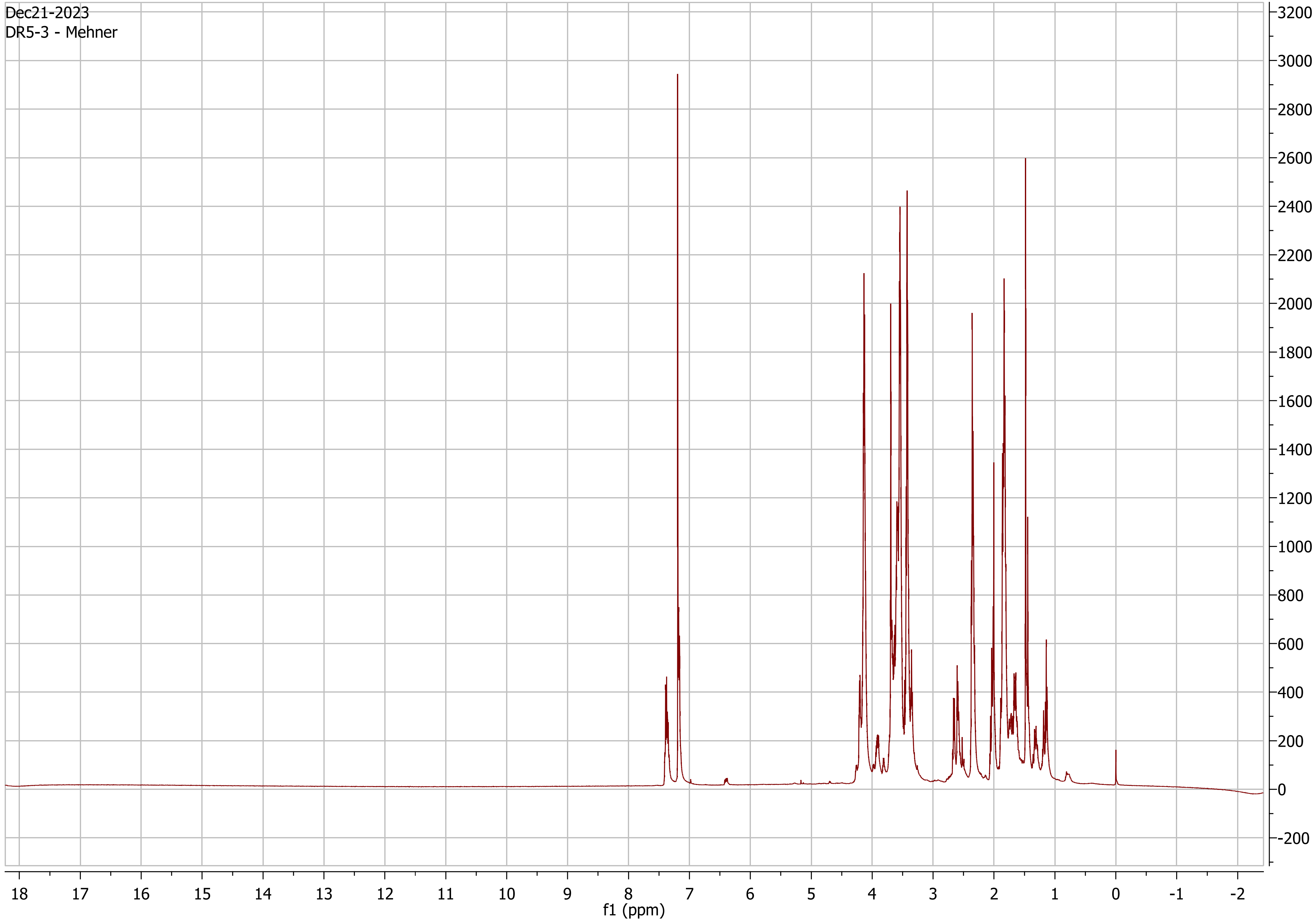

Dec21-2023
DR5-3 - Mehner
f1 (ppm)

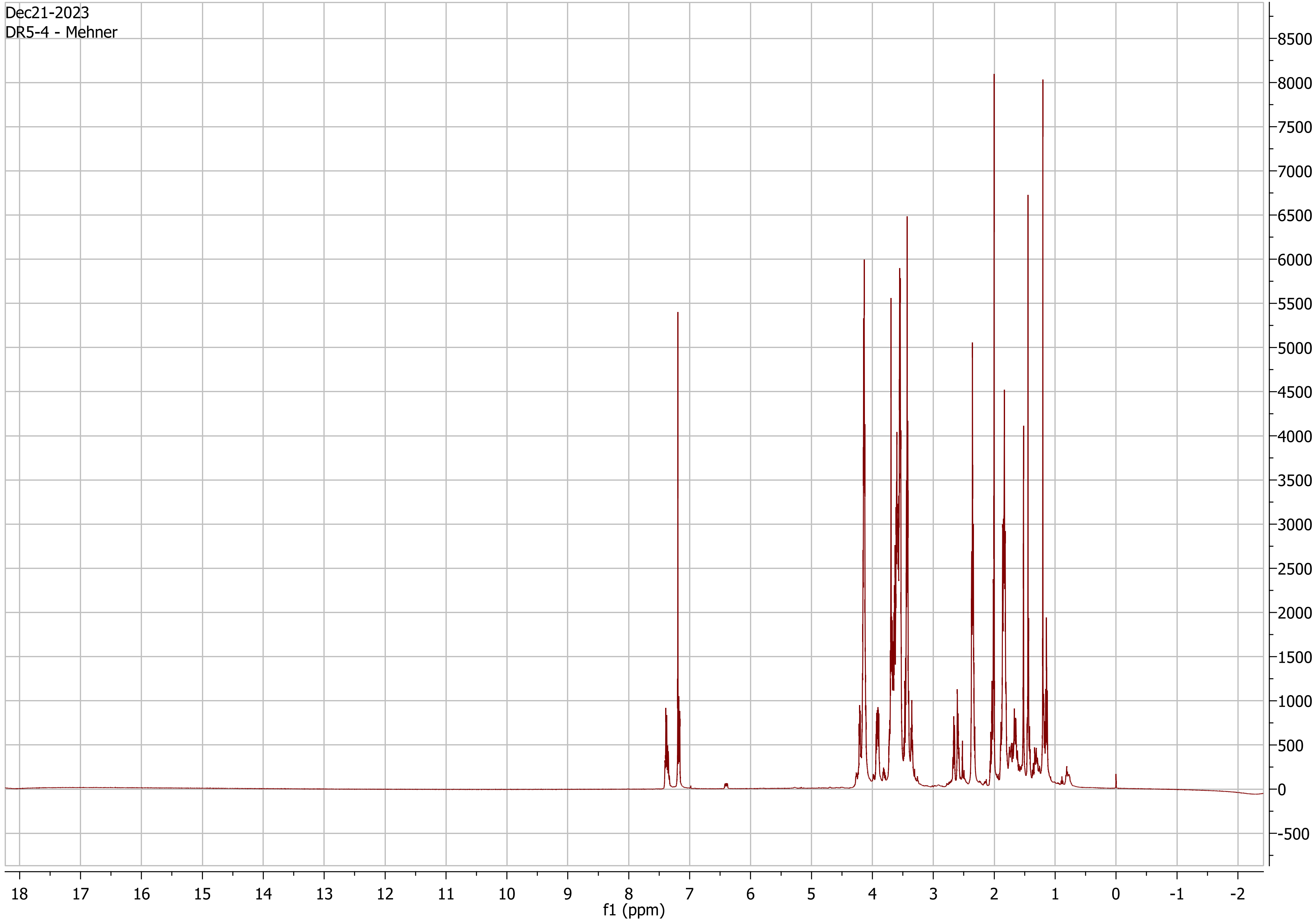
Dec21-2023
DR5-4 - Mehner
8500
8000
7500
7000
6500
6000
5500
5000
4500
4000
3500
3000
2500
2000
1500
1000
500
0
-500
18
17
16
15
14
13
12
11
10
9
8
7
6
5
4
3
2
1
0
-1
-2
f1 (ppm)

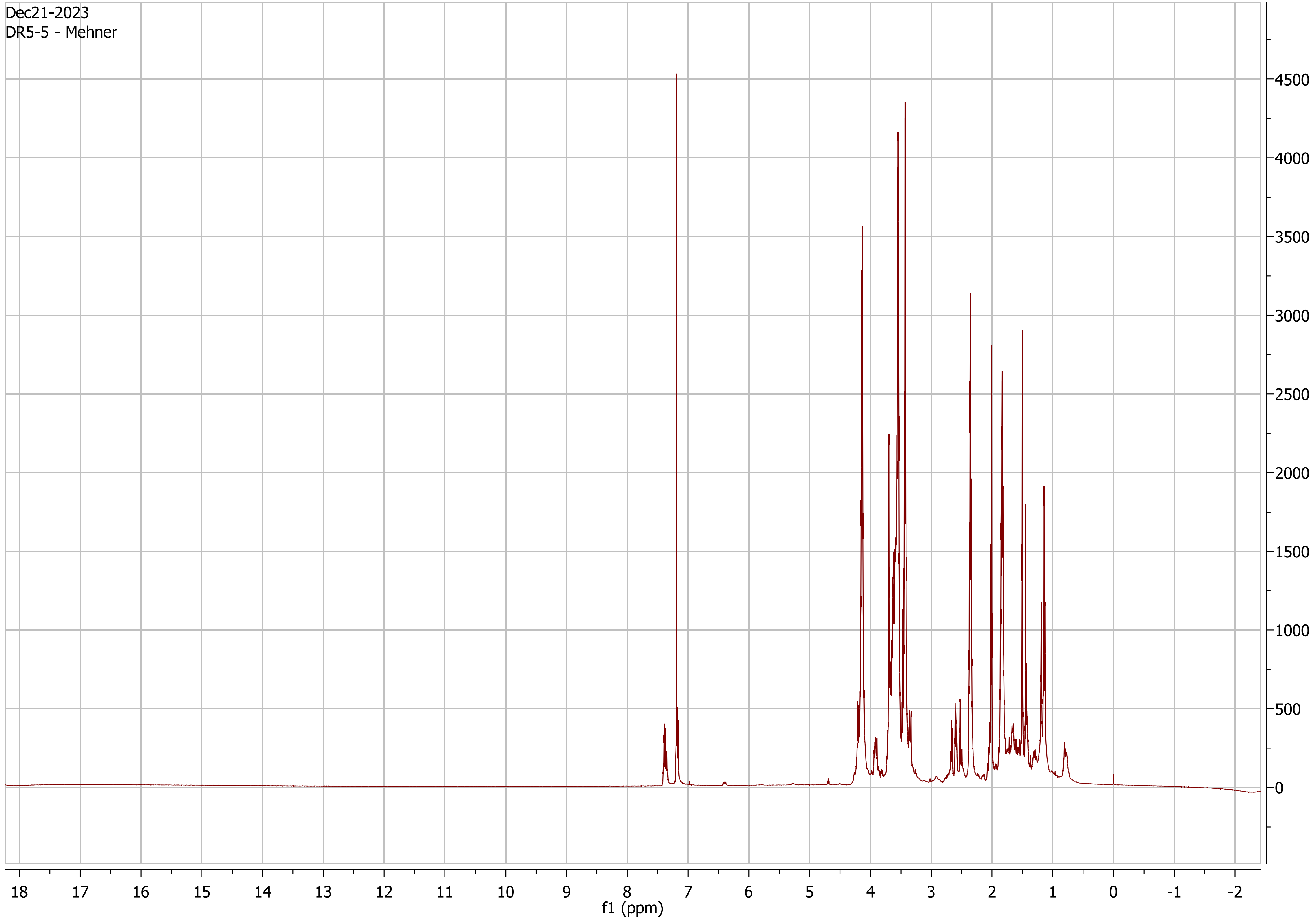
Dec21-2023
DR5-5 - Mehner
f1 (ppm)

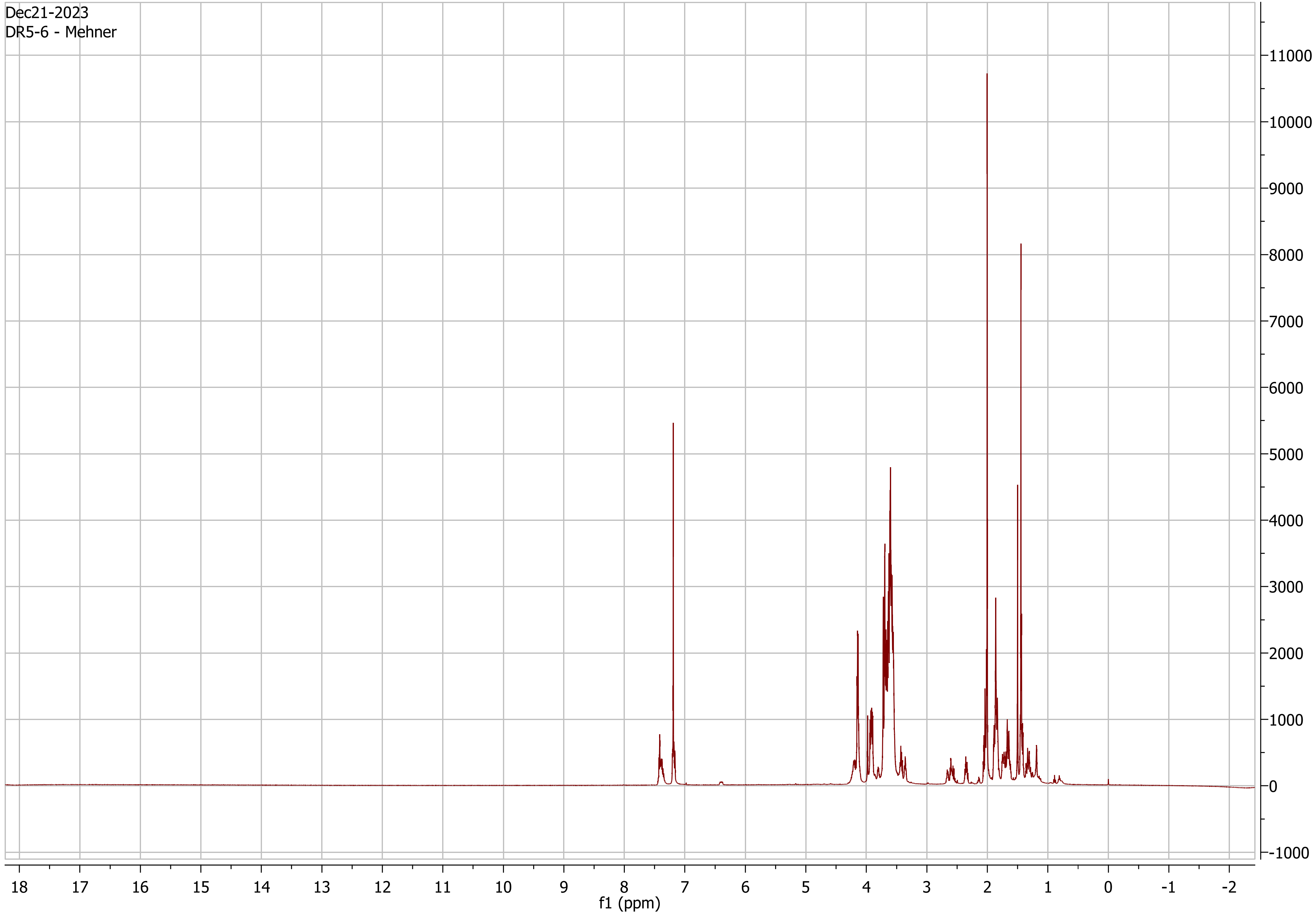

Dec21-2023
DR5-6 - Mehner
f1 (ppm)

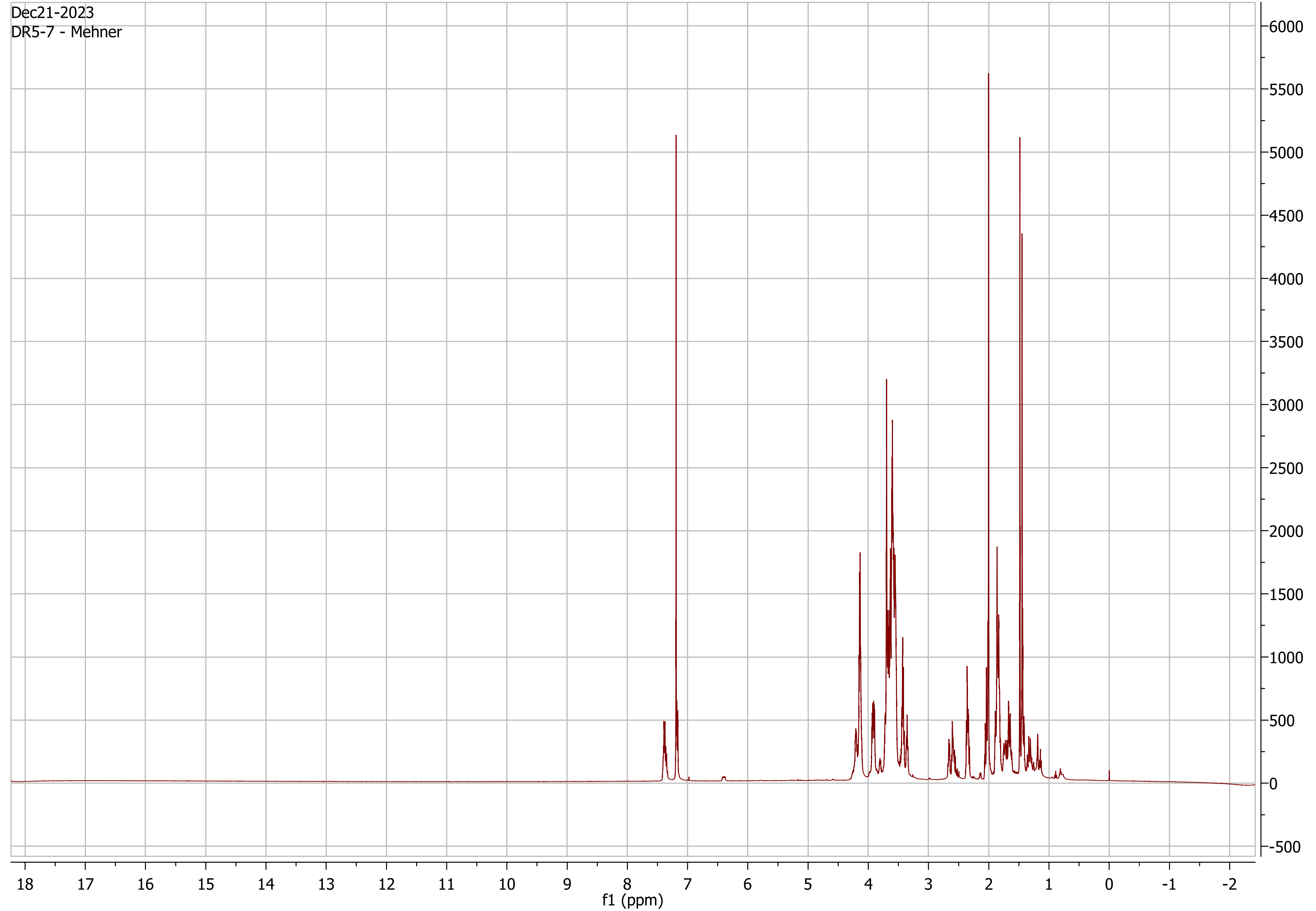
Dec21-2023
DR5-7 - Mehner
f1 (ppm)

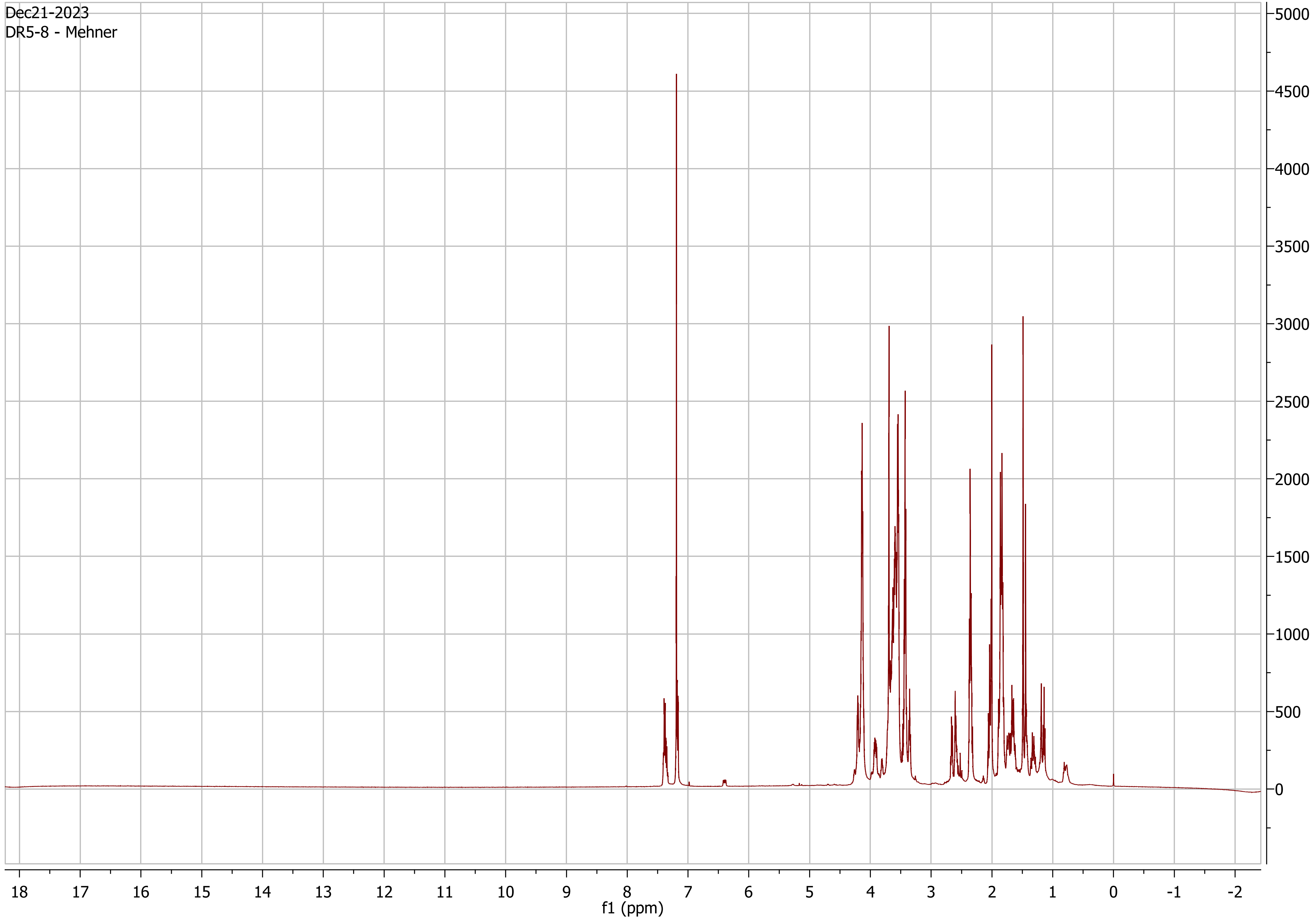
Dec21-2023
DR5-8 - Mehner
f1 (ppm)

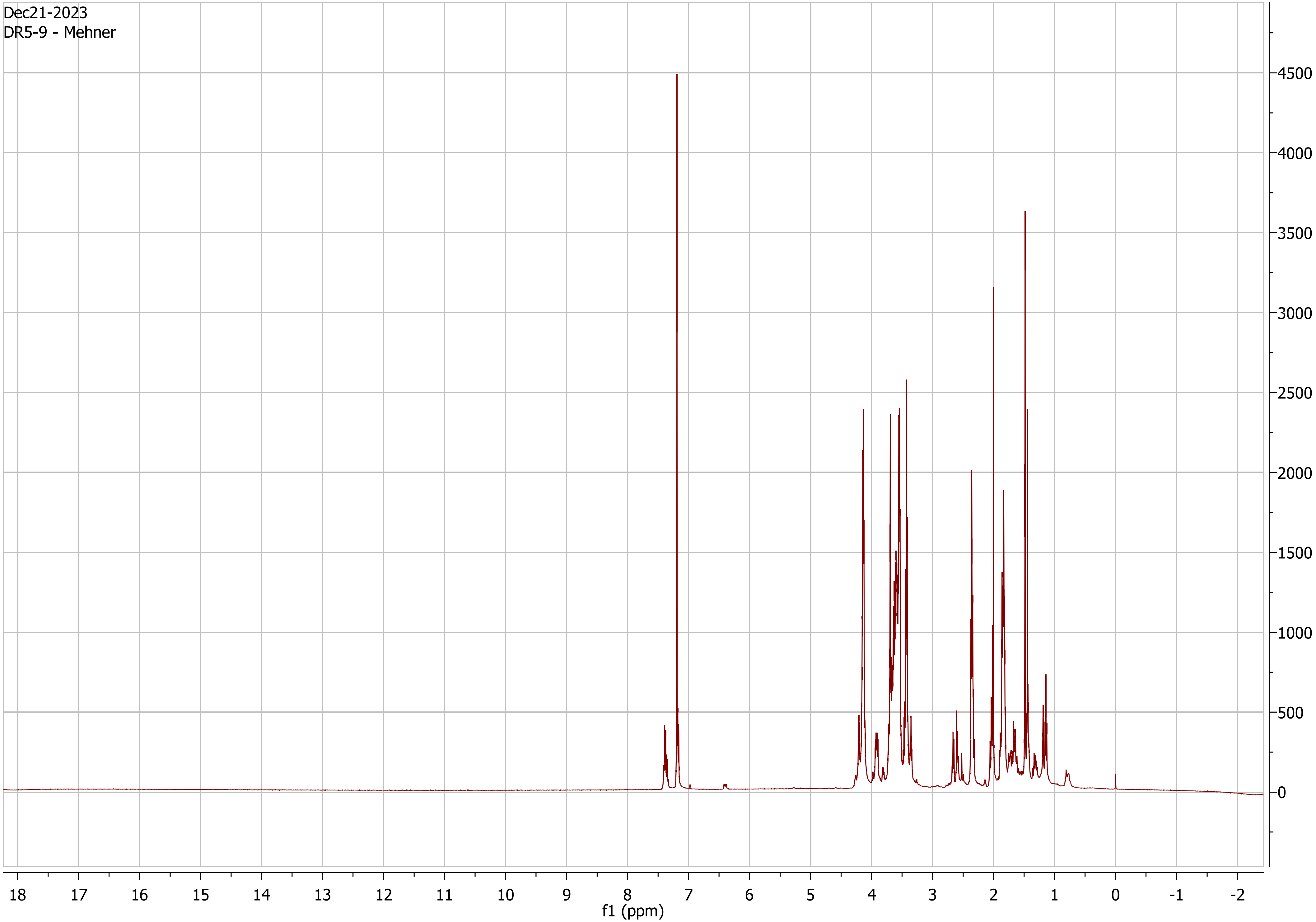

Dec21-2023
DR5-9 - Mehner
f1 (ppm)

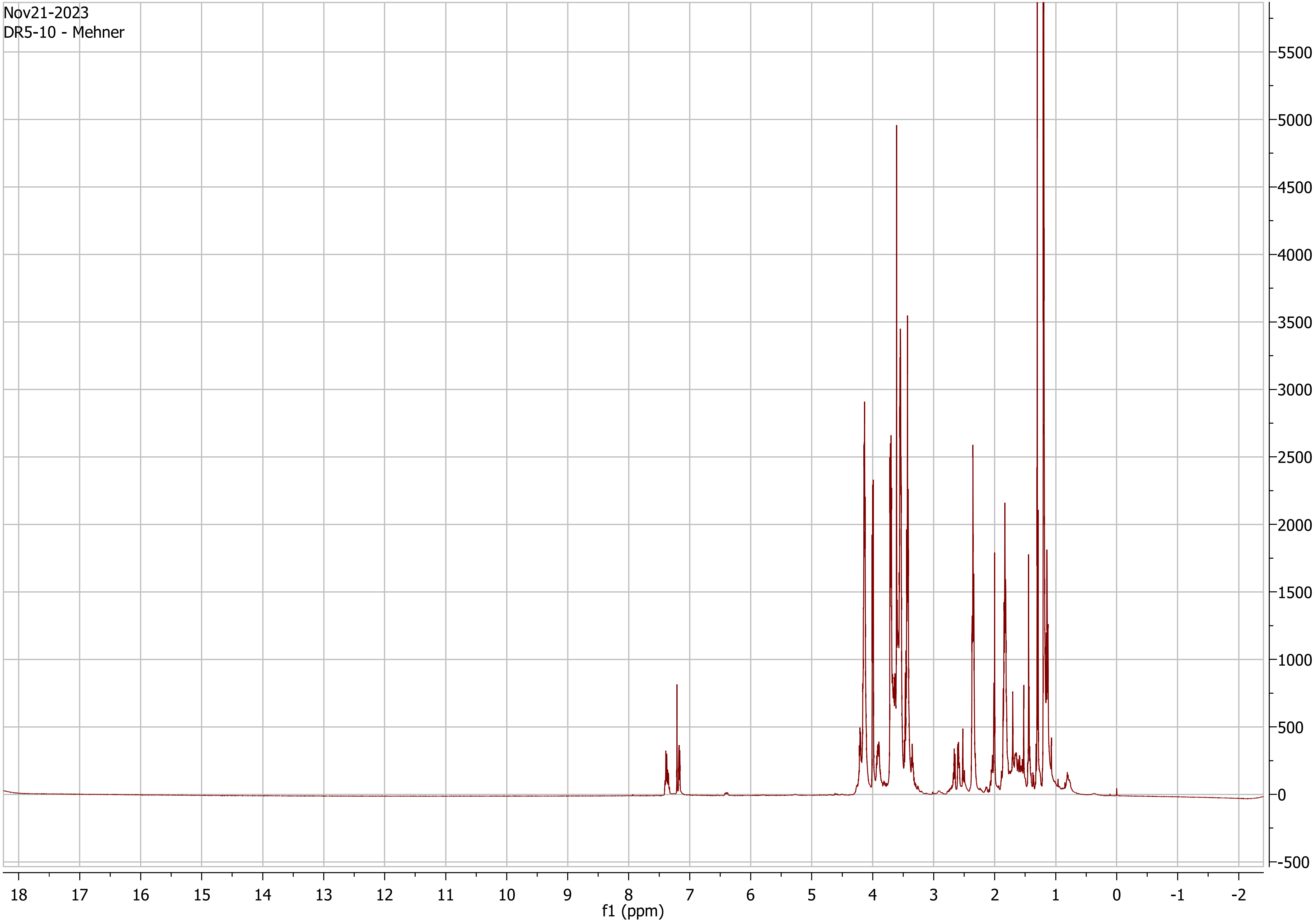

Nov21-2023
DR5-10 - Mehner
5500
5000
4500
4000
3500
3000
2500
2000
1500
1000
500
0
-500
18 17 16 15 14 13 12 11 10 9 8 7 6 5 4 3 2 1 0 -1 -2
f1 (ppm)

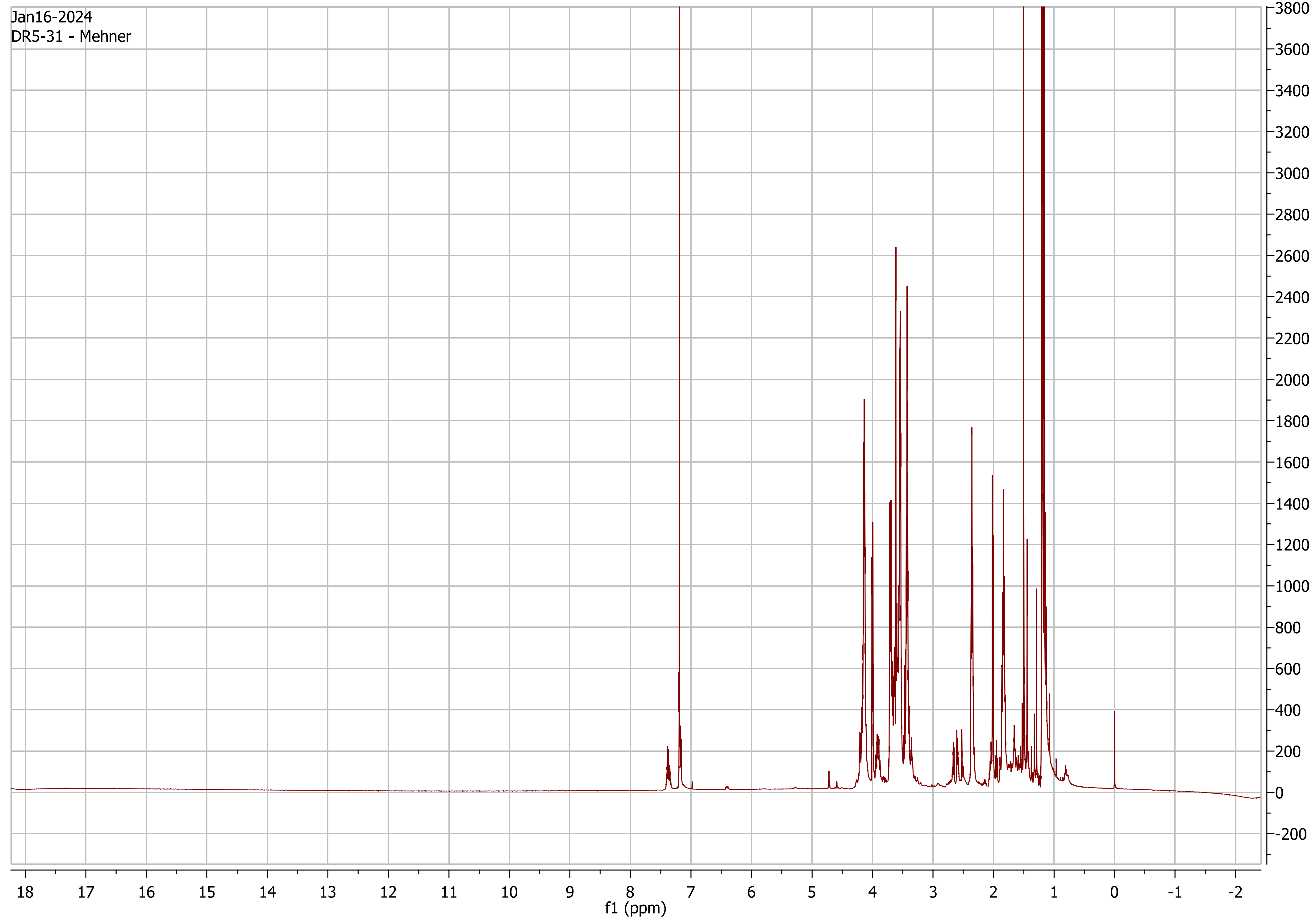

Jan16-2024
DR5-31 - Mehner
f1 (ppm)

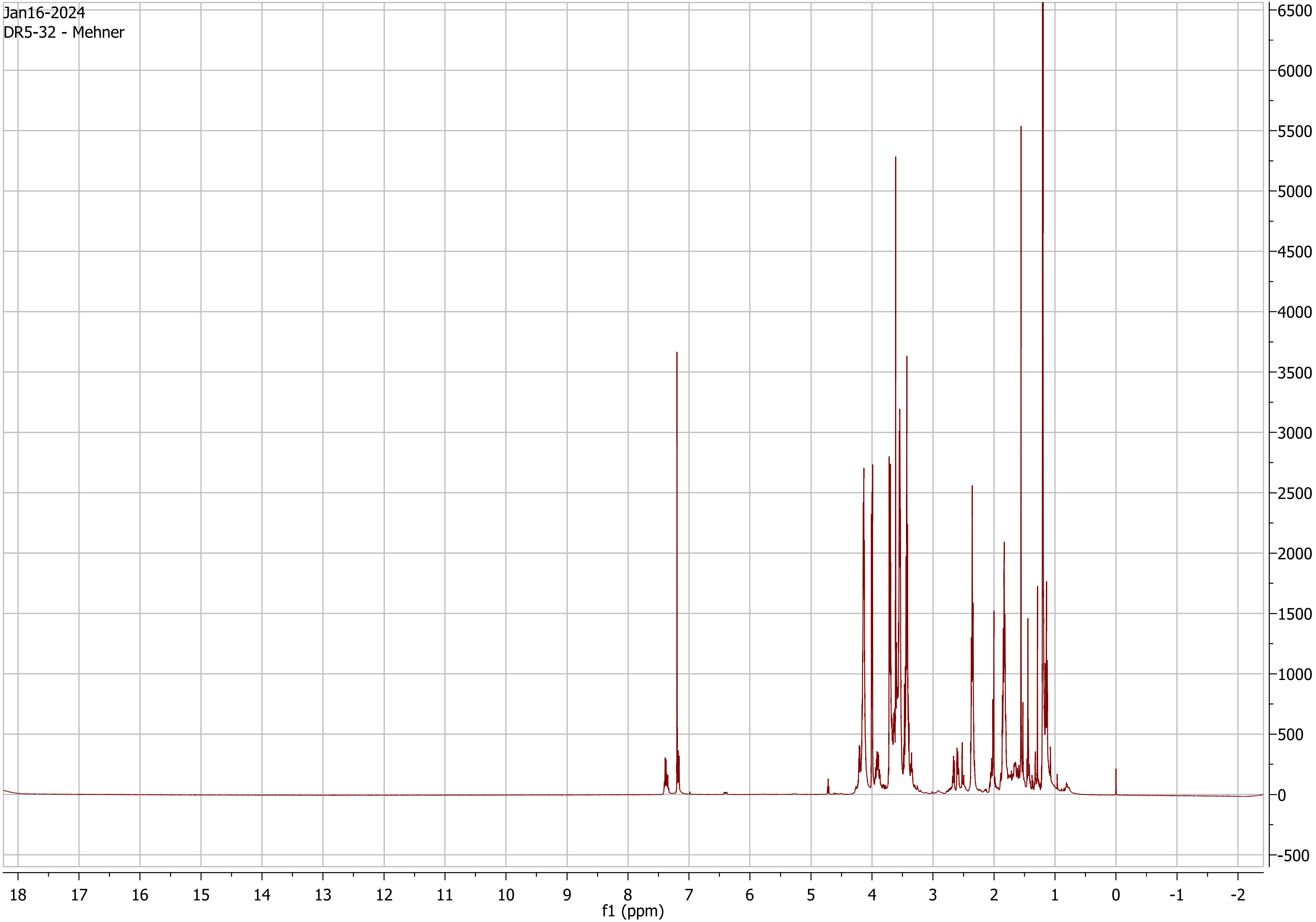

Jan16-2024
DR5-32 - Mehner
f1 (ppm)

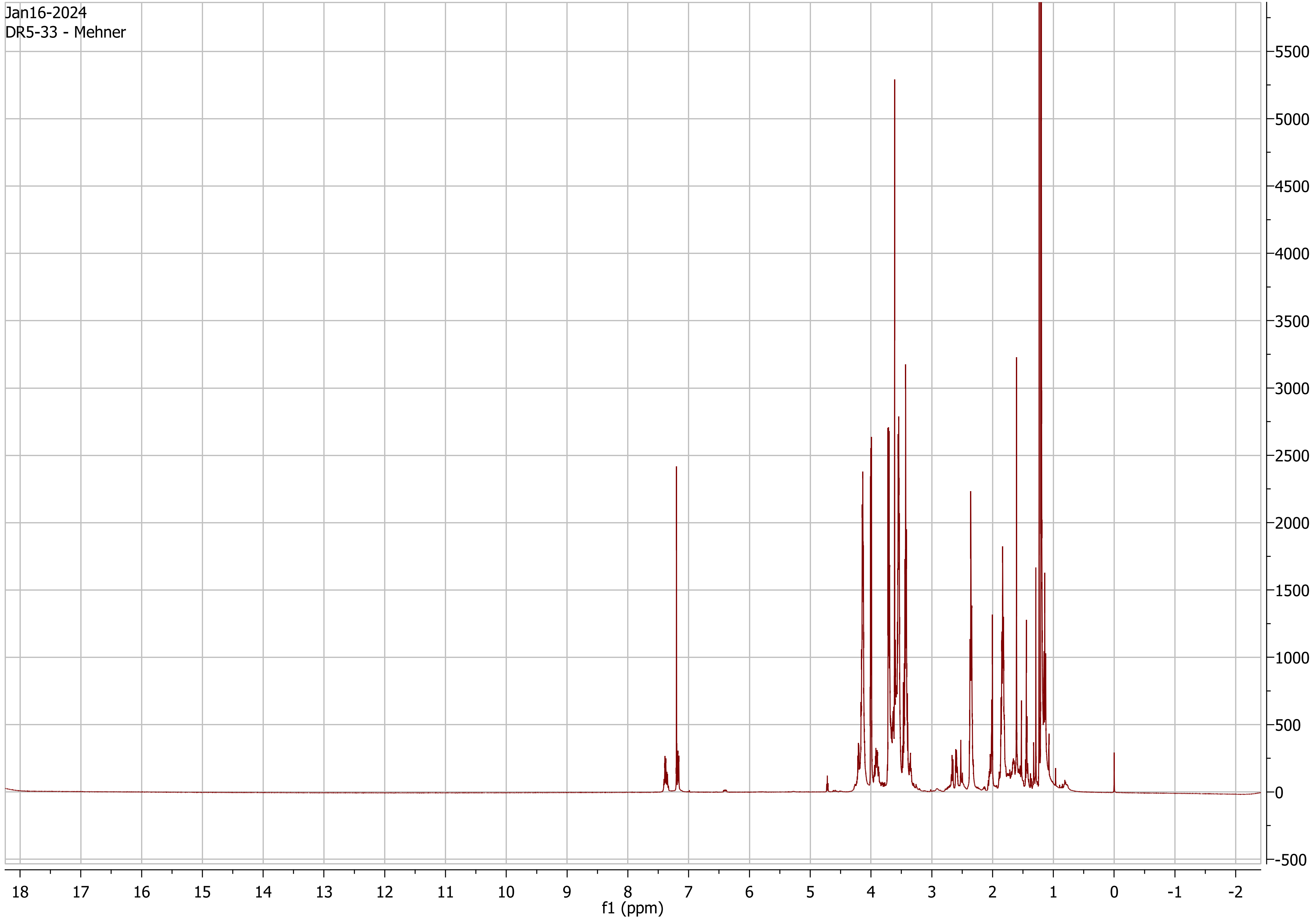

Jan16-2024
DR5-33 - Mehner
f1 (ppm)

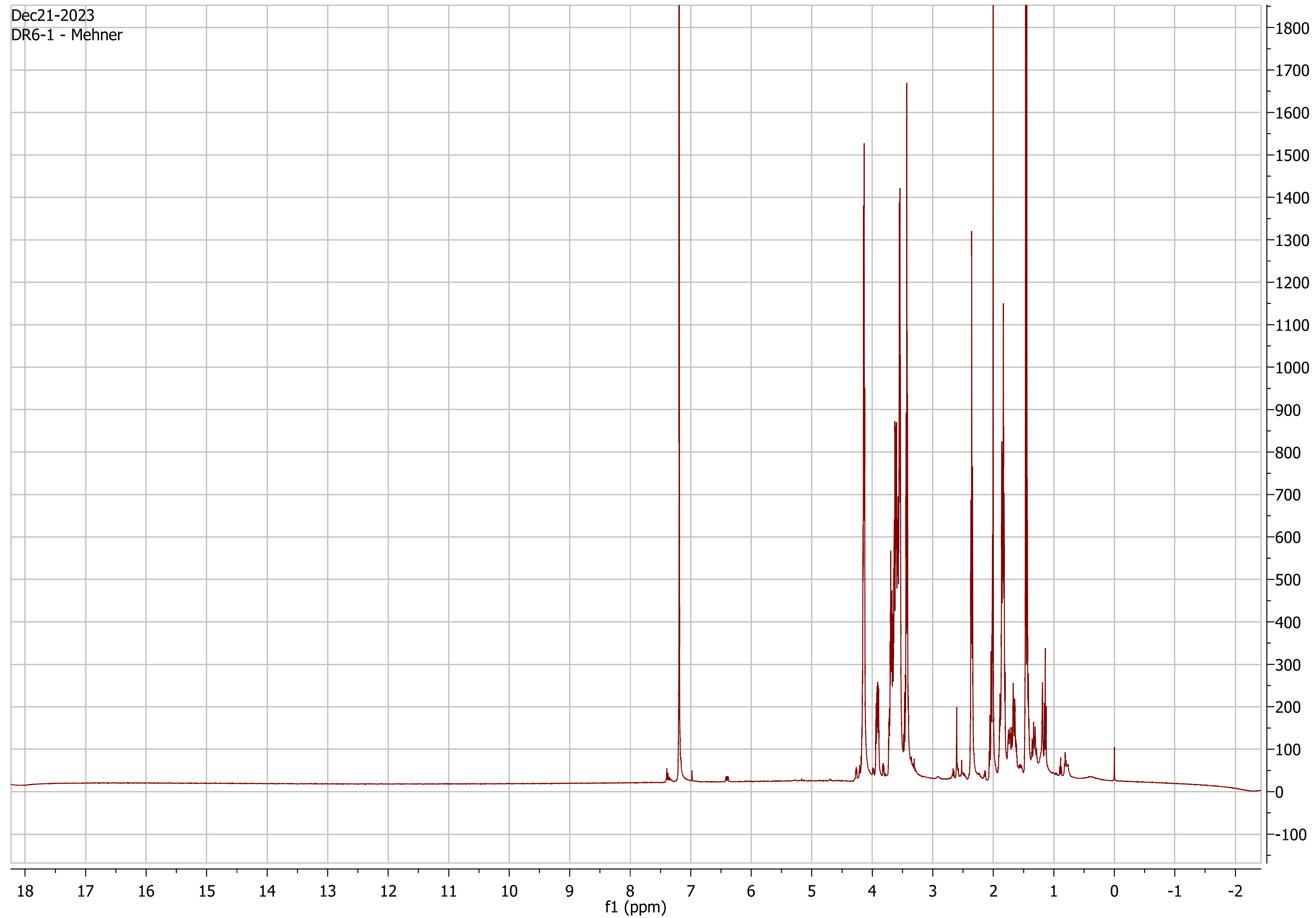

Dec21-2023
DR6-1 - Mehner
f1 (ppm)

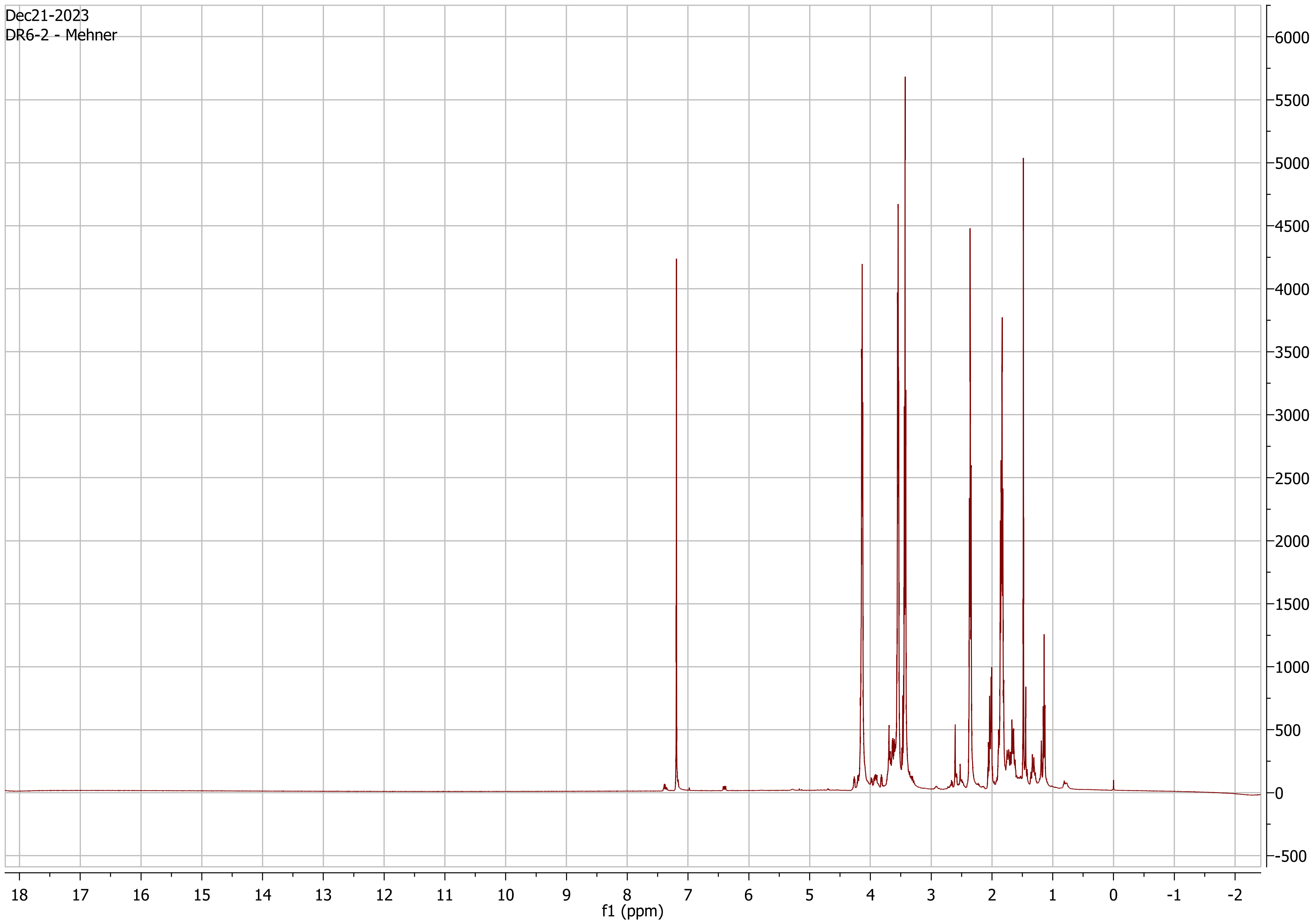

Dec21-2023
DR6-2 - Mehner
f1 (ppm)

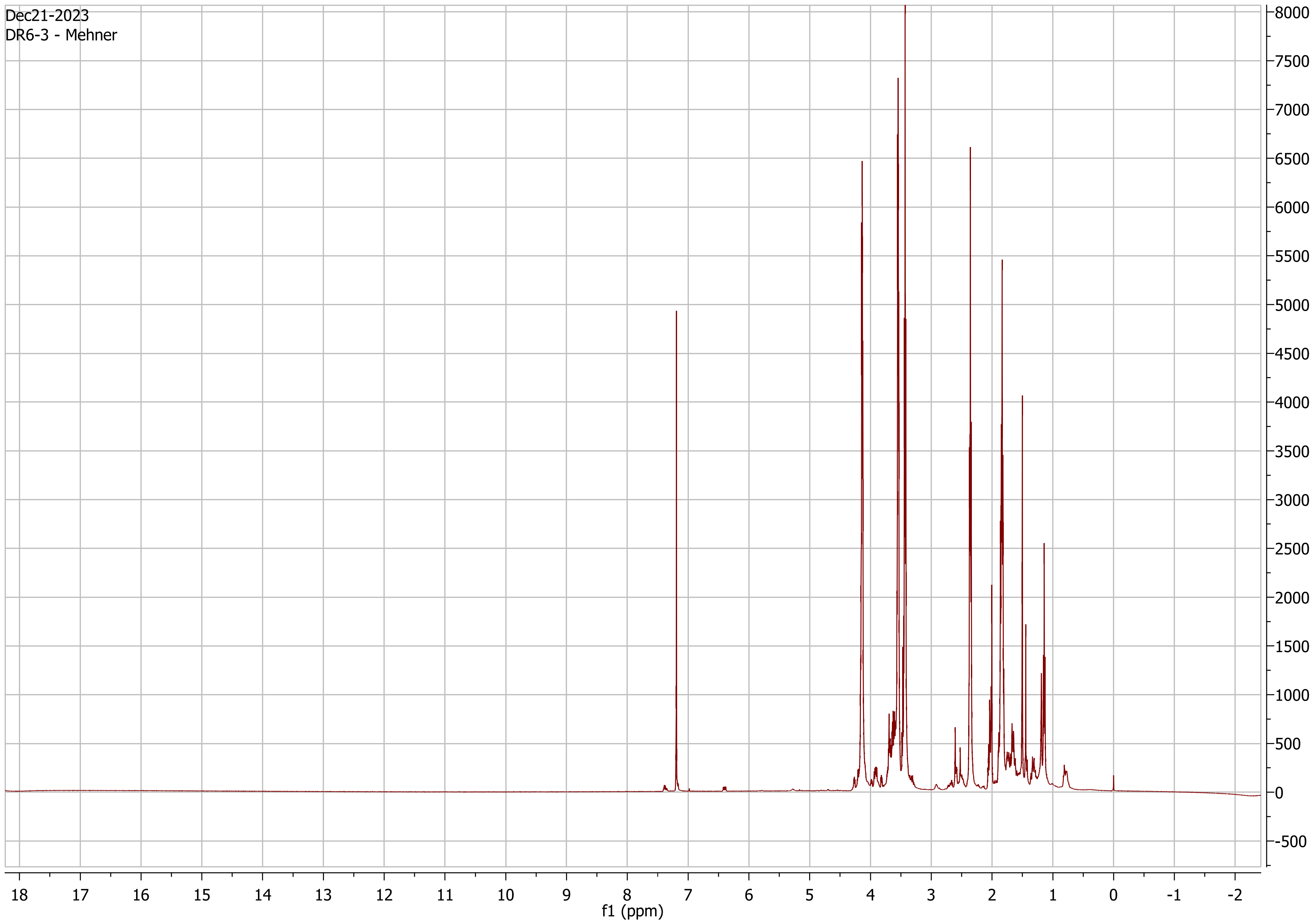
Dec21-2023
DR6-3 - Mehner
f1 (ppm)

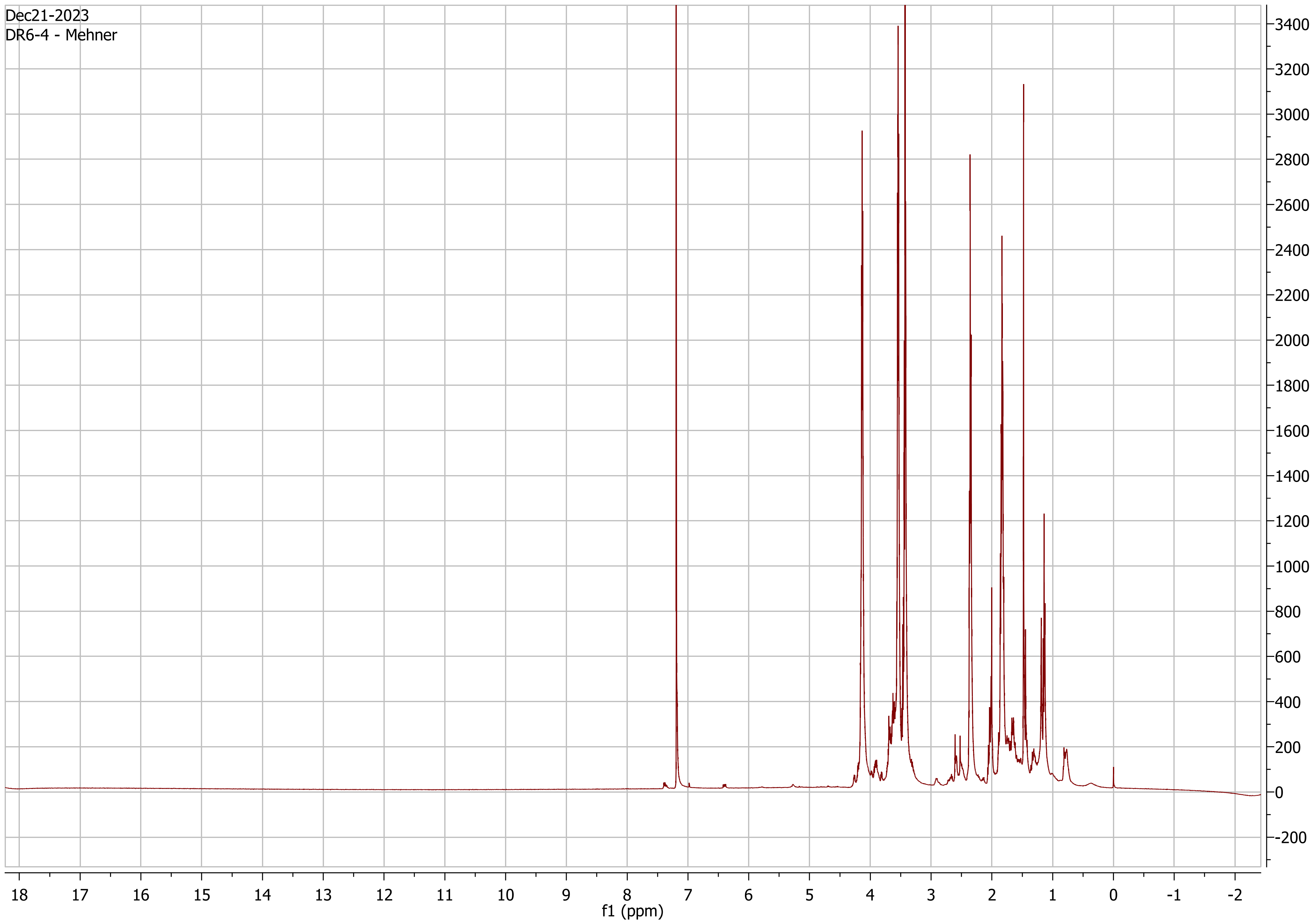

Dec21-2023
DR6-4 - Mehner
f1 (ppm)

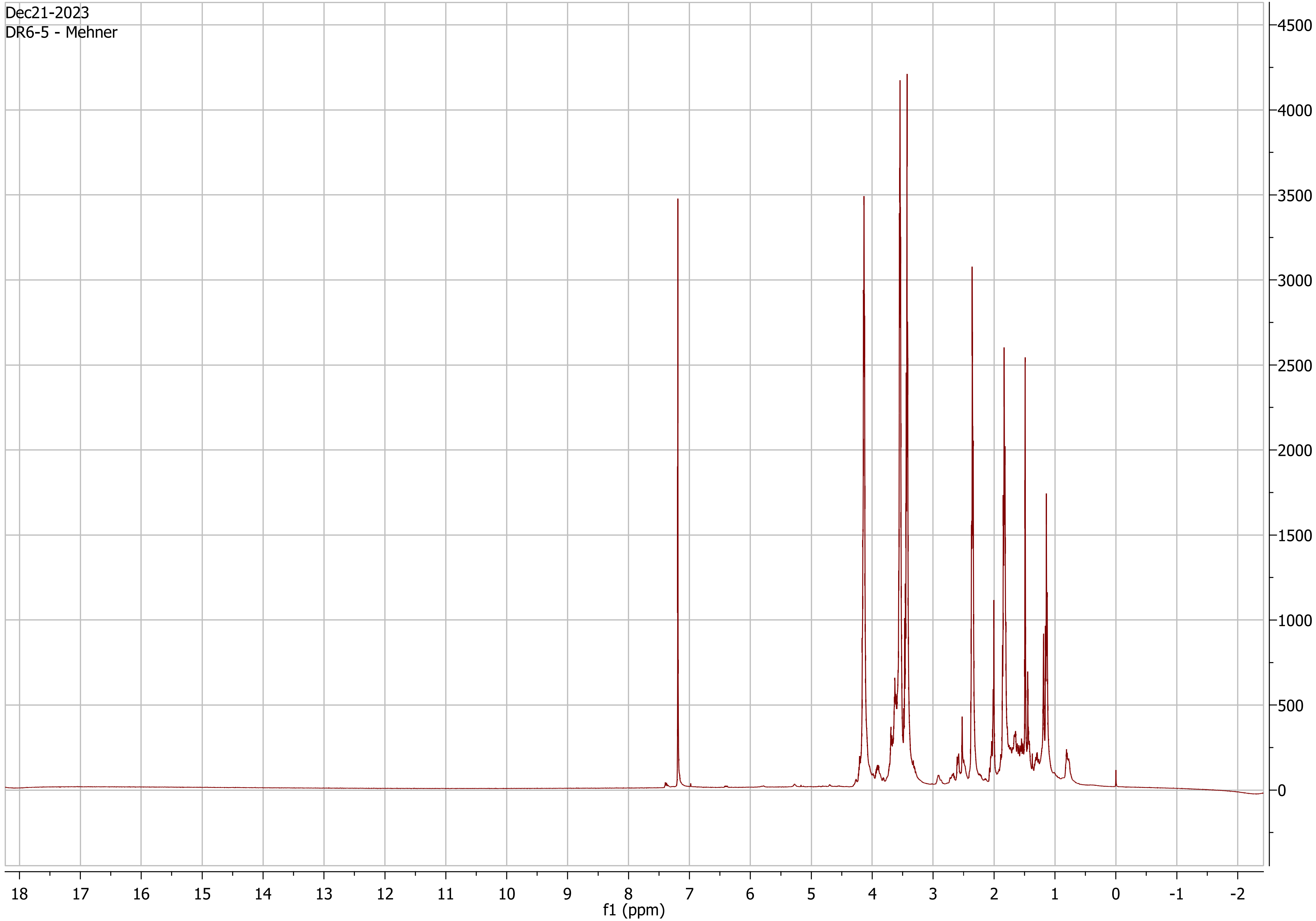

Dec21-2023
DR6-5 - Mehner
f1 (ppm)

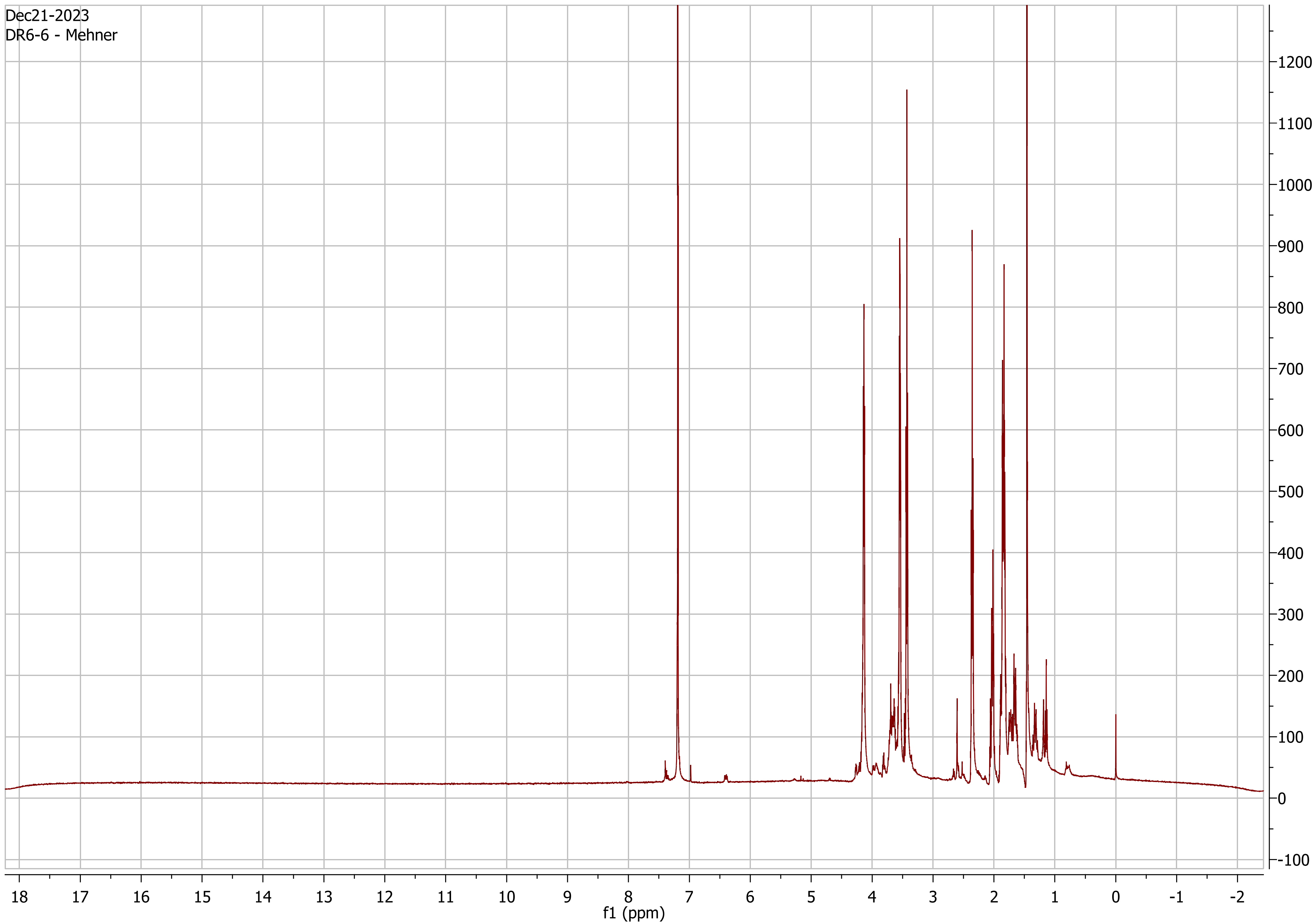
Dec21-2023
DR6-6 - Mehner
f1 (ppm)

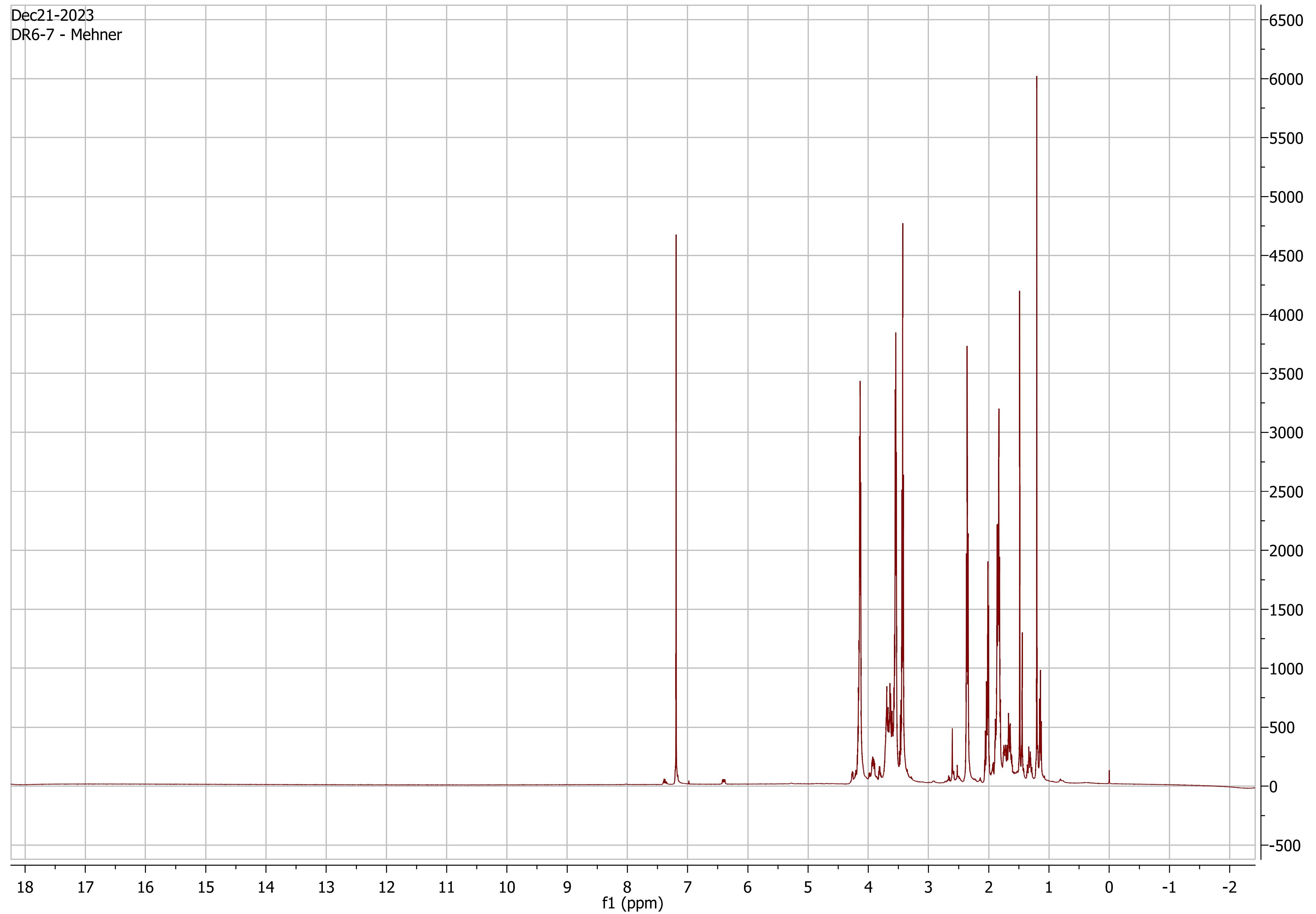
Dec21-2023
DR6-7 - Mehner
f1 (ppm)
18
17
16
15
14
13
12
11
10
9
8
7
6
5
4
3
2
1
0
-1
-2
6500
6000
5500
5000
4500
4000
3500
3000
2500
2000
1500
1000
500
0
-500

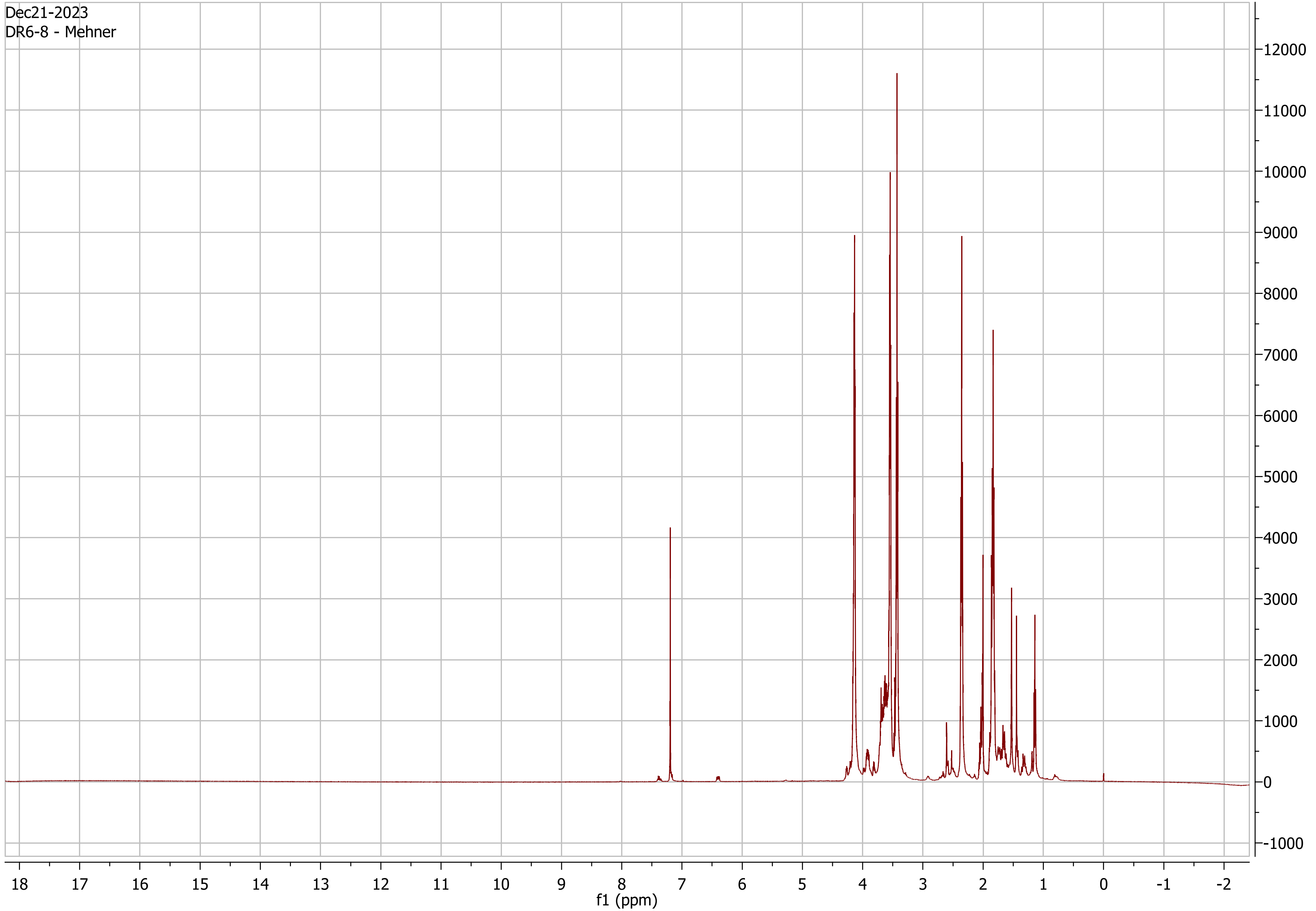

Dec21-2023
DR6-8 - Mehner
f1 (ppm)

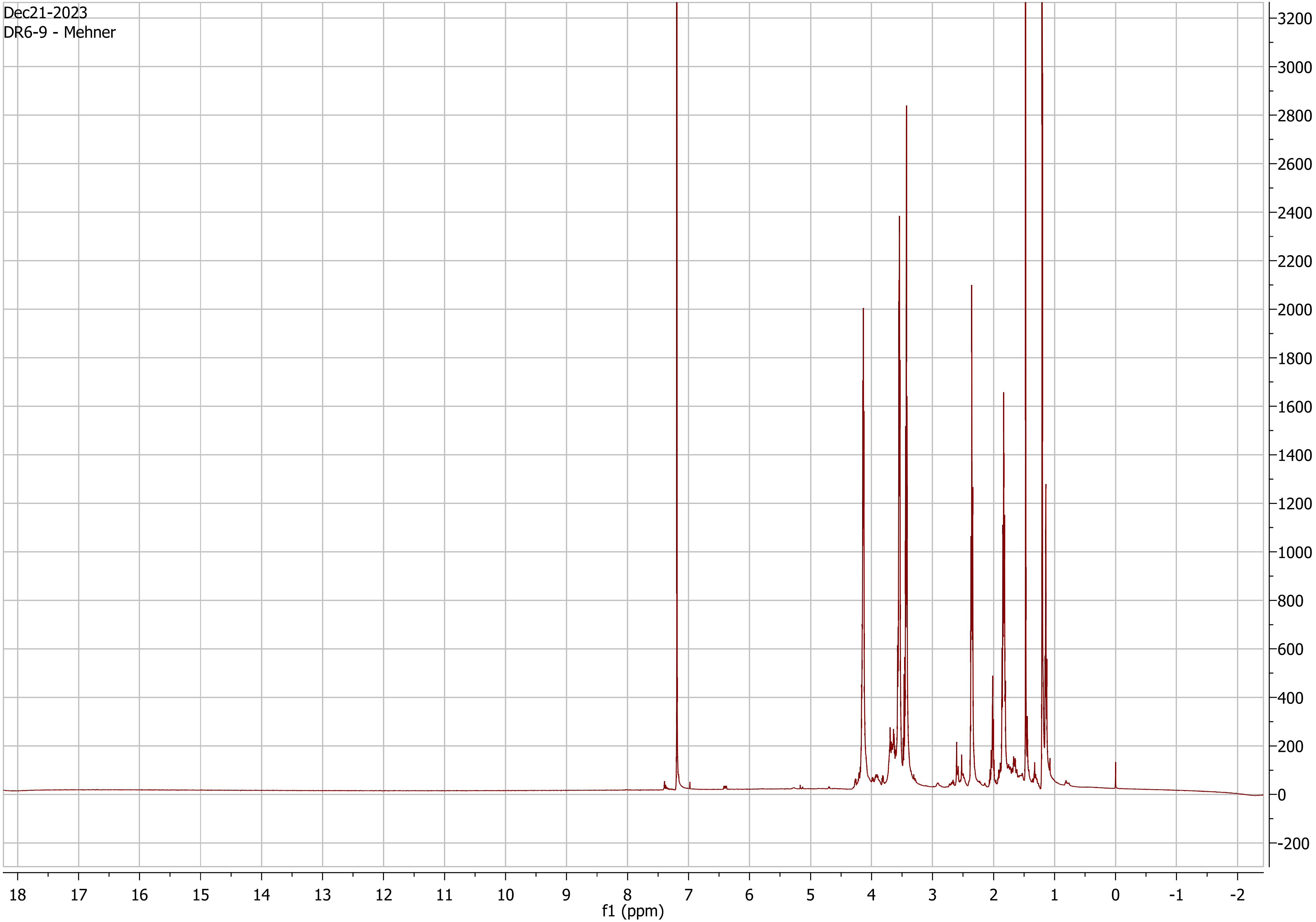

Dec21-2023
DR6-9 - Mehner
f1 (ppm)

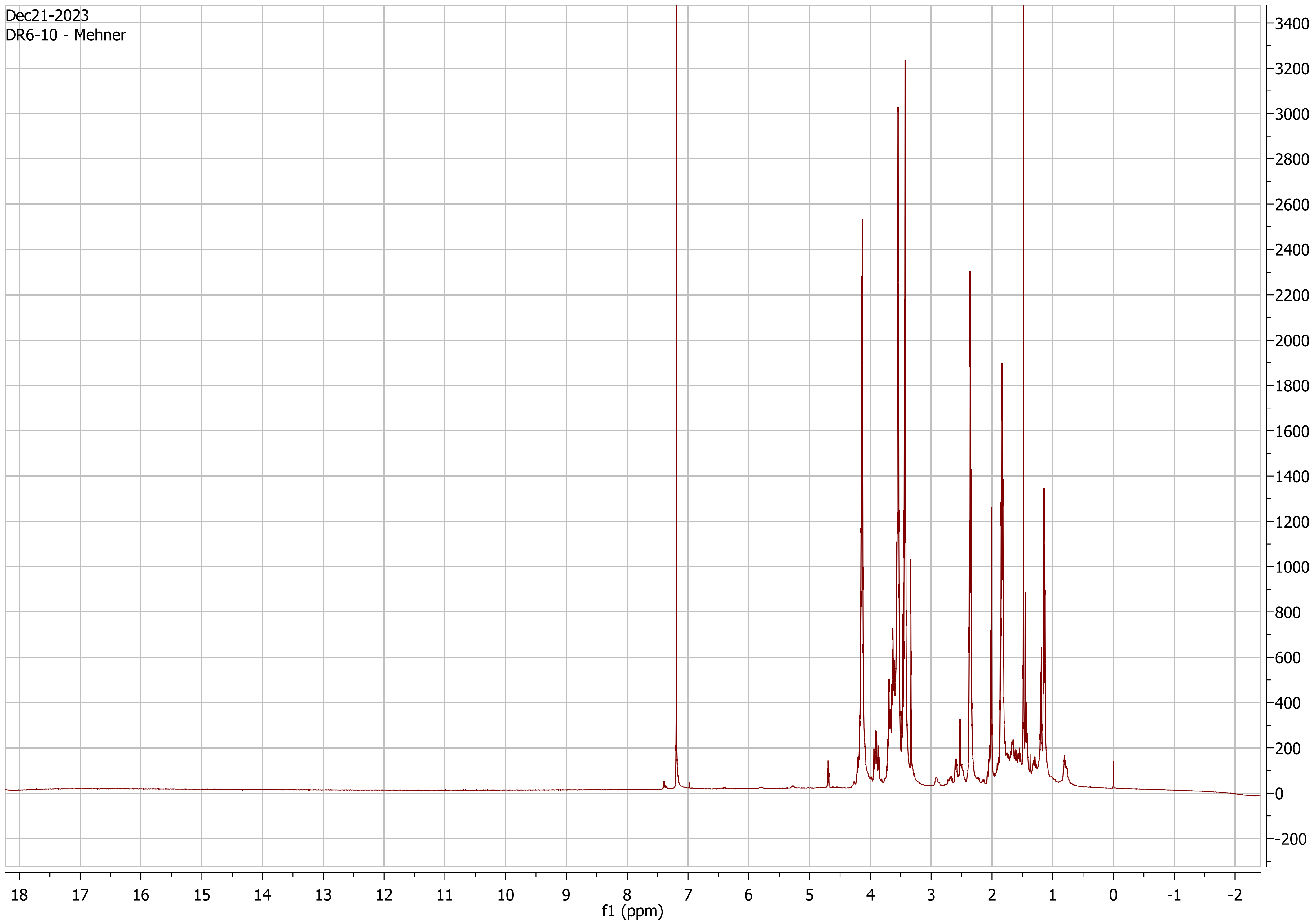
Dec21-2023
DR6-10 - Mehner
f1 (ppm)

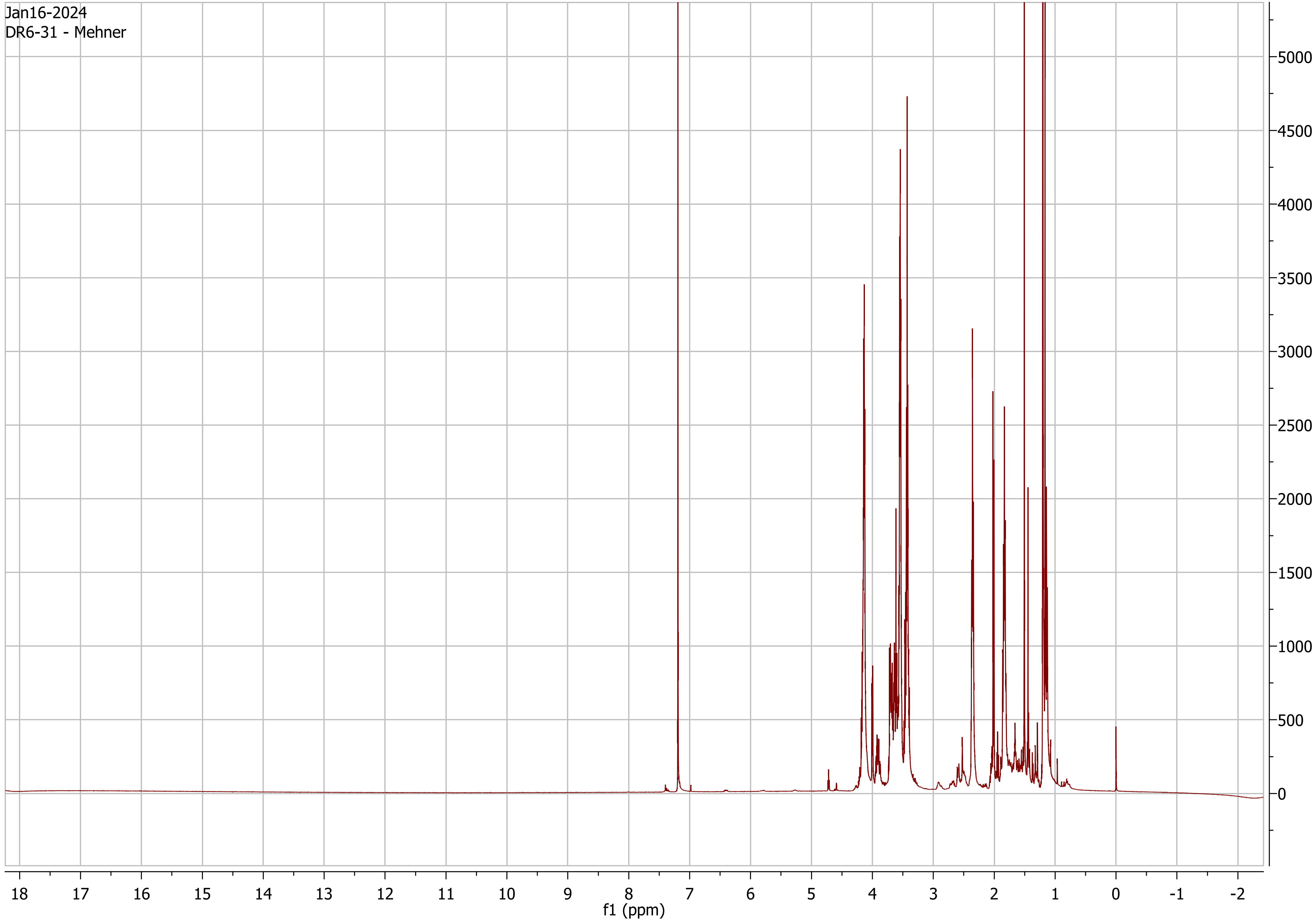
Jan16-2024
DR6-31 - Mehner
f1 (ppm)

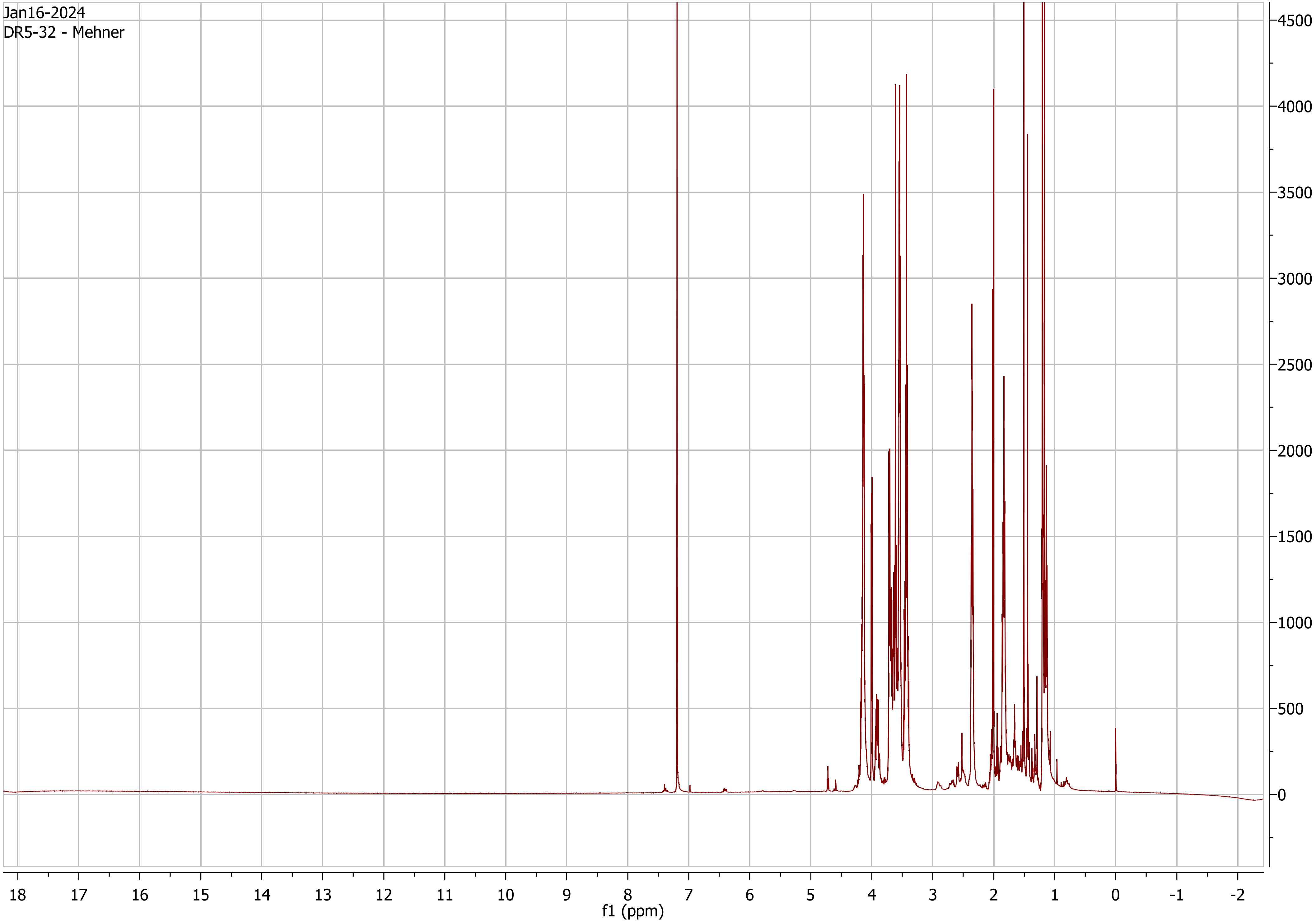
Jan16-2024
DR5-32 - Mehner
f1 (ppm)

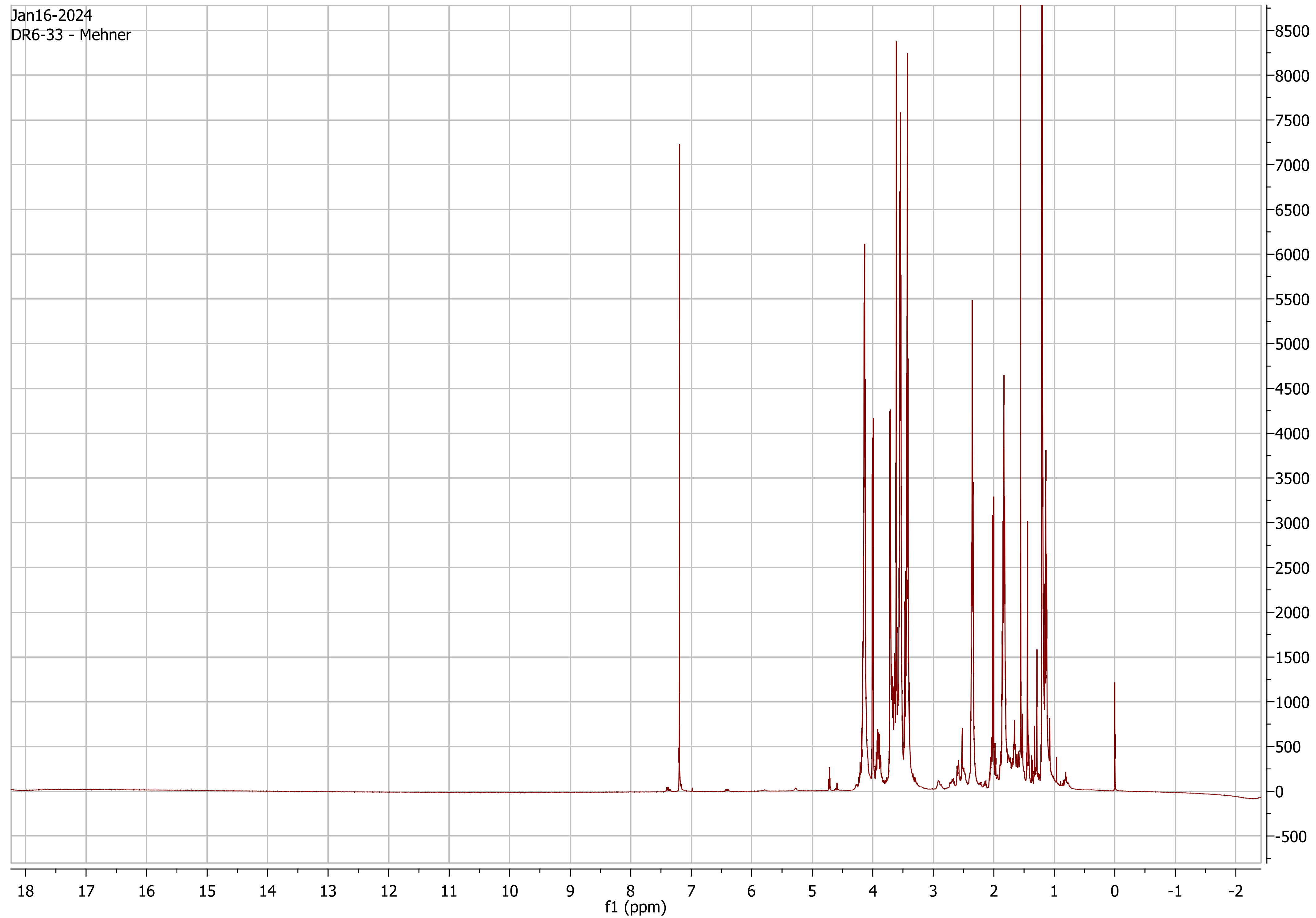
Jan16-2024
DR6-33 - Mehner
f1 (ppm)

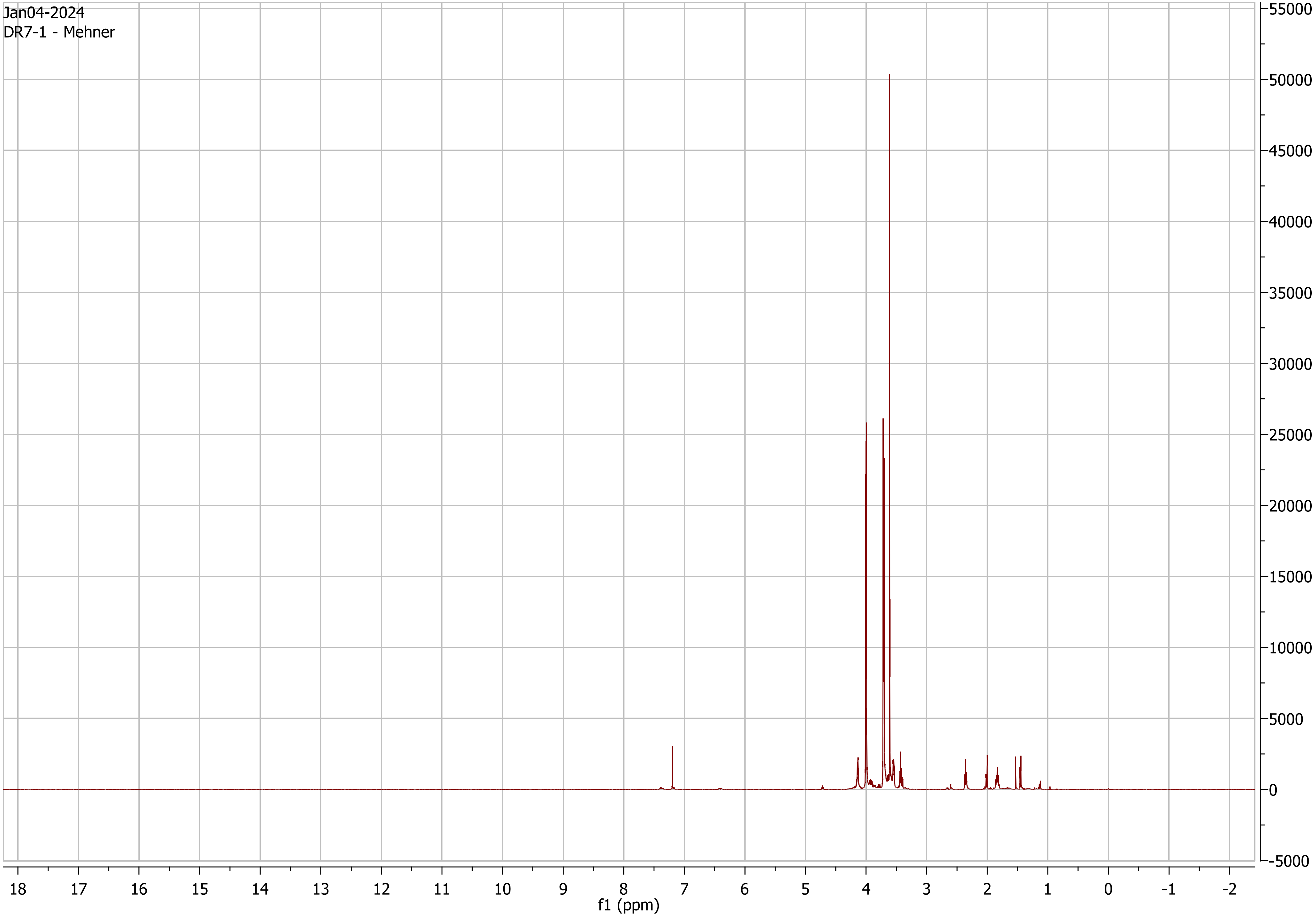
Jan04-2024
DR7-1 - Mehner
f1 (ppm)

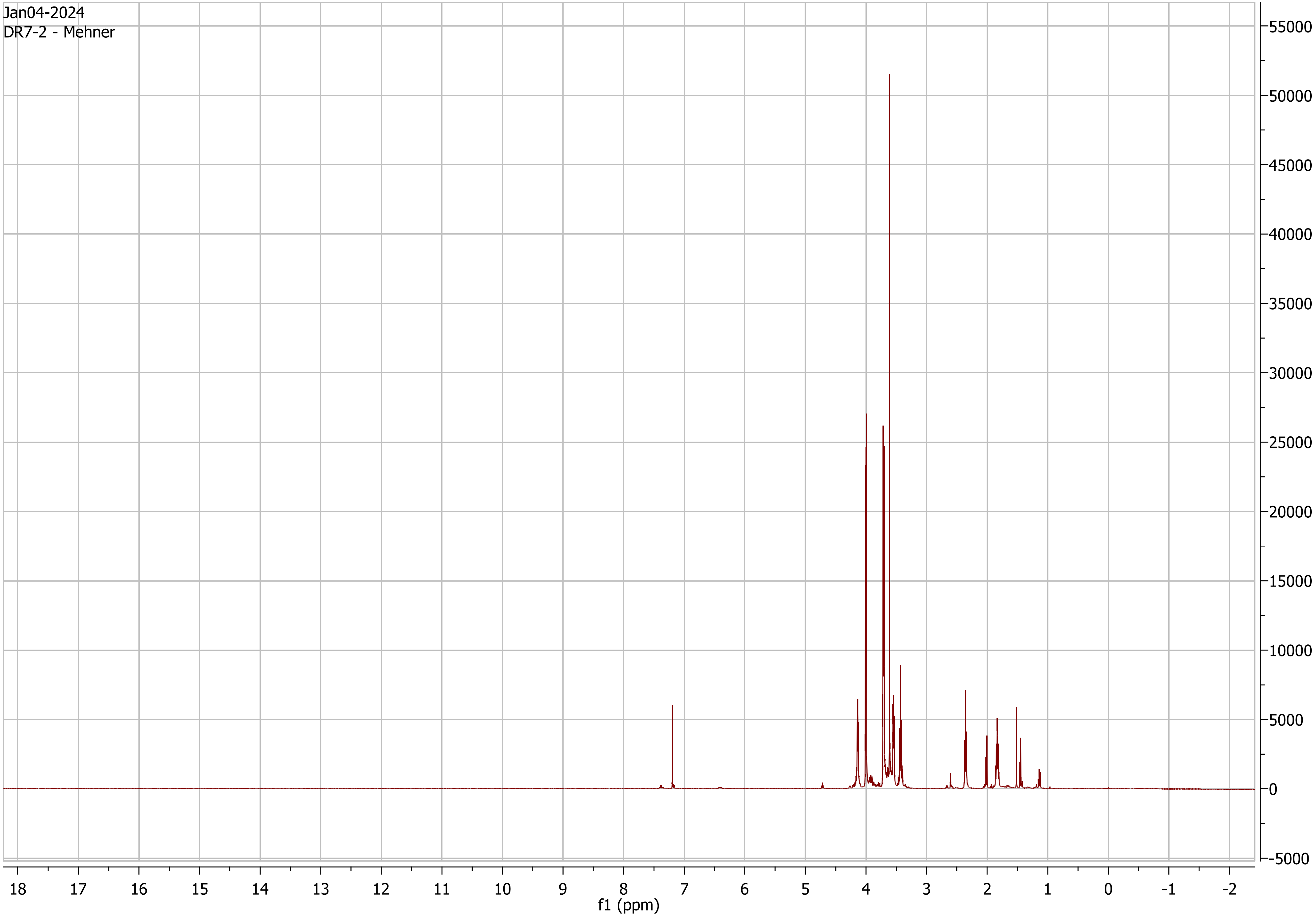

Jan04-2024
DR7-2 - Mehner
f1 (ppm)

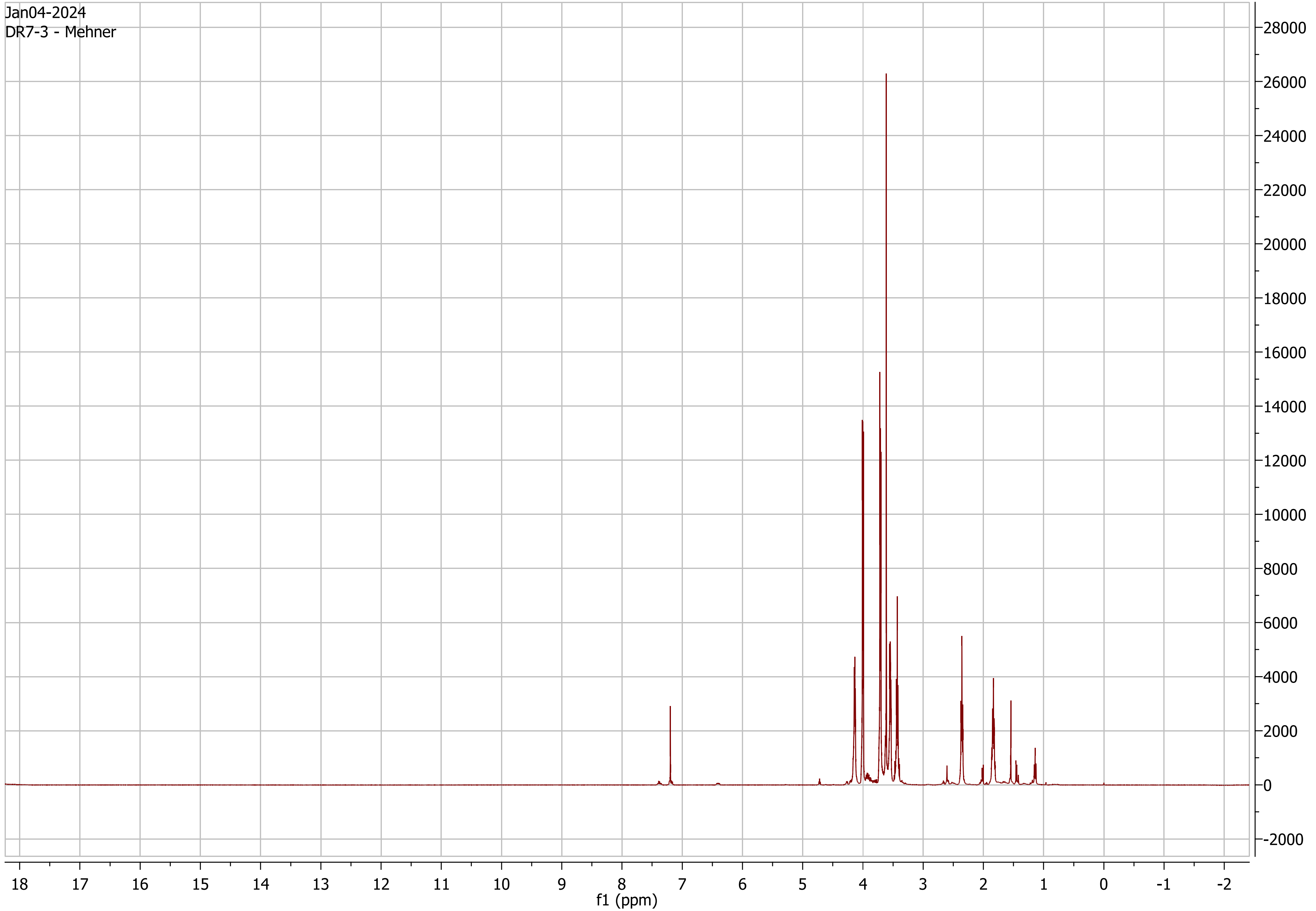

Jan04-2024
DR7-3 - Mehner
28000
26000
24000
22000
20000
18000
16000
14000
12000
10000
8000
6000
4000
2000
0
-2000
18
17
16
15
14
13
12
11
10
9
8
7
6
5
4
3
2
1
0
-1
-2
f1 (ppm)

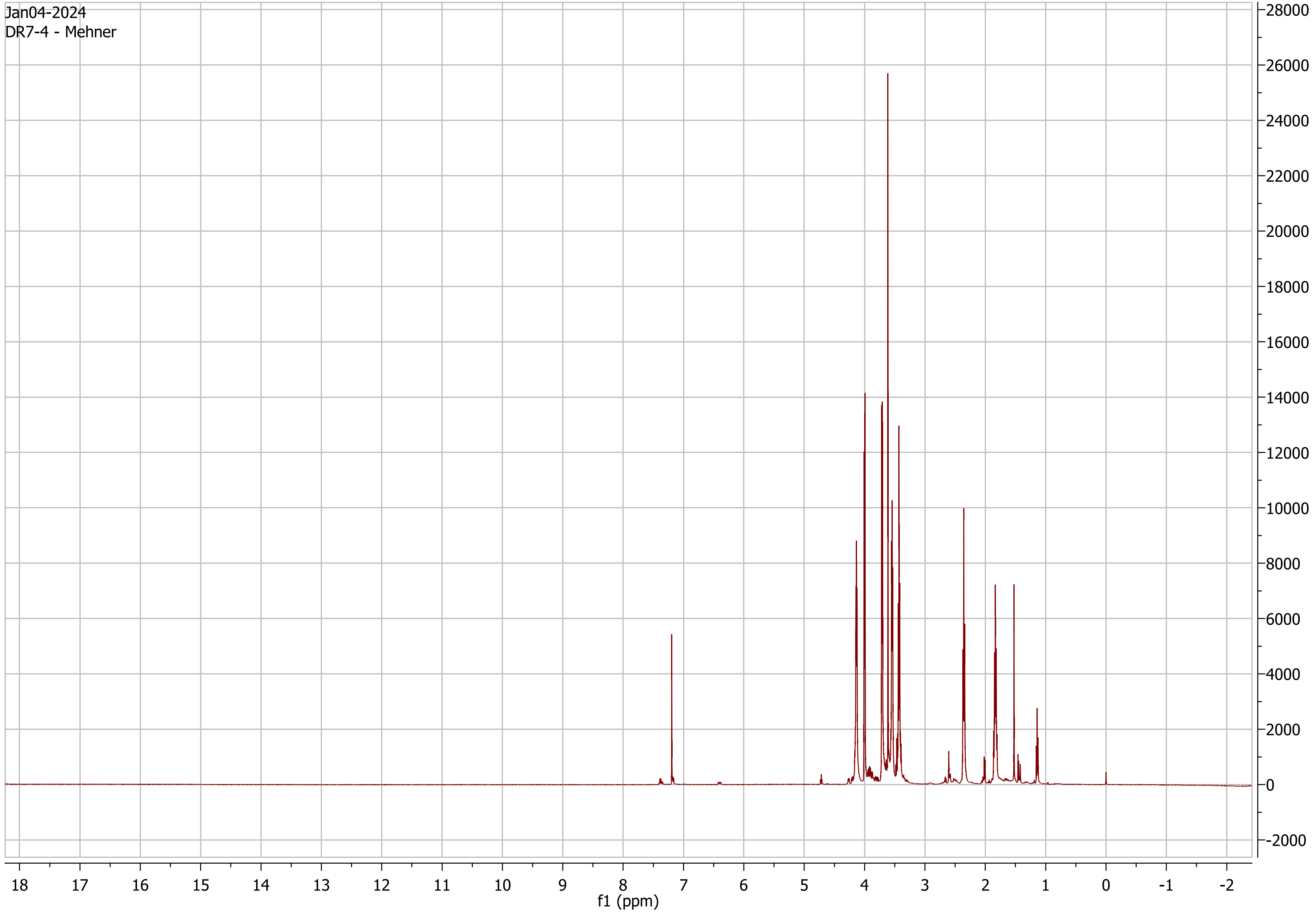

Jan04-2024
DR7-4 - Mehner
28000
26000
24000
22000
20000
18000
16000
14000
12000
10000
8000
6000
4000
2000
0
-2000
18
17
16
15
14
13
12
11
10
9
8
7
6
5
4
3
2
1
0
-1
-2
f1 (ppm)

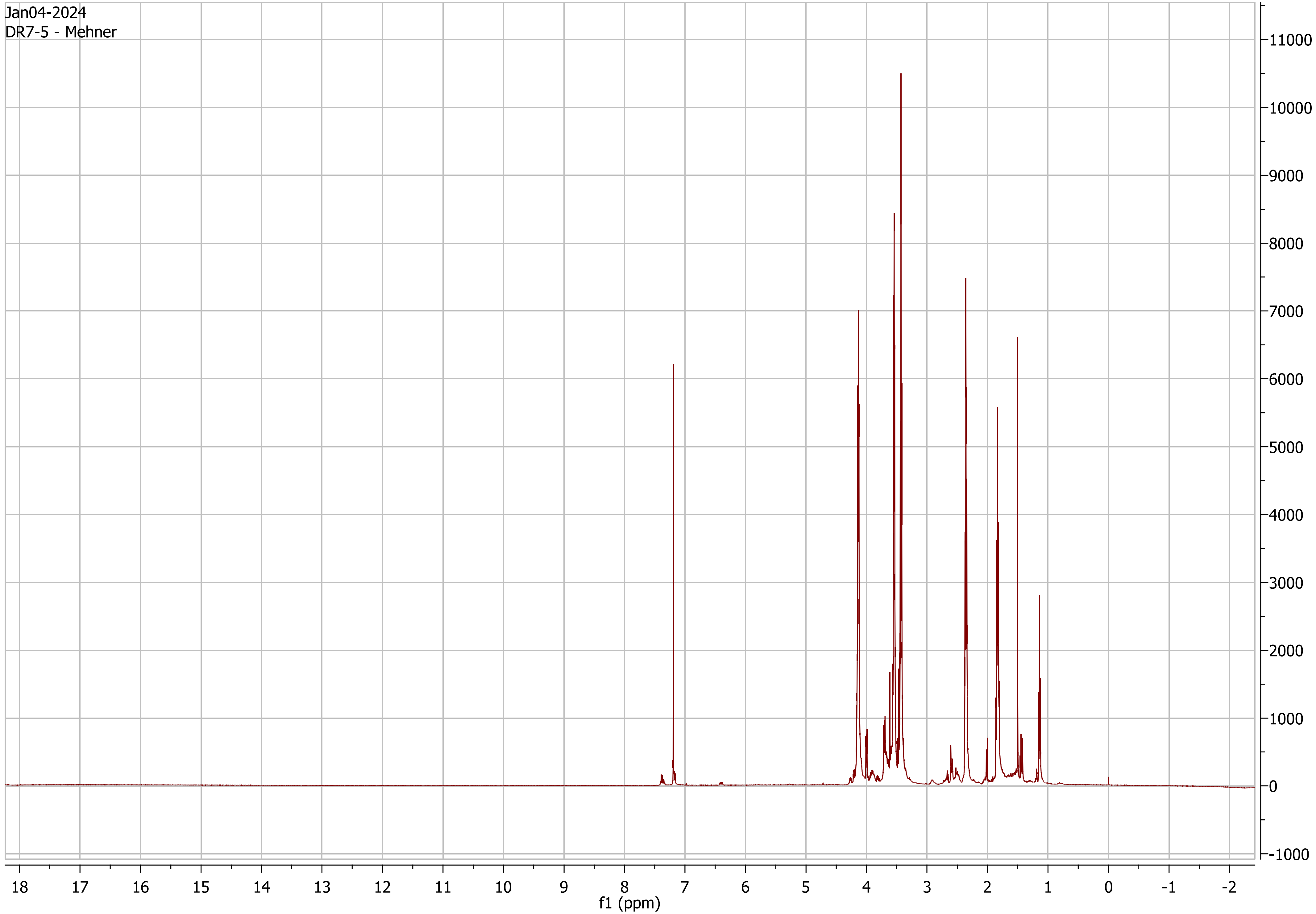

Jan04-2024
DR7-5 - Mehner
f1 (ppm)

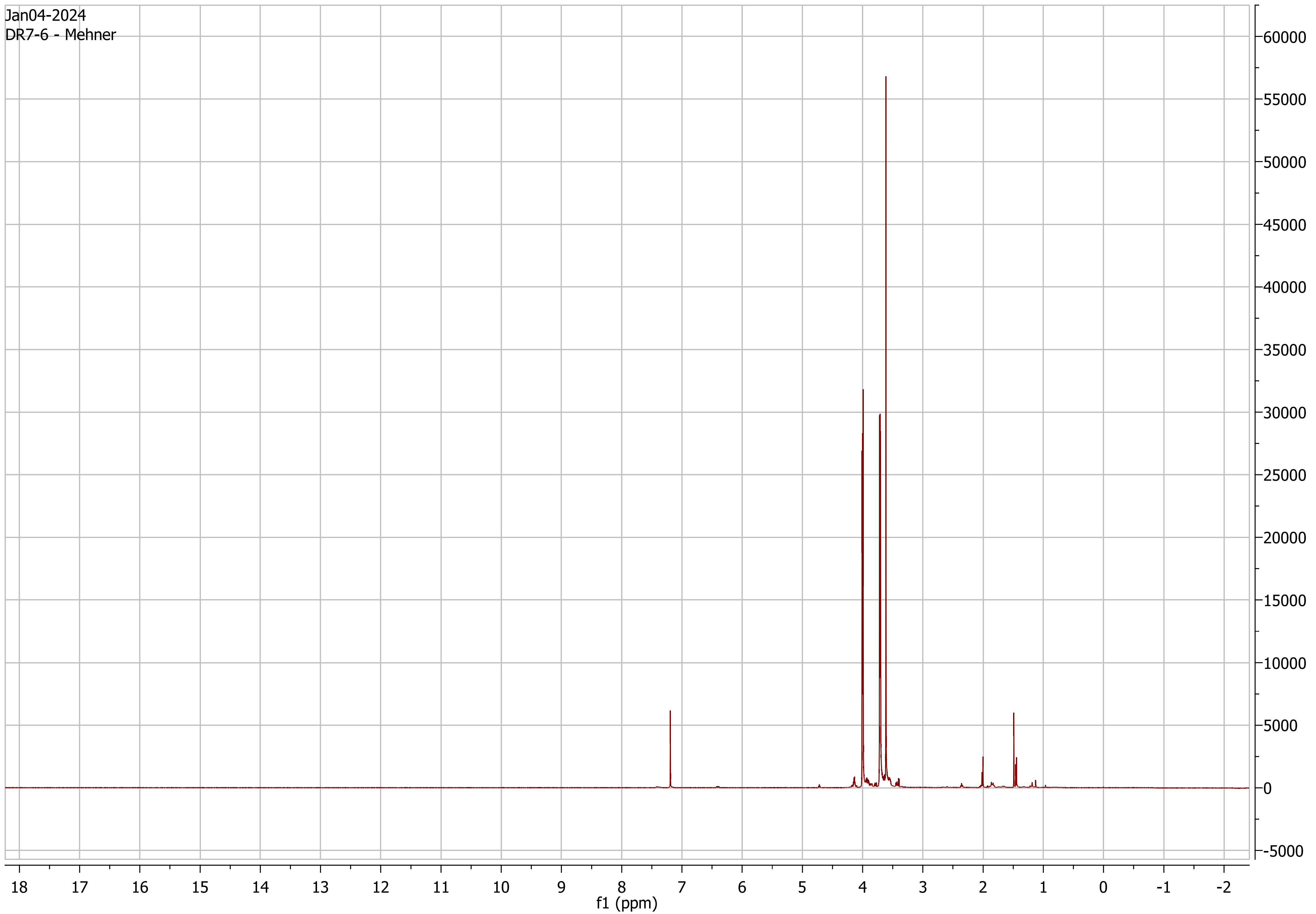

Jan04-2024
DR7-6 - Mehner
60000
55000
50000
45000
40000
35000
30000
25000
20000
15000
10000
5000
0
-5000
18
17
16
15
14
13
12
11
10
9
8
7
6
5
4
3
2
1
0
-1
-2
f1 (ppm)

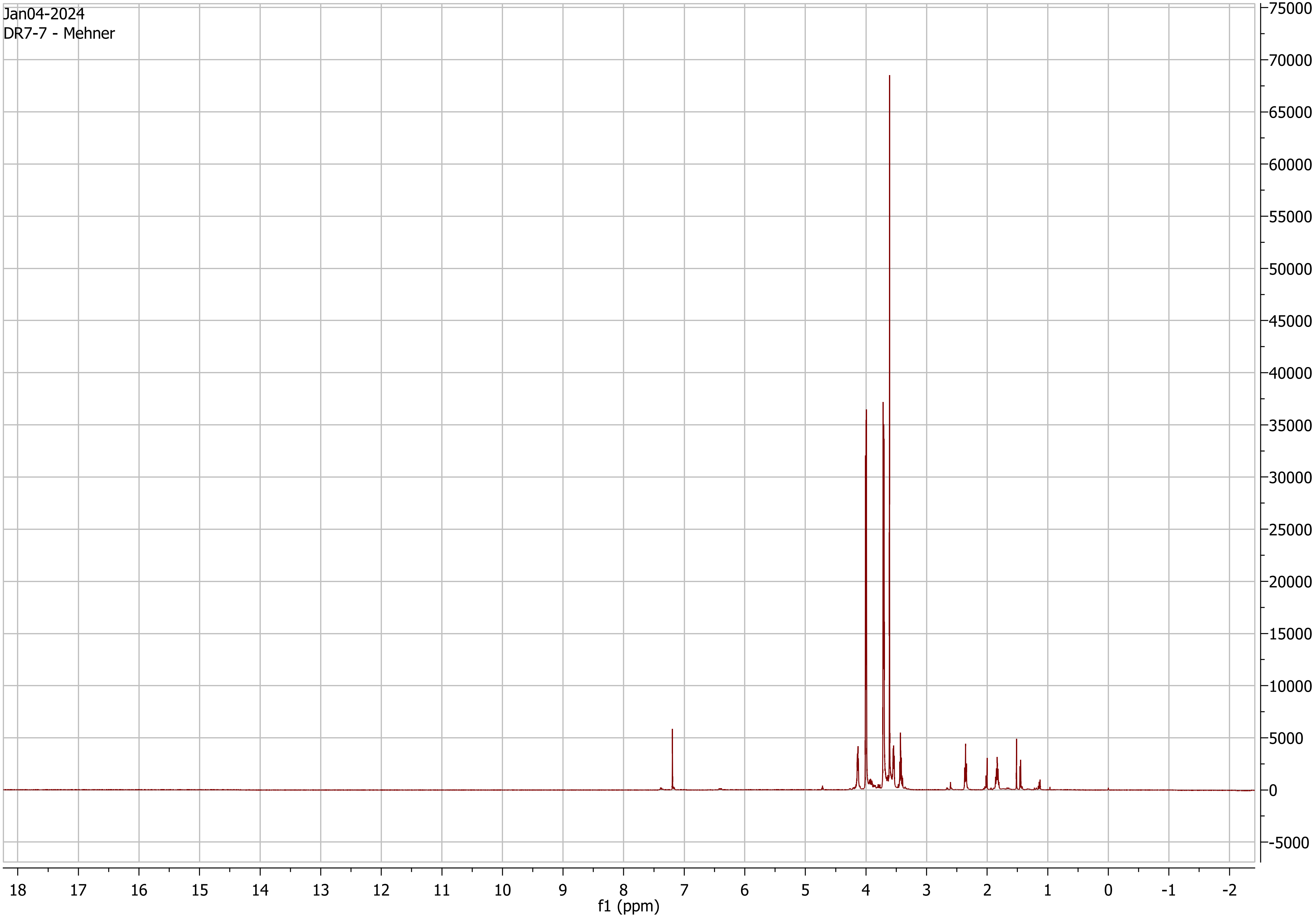

Jan04-2024
DR7-7 - Mehner
75000
70000
65000
60000
55000
50000
45000
40000
35000
30000
25000
20000
15000
10000
5000
0
-5000
18
17
16
15
14
13
12
11
10
9
8
7
6
5
4
3
2
1
0
-1
-2
f1 (ppm)

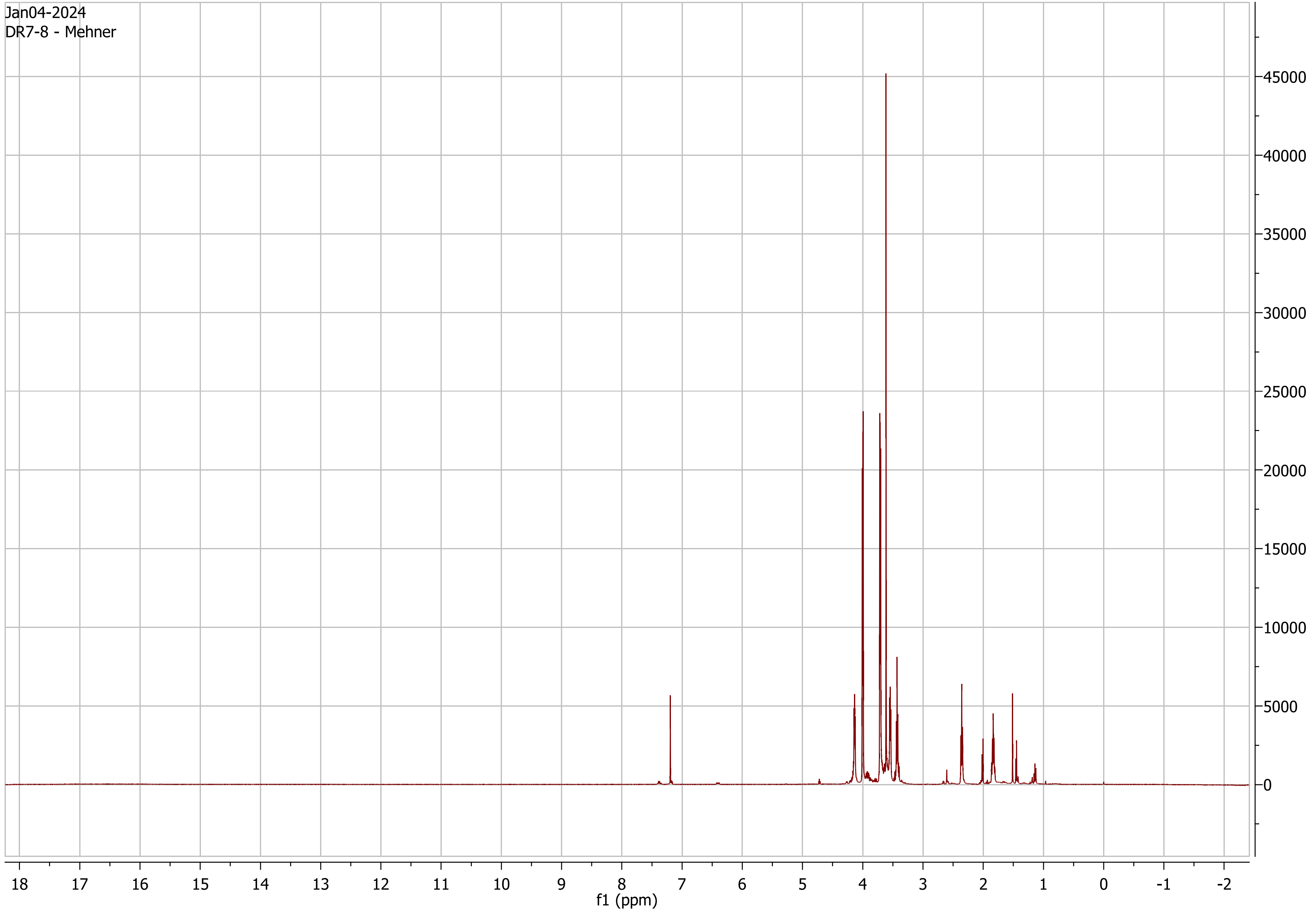
Jan04-2024
DR7-8 - Mehner
45000
40000
35000
30000
25000
20000
15000
10000
5000
0
18 17 16 15 14 13 12 11 10 9 8 7 6 5 4 3 2 1 0 -1 -2
f1 (ppm)

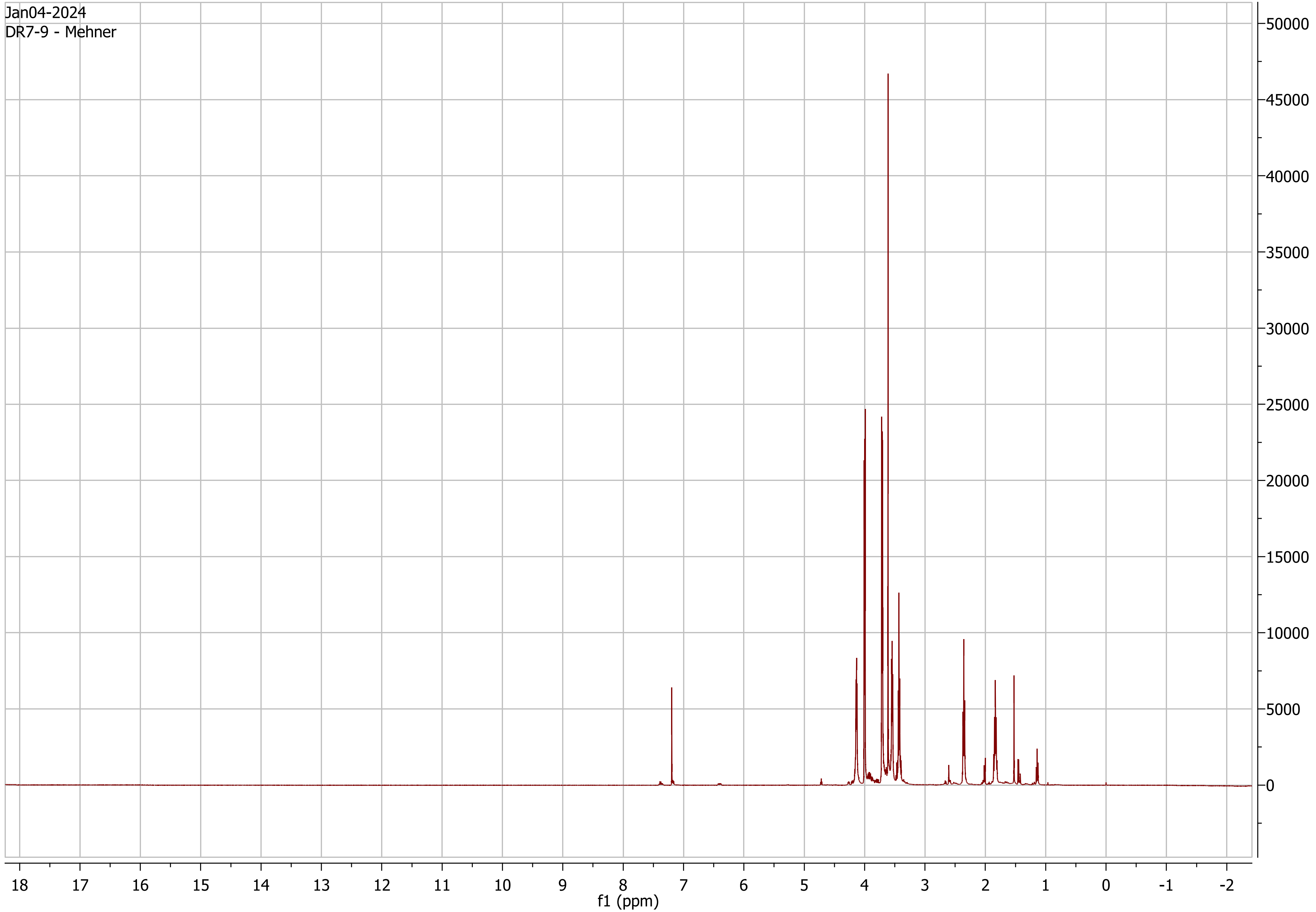
Jan04-2024
DR7-9 - Mehner
f1 (ppm)
18
17
16
15
14
13
12
11
10
9
8
7
6
5
4
3
2
1
0
-1
-2
50000
45000
40000
35000
30000
25000
20000
15000
10000
5000
0

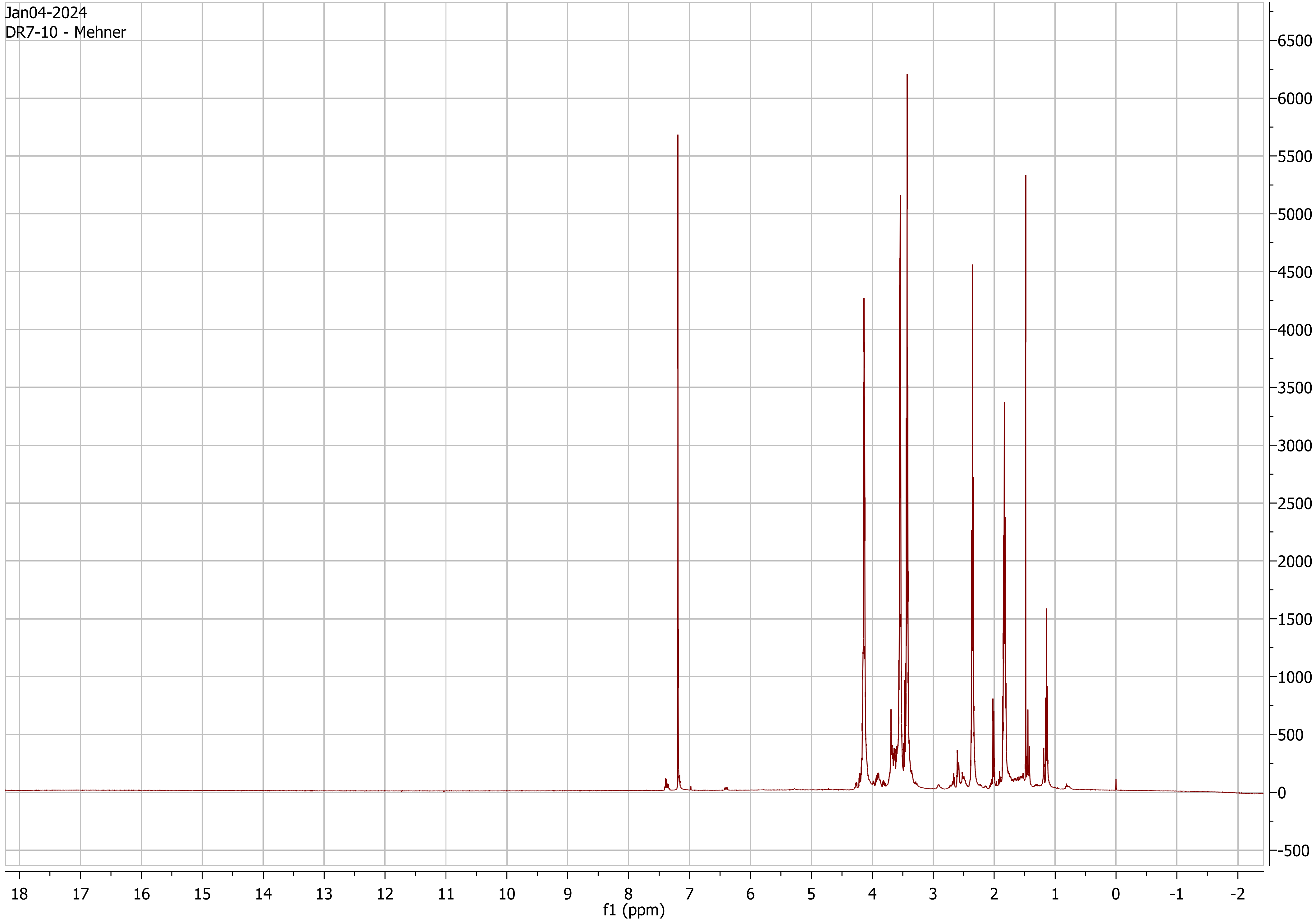
Jan04-2024
DR7-10 - Mehner
f1 (ppm)

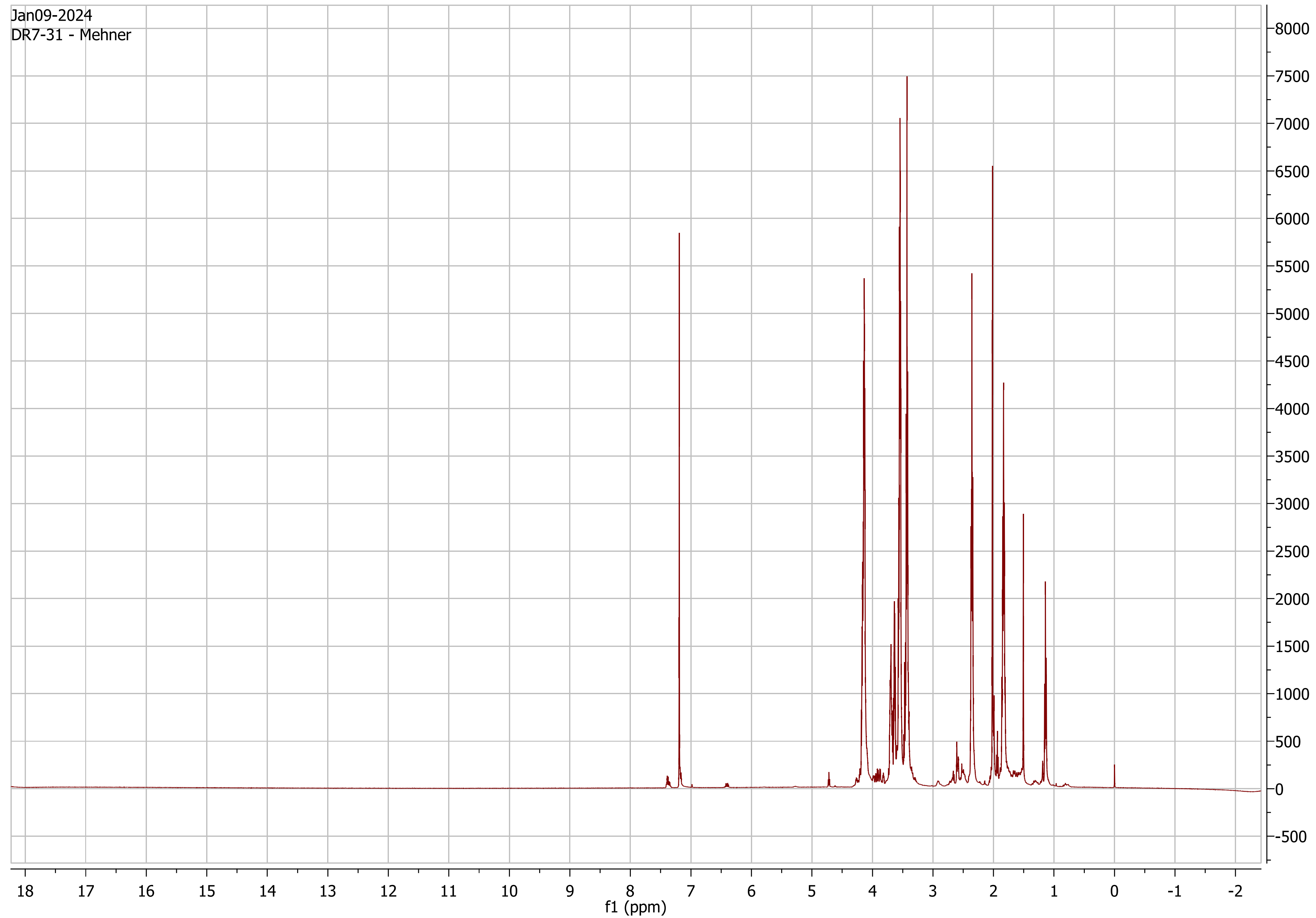

Jan09-2024
DR7-31 - Mehner
f1 (ppm)

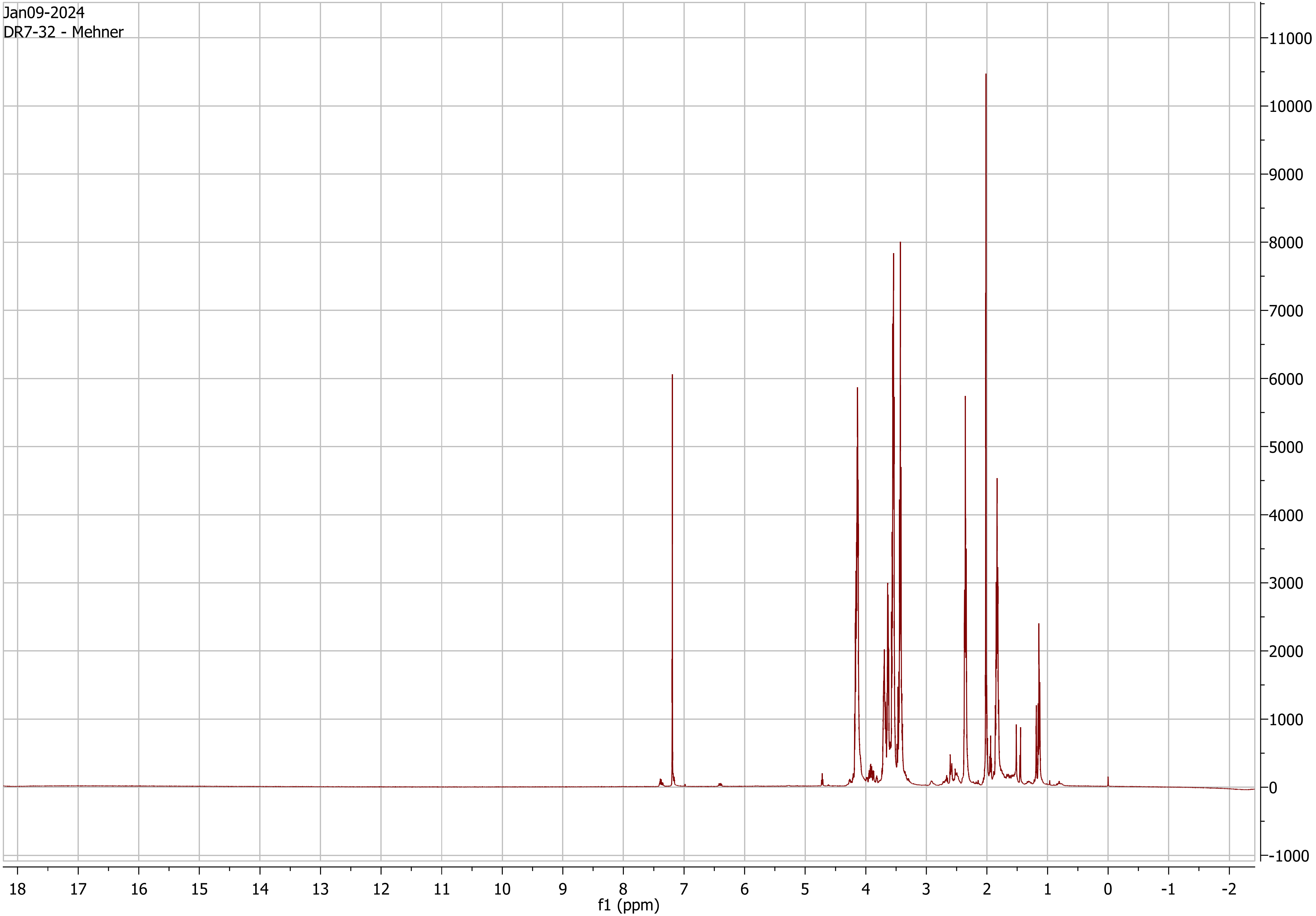
Jan09-2024
DR7-32 - Mehner
f1 (ppm)

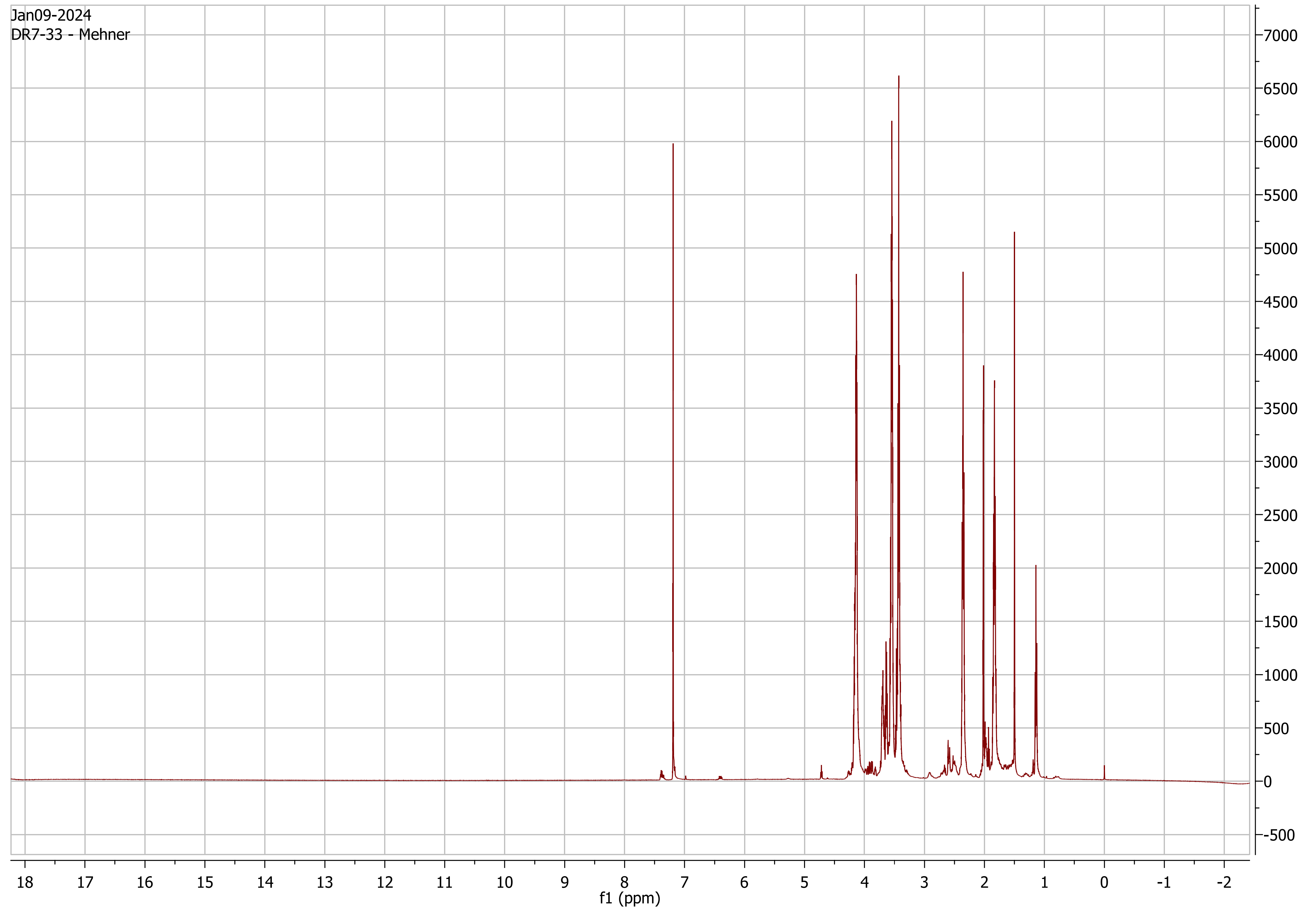
Jan09-2024
DR7-33 - Mehner
f1 (ppm)

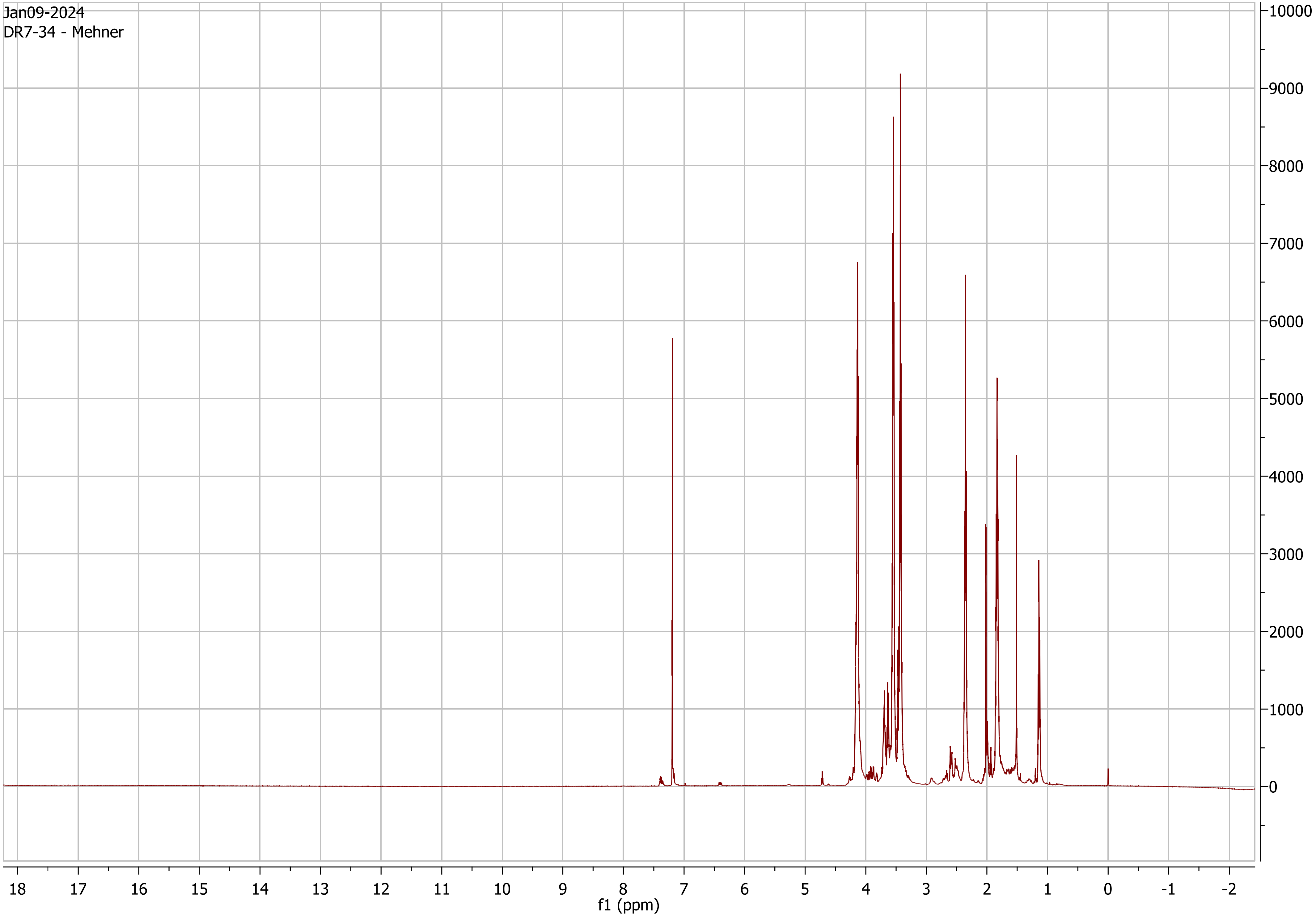
Jan09-2024
DR7-34 - Mehner
f1 (ppm)

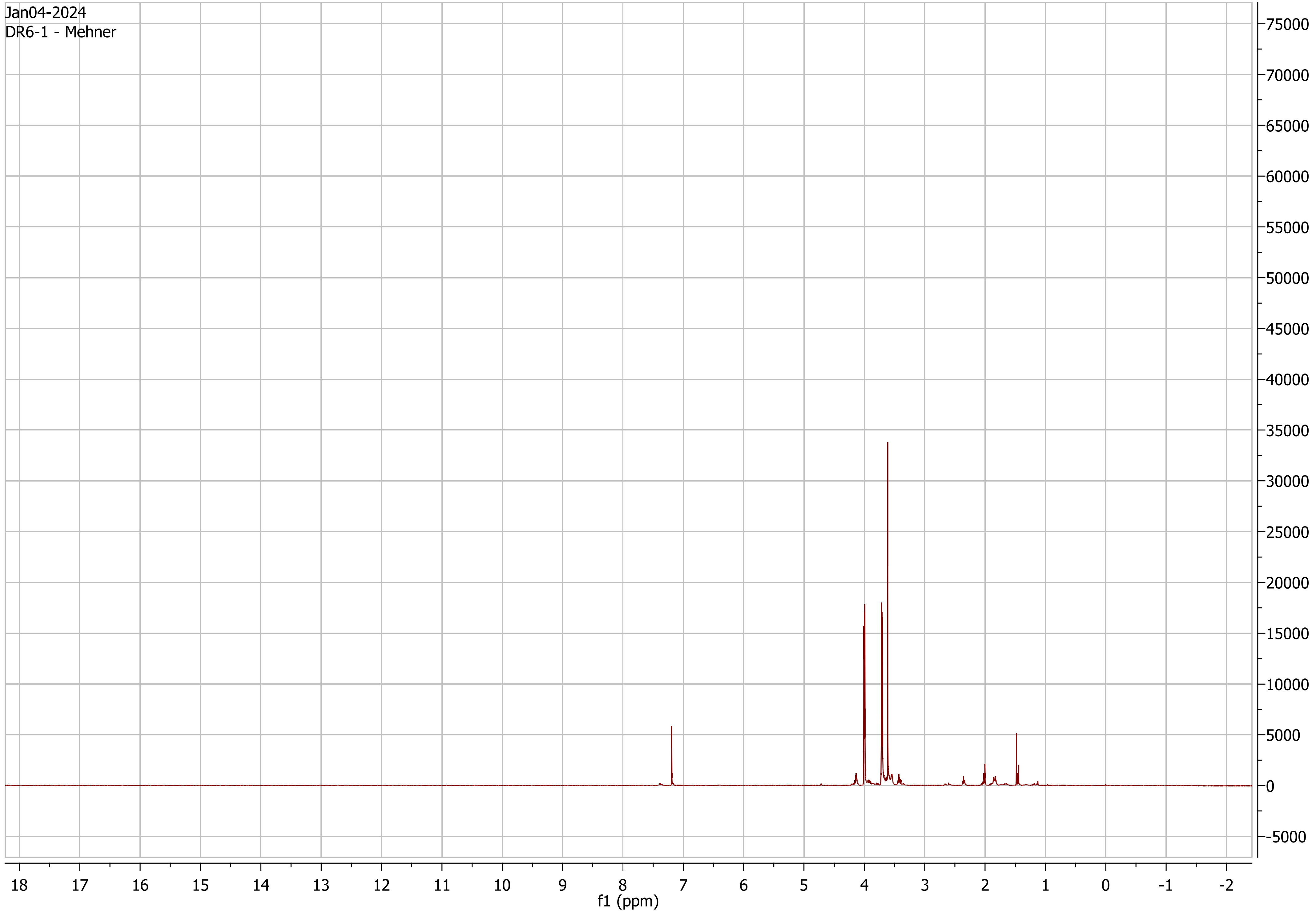
Jan04-2024
DR6-1 - Mehner
f1 (ppm)

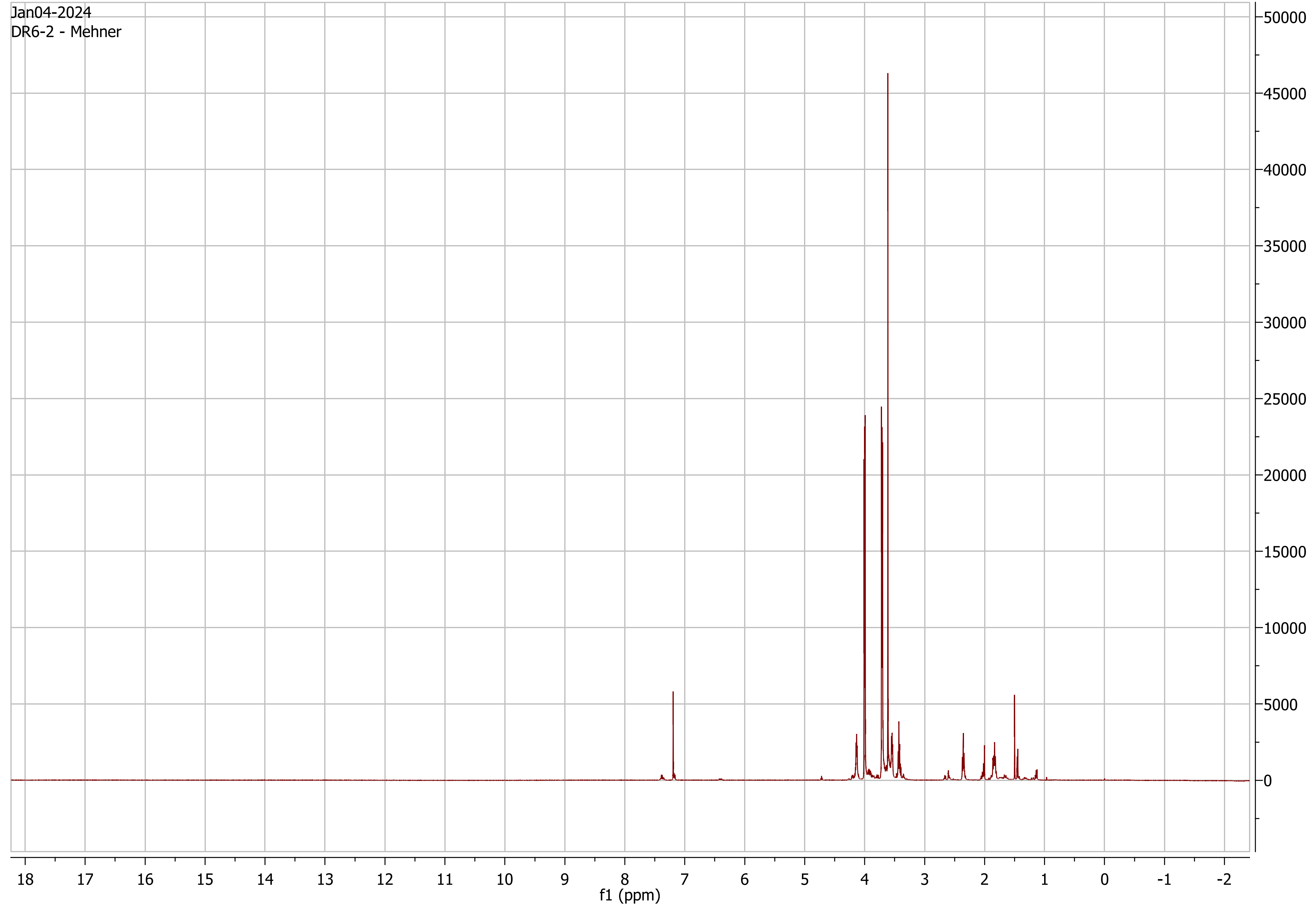
Jan04-2024
DR6-2 - Mehner
f1 (ppm)
18 17 16 15 14 13 12 11 10 9 8 7 6 5 4 3 2 1 0 -1 -2
50000 45000 40000 35000 30000 25000 20000 15000 10000 5000 0

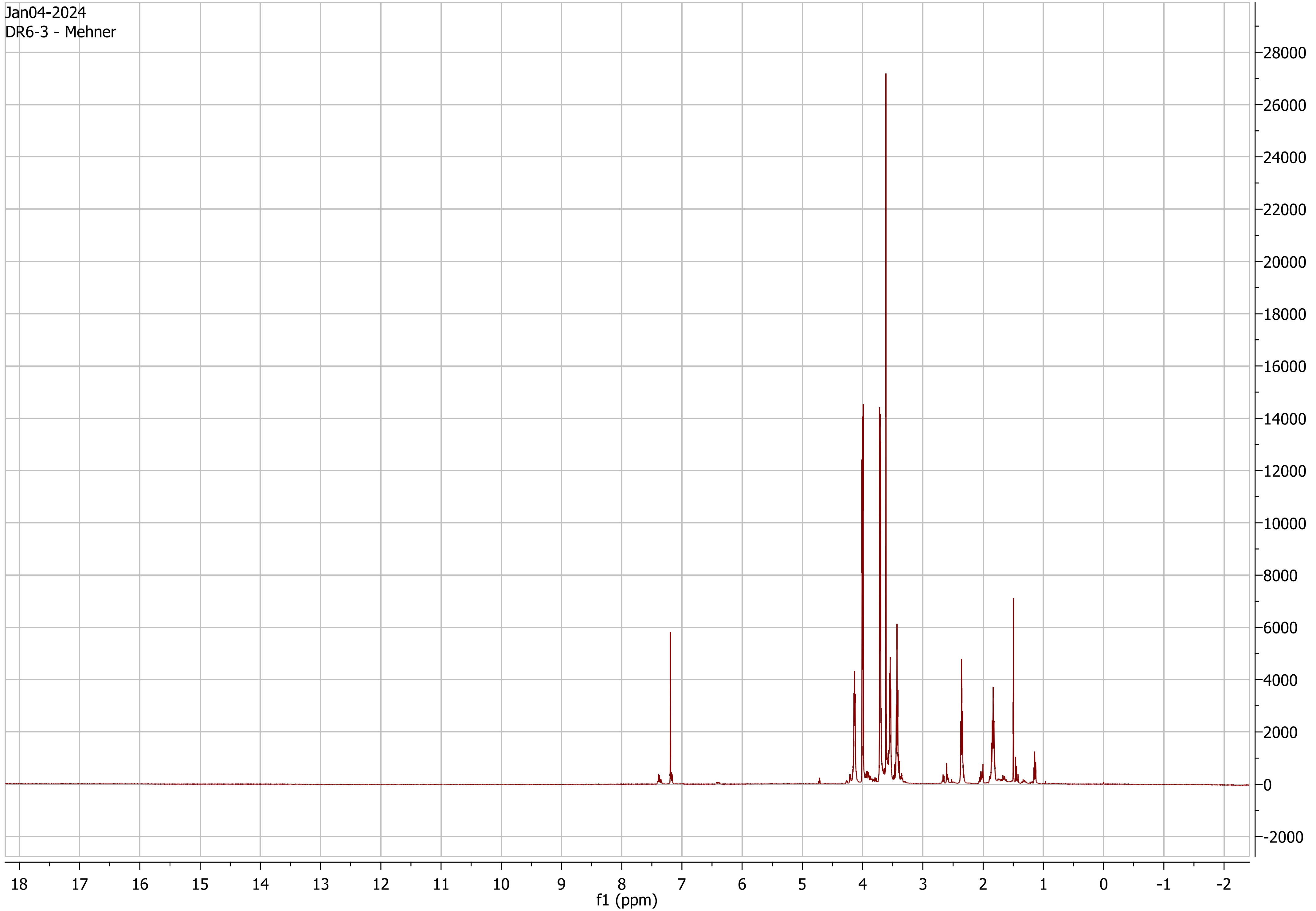
Jan04-2024
DR6-3 - Mehner
f1 (ppm)

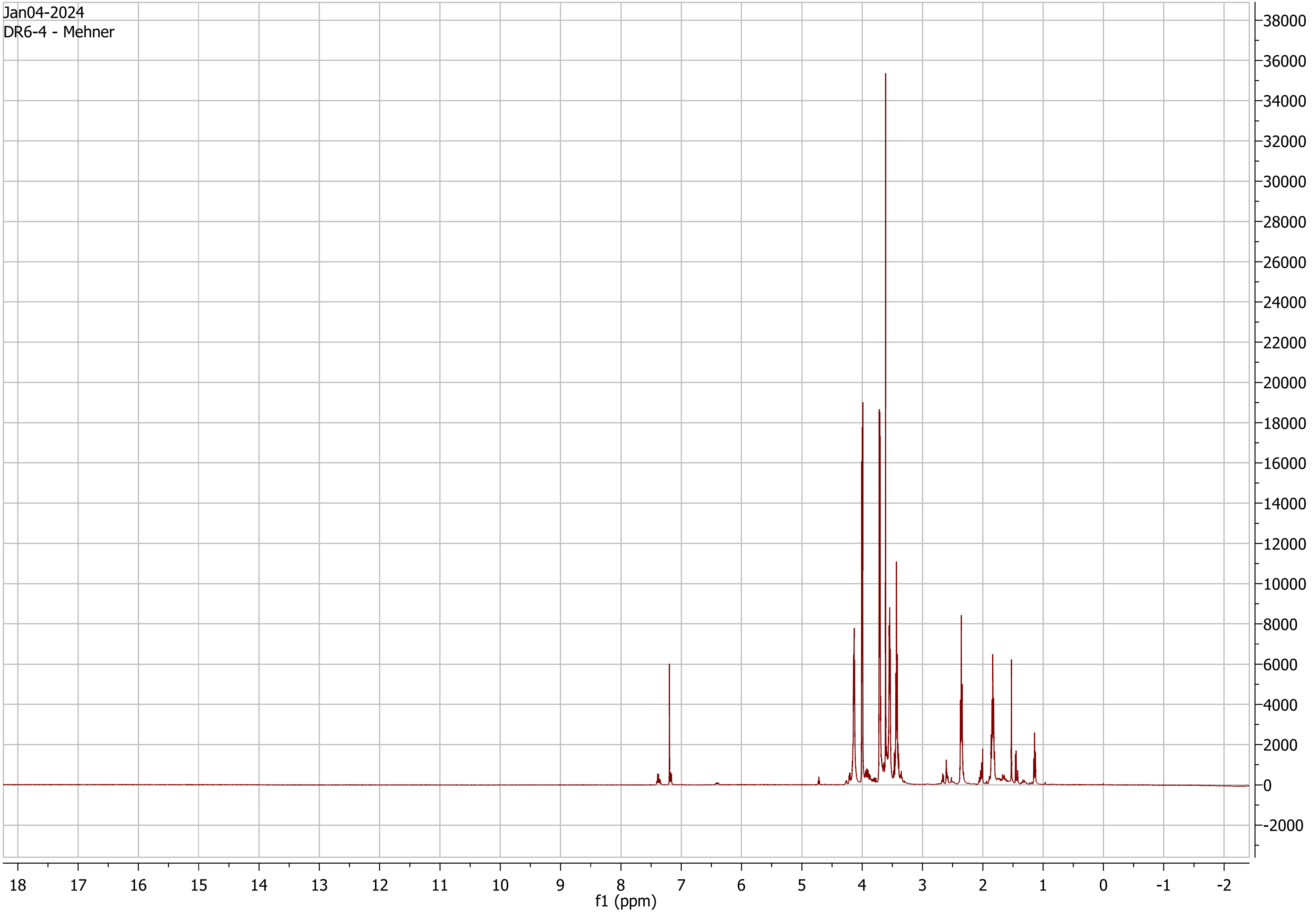

Jan04-2024
DR6-4 - Mehner
38000
36000
34000
32000
30000
28000
26000
24000
22000
20000
18000
16000
14000
12000
10000
8000
6000
4000
2000
0
-2000
18
17
16
15
14
13
12
11
10
9
8
7
6
5
4
3
2
1
0
-1
-2
f1 (ppm)

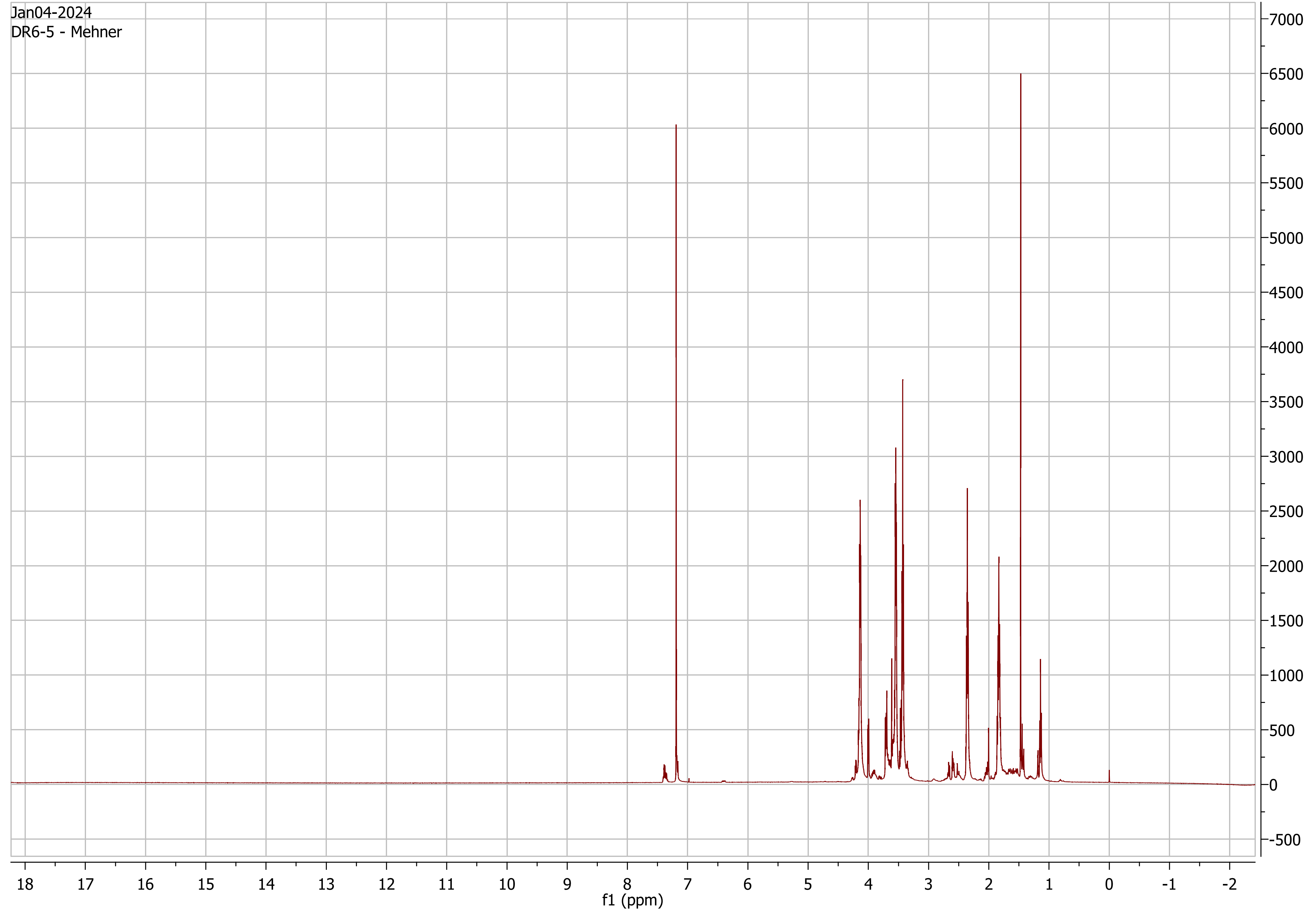
Jan04-2024
DR6-5 - Mehner
f1 (ppm)
18 17 16 15 14 13 12 11 10 9 8 7 6 5 4 3 2 1 0 -1 -2
7000 6500 6000 5500 5000 4500 4000 3500 3000 2500 2000 1500 1000 500 0 -500

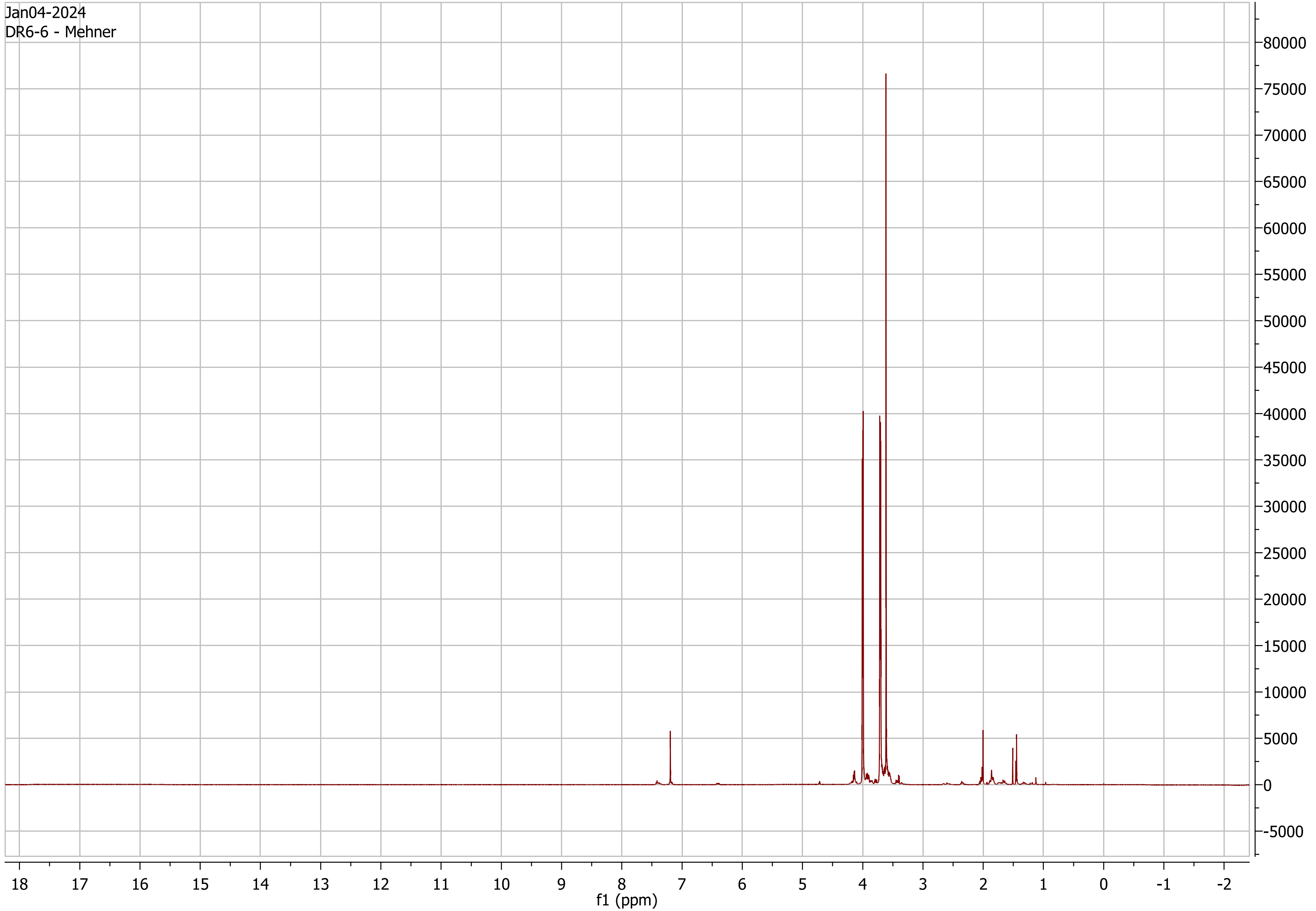
Jan04-2024
DR6-6 - Mehner
f1 (ppm)

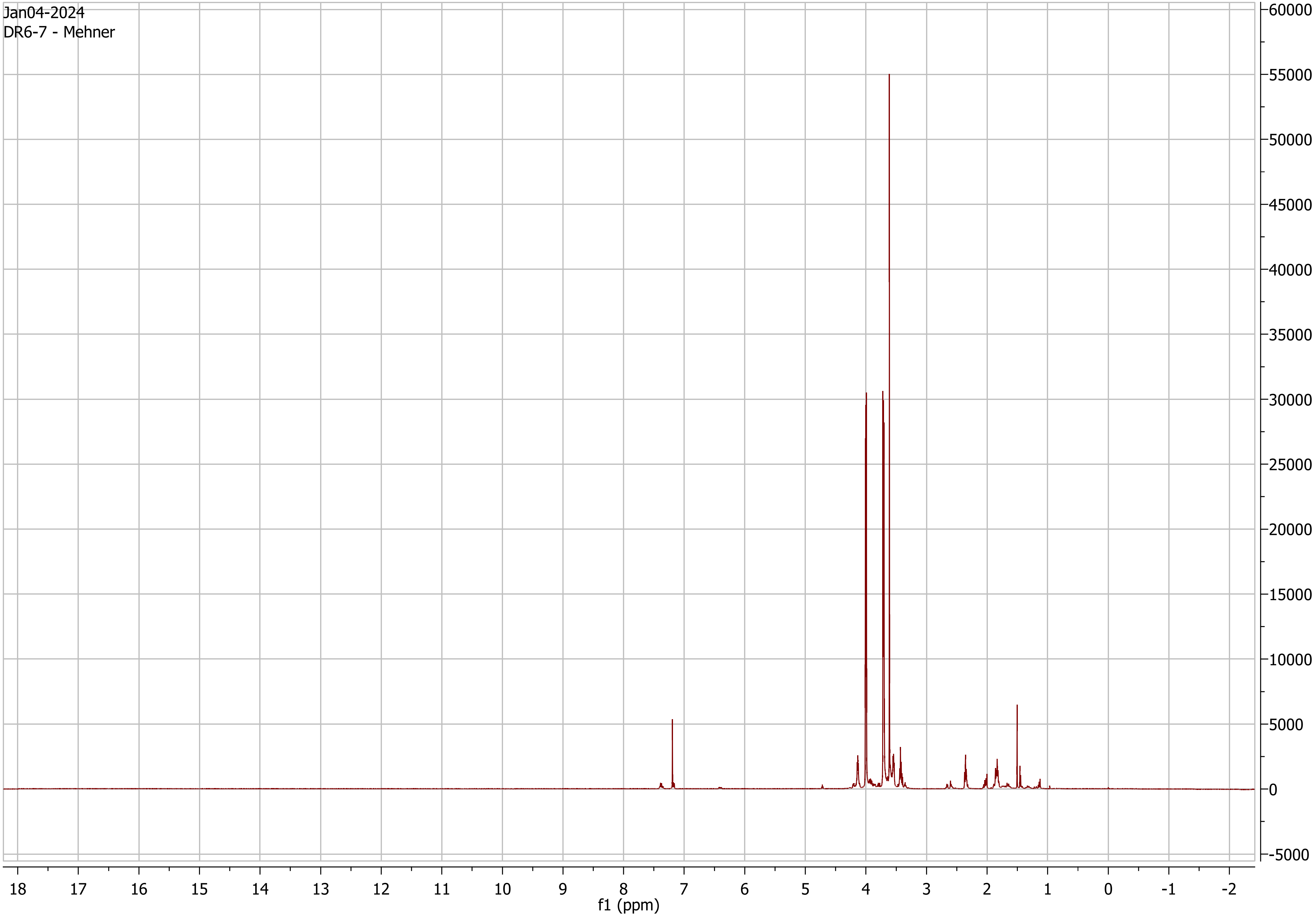
Jan04-2024
DR6-7 - Mehner
f1 (ppm)

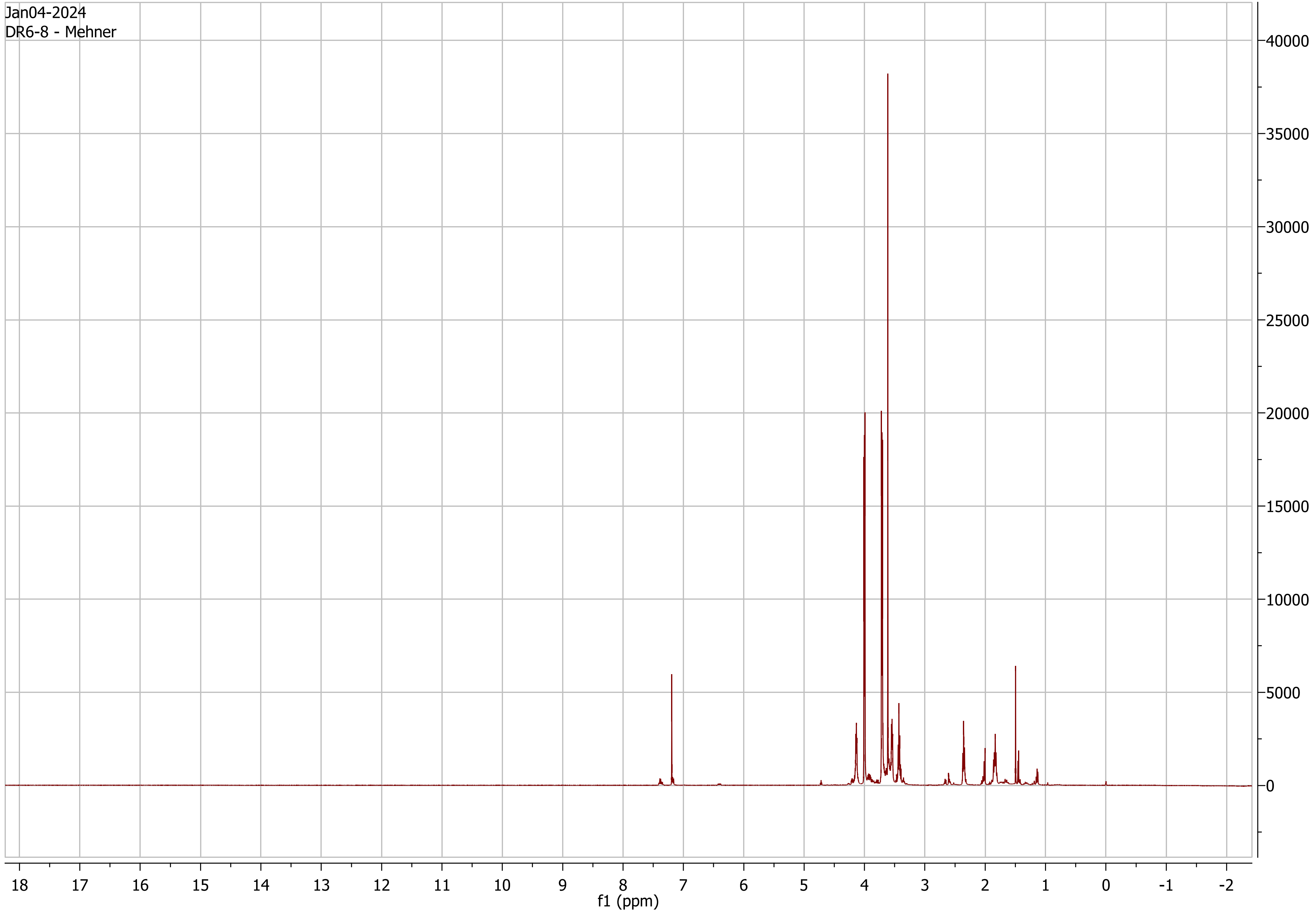
Jan04-2024
DR6-8 - Mehner
f1 (ppm)

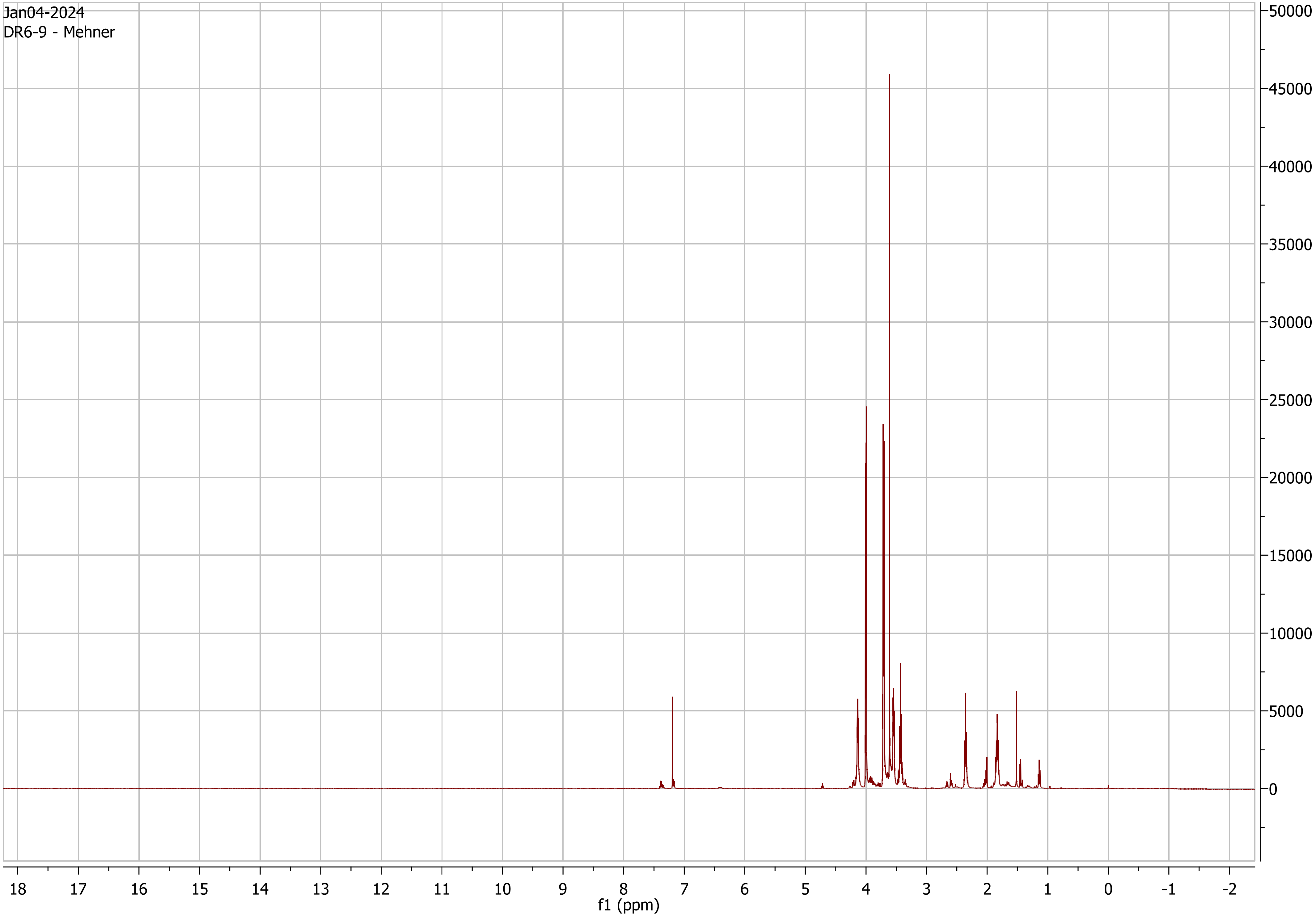
Jan04-2024
DR6-9 - Mehner
f1 (ppm)

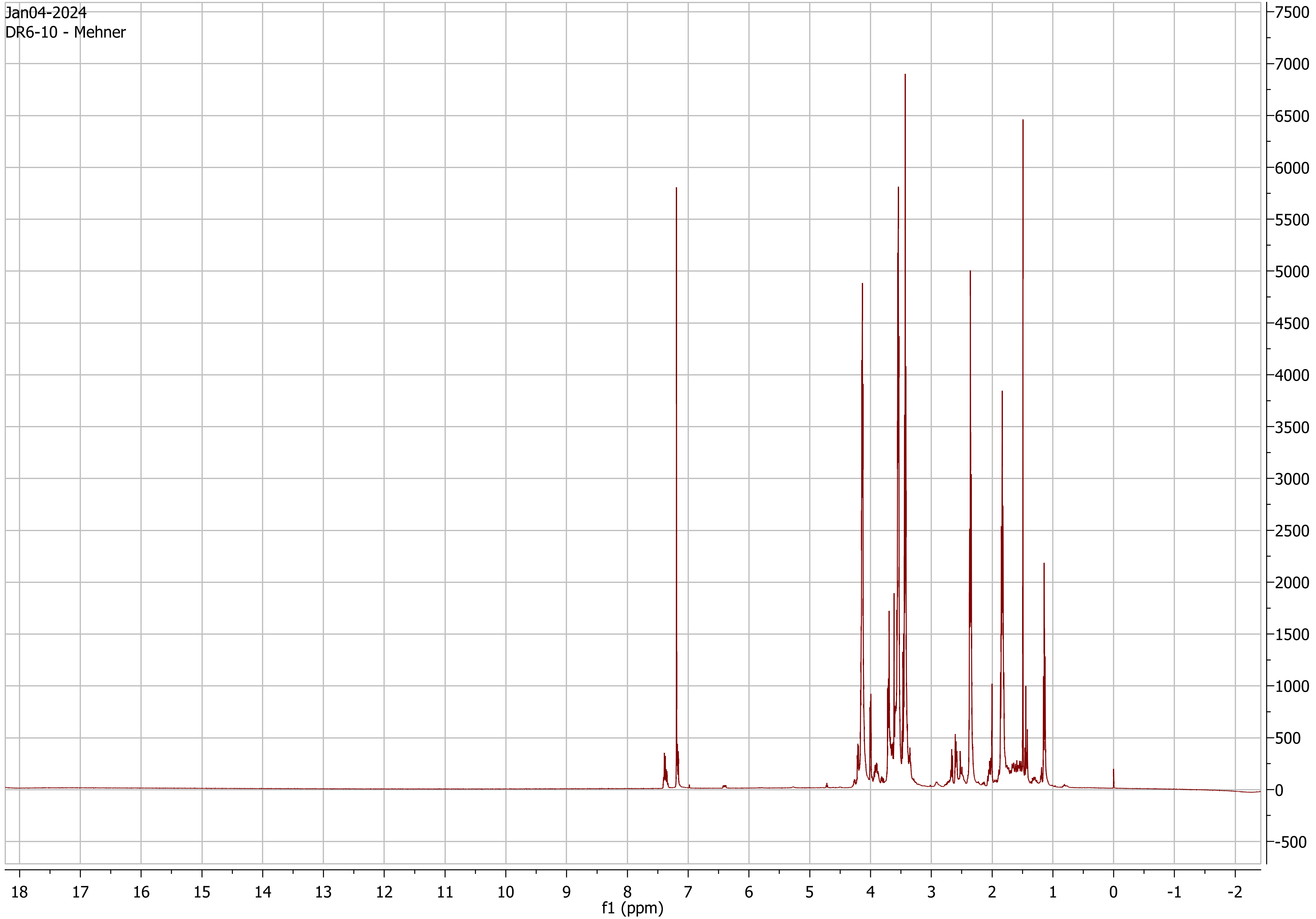

Jan04-2024
DR6-10 - Mehner
f1 (ppm)
18
17
16
15
14
13
12
11
10
9
8
7
6
5
4
3
2
1
0
-1
-2
7500
7000
6500
6000
5500
5000
4500
4000
3500
3000
2500
2000
1500
1000
500
0
-500

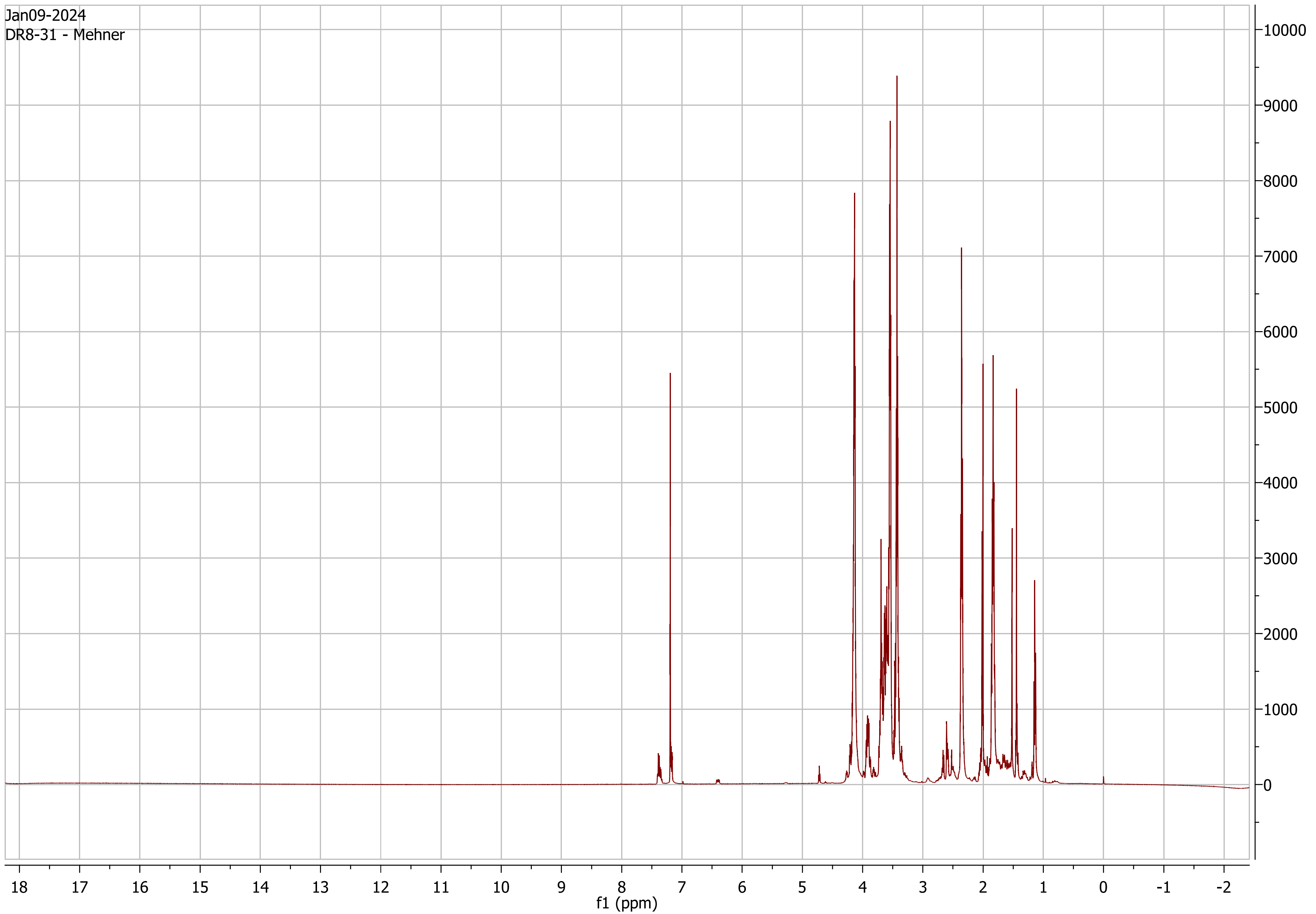
Jan09-2024
DR8-31 - Mehner
f1 (ppm)

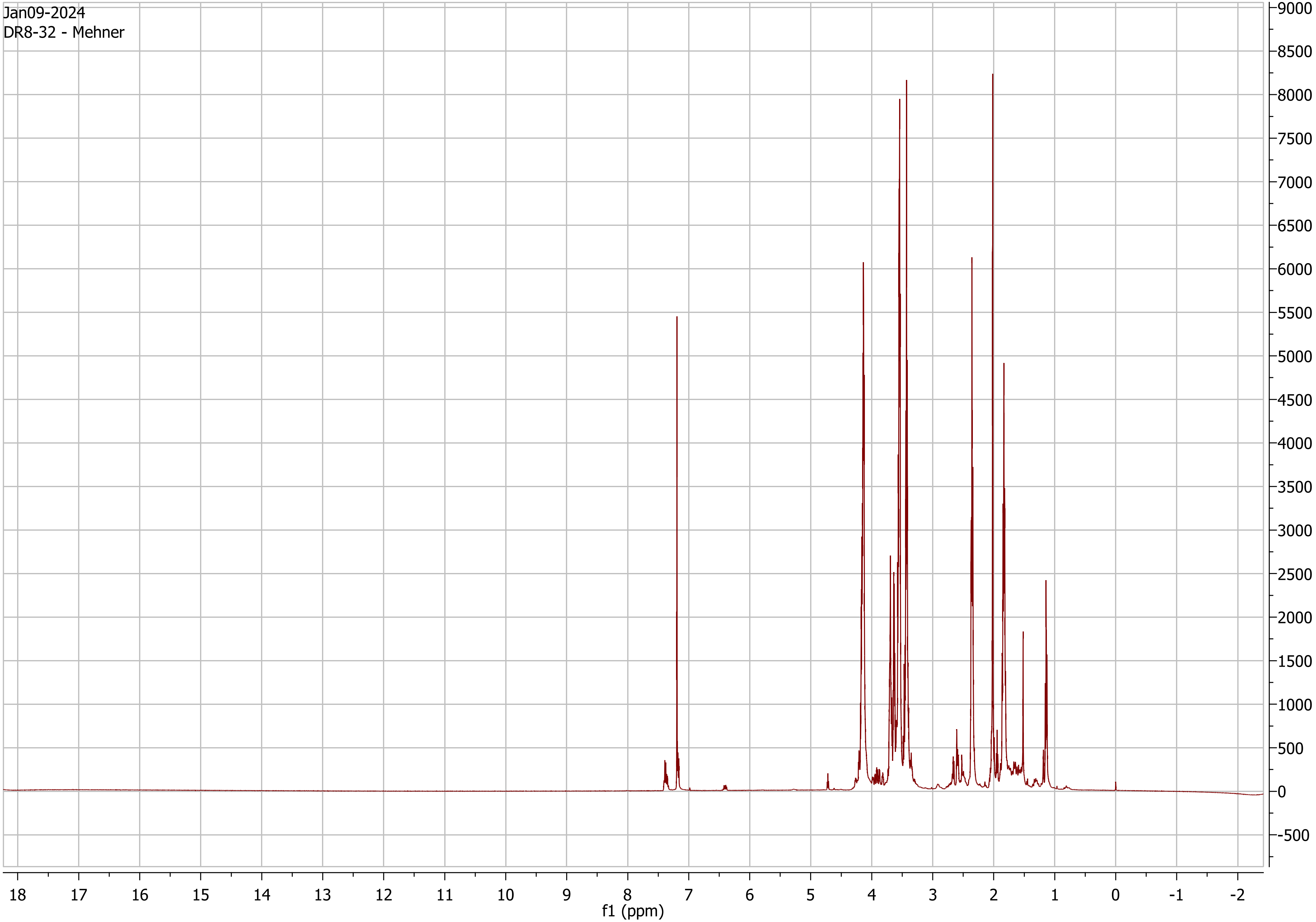

Jan09-2024
DR8-32 - Mehner
f1 (ppm)

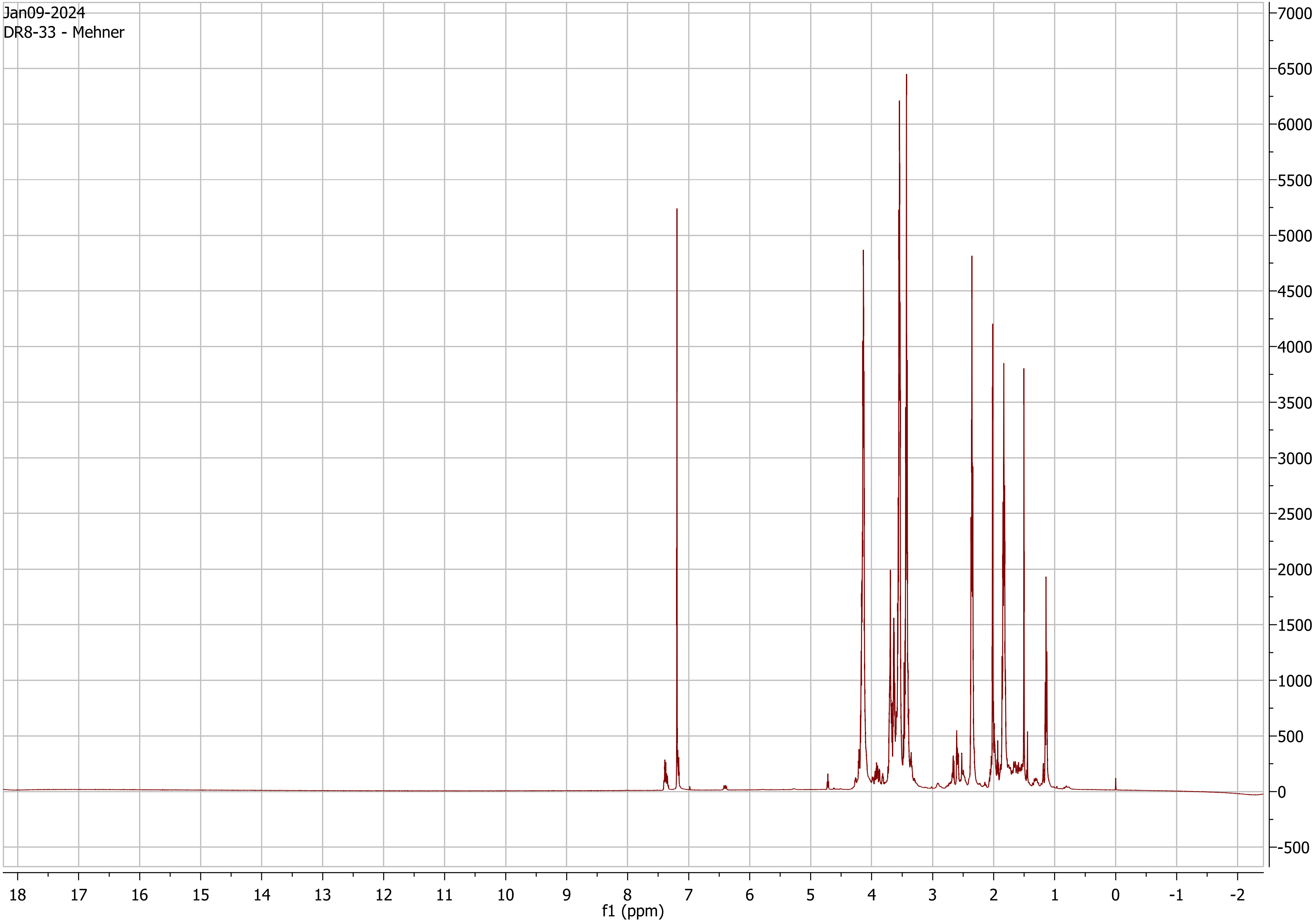

Jan09-2024
DR8-33 - Mehner
f1 (ppm)
18
17
16
15
14
13
12
11
10
9
8
7
6
5
4
3
2
1
0
-1
-2
7000
6500
6000
5500
5000
4500
4000
3500
3000
2500
2000
1500
1000
500
0
-500

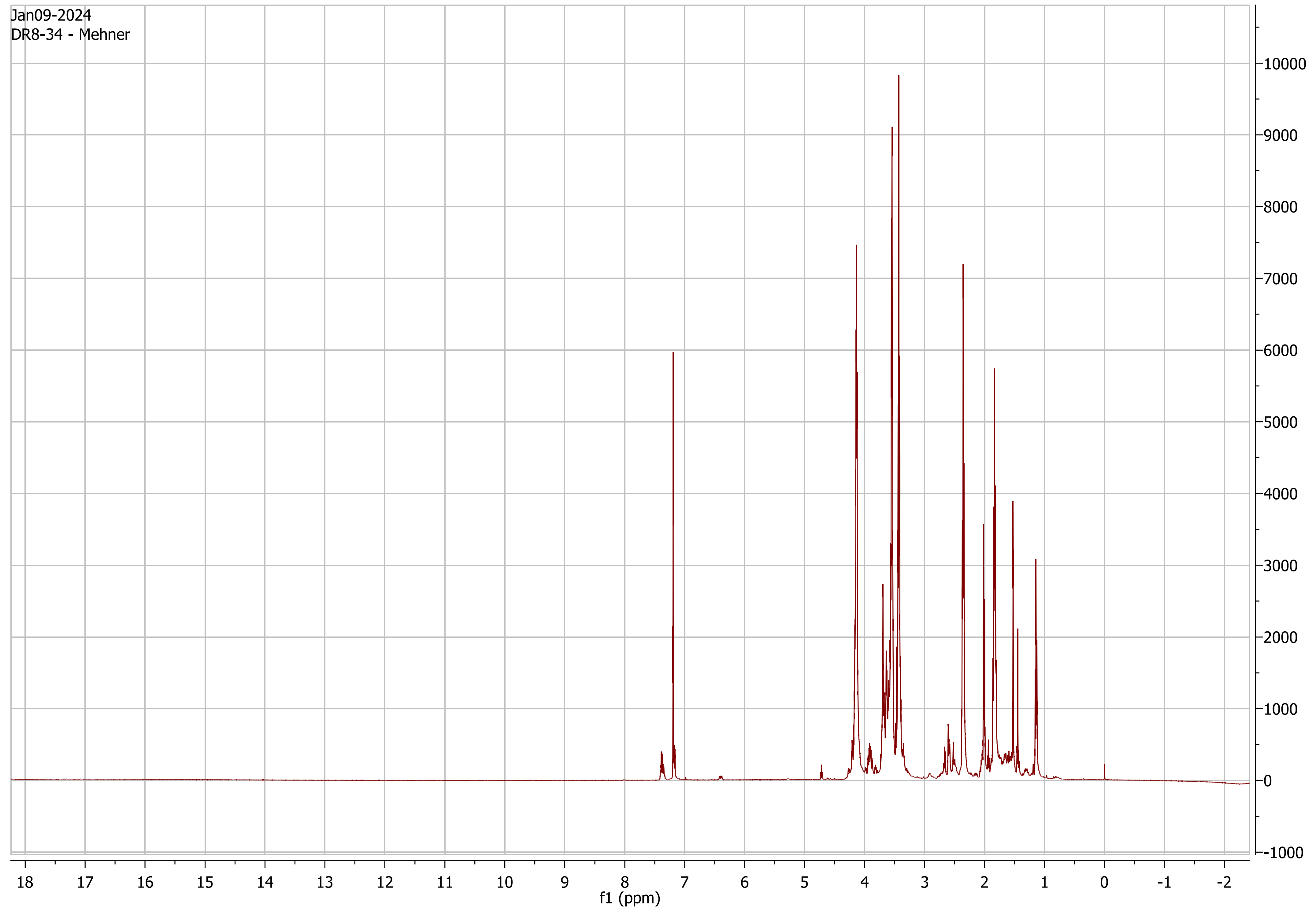

Jan09-2024
DR8-34 - Mehner
f1 (ppm)

May23-2024
DRR3-4-1 - Mehner

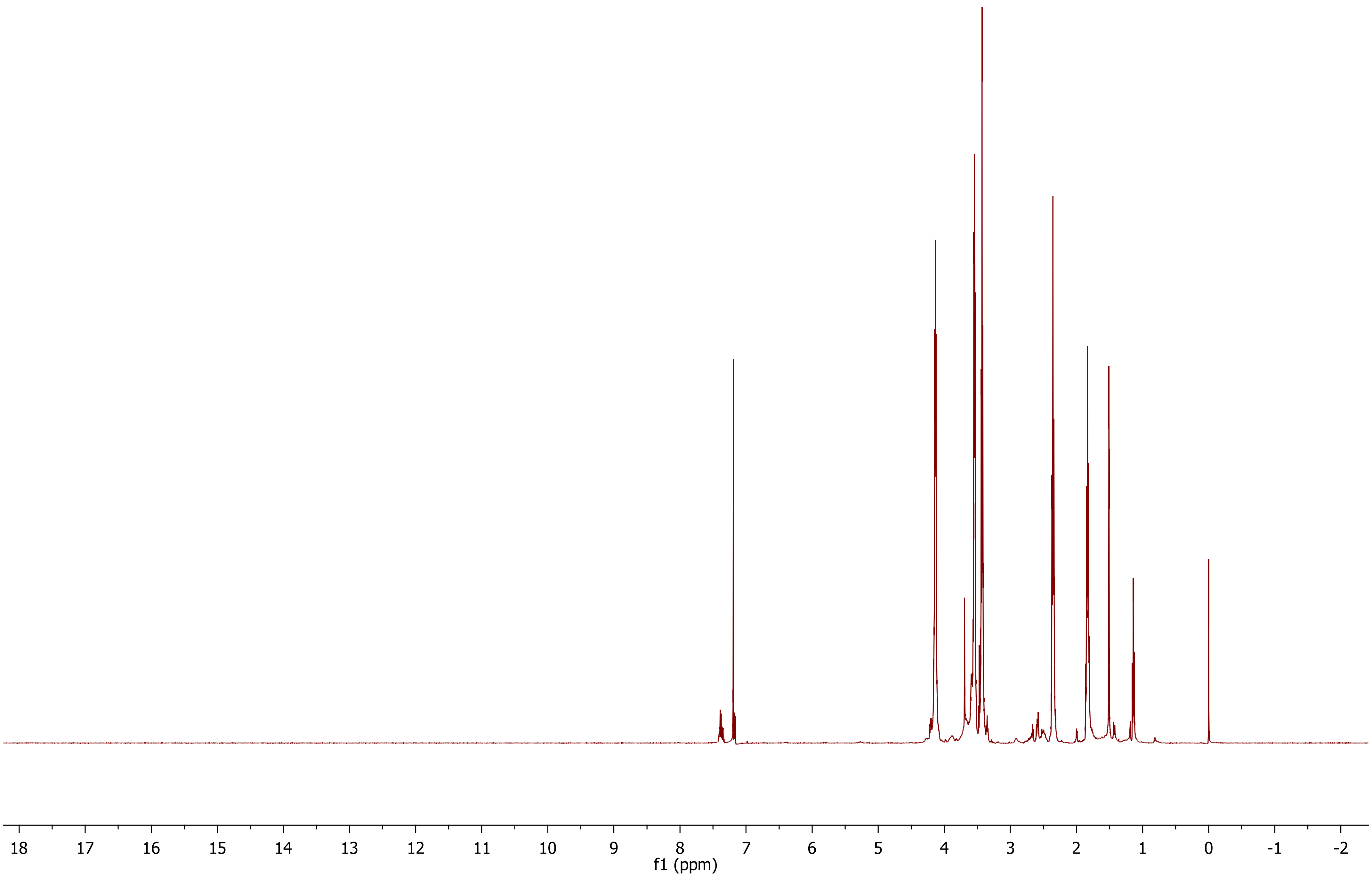

Supplement: Supplementary file 3 — Supplementary Data [file 42004_2026_1997_MOESM3_ESM.pdf]
